# Automated Library Generation and Serendipity Quantification Enables Diverse Discovery in Coordination Chemistry

**DOI:** 10.1021/jacs.2c11066

**Published:** 2023-01-17

**Authors:** Daniel J. Kowalski, Catriona M. MacGregor, De-Liang Long, Nicola L. Bell, Leroy Cronin

**Affiliations:** School of Chemistry, University of Glasgow, Glasgow G12 8QQ, United Kingdom

## Abstract

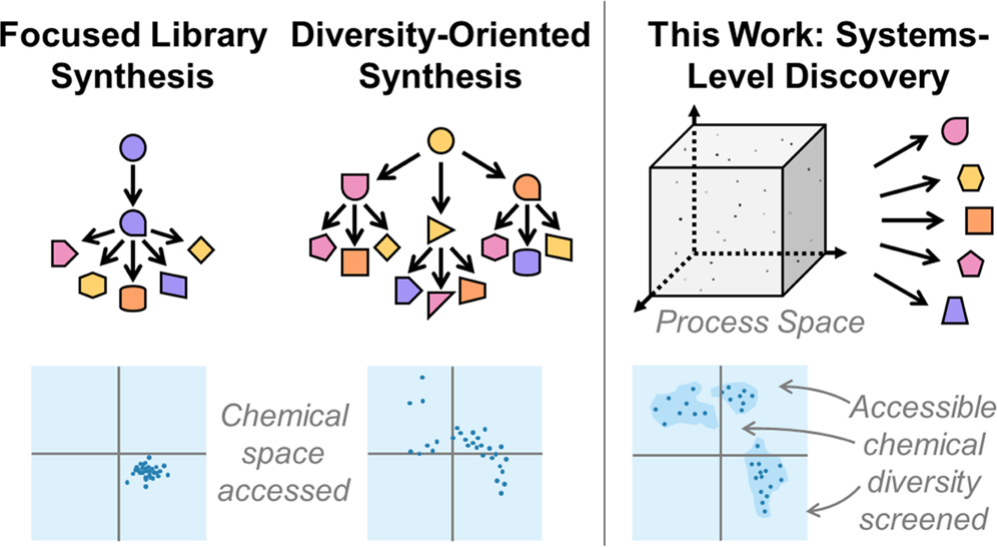

Library generation experiments are a key part of the discovery of new materials, methods, and models in chemistry, but the question of how to generate high quality libraries to enable discovery is nontrivial. Herein, we use coordination chemistry to demonstrate the automation of many of the workflows used for library generation in automated hardware including the Chemputer. First, we explore the target-oriented synthesis of three influential coordination complexes, to validate key synthetic operations in our system; second, the generation of focused libraries in chemical and process space; and third, the development of a new workflow for prospecting library formation. This involved Bayesian optimization using a Gaussian process as surrogate model combined with a metric for novelty (or serendipity) quantification based on mass spectrometry data. In this way, we show directed exploration of a process space toward those areas with rarer observations and build a picture of the diversity in product distributions present across the space. We show that this effectively “engineers” serendipity into our search through the unexpected appearance of acetic anhydride, formed *in situ*, and solvent degradation products as ligands in an isolable series of three Co(III) anhydride complexes.

## Introduction

Our ability to design target chemical species or synthetic routes (usually via target-oriented synthesis, TOS^1^) is predicated on previous knowledge, including models that attempt to explain patterns in chemical reactivity. New data is key to the development of materials, methods, or models, and serendipity often plays a large part in this process.^2,3^ Methods that “engineer” serendipity into the chemical discovery process are of interest but hard to achieve when developing reliable approaches for library generation, for example.^4−7^ Despite this, library generation is a powerful tool for gathering relevant data for pattern finding. It is possible to classify^8^ libraries as either “Focused” or “Prospecting,” depending on the region of chemical space accessed by members. Focused libraries are highly useful for establishing structure–activity relationships (SAR) for complex molecules; however, the diversity that can be generated through variations on the same retrosynthetic process is restricted.^8^

Prospecting libraries^8^ offer an alternative focus, with an increased possibility for serendipity and diversity-oriented synthesis (DOS), which aims to develop forward synthesis methodologies toward complex molecules, is excellent at generating diverse scaffolds for medicinal applications.^1,9,10^ For example, the approach of “Build/Couple/Pair” introduces diversity by mimicking the logic of biosynthetic pathways.^11−13^ However, DOS is heavily reliant on multicomponent or cascade transformations, of which there are only a limited number. In addition, DOS may not always add to the understanding or development of synthetic chemistry.^10^

**Figure 1:**
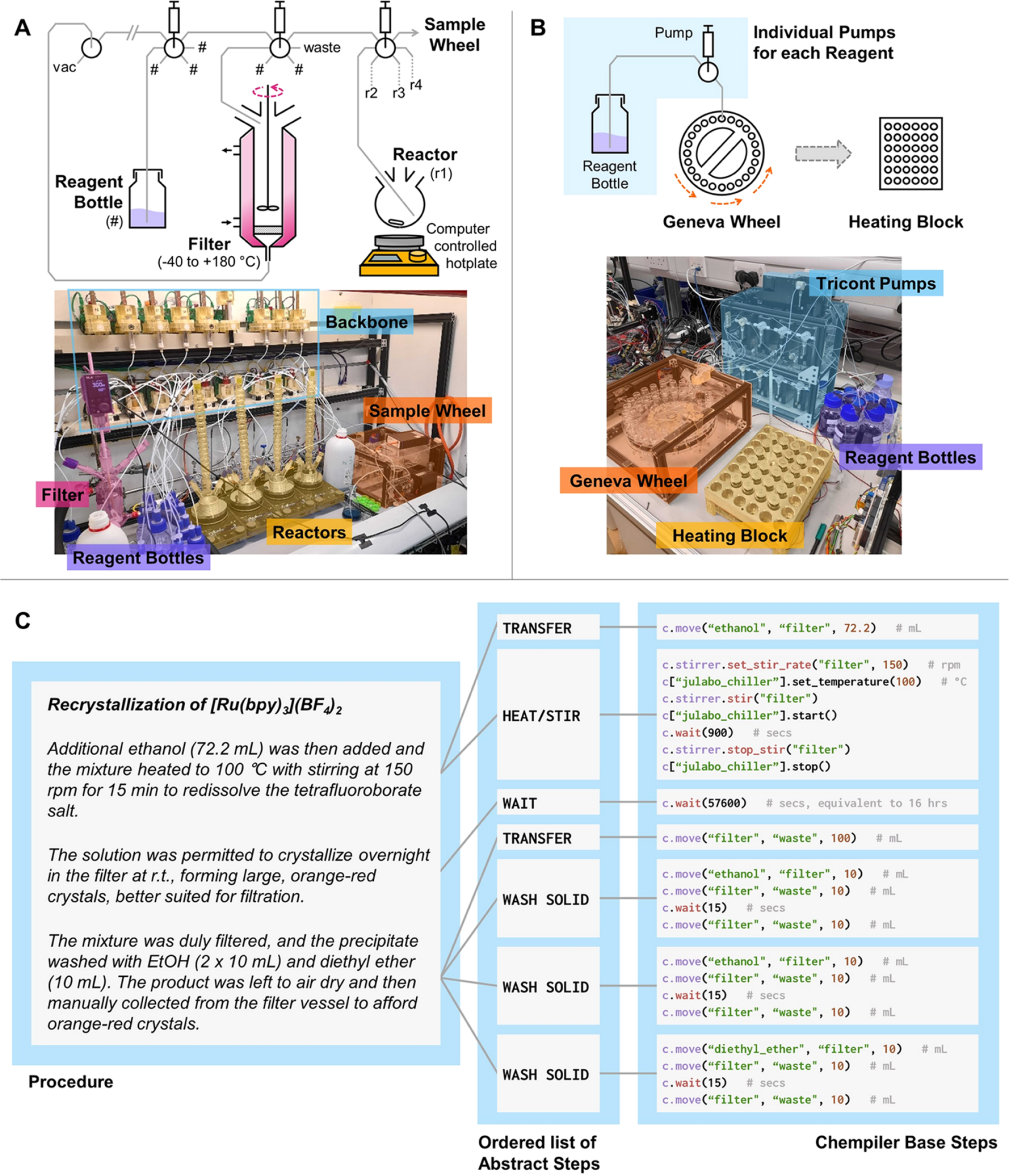
Chemical robots and coding logic used in this work. Simplified schematics and photographs are shown of the (A) Chemputer Platform with filter module and parallel reactors, and (B) Geneva Wheel Platform. Pump/valve combinations not accounted for in the schematic of the Chemputer are loaded with additional reagent bottles. The GWP schematic shows only a single example of the 10 pumps explicitly. (C) Overview of the structure by which automated syntheses are translated into code. Literature synthesis is broken into a series of abstract steps, to be executed in a certain order, which are then further broken into fundamental commands that can be communicated to the platform via the Chempiler package.

The development and application of automation and data science techniques have already enabled the expedience, miniaturization, and interpretation of library-generating methodologies,^14,15^ but this is often focused on a limited number of well-defined processes. In this work, we apply a universal synthesis platform^16^ to library generation and implement both algorithmic exploration and data science techniques to introduce a new, generally applicable workflow for prospecting library generation; see Figure 1.

We chose to apply these techniques to coordination chemistry since the highly reconfigurable and difficult-to-predict nature of these reactions,^17^ particularly those in multinuclear coordination space, make this area particularly unsuitable for DOS forward synthesis methods and thus reliant on serendipity.^2,18^ We address these aims by splitting the work into three phases: (i) target-oriented synthesis (TOS) of a range of coordination complexes for the validation of synthetic operations required for coordination chemistry in a universal synthesis platform, (ii) automated focused library generation, and (iii) automated generation of a prospecting library through algorithmically directed screening for chemical diversity within a process space of multinuclear species.

## Results and Discussion

### Key Synthetic Operations for Coordination Chemistry

The Chemputer is designed to automate liquid handling nondeterministically, meaning that chemical operations may be carried out by available modular hardware provided it is able to perform the required unit operations, regardless of topology.^19^ The minimal number of modules necessary for classical coordination chemistry included here are a temperature-controlled jacketed filter module, reactor modules (i.e., RBFs), and a sample storage carousel.

This work used previously developed Chemputer modules in a topology new to synthesis (Figure 1).^20−23^ Using multiple reactor modules permitted the parallelization of synthetic preparations, which could then be dispensed to vials held in the storage carousel to allow for multiple cycles of reactions before human intervention was required. Prospecting library generation used a higher-throughput setup^24^ based around the Geneva wheel sample storage carousel (Figure 1). To run chemical syntheses, abstracted unit operations are used to build up a codified form of any given literature procedure as described previously.^25^ The hardware is represented as a graph with nodes corresponding to individual modules (e.g., reactor, filter, input flask), and edges to the physical connections between these modules. The abstracted steps are then mapped onto the hardware to allow the same synthesis to be executed on multiple different platforms. The setup and code for all of the syntheses performed here are provided in the Supporting Information (SI), or associated GitHub repository.

### Exemplar Target-Oriented Syntheses

**Figure 2:**
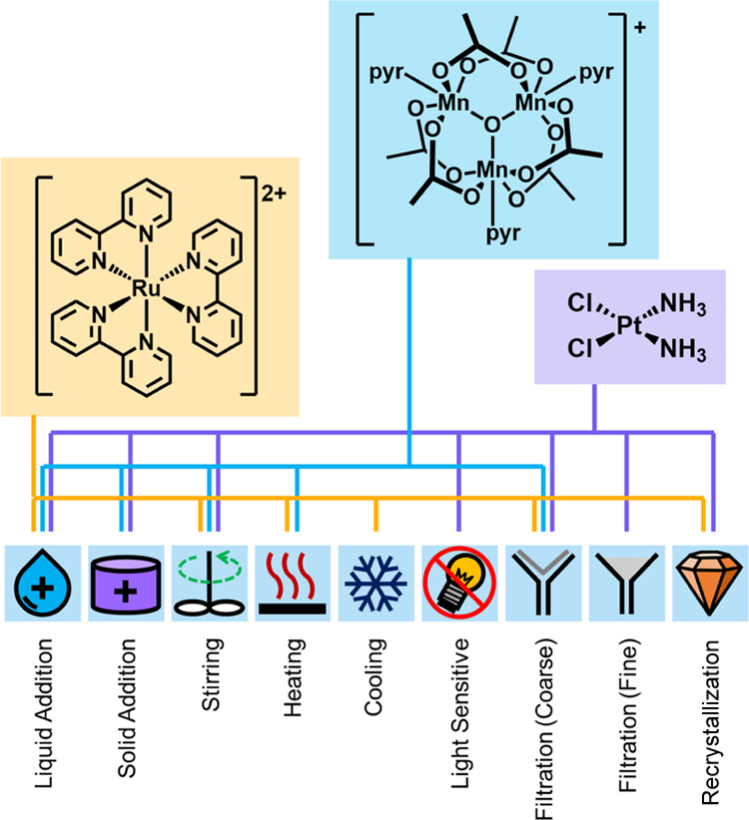
Synthetic operations common across coordination chemistry and their relation to the automated target-oriented syntheses conducted in this work. Coarse filtration uses the glass frit built into the jacketed filter module, and fine filtration uses a Thermo Scientific bottom-of-the-bottle filter suspended in the reactor module.

To validate the utility of our universal synthesis platform for inorganic chemistry, three complexes were selected as targets, encompassing a range of applications, synthetic complexity, and operations common to coordination chemistry (Figure 2). [Ru(bpy)_3_](BF_4_)_2_ is commonly used for photochemical applications^26−28^ and was synthesized in two steps: first, the ligand substitution of ruthenium(III) chloride hydrate with 2,2′-bipyridine, via a Ru(bpy)_2_Cl_2_ intermediate, and second, the subsequent anion metathesis to afford the tetrafluoroborate salt. Precipitation yields a fine, difficult-to-handle material, which was recrystallized from ethanol to yield higher-quality single crystals. The product was afforded in a yield of 26%, in comparison to 40% manually (without recrystallization).

[Mn_3_O(OAc)_6_(pyr)_3_]ClO_4_, {Mn_3_O}, has been widely explored in the literature as a model system in bio-inorganic chemistry,^29^ as a synthetic building block for molecular materials,^30,31^ and as a stoichiometric reagent.^32,33^ Tetrabutylammonium permanganate, *n*-Bu_4_NMnO_4_, must be added as a solid, due to rapid degradation upon dissolution in ethanol. Two sequential solid additions were achieved through preloading the jacketed filter with *n*-Bu_4_NMnO_4_ and a reactor module with solid manganese(II) acetate. In the automated procedure, the manganese(II) acetate was first dissolved in ethanol with stirring, then combined with acetic acid and pyridine, and this mixture was transferred to the jacketed filter to initiate the conproportionation with permanganate. Given the presence of four reactor modules in addition to the jacketed filter, this process could be repeated to allow sequential addition of up to five solid reagents. The final product is precipitated via anion metathesis with NaClO_4_ to give a yield of 49%, in comparison to 21% manually.

Cisplatin is an anticancer therapeutic and of great significance to the development of inorganic medicinal chemistry.^34,35^ Our automated procedure was conducted according to a variation of Dhara’s method^35,36^ presented by Boreham *et al*.^37^ Accessing the *cis* geometry is nontrivial, and is assured through conversion of the tetrachloroplatinate starting material to tetraiodoplatinate, where the stronger *trans*-effect of the iodo ligand promotes *cis*-substitution. The remaining iodo ligands are then removed through reaction with AgNO_3_, and replaced with chloride. As AgNO_3_ is photosensitive, the exclusion of light was achieved by covering the reaction vessel prior to initiating the reaction. Solid AgNO_3_ was added to this occluded reactor, along with the platinate starting material, to remove the need for storage and transference of a light-sensitive solution through the backbone. The fine precipitate of AgI generated on reaction contaminated the product in initial runs and required the addition of a disposable in-line filter through which the reaction mixture was passed to reach the backbone. Cisplatin was collected with a yield of 30%, in comparison to 43% manually.

In addition to the coordination complexes, both metastable polymorphs of calcium carbonate—vaterite and aragonite—were prepared from a common pool of reagents.^38^ This system is relatively sensitive to the synthetic environment and will readily convert to the thermodynamically stable calcite polymorph if not appropriately controlled. These syntheses speak to the fine control of temperature, stir rate, and addition rate, and to the meticulousness of filtration in the Chemputer. These aspects are likely to be advantageous in classical coordination chemistry, particularly during sensitive processes such as recrystallization.

### Focused Library Generation

With the synthetic operations validated, we next explored the development of noniterative focused libraries via two methods: (i) combinatorial reaction of a series of ligands with a metal complex and (ii) screening a range of values for a key synthetic parameter.

**Figure 3:**
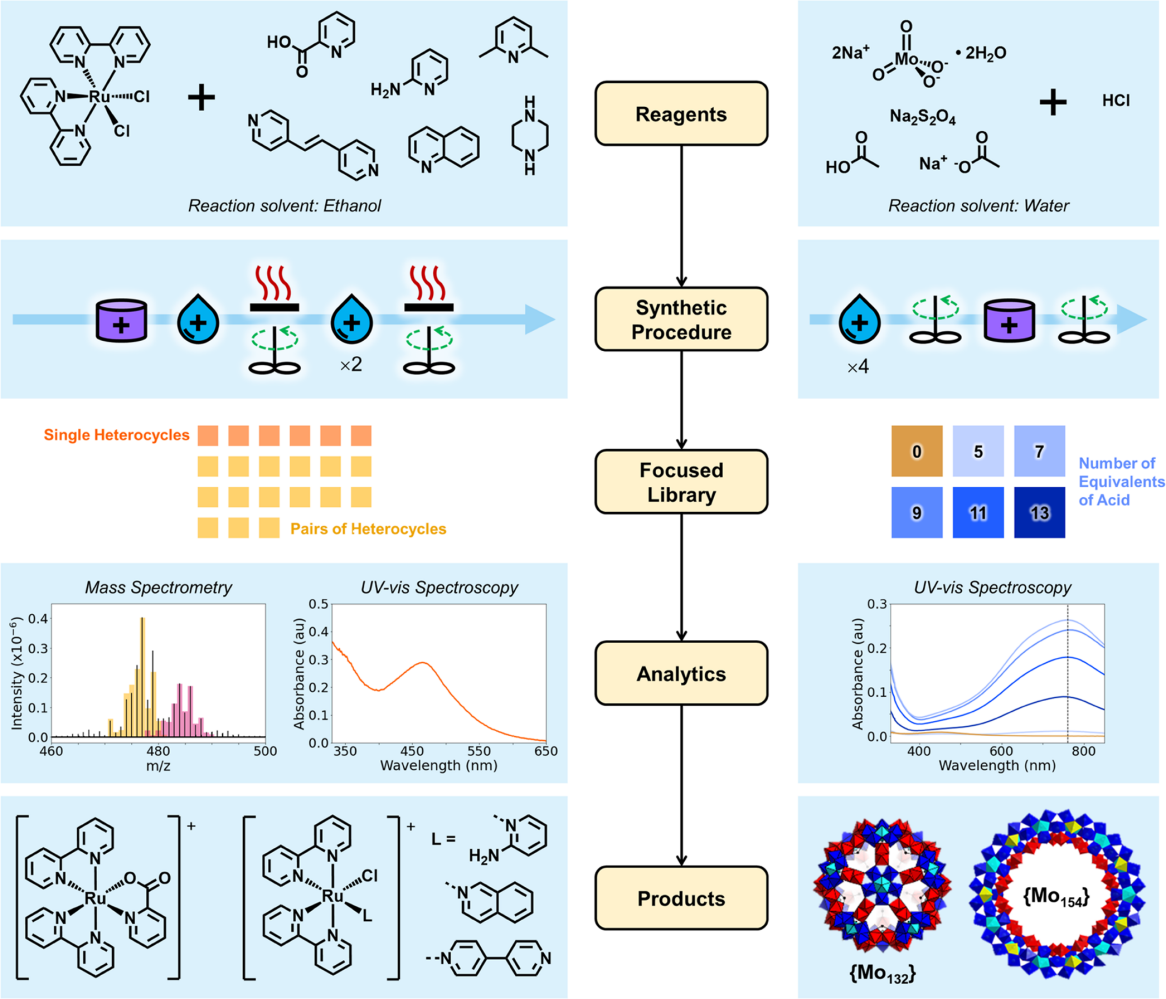
Workflow for the noniterative generation of focused libraries in the Chemputer. (Left) A library in chemical space of Ru(bipy)_2_Cl_2_ derivatives. (Right) A library in process space of reaction compositions leading to the formation of polyoxomolybdates. In each case, the reagents, synthetic procedure, library construction, methods of analysis, and isolated products are shown. Polyoxomolybdate polyhedra are colored according to the structural building blocks represented: {Mo{Mo_5_}}, cyan and blue; {Mo_1_}, yellow; {Mo_2_} in red. Note that {Mo_2_} building blocks in {Mo_132_} are formed from edge-sharing octahedral, and corner-sharing in {Mo_154_}.

Combinatorial production of focused molecular libraries by varying reagent combinations is key to determining structure–activity relationships.^8^ For this reason, the lead-optimization phase of medicinal discovery chemistry often involves late-stage combinatorial variation of pendant structural features attached to a core scaffold.^39^ As such, we automated the generation of a focused library via a combination of the Ru(bpy)_2_Cl_2_ intermediate from the synthesis of [Ru(bpy)_3_]^2+^ with a set of six *N*-heterocycles. Products were analyzed via MS and UV–vis spectroscopy (Figure 3, left).

Ru(bpy)_2_Cl_2_ was synthesized autonomously in the jacketed filter as previously and redissolved to serve as the metal precursor stock. The four reactors were operated in parallel, and the resultant product mixtures were transferred to the sample storage carousel. The reactor modules were then cleaned, and the next set of reactions was attempted (see the SI). A sequence of 20 reactions was shown to run consecutively without the need for human interaction, with only a single minor fault (one instance of the carousel failing to turn before dispensing). All of the heterocycles induced the formation of new complexes, except for 2,6-lutidine. 2-picolinic acid produced complexes containing a chelating κ^2^-carboxylate ligand. 4,4′-Bipyridine, isoquinoline, and 2-aminopyridine displaced a single chlorido ligand to form complexes bound through the pyridyl nitrogen. Piperazine showed evidence of new species by MS, with the isotope pattern indicating the presence of ruthenium, but these could not be unambiguously assigned. Pairwise combinations of the *N*-heterocycles were also attempted in a brute-force screen, with MS data implying that only complexes observed in the initial library were formed.

A second experiment varied the number of equivalents of hydrochloric acid in the synthesis of polyoxomolybdates—nanoscale species^40^ with interesting self-assembling behavior^41,42^ and a variety of applications.^43^ When conducted noniteratively, this is effectively a focused library in process space, as opposed to the chemical space exemplified by the previous library (Figure 3, right).

The system chosen required premixing solutions of Na_2_MoO_4_·2H_2_O, NaOAc, 50% acetic acid in water, and 1 M HCl in one reactor flask, before combining this mixture with solid sodium dithionite as a reducing agent in a second flask. Depending on the pH of the reaction mixture, the system self-assembles to give one of two polyoxometalates, the {Mo_132_} ball or a {Mo_154_} ring. Reactions were conducted using a series of equivalents (0–13) of 1 M HCl_(aq)_. The two species have previously been observed to coexist around pH 2.6,^42^ and we also observe this behavior where 5 equivalents of acid are used. The yield of {Mo_132_} was maximized in the absence of acid, and {Mo_154_} with 7 equivalents.

### Prospecting Library Generation

Our workflow for prospecting library generation uses an algorithm to direct the exploration of a process space toward areas of the space more likely to produce more novel species. This is achieved through the creation of a metric built on the difference between given experimental spectra and both the starting material and previous experimental spectra. The larger the difference, the larger the score, and thus the more likely a serendipitous or novel discovery has been made.

Directing the exploration toward the discovery of novelty explicitly should enumerate the chemical space accessible, effectively creating a prospecting library containing these enumerated products. However, unconstrained exploration does not guarantee purity in the systems produced, and so we also propose a method of coalescing and deconvoluting the resulting MS dataset to allow chemical diversity to be screened at the systems level. This removes the requirement to optimize the purification of many systems of varying composition.

**Figure 4:**
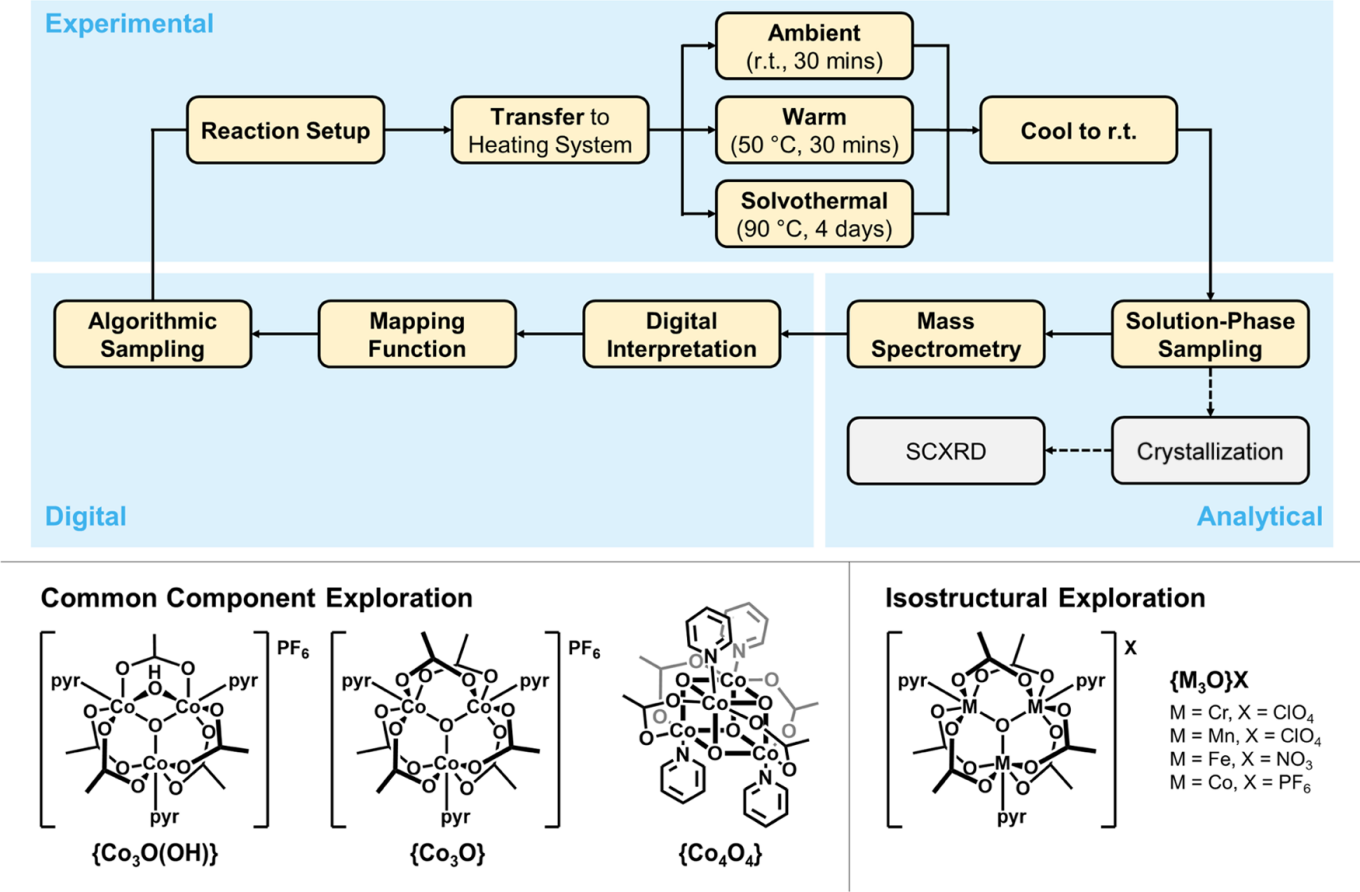
(Top) Workflow for the autonomous exploration of a cluster metal carboxylate search space. Steps are broken down into the Experimental, Analytical, and Digital sections of the workflow. Single-crystal X-ray diffraction (SCXRD) analysis was attempted where crystals of sufficient quality could be isolated but did not inform the sampling of further iterations. (Bottom) Cluster starting materials used in the two explorations to generate the prospecting libraries.

We decided to probe cluster metal carboxylates, as such species offer a highly diverse chemical space of theoretical products. The system is highly reconfigurable, with aggregation or decomposition leading to a large space formed by very few building blocks. The combinatorial nature of this chemical space makes reaction outcomes difficult to predict heuristically or by simulation.^2^ Cluster metal carboxylates have a wide range of potential applications as biological model compounds and materials.^29,30,32,44−51^ The space of possible compounds is expanded through the use of additives with structure-directing effects, which were chosen for their potential to promote higher nuclearity structure formation through templation (lanthanide chloride hydrate salts), bridging between self-contained cluster building blocks (ditopic carboxylic acids), and steric shielding of the metal-oxo core (*N*,*N*′,*N*″-trimethyl-1,4,7-triazacyclononane, TMTACN). Two chemical systems were ultimately used, one involving species formed only from Co(III), acetate, pyridine, and water (the Common Component Exploration), and the other comprising species with the general formula [M_3_O(OAc)_6_(pyr)_3_]^+^ and a variety of metals, M = Cr, Mn, Fe, Co (the Isostructural Exploration). The exploration proceeded iteratively, following a common pattern from similar experiments, which includes reaction, analysis, and digital interpretation of the analytical data to score each reaction. Finally, suggestion of a subsequent round of experiments is made by a digital agent (Figure 4).^6^

Rather than using the Chemputer in the same form as for the focused libraries, reactions were performed directly in a variation of the sample storage carousel. This increased the throughput of the exploration beyond the four reactor modules to a maximum of 24 simultaneous reactions. The separate Geneva Wheel Platform (GWP) was outfitted to permit direct sample dispensing and stirring,^24^ with a batch heater used to enable temperature control of the reaction. Reactions were conducted under three temperatures—ambient conditions for 30 min, 50 °C for 30 min, and solvothermally at 90 °C for 4 days. An iteration comprised 48 reaction compositions each conducted at the three temperatures. Electrospray ionization mass spectrometry (ESI-MS) spectra were recorded for every combination of composition and temperature by sampling the diluted reaction mixtures directly.

MS spectra were then scored according to a bespoke metric designed to quantify the novelty of experiments. The metric comprises two halves: (i) a score of the percentage difference between the five most intense peaks in the spectra of each starting material and the five most intense peaks in each experiment, and (ii) a count of any peaks in the experiment spectrum (above a noise threshold) that do not appear in cluster starting material spectra. Weighting of these peaks by the inverse of their frequency within the dataset means that commonly occurring peaks contribute less to this second measure than do rarer peaks. The scores are recalculated with each iteration, as the frequency parameter of the second measure must be updated to cover the full dataset fairly.

The two halves of the novelty score were then individually feature-scaled between 0 and 1, to weight them evenly, before averaging to give a final score for each experimental spectrum. These scores were further averaged over the three temperatures. In this way, each of the 48 compositions used in an iteration is given a single numerical score between 0 and 1. Averaging across temperatures runs the risk of obscuring some of the variation in the results due to this variable, but with this methodology validated, steps can be taken to improve on this in future explorations.

The initial iteration of experimental compositions was chosen by Latin Hypercube sampling (LHS) to ensure an even spread of data points over the search space. Subsequent experiments were chosen via Bayesian optimization with a Gaussian process surrogate model as prior (GPBO).^52^ The GP model was built from the calculated novelty scores using a combined Matérn and white noise kernel. The optimization used Lower Common Bound (LCB) as the acquisition function to permit easy tuning between exploitation and exploration of the space. Uniform random sampling was also used to generate experiments for one iteration of the Common Component Exploration, to provide a comparison for GPBO. A short primer on each method is available in the SI. The data were evaluated by other offline analytical methods where possible. Any experiments yielding crystalline species were analyzed by SCXRD, and all MS spectra were then subjected to a digital pipeline to deconvolute usable chemical insights from the spectral data (discussed in the next section).

Under ambient and warm (50 °C) conditions, the first and second iterations of the Common Component Exploration afforded a number of crystals that matched species reported to be intermediates in the synthesis of {Co_3_O} and {Co_3_O(OH)} starting materials.^53^ Two of these were distinct species—[Co_3_O(OH)_2_(OAc)_3_ (pyr)_5_](PF_6_)_2_ and [Co_2_(OH)_2_(OAc)_3_(pyr)_4_]PF_6_—and a further two methanolysis products. In assessing the significance of these discoveries, it is of note that the optimizer does not bias its data collection to align with any pre-programmed chemical heuristic. Although these complexes are known in the literature, they are completely new to this system and are scored accordingly.

**Figure 5:**
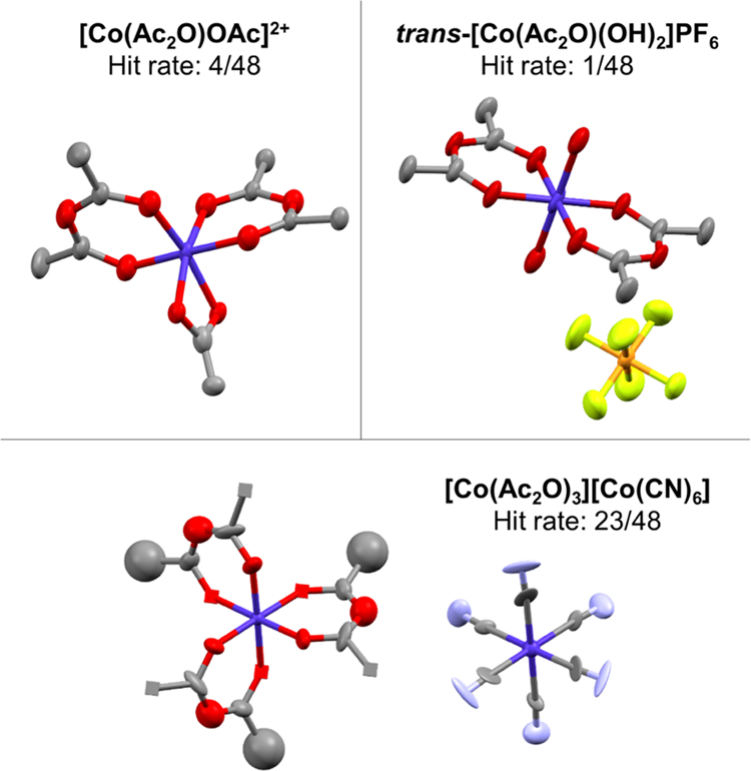
Cobalt(III) anhydride complexes isolated from solvothermal (90 °C for 4 days) samples in the first iteration of the Common Component Exploration. “Hit rate” refers to the number of experiments affording crystals of the material from the first iteration. Ellipsoid representations of Co, dark blue; C, gray; O, red; N, light blue; P, orange; F, light green.

Under solvothermal conditions, the first iteration of the Common Component Exploration afforded crystals corresponding to a family of three acetic anhydride complexes of Co(III) (Figure 5). All of these appear to be new to the literature, with only a single example of an acetic anhydride complex (of Ti(IV))^54^ found in the Cambridge Structural Database. Two of the complexes formed appear to be of structural types proposed from *in situ* analysis of anhydride complexes of other metal ions,^55−58^ and the third, [Co(AcOAc)_2_(OAc)]^2+^, has not been proposed previously with these ligands.^59,60^ Counteranions could not be unambiguously assigned for this species; however, ^1^H NMR data show agreement with the crystal structure of the cationic portion. Acetic anhydride is believed to form via condensation of the acetic acid and NMR analysis implies that this is promoted in the presence of cobalt in either the +II or +III oxidation states. Cyano ligands are hypothesized to originate from the acetonitrile solvent, with metal-mediated C–C cleavage of acetonitrile previously reported, including an example with a cationic Rh(III) complex.^61−67^

The Isostructural Exploration afforded no crystals under ambient or warm conditions except for methanolysis products. Under solvothermal conditions, however, almost all samples afforded crystals of [M(OMe)_2_(OAc)]_10_, {M_10_}. Under comparable conditions with only a single metal cluster precursor, only the Fe(III) system gave an analogous species,^68^ although {Cr_10_} rings are also reported in the literature.^69^ An IR spectrum of the crystals did not match that of the {Fe_10_} ring precisely, with notable differences in the M–O stretching region, implying a multimetallic composition.

### Deconvolution of MS Data from the Prospecting Library

**Figure 6:**
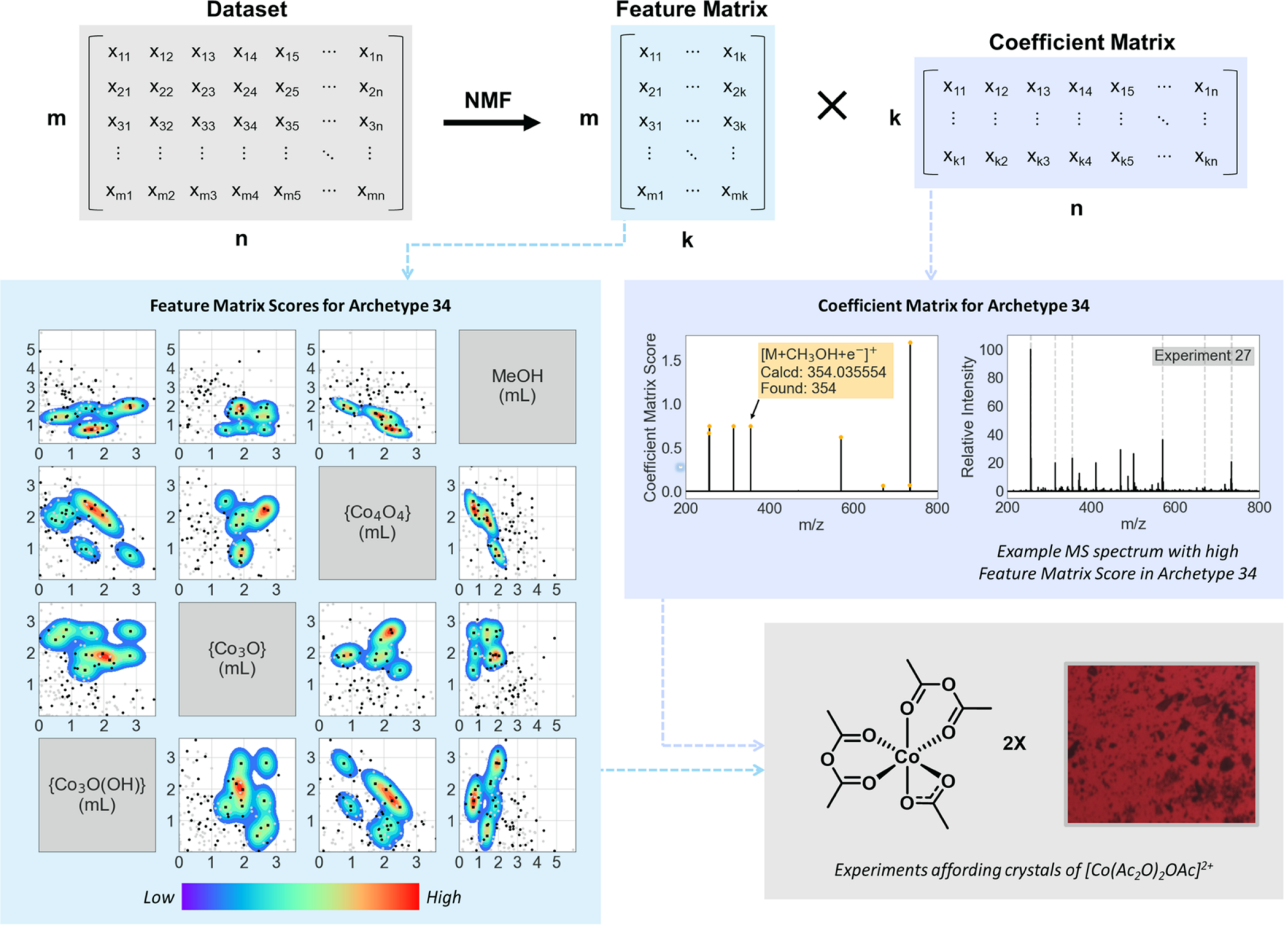
(Top) Overview of the NMF process, where *m* is the experiment number, *n* corresponds to each unique peak observed throughout the dataset, and *k* is effectively groupings of commonly occurring peaks in the *n* set—i.e., MS archetypes. (Bottom left) Truncated matrix plot of the search space. Data points indicate conditions of each experiment. A kernel density estimation, weighted by the feature matrix score for the [Co(Ac_2_O)OAc]^2+^-related archetype, indicates where this product distribution is located within the process space. (Mid right) Corresponding coefficient matrix scores and comparison with experimental MS data.

Deconvolution was achieved by taking the list of unique peaks for each experiment at a given temperature, converting these to an ordered list indicating the presence or absence of a peak at a given *m*/*z* with a binary notation, and the array of these “barcodes” subjected to non-negative matrix factorization (NMF), to afford a lower-rank approximation of the original barcode array (Figure 6). The rank is determined by screening ranks from 2 to the number of experiments in the barcode array and using as a threshold the rank at which reconstruction error becomes minimized. In this way, the coefficient matrix corresponds to groupings of *m*/*z* values that commonly occur together—effectively, fingerprints corresponding to specific product distributions. The feature matrix provides the composition of the MS spectrum for each experiment in terms of these proposed product distributions. We refer to the proposed product distributions as “Archetypes.”

NMF was chosen over similar methods (e.g., principal component analysis, PCA) due to the ability to reconstruct the approximated original dataset through only additive combination, which the original paper from Lee and Seung^70^ highlights as being “compatible with the intuitive notion of combining parts to form a whole”. This leads to more intuitively interpretable approximated matrices. While NMF has been applied to the interpretation of MS spectra before (including the deconvolution of overlapping spectra,^71^ the interpretation of large datasets,^72^ and unsupervised classification in mass spectrometry imaging^73,74^), we are not aware of any literature leveraging the beneficial properties of the technique in a discovery context.

Examination of the feature matrix scores for those experiments in the Common Component Exploration yielding crystals of [Co(AcOAc)_2_ (OAc)]^2+^ can be used to demonstrate that the archetypes have chemical significance. All four of these experiments show high scores in a particular archetype (with some feature matrix variation unique to each experiment). Examination of the corresponding entry in the coefficient matrix indicates that this archetype corresponds to peaks that may be assigned to the crystallized species, notably *m*/*z* = 354, assigned as [M + CH_3_OH + e^–^]^+^. The presence of this archetype in samples from later iterations indicates the likely presence of [Co(AcOAc)_2_(OAc)]^2+^ in these experiments, despite no SCXRD-quality crystals being collected.

The feature matrix scores for a given archetype can be used to draw conclusions about the areas of the process space (i.e., the synthetic conditions) that afford a given product distribution. This is represented for the discriminatory [Co(AcOAc)_2_(OAc)]^2+^ archetype in Figure 6 as a kernel density estimation weighted by the archetype’s feature matrix score for each experiment. The archetype with the largest percentage contribution to the summed feature matrix scores of a given experiment may be readily calculated, giving potential insights into the purity of product mixtures—information that has value for further synthesis but is otherwise difficult to obtain from MS data.

The NMF approximation may also give insight into the efficacy of our exploration approach. Comparing the increase in archetypes discovered between the initial Common Component Exploration Run 1 and the GPBO-directed Run 2 at all temperatures, against the increase between Run 1 and the randomly-sampled Run 3, we observe that GPBO discovers more archetypes (and thus more product distributions) than random sampling. The percentage of archetypes discovered in Run 2 vs Run 3 that are shared discoveries varies between 50 and 80% depending on temperature, indicating that, to some extent, different areas of the search space are targeted.

If NMF approximations are created not from datasets of all experiments in an exploration at a given temperature, but from experiments from each iteration at each temperature, we observe that GPBO-directed Runs 2 and 4 tend to require a higher rank to minimize reconstruction error. This implies a greater chemical diversity discovered during these GPBO runs. In the Isostructural Exploration, this trend is not readily apparent, which we suggest is due to an overall lower chemical diversity present in the search space. Thus, GPBO appears to be a better method of exploration than random sampling for the discovery of novelty in process space, in terms of the number and diversity of new product distributions discovered.

## Conclusions

In this work, we have demonstrated the production of focused and prospecting libraries^8^ in coordination chemistry using programmable chemical reaction platforms like the Chemputer.^16^ Such platforms can generalize our methods beyond the examples shown due to the use of a programming approach based on the abstraction of chemical syntheses.

Generalizability is demonstrated through the initial TOS of three coordination complexes and the metastable polymorphs of calcium carbonate. Key facets of inorganic synthesis are represented, including purification by recrystallization, self-assembly of multinuclear species, stereoselective synthesis, and preparation of thermodynamically unstable phases. Parallelization was then used to generate two focused libraries. The first allowed the synthetic enumeration of a number of Ru(bipy)_2_Cl_2_ derivatives, and the second demonstrated the relationship between synthesis conditions and yield of two polyoxomolybdates. In this way, we show that focused libraries can be developed in both chemical and process space by our system.

Further to this, we provide a workflow for the forward synthesis of a prospecting library of coordination entities that links the product distributions discovered to the areas of process space they occur within. This allows us to screen for the diversity present in the search space while overcoming the common criticism of DOS as not leading to the development of synthetic chemistry.

Our algorithmic explorer has no knowledge of chemistry and is driven toward the areas of the search space showing the rarest results by a novelty quantification metric calculated from MS data. We demonstrate that this method is superior to random sampling—building on previous reports of accelerated serendipity through random choice of reactions^5^ to “engineered” serendipity through algorithmic direction. We propose that the resultant dataset of non-starting material peaks can be used to isolate product distributions and demonstrate the validity of this for a species characterized orthogonally by SCXRD. We are able to discover and direct our exploration from the systems level, without losing time, effort, and resource to the purification of many uninteresting results.

Our workflow for screening diversity is generalizable to any system, that can be analyzed reliably by MS, rather than optimized toward a particular application as for DOS. While we chose to direct our exploration by quantifying the novelty of the result, the mapping function could be changed to assay for a particular function or behavior. Central to the diversity discovered is the choice of process space, and further work will aim to highlight considerations and heuristics to allow chemists to best formulate these exploration problems. Future work should also focus on increasing the complexity of the synthetic processes that can be screened (effectively expanding the dimensionality of the process space) to increase the complexity of the product species accessible.

## Data Availability

Code and related data files are available from GitHub at: github.com/croningp/DigitalCoordinationChemistry.

## Supporting Information Available

Further discussion of platform construction, data science workflows, and experimental data (PDF)

## Abbreviations

**DOS**: diversity-oriented synthesis
**ESI**: electrospray ionization
**GWP**: Geneva Wheel Platform
**GPBO**: Gaussian process Bayesian optimization
**LCB**: lower common bound
**MLCT**: metal ligand charge transfer
**LHS**: Latin hypercube sampling
**MS**: mass spectrometry
**NMF**: non-negative matrix factorization
**NMR**: nuclear magnetic resonance
**SAR**: structure–activity relationship
**SCXRD**: single-crystal X-ray diffraction
**TMTACN**: 1,4,7-trimethyl-1,4,7-triazacyclononane
**TOS**: target-oriented synthesis
**UV–vis**: ultraviolet–visible (spectroscopy)
