# Supplementary material for: Automated Library Generation and Serendipity Quantification Enables Diverse Discovery in Coordination Chemistry
Source: J Am Chem Soc. 2023 Jan 17;145(4):2332–41. doi: 10.1021/jacs.2c11066 (PMC9896557; doi:10.1021/jacs.2c11066)
Supplement: Supplementary file 1 — ja2c11066_si_001.pdf [file ja2c11066_si_001.pdf]

Automated Library Generation and Serendipity Quantification enables Diverse Discovery in Coordination Chemistry

Daniel J. Kowalski, Catriona M. MacGregor, De-Liang Long, Nicola L. Bell, Leroy Cronin*

School of Chemistry, University of Glasgow, University Avenue, Glasgow, G12 8QQ, UK

**Correspondence email: lee.cronin@glasgow.ac.uk*

Supplementary Information

Contents

1 ChemPU Platform 6
1.1 Hardware 6
1.1.1 Methods used during Platform Construction 6
1.1.2 Wiring and Ethernet Switch 6
1.1.3 Chemputer Frame 7
1.1.4 Tubing 7
1.1.5 Pumps 8
1.1.6 Valves 9
1.1.7 Reactor Modules 10
1.1.8 Jacketed Filter Module and Chiller 11
1.1.9 Sample Wheel 12
1.1.10 Reagent bottles 13
1.1.11 Waste Containers 13
1.1.12 Vacuum 13
1.1.13 PoE Converter 13
1.2 Software 14
1.2.1 Abstraction of Chemical Synthesis 14
1.2.2 Chempiler Software Suite 14
1.2.3 CommanduinoLabware 15
1.2.4 Graph File (.json) 15
1.2.5 Digitised Procedure (.py) 15
1.3 Configuration 18
1.4 Error Testing 20
1.4.1 Accuracy of the Pump/Valve Combinations 20
1.4.2 Error in Dispensing of a Given Volume through the Backbone 21
2 Modular Wheel Platform 24
2.1 Hardware 24
2.1.1 Geneva Wheel Mechanism 24
2.1.2 Fan Stirrer 25
2.1.3 Dispensing 26
2.1.4 Vial Heater 26
2.1.5 Control and Power 26
2.2 Software 27
2.3 Error Testing 28

3 Target-Oriented Syntheses of Coordination Compounds ... 30
3.1 Chemicals and Analytical Measurements ... 30
3.2 General Operation and Cleaning Procedures ... 31
3.2.1 General Notes ... 31
3.2.2 Jacketed Filter Module Cleaning Procedure ... 31
3.2.3 Reactor Module Cleaning Procedure ... 31
3.2.4 Input Cleaning ... 32
3.2.5 Washing Solid Residues ... 32
3.3 Preparation of Metastable Calcium Carbonate Polymorphs ... 33
3.3.1 Vaterite ... 33
3.3.2 Aragonite ... 34
3.3.3 Comparative IR Data ... 34
3.4 Target 1: Tris(2,2'-bipyridine)ruthenium tetrafluoroborate ... 35
3.4.1 Bis(2,2'-bipyridine)dichlororuthenium(II), $Ru(bpy)_2Cl_2.xH_2O$ ... 35
3.4.2 Tris-(2,2'-bipyridyl)ruthenium tetrafluoroborate hexahydrate, $[Ru(bpy)_3](BF_4)_2.6H_2O$36
3.5 Target 2: Trinuclear oxo-centred Mn(III) Acetate, $\{Mn_3O\}$ ... 38
3.5.1 Tetra-*n*-butylammonium permanganate, $TBAMnO_4$ ... 38
3.5.2 $[Mn_3(\mu_3\text{-}O)(OAc)_6(pyr)_3]ClO_4$ ... 39
3.6 Target 3: Cisplatin ... 40
3.6.1 Stability of KI(aq) Stock Solution ... 40
3.6.2 *cis*-diamminediiodoplatinum(II), $[Pt(NH_3)_2I_2]$ ... 41
3.6.3 Cisplatin, $[Pt(NH_3)_2Cl_2]$ ... 42
4 Focused Library in Process Space: $\{Mo_{132}\}$ and $\{Mo_{154}\}$ ... 43
4.1 General Procedure ... 43
4.2 UV-vis Spectroscopy ... 44
5 Focused Library in Chemical Space: $Ru(bpy)_2Cl_2$ ... 45
5.1 General Procedure ... 45
5.2 Results from Reactions with a Single N-Heterocycle ... 46
5.2.1 Overview of Characterisation Data ... 46
5.2.2 Starting Material, $[Ru(bipy)_2Cl_2]$ ... 47
5.2.3 2,6-Lutidine ... 48
5.2.4 2-Picolinic Acid ... 49
5.2.5 Piperazine ... 50
5.2.6 2-Aminopyridine ... 51
5.2.7 Isoquinoline ... 52
5.2.8 4,4'-Bipyridine ... 53

5.3 Results from Pairwise Reactions 54
5.3.1 MS Data 54
5.3.2 UV Data 55
5.3.3 Summary 56
6 Automated Exploration 57
6.1 Preparation of Starting Materials 57
6.1.1 $[Cr_3(\mu_3\text{-}O)(OAc)_6(H_2O)_3](OAc).xH_2O$ 57
6.1.2 $[Cr_3(\mu_3\text{-}O)(OAc)_6(pyr)_3]ClO_4$, $\{Cr_3O\}$ 58
6.1.3 $[Mn_3(\mu_3\text{-}O)(OAc)_6(pyr)_3]ClO_4$, $\{Mn_3O\}$ 59
6.1.4 $[Fe_3(\mu_3\text{-}O)(OAc)_6(H_2O)_3]NO_3.xH_2O$ 60
6.1.5 $[Fe_3(\mu_3\text{-}O)(OAc)_6(pyr)_3]NO_3$, $\{Fe_3O\}$ 61
6.1.6 $[Co_3(\mu_3\text{-}O)(\mu_2\text{-}OH)(OAc)_5(pyr)_3]PF_6$, $\{Co_3O(OH)\}$ 62
6.1.7 $[Co_3(\mu_3\text{-}O)(OAc)_6(pyr)_3]PF_6$, $\{Co_3O\}$ 63
6.1.8 $Co_4(\mu_3\text{-}O)_4(OAc)_4(pyr)_4$, $\{Co_4O_4\}$ 64
6.2 Autonomous Exploration General Procedure 65
6.3 Additional Characterisation Data 66
6.3.1 Heteroleptic Cobalt(III) Anhydride Complex, $[Co(Ac_2O)_2OAc]X_2$ 66
6.3.2 $\{Fe_{10}\}$ Ring, $[Fe(OMe)_2OAc]_{10}$ 67
6.3.3 $\{M_{10}\}$ Ring, $[M(OMe)_2OAc]_{10}$ 67
6.4 Additional Experimental Notes 69
6.4.1 Monitoring Degradation in Starting Material Stock Solutions 69
6.4.2 Origins of Acetic Anhydride under Solvothermal Condtions 71
6.5 Non-Statistical Sampling Methods 74
6.5.1 Uniform Random Sampling 74
6.5.2 Latin Hypercube Sampling 75
6.6 Gaussian Process Bayesian Optimisation 77
6.6.1 Surrogate Model 77
6.6.1 Acquisition Function 79
6.6.2 Constant Liar Strategy 79
6.7 Mapping Function 80
6.7.1 Overview Flowchart 80
6.7.2 Validation of Samples 81
6.7.3 Examples: Sample Novelty 82
6.7.4 Examples: Average % Difference to Standards Component 83
6.7.5 Test Dataset 84
6.8 Reproducibility of MS Data from Explorations 87

6.9 Reaction Conditions and Results from the Common Component Exploration 88
6.9.1 Overview 88
6.9.2 Reaction Conditions .... **Error! Bookmark not defined.**
6.9.3 Graphical Representations of Reaction Conditions Sampled 89
6.9.4 Mapping Function Data.. **Error! Bookmark not defined.**
6.9.5 List of Unique Peaks 92
6.10 Reaction Conditions and Results from the Isostructural Exploration................................ 103
6.10.1 Overview 103
6.10.2 Reaction Conditions .... **Error! Bookmark not defined.**
6.10.3 Graphical Representation of Reaction Conditions Sampled...................................... 104
6.10.4 Mapping Function Data.. **Error! Bookmark not defined.**
6.10.5 List of Unique Peaks 107
6.11 Dataset Analysis of the Unique Peak Data for the Common Component Exploration....... 113
6.11.1 Dimensionality Reduction by Iteration 113
6.11.2 Dimensionality Reduction by Temperature 115
6.12 Dataset Analysis of the Unique Peak Data for the Isostructural Exploration 130
6.12.1 Dimensionality Reduction by Iteration 130
6.12.2 Dimensionality Reduction by Temperature 131
6.13 Chemical Validation of NMF Archetypes 133
6.13.1 Correlation with Archetypes by Temperature for entire Solvothermal Dataset 133
6.14 Crystallographic Data 139
6.14.1 Data for novel species.. 139
6.14.2 Bond Valence Calculations 141
6.14.3 Data for previously reported species.. 141
7 References 142

1 ChemPU Platform

1.1 Hardware

Where this work employs Chemputer modules previously reported only a description of the module is provided. For more in-depth build instructions for the individual modules, a full inventory, and an extended system specification please refer to the supplementary information for Angelone *et al.*[1]

1.1.1 Methods used during Platform Construction

3D-Printing. Performed on a Connex 500 printer from Stratasys using the Fullcure 720 translucent resin for the major body of the printed parts and VeroBlack as the coloured resin in cases where two-coloured prints were required. Once the print was complete, the parts were cleaned first by manually scrapping away the bulk of the support material, followed by thoroughly washing them in a water jet cleaning station (Quill Vogue Polyjet). Finally, the parts were placed in a 0.1 M NaOH(aq) bath for 30 min, and cleaned again with the water jet. Particular attention was paid to small holes for screws/magnets/etc. to ensure no residual support material was present.

Laser Cutting. Performed on a Monster1060 CO2 Laser system (ML1060 130 W) from Radecal with the RDWorksV8 software. The applied parameters are summarised in Table S1.

Table S1: *Settings for the laser cutter*

**Material**	6 mm Acrylic
**Laser power**	70 – 85%
**Speed**	10 mm/s
**Air**	On
**Laser through mode**	Enabled
**Air pressure**	0.3 MPa
**Flow rate**	33 L/min

Soldering. Performed with a Tenma Digital Soldering Station (60 W) set to 270 °C. The solder wire used was Loctite (60EN alloy, 0.7 mm diameter, 2C core, X39 flux) with extra Amtech NC-559-TF flux.

1.1.2 Wiring and Ethernet Switch

The majority of the devices required to operate the rig are run through a NETGEAR ProSafe GS752TPP 48-Port Gigabit PoE+ Smart Switch. The switch is then connected to a PC tower via Ethernet.

Ethernet cables were used to connect the switch to the Pumps, Valves, and PoE convertors. PoE convertors were connected via Serial cables to the stirrer-hotplates and circulator. The overhead stirrer is connected directly to the PC tower via USB.

Wires were suspended from the base of the shelving using RS Components Cable Clip Black PVC Retaining Clips and Cable Clip Black Screw Nylon Cradle Clips. CabGrip cable managers, attached directly to the V-Slot Linear Rails of the frame, were also used to ensure no cables were permitted to trail.

1.1.3 Chemputer Frame

The Chemputer is built up from a frame built from OpenBuilds® parts obtained from Ooznest. V-Slot Linear Aluminium Rails are used to form the body of the frame and joined by L-brackets and T-plates secured with Drop-In Tee Nuts (M5) and accompanying low profile bolts.

Size was determined by the dimensions of the fumehood. The same connectivity can be trivially rescaled to fit whatever space is required.

Shelving for the pumps and valves is mounted to this frame. The shelves each comprise two layers of 12 mm polypropylene custom cut by JetCut. The lower layer provides a solid base, and an upper layer acts as a mount, with holes cut to correspond to the footprints of 12 pumps (for the upper shelf) or valves (for the lower shelf). An additional piece of custom 6 mm polypropylene, also obtained from JetCut, is used as a guard on the front of the upper shelf.

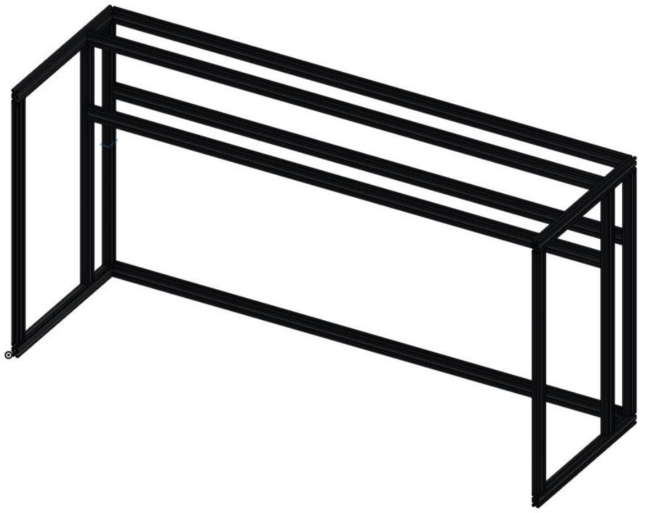

Figure S1*: 3D-rendering of the Chemputer Frame. Reproduced from Angelone* et al.[1]

1.1.4 Tubing

Tubing used was 1/8” (3.2 mm) PTFE from Cole-Parmer®. Connections to the pumps and valves made use of Thames Restek Flangeless Fitting Natural, Polypropylene, 1/4 -28 Flat-Bottom for 1/8” OD.

1.1.5 Pumps

The motion of the syringe is controlled by a Nema17 Linear Stepper Motor with Tr8*4(P2) 250 mm length leadscrew. A lead screw nut is attached to an in-house 3D printed carriage, which is in turn connected to the barrel of the syringe. This in-house carriage is further secured to a complementary guide rail and carriage system from Moore International to ensure vertical motion only.

Another in-house print acts as a mount for the body of the syringe and connection to the motor to permit dispensing by rotation of the lead screw. An in-house printed plate secures the syringe. The syringes attached are sourced from ILS with a volume of 10 mL.

To permit determination of the home position, a hall sensor board is attached to the printed mount and a MagnetExpert® 4mm dia x 3mm thick N42 Neodymium Magnet to the printed carriage. The Hall sensor board comprises a Hall sensor unit (from Texas Instruments) and bespoke circuit board.

Both the hall sensor and the motor are connected to a custom PCB driver/controller setup which permits control of the pump via Ethernet.

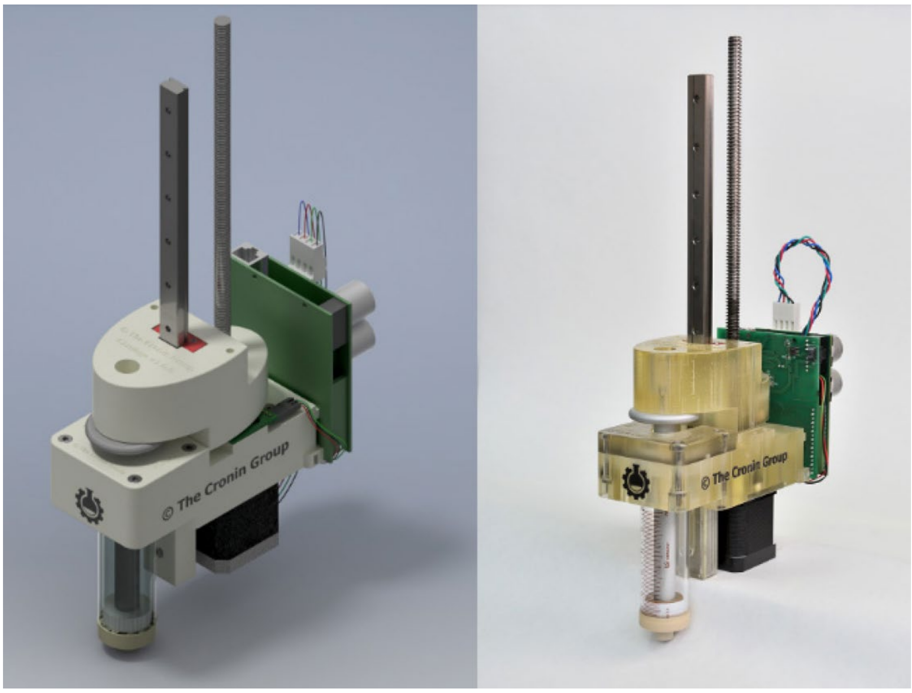

***Figure S2**: (Left) Autodesk Inventor rendering of the pump assembly. (Right) Photograph of the valve assembly. Reproduced from Steiner* et al.[2] *Both are shown with a 10 mL syringe.*

1.1.6 Valves

The valve comprises a NEMA23 motor with D-cut shaft from Longs Motors, on to which is mounted an in-house 3D printed drum. The drum is used to connect the motion of the motor to a Thames Restek 6-port selection valve 0.063 red into which the tubing is connected.

The drum and valve are held in place by an in-house 3D printed housing, which also contains mounts for a Hall sensor board (as per the pump) and custom PCB driver/controller boards (into which the Hall sensor board and motor are wired). Six First4Magnets 2mm dia x 4mm thick N35 Neodymium Magnet are mounted at each position of the drum corresponding to a different valve port to allow selection of a particular port in concert with the Hall sensor board. The magnet at position 3 has its orientation inverted to provide a home position.

As for the pump, the PCB driver/controller boards permit control over Ethernet.

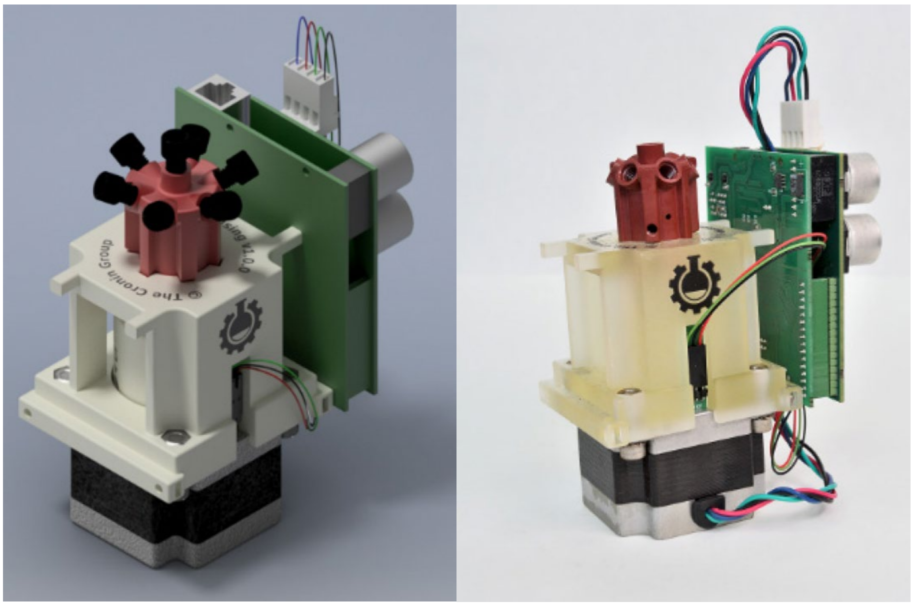

Figure S3*: (Left) Autodesk Inventor rendering of the 6-way pump assembly. (Right) Photograph of the valve assembly. Reproduced from Steiner* et al.[2]

1.1.7 Reactor Modules

Reactor modules were largely the same as any typical laboratory glassware setup. A VWR three-necked 100 mL round-bottomed flask (B24/29 centre, B14/23 side), containing a magnetic stirrer bar and fitted with an Asynt air condenser, was clamped in a DrySyn heating block above an IKA RCT Digital stirrer-hotplate fitted with temperature probe. The hotplate was connected to a PoE converter via serial lead and then wired into the Ethernet hub to permit digital control.

One side arm was fitted with a glass GL 18 to B14 connector from VWR, which suspended tubing as deep inside the flask as possible, and the other with a glass stopper. As for the reagent bottles, the connector required a VWR GL 18 screw cap for tube connection, and appropriate insert with 3.2 mm inner diameter.

The four reactors were secured in place using typical laboratory clamp stands, support rods, flask clamps and bossheads.

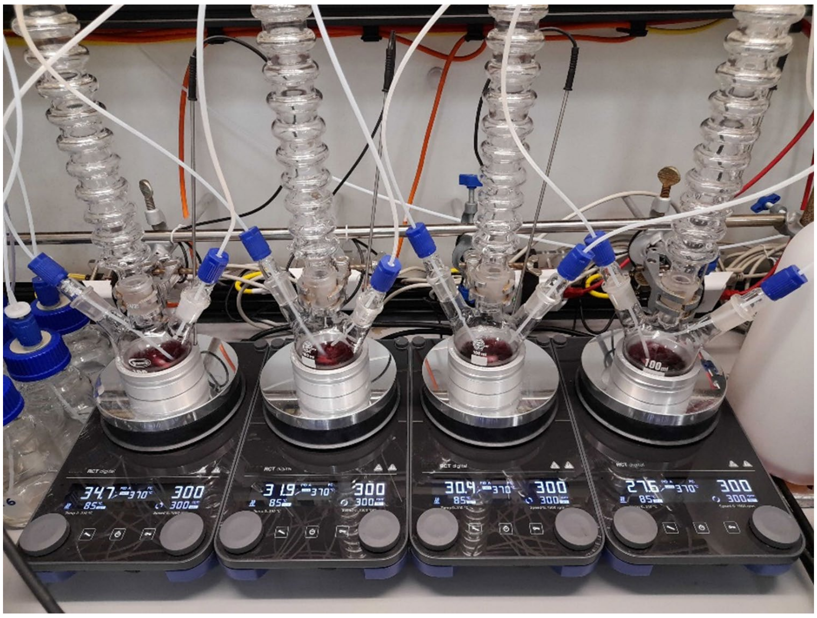

***Figure S4**: Photograph of the parallelised reactor modules in use.*

1.1.8 Jacketed Filter Module and Chiller

The jacketed filter itself was custom glassware provided by the University of Glasgow Glassblowing Service.[3] This consisted of a central chamber with three necks (B29/32 centre, B14/23 side), and surrounded by a cavity, through which heated or cooled fluid could be circulated to maintain temperature. The base of the central chamber was marked by a sintered glass frit, below which is an outlet capable of connection to the tubing used in the remainder of the rig.

Temperature control was achieved using a Cole-Parmer JULABO CF41 Cryo-Compact Circulator, using JULABO Thermal H10 bath fluid to permit a working temperature range of -40 to +180 °C.

The central neck of the flask was kept open, and an IKA® Microstar 7.5 control overhead stirrer (30-2000 rpm) connected to VWR Stirring Shaft (300 x 8 mm) and Stirrer Blade threaded through to permit agitation of the flask contents. One side arm acted as an inlet for the tubing via a VWR GL18 to B14 connector, GL18 screw cap for tube connection and Insert for screw cap GL18, 3.2 mm inner diameter. The remaining side arm was fitted with either an air condenser or a glass stopper as appropriate.

The connection at the base of the jacketed filter is threaded, and connected to tubing via a Cole-Parmer Dibafit™ Threaded Coupling, ¼"-28 UNF(F) flat bottom, PEEK.

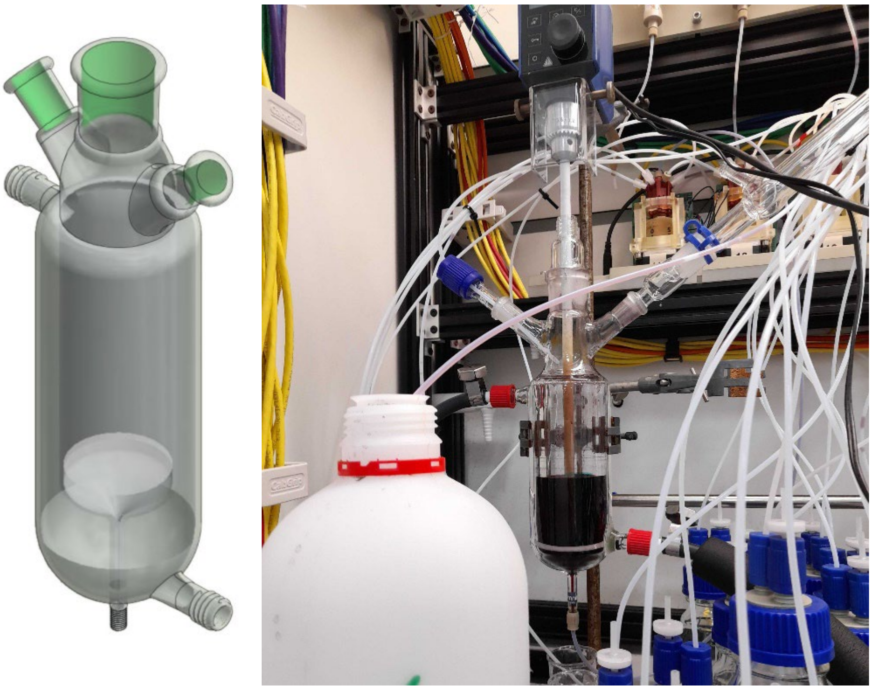

***Figure S5**: (Left) 3D-rendering of the jacketed filter. Reproduced from Steiner* et al.[2] *(Right) Photograph of the jacketed filter module in use.*

1.1.9 Sample Wheel

OpenBuilds® parts obtained from Ooznest. V-Slot Linear Rails are used to form the body of the frame and joined by L-brackets and T-plates secured with Drop-In Tee Nuts (M5) and accompanying low profile bolts

The sample wheel consists of a frame composed of the same OpenBuilds® parts obtained from Ooznest as the Chemputer Frame. A wheel cut in-house from 6mm acrylic is mounted from a central 3D printed axis and further 3D printed supports. Controlled, stepwise motion is achieved via a 3D printed Geneva Wheel mechanism attached to a Nema 17 Bipolar 45Ncm 2A 42x42x40 mm motor from StepperOnline. The motion of the wheel is controlled by an Arduino board, and the device is powered by an SF Series™ SF450 – 450 Watt 80 PLUS® Gold Certified High Performance SFX Power Supply Unit from Corsair.

Holes in the wheel were sized to permit suspension of VWR® 50 mL Centrifuge Tubes (clear, PP, conical-bottom), with a maximum capacity of 20 tubes.

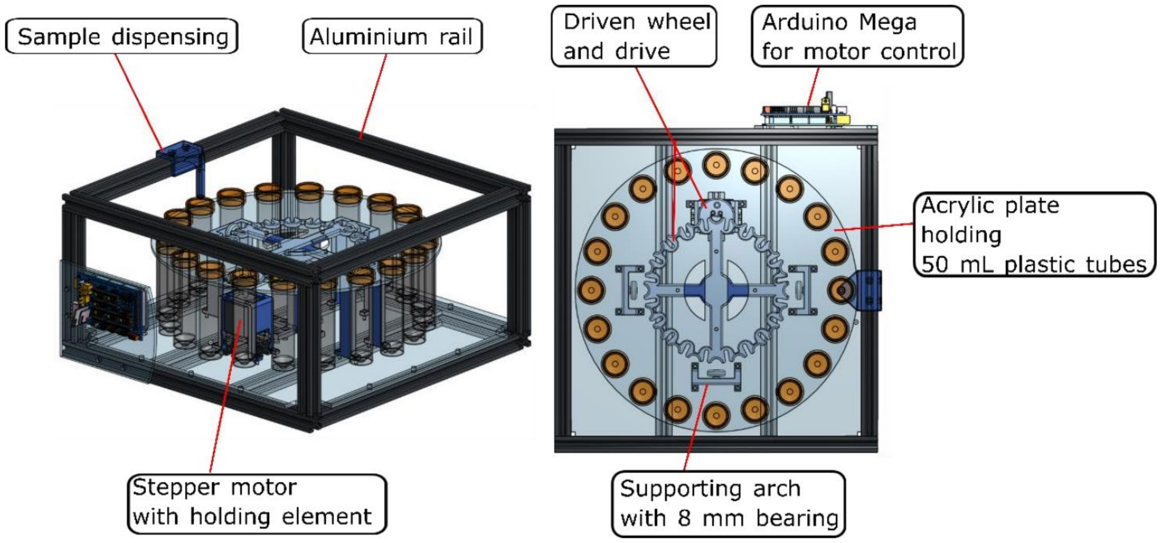

Figure S6: *Schematic of the sample wheel, shown as isometric and birds-eye view projections. Reproduced from Asche* et al.[4]

1.1.10 Reagent bottles

Reagent were stored in VWR Laboratory bottles, round, with screw cap, DURAN in 100 mL, 250 mL, and 500 mL sizes. VWR screw caps, GL 45, polypropylene, with two ports GL 14, fitted with appropriate GL 14 inserts, 3.2 mm inner diameter and GL 14 screw caps for tube connections were used to connect to the backbone. One port secured tubing (submerged to the full depth of the bottle) and the other held a Cole-Parmer Check Valve, 1/8" hose barb, diaphragm to ensure the bottles did not become pressurised.

1.1.11 Waste Containers

For smaller volumes, waste containers may be set up as for reagent bottles. However, to make waste disposal less tiresome, old 2.5L laboratory solvent bottles were reused after cleaning. These were compatible with the same screw caps as the reagent bottles, or could simply have the tubing suspended into the interior of the bottle.

1.1.12 Vacuum

Vacuum pump used was a vacuubrand® MZ 2C NT diaphragm pump. This was turned on and off manually, and connected to the jacketed filter via a valve to allow exposure to be controlled digitally.

1.1.13 PoE Converter

The PoE Converters consist of a mounting panel custom cut from transparent, colourless, 6 mm acrylic, on to which are mounted an Insight D-Link DPE-301GS PoE Splitter set to 5 V output, and RS Components D-sub, 9-Pin (Serial) to RJ45 Network Adaptor by use of PCB spacers. The PoE splitter and Network Adaptor are connected via an RS Components STP Cat6a Cable (200 mm, Yellow, RJ45) and power cable (included with the splitter). For further settings see Angelone *et al.*[1]

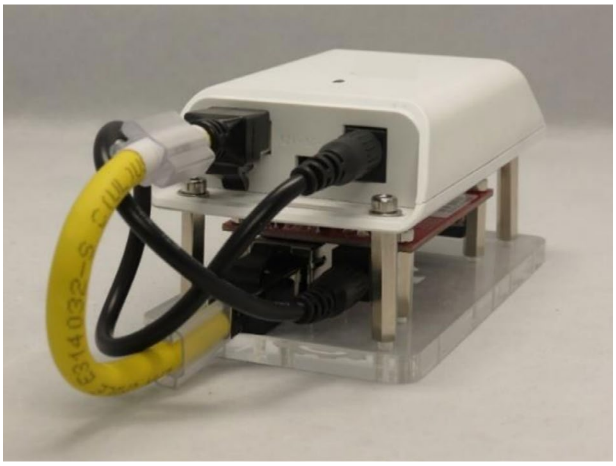

***Figure S7**: Photograph of the PoE Converter, highlighting the connectivity between the PoE splitter and 9-Pin (Serial) to RJ45 Network Adaptor. Adapted from Angelone* et al.[1]

1.2 Software

1.2.1 Abstraction of Chemical Synthesis

The hardware detailed above is controlled by software, the Chempiler, developed in-house, which has been reported in detail elsewhere.[2, 5] However, a brief overview is given below, highlighting these pre-existing tools and their usage when writing code for this project.

Chemical synthesis can be defined by a relatively small number of abstract operations (e.g. filter, stir, heat, separate, evaporate), which are used to build up the four-step structure with which chemists are innately familiar: (1) Reaction, (2) Workup, (3) Isolation, and (4) Purification.

These abstract steps may be performed with multiple different sets of hardware to achieve the same result – and offer significant flexibility to the laboratory chemist.

The Chempiler permits the divorce of an encoded procedure from a stringent, deterministic route through a largely unchanging platform, in favour of mapping the abstract operations of synthesis onto a variety of modules, based on traditional glassware setups.

This abstraction from the constraints of any one module to a series of fundamental operations offers a universal way of describing and mapping chemical robots.

1.2.2 Chempiler Software Suite

The Chempiler Software Suite comprised three python libraries, developed in-house and covering all the functionality necessary to execute a chemical procedure.

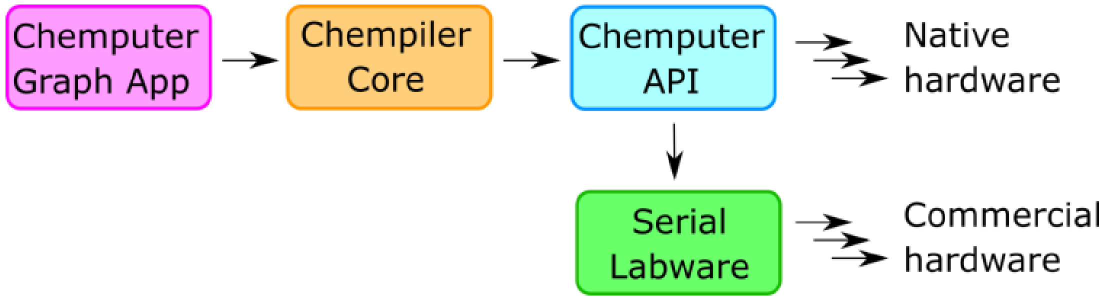

***Figure S8**: Simplified schematic showing the relationships between the various elements of the Chempiler software suite.*

1. ***Chempiler Core*** – Transforms abstract step instructions into hardware-bound actions. Requires the code to have knowledge of the topology of the rig, which is provided as a directed multigraph defined in a local .json file.

2. ***ChemputerAPI*** – Used to interface with native hardware. This includes the pumps and valves for automated fluid handling, and any sensing.

3. ***SerialLabware*** – Used to interface with commercial hardware. Examples of commercial hardware that have been incorporated into the Chemputer via SerialLabware include: hotplates, overhead stirrers, and chillers.

In addition to these python libraries, the web-based ChemIDE application[6] provides a GUI to enable creation of the .json file defining the topology of the platform as a graph. Nodes represent physical

devices with associated properties, and edges represent the physical connections between devices to permit fluid routing around the rig. An example of a Chemputer graph is shown in Fig. S13.

1.2.3 CommanduinoLabware

Outside this core architecture, an additional in-house python library is required to control the sample wheel. CommanduinoLabware is used to interface with the stepper motor via an Arduino controller to control the motion of the wheel.

CommanduinoLabware devices must be defined as custom nodes in the .json graph file.

1.2.4 Graph File (.json)

Generated by the ChemIDE web application.[6] The file contains a description of each node, detailing all associated parameters, and of each edge.

```
"nodes": [
  {
    "id": "valve1",
    "type": "valve",
    "x": 40,
    "y": 240,
    "internalId": 1,
    "label": "valve1",
    "class": "ChemputerValve",
    "name": "valve1",
    "address": "192.168.1.11",
    "current_volume": 0
  },
```

```
"links": [
  {
    "id": 34,
    "sourceInternal": 10,
    "targetInternal": 11,
    "source": "valve2",
    "target": "valve3",
    "port": "(1,2)"
  },
```

***Figure S9**: Code snippets showing the format of a graph node (left), and an edge between tow nodes (right). Node shown is a value, other types of node include pump, reactor, filter etc. to represent different physical devices.*

1.2.5 Digitised Procedure (.py)

Chemical procedures were presented to the Chemputer as a .py file containing (i) a list of libraries to import, (ii) definitions for the graph file, any custom graph nodes, and necessary reaction constants, (iii) creation of an instance of the Chempiler object, and (iv) a series of Chempiler functions that act as the experimental procedure.

For repetitive operations, user-defined functions were also included to reduce the complexity of the code.

Where necessary, breakpoints were included, prompting the user to confirm before the procedure would be continued.

```python
############################
#    Import Libraries     #
############################

# in-house Chemputer libraries and related
from chempiler import Chempiler
import xdl
import ChemputerAPI
import commanduinolabware
import logging

# for file handling
import appdirs
import os


############################
#    Chempiler Object     #
############################

# set working directory to same as this script
os.chdir(os.path.dirname(os.path.abspath(__file__)))
path=os.getcwd()

# define graph and xdl files
GRAPH_FILE = path + "\\ACC_Graph_v5_MoBlueExplore.json"

simulation_state = True

# create Chempiler object
c = Chempiler(
    experiment_code = "ACC_Mo2_Blue",
    output_dir=path,
    simulation=simulation_state,
    graph_file=GRAPH_FILE,
    device_modules=[ChemputerAPI, commanduinolabware]
)

# import sample wheel
cfg = {
    "ios": [
        {
            "type": "serial",
            "port": "COM6"
        },
    ],
    "devices": {
        "wheel": {
            "command_id": "X",
            "config": {
                "reverted_switch": True,
                "reverted_direction": False,
                "enabled_acceleration": False,
                "speed": 5000,
                "homing_speed": 12000,
                "acceleration": 2000
            }
        }
    }
}

if simulation_state == True:
    # code for simulated modular wheel device
    device=c.graph.graph.nodes['modular_wheel']['obj']
else:
    # code for real modular wheel device
    c.graph.graph.nodes['modular_wheel']['obj'] = commanduinolabware.CommanduinoLabware(config=cfg)
```

***Figure S10**: Example of the start of a ChemPU procedure, showing the instantiation of the sample wheel separately from the rest of the modules (which are initialised via creation of the Chempiler object).*

```
# start recording
c.start_recording(0)
c.camera.change_recording_speed(20)

# prime backbone and inputs
prime_inputs(["input14","input12","input20","input19","input18"], 3)   # don't need to do with input13, as backbone wash achieves this
wash_backbone("input13", 3)   # w/ EtOH

# dissolve complex
prime_filter("input13")
c.move("input13", "filter", amount_EtOH-2)   # EtOH (note that 2 mL is held back to wash bipyridyl into flask later on)
c['julabo_chiller'].set_temperature(reaction_temp)   # °C
c.stirrer.set_stir_rate("filter", reactor_stirring_rate)
c.stirrer.stir("filter")
c['julabo_chiller'].start()
c.camera.change_recording_speed(100)
c.wait(900)   # secs, 15 mins   gives some time for dissolution before the addition of other reagents
#c.stirrer.wait_for_temp("filter")

# reaction
c.camera.change_recording_speed(20)
c.move("input14", "filter", amount_bipyridyl)   # 2,2'-bipyridyl soln in EtOH
c.move("input13", "filter", 2)   # wash in to filter with held over EtOH
wash_backbone("input13", 3)   # w/EtOH
c.camera.change_recording_speed(100)
for i in range(4):
    c.wait(17280)   # secs, 4.8 hrs - repeated 4 times for a total of 19.2 hrs
    c.move("input13", "filter", amount_EtOH)
c.wait(17280)   # secs, 4.8 hrs - added to the above gives total rxn time of 24 hrs
c['julabo_chiller'].stop()
c.stirrer.stop_stir("filter")

# precipitation and filtration
c.camera.change_recording_speed(20)
prime_inputs(["input12"], 3)
c.stirrer.set_stir_rate("filter", 30)
c.stirrer.stir("filter")   # stir gently with overhead stirrer to mix properly
c.move("input12", "filter", amount_aq_sat_NaBF4, dest_port="top")   # sat NaBF4(aq)
c.move("input5", "filter", 3, dest_port="top")  # use air to blow in the remaining sat NaBF4(aq)
wash_backbone("input13", 3)   # w/EtOH
c.wait(600)   # secs, 10 mins to permit precipitation
c.stirrer.stop_stir("filter")

# sanity check
print("Begin Recryst?")
c.breakpoint()
```

Figure S11: *Section from the synthesis of [Ru(bpy)$_3$](BF$_4$)$_2$ from Ru(bpy)$_2$Cl$_2$.*

1.3 Configuration

The Chemputer design is modular, with these modules connected to one another via a "backbone" of linked 6-way valves, each paired with a mechanical pump.

The precise configuration of the platform used in these experiments is built from a backbone of 7 pumps, each connected to a 6-way valve, and with one extra valve connected to pump1 via valve1 to permit the jacketed filter to be exposed to vacuum. The exact specifications for each module have been outlined above under 'Materials and Methods'.

The backbone links four separate reactor modules, a jacketed filter module, sample wheel, three waste flasks, and 18 reagent flasks. Although only one reactor is necessary, in addition to the jacketed filter, for most of the syntheses demonstrated, multiple reactors were required for the parallel processes required in the library generation experiments. Five reagent flasks contained common solvents or solutions used in cleaning (acetone, water, acetonitrile, 0.05M Na_4EDTA in water, 0.1M HCl or HNO_3 in water), and the remainder were altered depending on the reaction in question. The large number of chemical inputs available meant that in many cases the reagents for multiple processes were able to be connected to the system simultaneously.

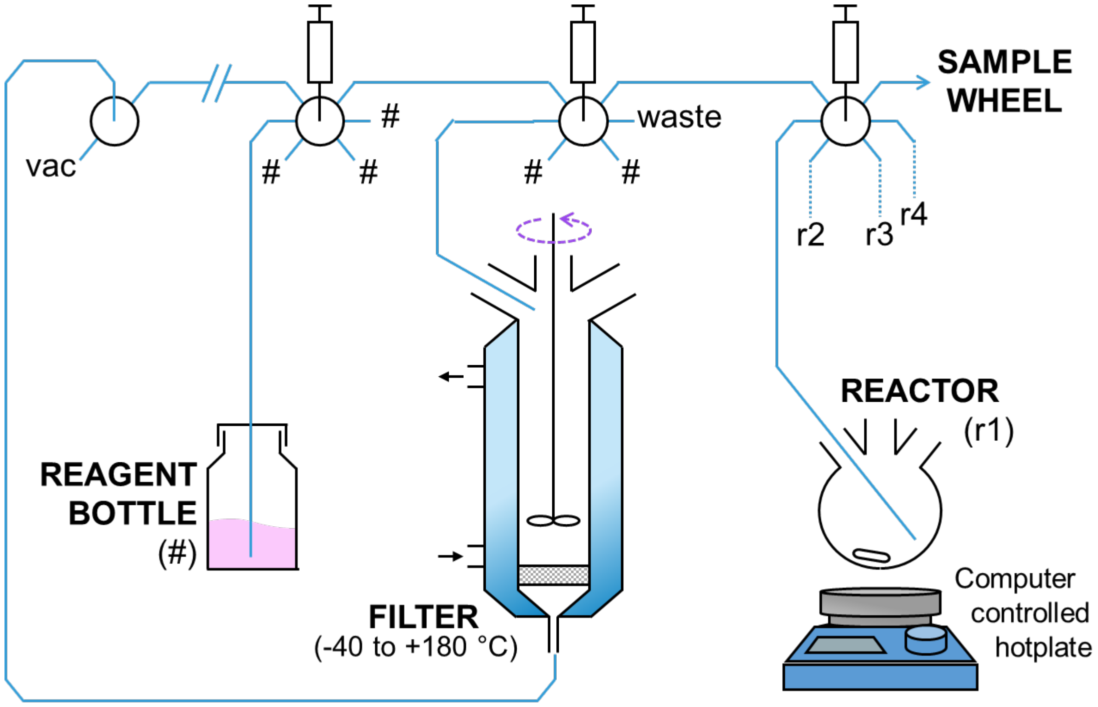

***Figure S12**: Process Diagram for the ChemPU platform layout. Reproduced from manuscript.*

Default speed at which fluids were moved throughout the platform was 40 mL min^{-1}. Unless otherwise specified, reactor modules were stirred at 300 rpm, and the jacketed filter at 150 rpm. The 'dead volume' used to prime the tubing from the backbone to the base of the filter was 10 mL.

The same configuration of the rig was used for all syntheses. However, the preparations of tris(2,2'-bipyridine)ruthenium tetrafluoroborate, {Mn_3O}, and Cisplatin required only a single reactor module and no sample wheel, whilst the preparation of the polyoxometalate species and library generation experiment required multiple reactors and the sample wheel. In the former case, the majority of the synthesis is focused on the jacketed filter, with the reactor used to permit premixing of liquid reagents or sequential solid additions.

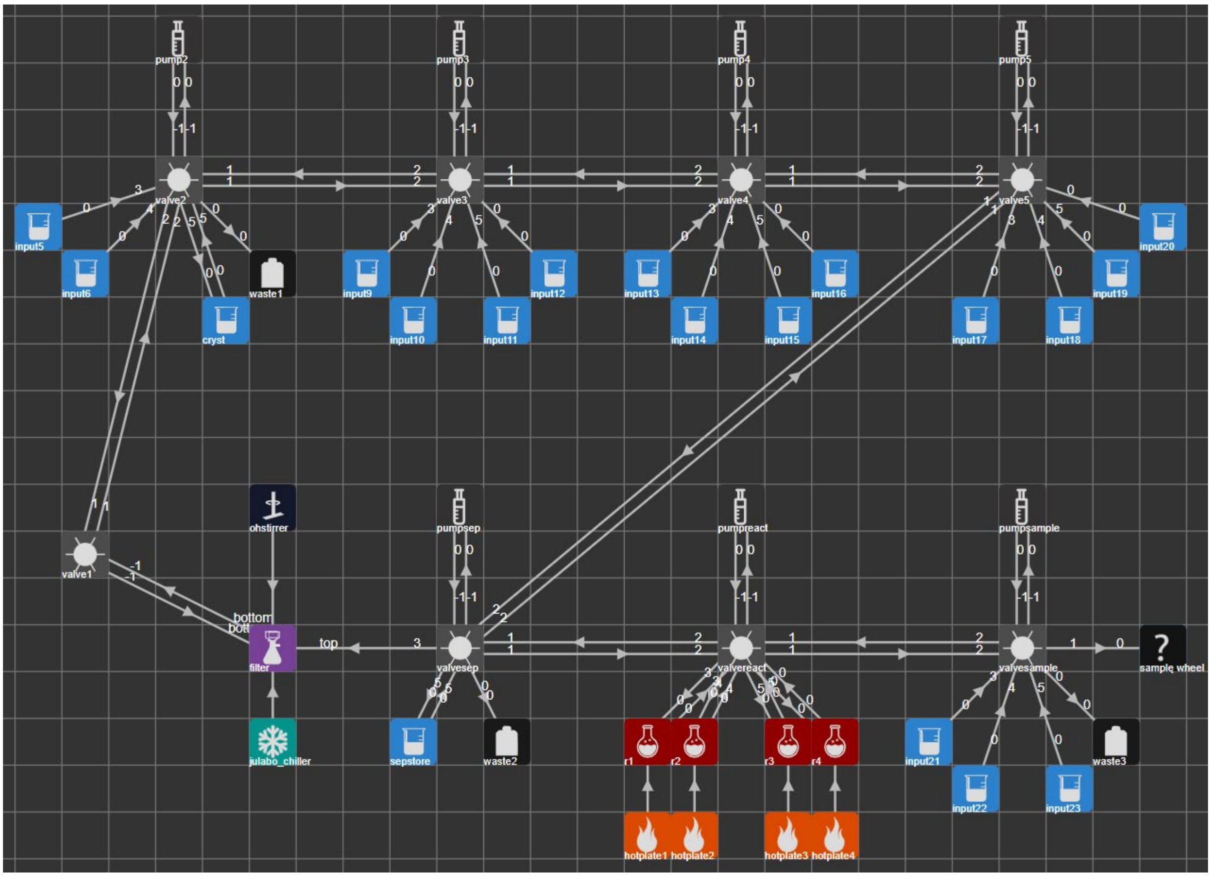

Figure S13: *Graph showing the full configuration of the ChemPU platform used in these experiments. Nodes represent pieces of hardware and the connections between these are shown as directional edges, with the port on the hardware indicated in small white characters. Ports are either integers or specific text strings (e.g. 'bottom', 'top'). Inputs (blue nodes) and waste containers (black) are connected to the backbone of pumps (dark grey) and valves (lighter grey), and can be moved through this to reach the jacketed filter (purple), or reactor modules (red). Jacketed filter is connected to a chiller (sea green) and overhead stirrer (navy), and reactors are heated by a remote controlled hotplate (orange). Inputs 5-20 are used for reagents, and inputs 21-23 for cleaning solutions. Sample wheel is depicted here as a 'custom' module, represented as a black square with '?' icon (bottom right). Image created in ChemIDE web application.*[6]

1.4 Error Testing

1.4.1 Accuracy of the Pump/Valve Combinations

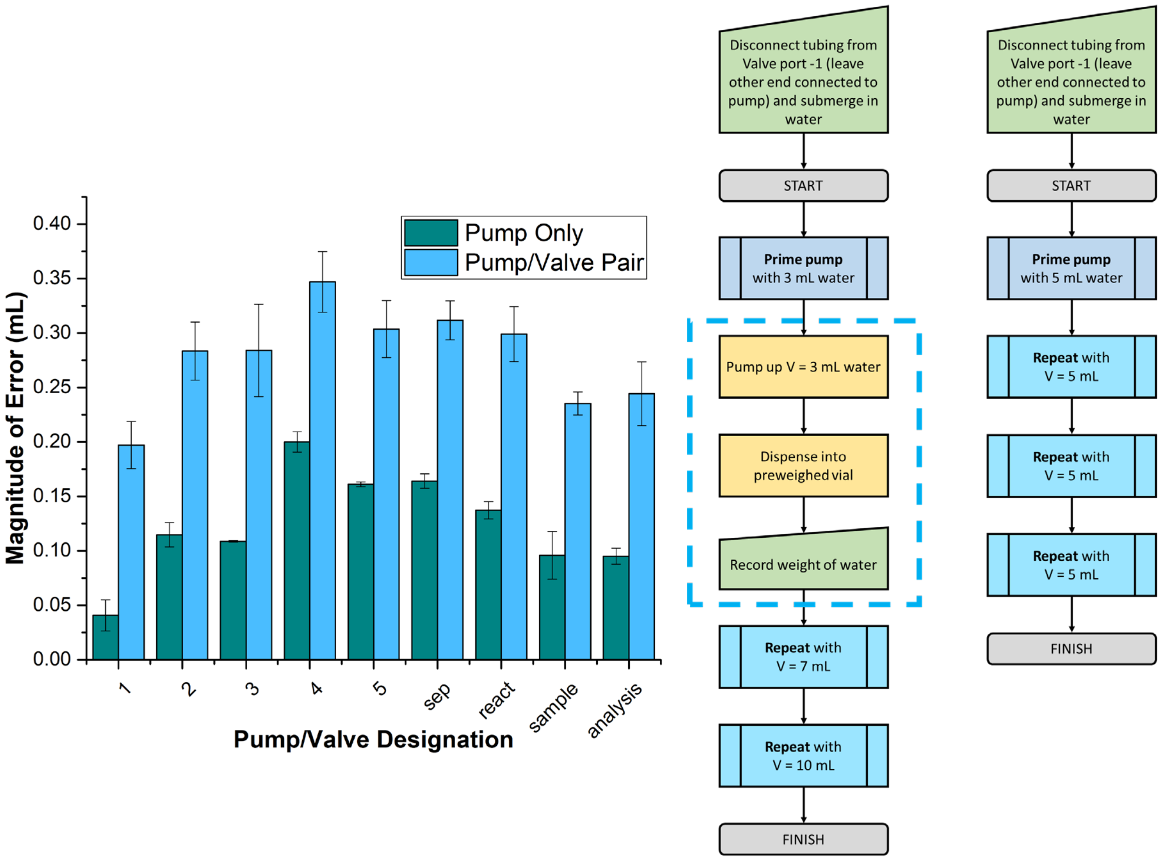

***Figure S14**: Graph showing the magnitude of error (mL) in each pump when dispensing up and down from the pump alone (described by the flowchart on the left), or via a valve (described by the flowchart on the right). The section of the flowchart outlined in a dashed blue line is equivalent to the process indicated by the boxes of that colour.*

Each pump and pump/valve pair was tested to determine a rough value for the error (and the variability of this) in the in-house 3D printed pump by pumping up and dispensing theoretically known volumes of water. The amount dispensed was weighed and compared with the theoretical value to determine an error measurement.

All samples were under the ideal value, but plots are shown with the magnitude of the error only (Fig. S14). Each measurement was repeated 3 times for each pump (with three different volumes: 3, 7, and 10 mL, and 6 for each pump/valve pairing (one aliquot of 5 mL water through each of the valve's six ports). The average of these errors is shown in Fig. S14. Error bars are calculated for the pumps only by pumping 3 x 5 mL volumes and taking the larger deviation between average and either the maximum or minimum value. For the pump/valve combination, error bars are calculated similarly from the average and maximum or minimum value of the 6 x 5 mL volumes transferred.

Use of the average is justifiable in this case as there was no correlation between the magnitude of the error noted, and the volume of water transferred (see Fig. S15).

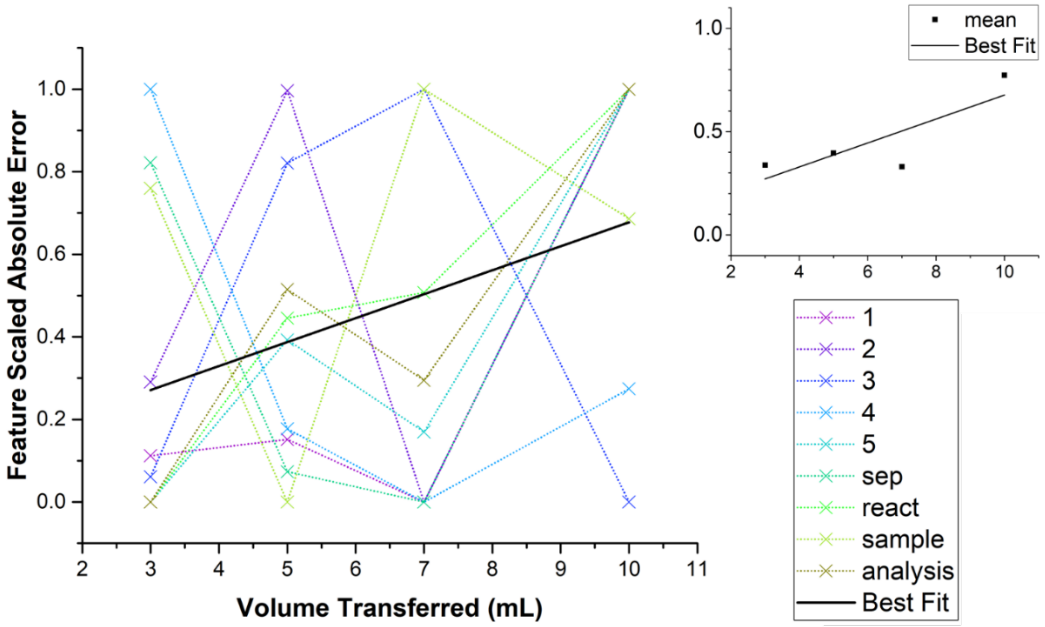

Figure S15: *Graph showing the lack of correlation between the absolute error measured for each pump and the volume transferred in the test. The insert (top right) is included to clarify that the positive trend shown by linear regression (labelled 'Best Fit') is not due to a clear correlation, but instead is biased by a higher average value of absolute error for measurements transferring 10 mL, whilst other measures have little or no change in the size of their scaled error with increasing volume transferred.*

The much larger errors for the pump/valve pair are due in part to the lengths of tubing used in this experiment. For the measurement of the error in the pumps only, the tubing length between the input water/collection vessel and pump was 18.5 cm. For the measurement of the error in the pump/valve pairings, the pump and valve were connected by an 18.5 cm length and the input water/collection vessel connected to the valve ports by 87 cm lengths. These specific lengths are the same as those used in the finished platform.

1.4.2 Error in Dispensing of a Given Volume through the Backbone

When dispensing multiple reagents across several reactors it is imperative to understand how accuracy of dispensing changes on repeated transfers as this has significant implications for contamination that must be understood for exploration to be easily replicable.

By pumping successive aliquots of water through the backbone from a completely dry start, collecting each and weighing the volume of water dispensed, we were able to gain a better understanding of the accuracy of our hardware and how effectively the system is purged (see Fig. S16).

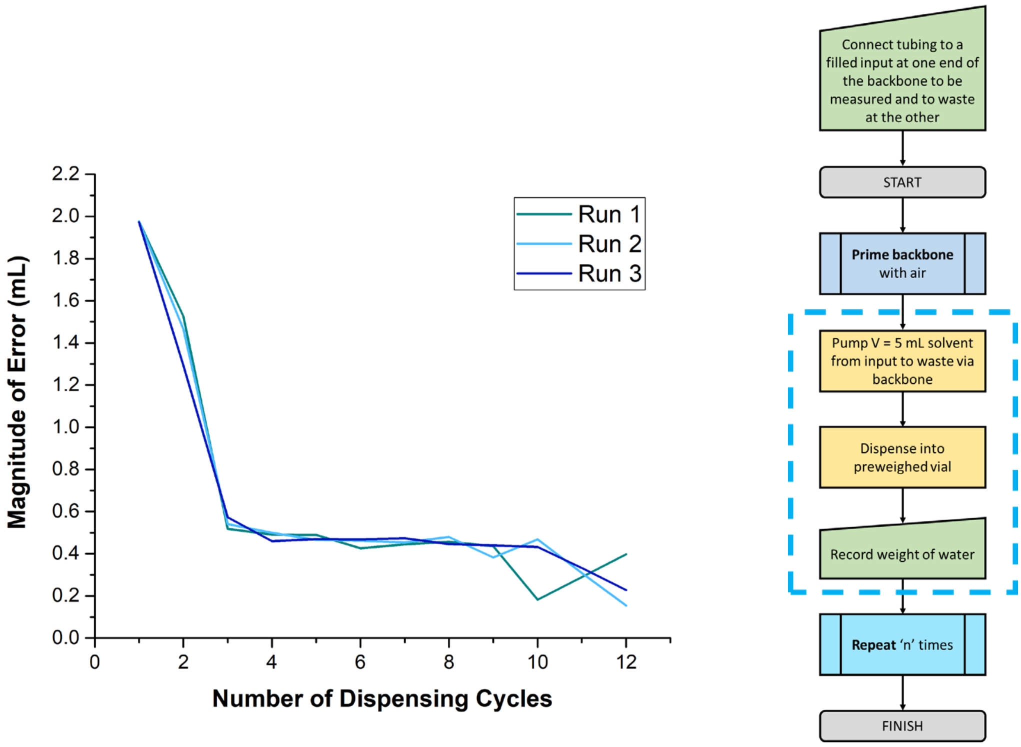

Figure S16*: Data and flowchart detailing a dispensing experiment, wherein constant volumes of water are contiguously passed along the backbone of the platform (which here comprises 9 pumps), with each aliquot weighed to determine how the error in the measurement changes.*

There is a large error in the first measurement, however this is likely due to the priming of the tubing from the input to the backbone (which can be eliminated before running a synthesis) and from the backbone to the collection vessel. This may also account for the large error in the second measurement.

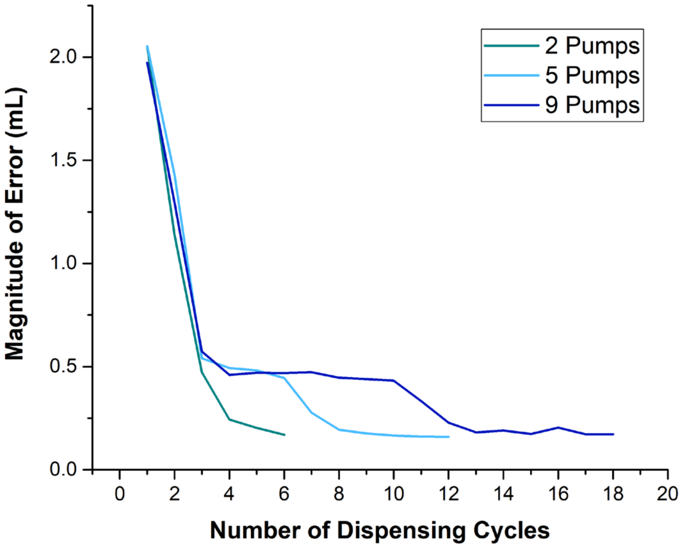

Figure S17*: Data for a similar experiment to that described in Fig. S#, but where the backbone length was varied.*

After the initial sharp reduction in error, a plateau is reached at approximately 0.5 mL for the next 7 measurements, before the error reduces to a second plateau of approximately 0.2 mL. The duration of the first plateau was subsequently shown to depend on the length of the backbone (Fig. S17), and

is therefore likely to be related to the purging of the valves themselves. It is of note however, that there is not a gradual decrease as each valve is purged in turn, only a drop off after *n+1* cycles, where *n* is the number of pumps in the backbone.

The second plateau at ~0.2 mL is taken as the irreducible error. It is worth noting that it is roughly equivalent to the very lowest error measurement for a pump/valve pairing (Fig. S14).

2 Modular Wheel Platform

2.1 Hardware

The Modular Wheel Platform comprises a cage of OpenBuilds® V-Slot Linear Aluminium Rails with laser-cut acrylic base plate (4 or 6 mm) to which a rotatable wheel (with attached acryclic vial holder) and supports are attached to permit sequential dispensing of stock solutions into vials. An in-house 3D printed tube holder is attached to the top edge of the cage to afix input tubing in the correct position.

Multiple vial sizes can be allowed for by changing the dimensions of the vial holder (and rails, if necessary) with the underlying mechanism and setup remaining constant.

For a detailed account of the hardware and construction, please refer to Salley *et al*.[7] A brief discussion of the modules present in this work follows.

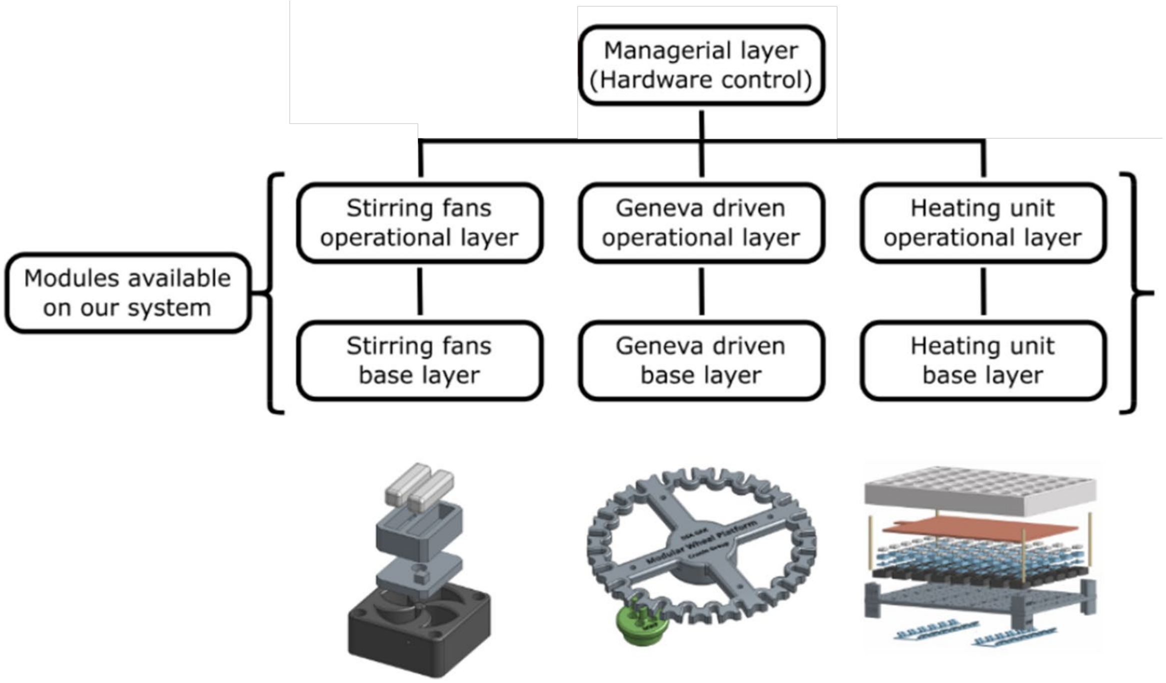

Figure S18*: Hardware modules available to the system in this work. All are controlled digitally via bespoke in-house software. Adapted from Salley* et al.[7]

2.1.1 Geneva Wheel Mechanism

The central Geneva Wheel system is constructed from a variety of in-house 3D prints produced via an Objet500 Connex printer and laser-cut acrylic (either 4 or 6 mm), with some additional fittings as required (screws, washers, nuts, bearings). A Nema17 Linear Stepper Motor is used to rotate the wheel, with the Geneva mechanism ensuring this motion occurs in a stepwise manner. For this work, a 24 position vial tray was used, comprising a circular base plate of solid acrylic and elevated plate with appropriately sized holes cut to permit vials to sit around the rim of the circular plate at regular intervals. To move stepwise through the 24 available positions a 15° Geneva drive wheel was used.

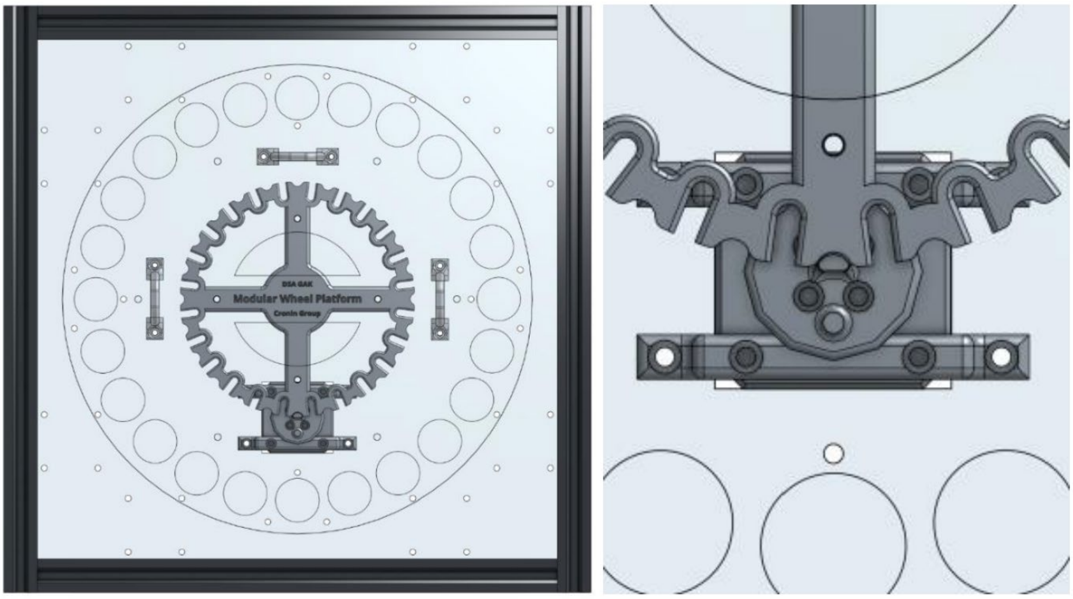

***Figure S19**: Geneva Wheel Mechanism. (Left) Overhead view of the entire platform showing 3D-printed Geneva wheel assembly and vial tray supports in filled dark grey. (Right) Close view of the Geneva mechanism. Reproduced from Salley* et al.[7]

2.1.2 Fan Stirrer

Arduino-controlled fan stirrers were constructed from 25x25 mm DC fans, with a custom 3D printed mount housing two Nd magnets. Once the initial standard 9000 rpm speed of the unhindered fan is reduced to 200-1000 rpm via a user-controlled signal, the rotating magnets are able to induce appropriate motion in 12 mm Teflon-coated magnetic stirrer bars placed in the vials.

To enable stirring during the dispensing stage, a mantle containing 24 bespoke magnetic stirrers was constructed and magnetically-mounted below the rotating vial tray. Stirrers did not move with the vial tray, but instead, as the vials were moved stepwise between positions, they were switched from a position above one stirrer to the adjacent.

Additional stirrers were fixed below the heating plate to permit agitation during heating.

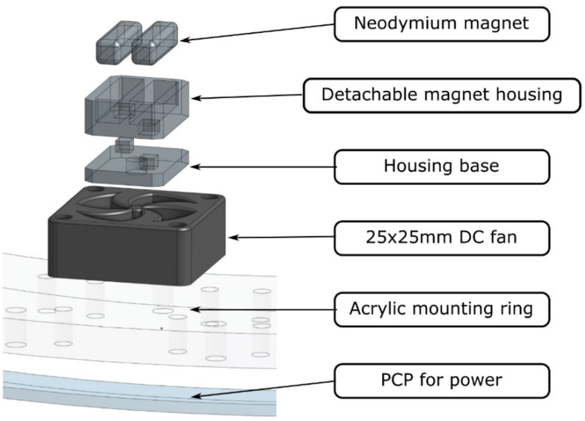

***Figure S20**: Exploded view of fan stirrer assembly. Reproduced from Salley* et al.[7]

2.1.3 Dispensing

Pumps used were commercially-available TriContinent C-Series Syringe Pumps (Model 8657-15) fitted with TriContinent Prime Series Syringes of a variety of sizes, as follows: 5 mL for cluster stock solutions and methanol, 2.5 mL for lanthanide chloride hydrate stock solutions, and 1 mL for organic stock solutions. For practicality, these were mounted on a series of shelves constructed from OpenBuilds® V-Slot Linear Aluminium Rails and laser-cut acryclic (4 or 6 mm).

Tubing for transport between stock-pump and pump-vial was 1/16" OD PTFE tubing. Flangeless fittings (polypropylene 1/4-28 flat bottom for 1/16" OD) were used to connect tubing to the pumps.

2.1.4 Vial Heater

The heater consists of a silicone heating plate within line temperature control, below a thermally-conductive single 48-well aluminium-milled unit, and above an array of fan stirrer assemblies and an acryclic base plate. PCP strips are mounted to the underside of the base plate to power the stirring assemblies.

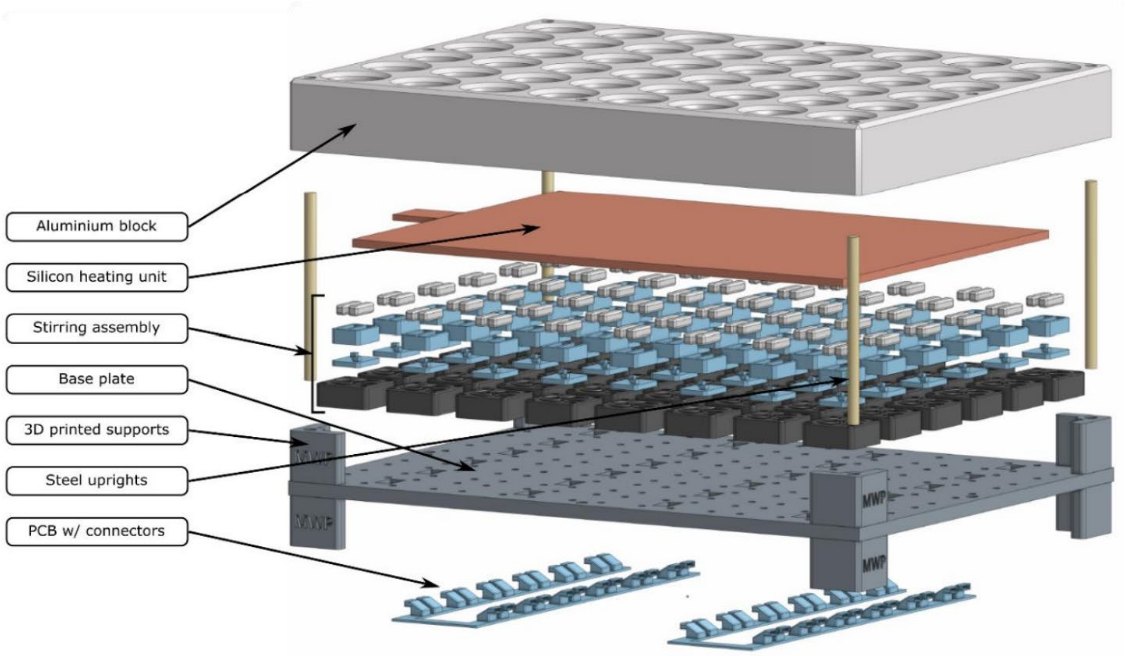

***Figure S21**: Exploded view of the vial heater. Reproduced from Salley* et al.[7]

2.1.5 Control and Power

Stepper motors, pumps, and fans were controlled via an Arduino Mega 2560/RAMPs combination. The platform was powered using a Corsair SF450 modular unit.

2.2 Software

In common with the Chemputer (discussed above), the Modular Wheel Platform permits abstraction of chemical synthesis into a digital code corresponding to a relatively small number of practical unit operations. This allows the easy generalisation of the hardware to multiple different chemical systems, provided the manipulation of these systems is limited to the unit operations possible in the Platform. Additional modules are given in Salley *et al.*[7] to extend the capabilities of the Platform beyond what is covered here. Where the operation is difficult to automate (e.g. sealing and transfer of a solvothermal reaction), the hardware may be used in tandem with manual intervention.

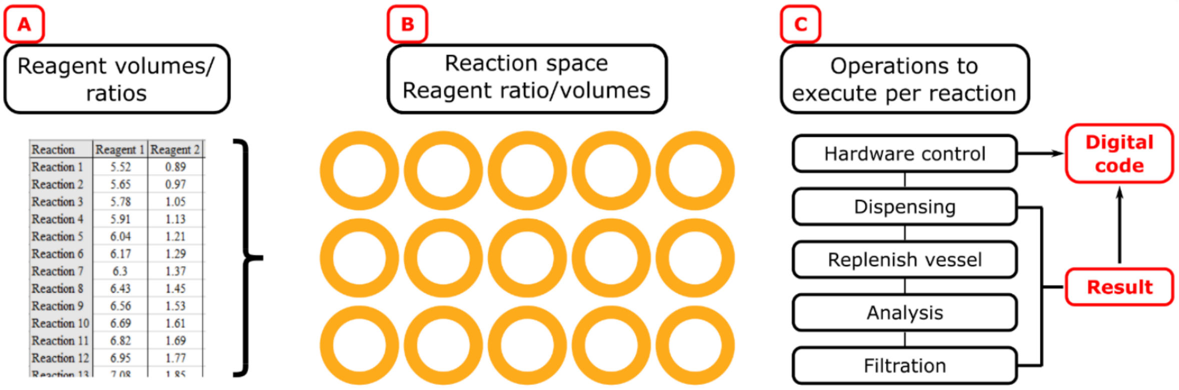

***Figure S22**: Overview of the abstraction of synthesis into digital code for use in the MWP. (A) Table format of desired reaction volumes and reagent ratios. (B) Extraction of data contained in A into individual reaction files. (C) Mapping to and Execution of the operations on the hardware. Note that filtration was omitted from the workflow shown in C for this work. Reproduced from Salley* et al.[7]

The control software utilised in this work requires two 'layers': the Experimental Layer manages the interpretation of inputs and construction of a procedure, which is then performed by the Core Layer. The Experimental layer constitutes a Base sub-layer defining a series of methods common to each experiment (the abstracted synthesis operations) and child classes of this to string these operations together into full procedures. The Core Layer consists of a central Manager permitting interaction with the hardware via specific operational modules to interface with each component.

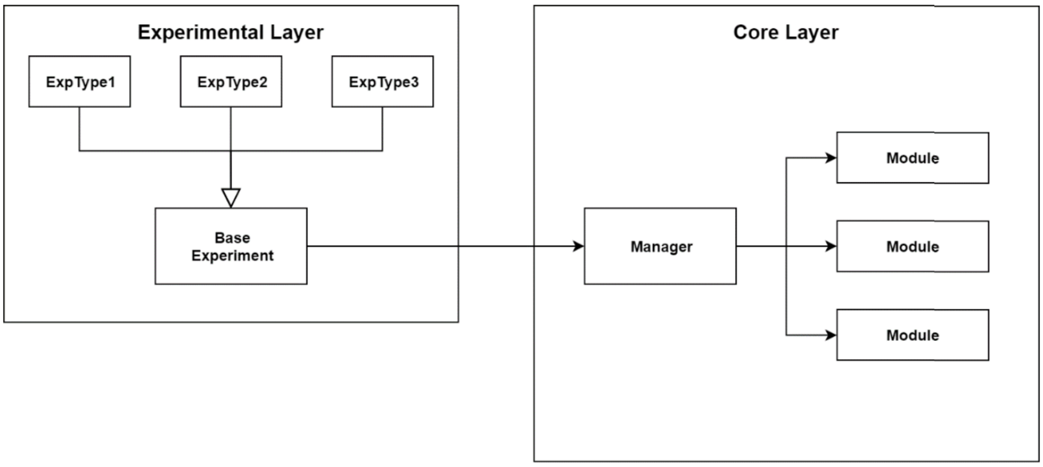

***Figure S23**: Overview of the software architecture relevant to this work. Reproduced from Salley* et al.[7]

2.3 Error Testing

The accuracy and precision of the volumes dispensed by the MWP were examining in a similar manner to the ChemPU. Specified volumes of water were dispensed into pre-weighed glass vials, and the filled vial weighed. Assuming the density of water to be 1 g cm^{-3} under measurement conditions, this permitted calculation of the volume of water dispensed.

***Table S2**: Results of error testing for the MWP when dispensing 1.00, 0.10, or 0.01 mL water.*

Exp #	Weight (g) *Vial*	*Vial + Water*	Water Volume (mL) *Desired*	*Measured*
1a	10.1195	11.1168	1.00	0.9973
1b	9.9988	10.9823	1.00	0.9835
1c	10.0527	11.0471	1.00	0.9944
2a	10.0164	10.1149	0.10	0.0985
2b	9.9364	10.0384	0.10	0.1020
2c	9.9570	10.0517	0.10	0.0947
3a	9.9182	9.9351	0.01	0.0169
3b	9.9599	9.9696	0.01	0.0097
3c	10.0953	10.1039	0.01	0.0086

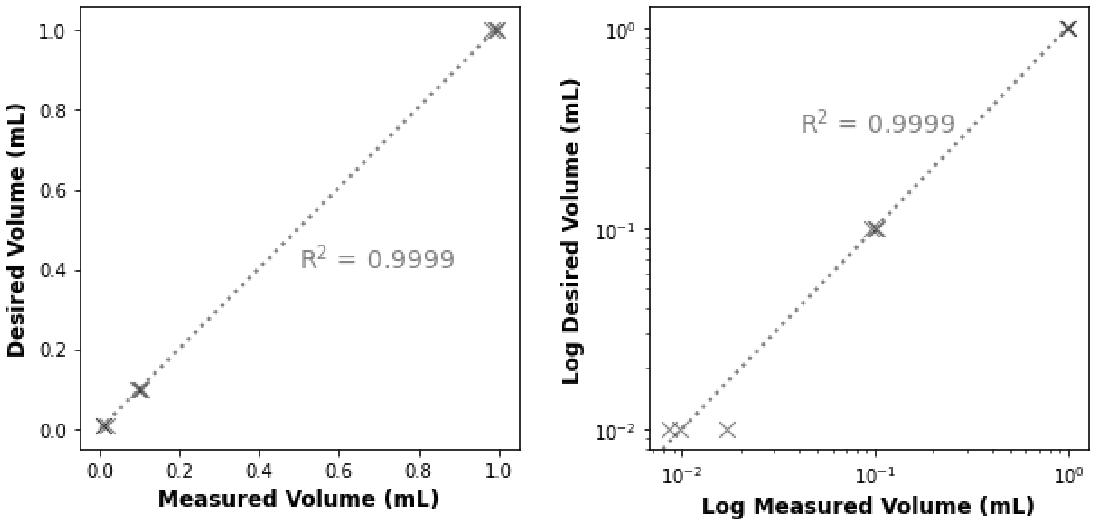

***Figure S24**: Measured volumes of water dispensed by the MWP platform, plotted against the desired volume to dispense. Linear regression is performed on these data and the resultant line, with an R^2 score approaching 1, plotted. Data is from Table S2, and is displayed on both normal axes (left), and logarithmic axes (right) to better demonstrate the relative spread of the datapoints for each desired volume.*

The data above indicate that for dispensed volumes of 1.00, and 0.10 mL there is high accuracy and precision, volumes of 0.01 mL appear to be less precise. Additional datapoints were recorded at 0.01 mL water dispensed to ensure anomalous data was not the cause of this discrepancy.

__Table S3__: Results of additional error testing for the MWP to expand the dataset for dispensing 0.01 mL water.

**Exp #**	**Weight (g)** *Vial*	*Vial + Water*	**Water Volume (mL)** *Desired*	*Measured*
3d	10.0236	10.0317	0.01	0.0081
3e	9.9884	9.9887	0.01	0.0003
3f	10.0102	10.0171	0.01	0.0069
3g	10.0563	10.0652	0.01	0.0089
3h	9.9470	9.9542	0.01	0.0072
3i	9.9764	9.9866	0.01	0.0102
3j	9.9831	9.9957	0.01	0.0126

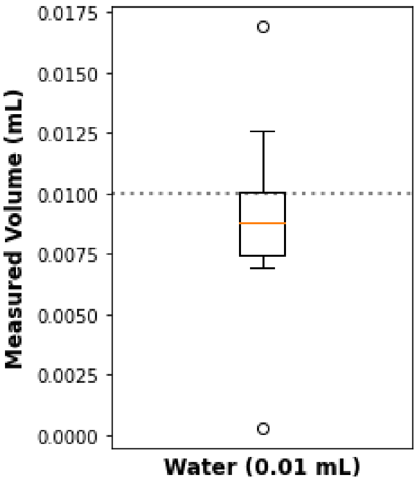

***Figure S25**: Spread of datapoints taken for 0.01 mL water. Data included from Table S2 (3a-c) and Table S3 (3d-j).*

From the expanded dataset for 0.01 mL, it can be seen that on average the amount dispensed is slightly below that desired (as might be expected). However, whilst ~80% of datapoints are within 25% of the desired volume, there are significant outliers both above and below this value.

Ultimately, it appears that the limit of precision for the MWP lies somewhere between 0.10 and 0.01 mL, with volumes equaling and above 0.10 mL appearing to be dispensed reliably.

3 Target-Oriented Syntheses of Coordination Compounds

3.1 Chemicals and Analytical Measurements

Solvents and Reagents. Used as received from commercial suppliers unless otherwise stated.

ATR-FTIR measurements. Performed with a Specac Golden Gate™ ATR single reflection accessory using a Sapphire anvil, and connected to a Thermo Scientific Nicolet™ iS5 FTIR spectrometer. All samples were recorded in air and under ambient conditions.

NMR measurements. Performed with a Bruker Avance III HD 600 spectrometer operating at 600.1 and 150.9 MHz for ^{1}H and ^{13}C respectively. Spectra were collected at 298 K, chemical shifts are reported in ppm, and were calibrated for the (residual) NMR solvent signal. Multiplicities are given as singlet (s), doublet (d), triplet (t), quartet (q) and multiplet (m), with coupling constants reported in Hz. The spectra were processed with TopSpin 4.0.5.

ESI-MS Measurements. Performed using a Bruker MaXis Impact instrument in MeOH. A standard tuning mix was used, and the machine calibrated for 100-1500 m/z. Dry gas temperature was 180 °C. All scans were recorded with positive ion polarity, and capillary tip voltage set to 50 V. Spectra were processed with Bruker's DataAnalysis 4.1 software.

MALDI Measurements. MALDI measurements were performed by the Mass Spectrometry Facility at the University of Strathclyde using a Shimadzu Biotech Axima CFR 2.8 spectrometer without a matrix.

UV-vis measurements. Performed using a Jasco V670 Spectrophotometer, outfitted with Deuterium and Halogen Light Sources. Disposable cuvettes (12.5 x 12.5 x 45 mm) from Brand were used.

XRD measurements. Suitable single crystals were selected and mounted onto a rubber loop using Fomblin or Parabar oil. Single-crystal datasets and unit cells were collected at 150(2) K on a Rigaku XtaLAB Synergy R HyPix-Arc diffractometer equipped with a graphite monochromator (λ (MoKα) = 0.71073 Å) on a microfocus X-ray source of rotating anode (50 kV, 24 mA). Data collection and reduction were performed using CrysAlisPro software package. Structure solution and refinement were carried out by using SHELXT-2018/3 and SHELXL-2018/3 on Olex2 and finalized using WinGX. Most of the non-hydrogen atoms (including those disordered) were anisotropically refined. Corrections for incident and diffracted beam absorption effects were applied using analytical numeric absorption correction on multifaceted crystal models. CCDC 2211731-2211733 contain the supplementary crystallographic data for compound reported in this paper and can be obtained free of charge via www.ccdc.cam.ac.uk./data_request/cif.

3.2 General Operation and Cleaning Procedures

3.2.1 General Notes

Tubing to stock solutions was primed before reactions were commenced, and air was used to flush through any lingering material following additions. Before use, the tubing and dead volume below the jacketed filter frit were also primed with reaction solvent, to prevent leakage of the reaction mixture below this level.

3.2.2 Jacketed Filter Module Cleaning Procedure

The following procedure was used to clean the filter module and backbone in between each reaction to attempt to prevent cross-contamination. Once reagent bottles containing the cleaning solutions are attached, the entire process is automated, taking approximately 2 h to complete. In rare cases of particularly stubborn residues, it was necessary to detach the jacketed filter and clean this manually.

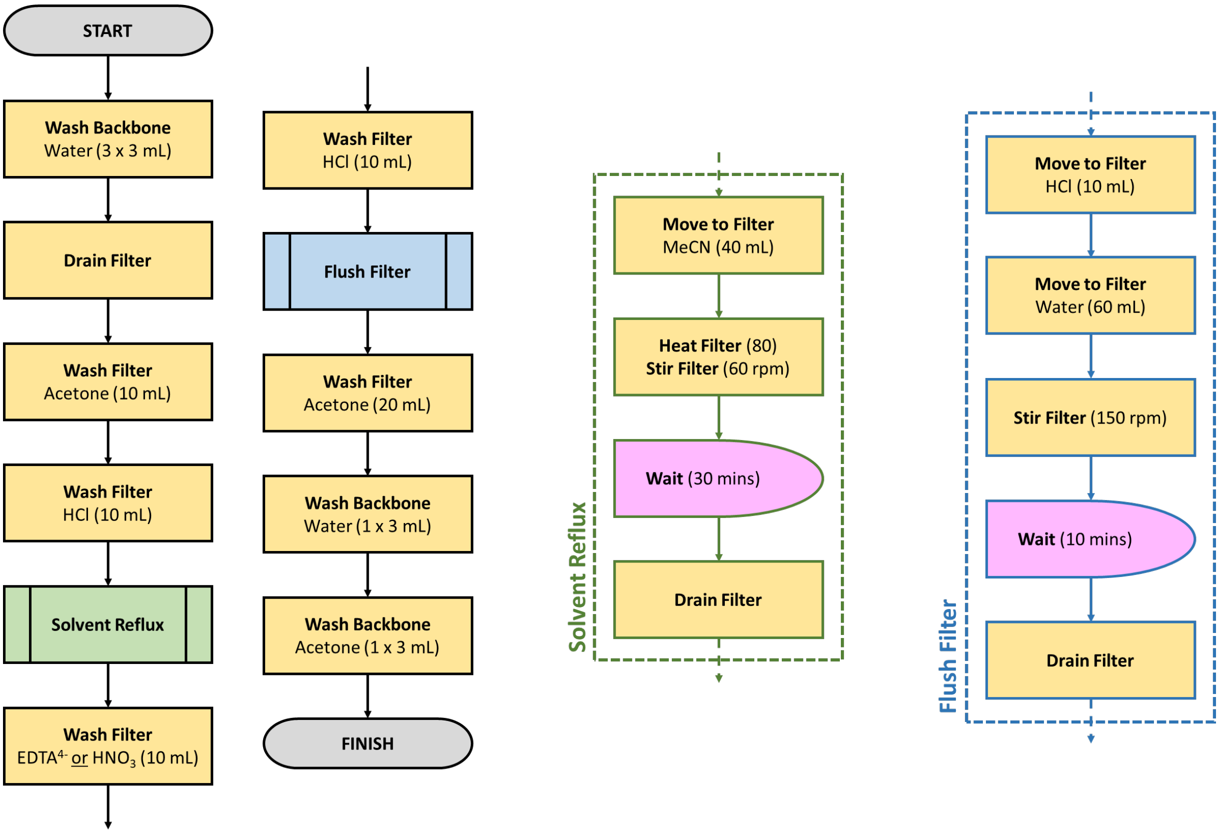

***Figure S26**: Flowchart detailing the procedure used to clean the filter module between runs.*

3.2.3 Reactor Module Cleaning Procedure

Reactor modules were washed in a similar manner to the jacketed filter.

As the reactors cannot be flushed through in the same manner as the jacketed filter (having only a single input/output), the cleaning of these relied on fewer, larger washes to dilute and remove any contamination. The input/output tubing cannot reliably empty the flask completely, and so dilution is also of key importance to counteract this. A final wash with volatile solvent and period of drying gives the smallest risk of contamination, but where the flask is needed in quick succession, a final wash with the reaction solvent for the next process sufficed.

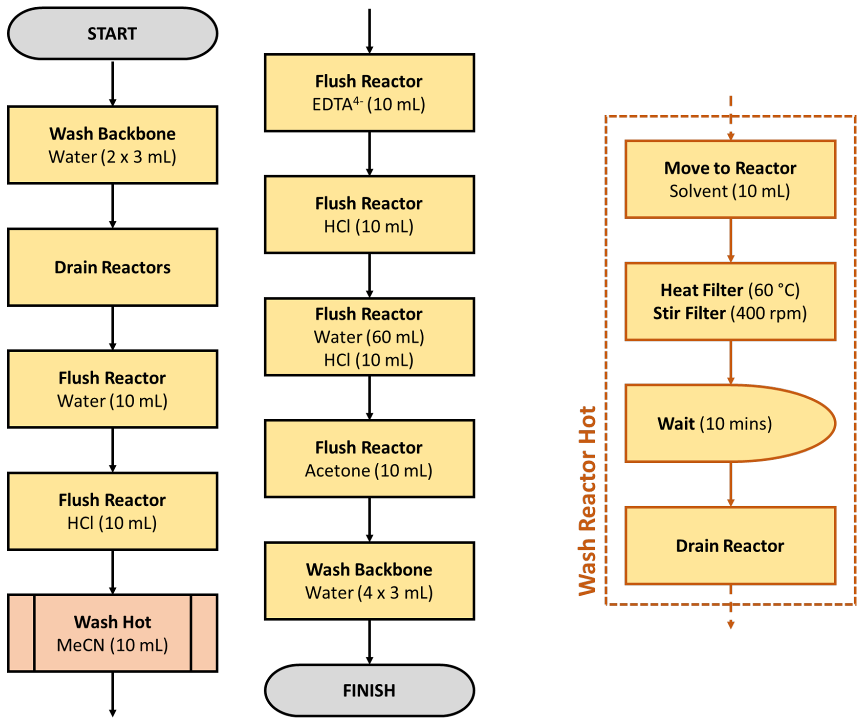

Figure S27: *Flowchart detailing the reactor washing procedure.*

3.2.4 Input Cleaning

It was also necessary to flush tubing between input bottles and backbone when these were interchanged. This process may also be automated by allowing the length of tubing to rest in an empty beaker, and then flushing a suitable solvent (usually water or acetone) into this beaker via the tubing. Flushing can be repeated multiple times until the operator is satisfied the tubing is clean, and with any combination of solvents.

3.2.5 Washing Solid Residues

Where filtration left a solid residue, which was then subsequently washed with solvent, the following general procedure was used.

A specified volume of solvent was moved to the filter via the top port at 70 mL min^{-1}. Once addition was complete, the solvent was removed from the bottom port of the filter at a rate of 20 mL min^{-1}. To ensure all solvent was transferred, it was necessary to instruct the platform to move an amount equal to the original volume of solvent multiplied by 1.6. This number was then usually rounded up to the nearest multiple of 10. The removed solvent was disposed of to waste, and the rig paused for 15 secs (to permit any remaining solvent to drain to the base of the filter) before one final 10 mL aliquot was removed to waste.

3.3 Preparation of Metastable Calcium Carbonate Polymorphs

This target was accessed via an adaptation of a literature procedure.[8]

Setup: Individual 250 mL reagent bottles were charged with (i) reagents: aqueous calcium chloride dihydrate solution, aqueous potassium carbonate solution, (ii) solvents: deionised water and acetone. All were stored under ambient conditions in air, and introduced to the platform in large excess. Cleaning was conducted manually for these procedures, due to the need for mechanical action to effectively remove the calcium carbonate residue.

3.3.1 Vaterite

Automated Procedure: Tubing from each input to the backbone was primed by transferring 3 mL of each input to waste. As the two reagent solutions precipitate rapidly if combined, the sequence for priming was as follows: (i) water, (ii) aqueous calcium chloride dihydrate, (iii) water, (iv) acetone, (v) water, (vi) aqueous potassium carbonate, (vii) water, and (viii) acetone. Chemical inputs were located on separate pumps to reduce the opportunities for accidental contamination. Backbone was rinsed by transferring water (2 x 3 mL) from the relevant input bottle to waste containers at either end of the backbone, and tubing from the backbone to the base of the filter primed with water (10 mL).

Aqueous potassium carbonate solution (10.00 mL, 2.0 M, 20.0 mmol, Eq: 1) was transferred to the filter and flushed through with air. The backbone was washed with water (4 x 3 mL) to remove traces of the carbonate solution. The filter heated to 30 °C with stirring at 300 rpm for 15 mins (to ensure temperature was reached before subsequent additions).

With heating and stirring maintained, aqueous calcium chloride dihydrate (10.00 mL, 2.0 M, 20.0 mmol, Eq: 1) was transferred to the filter at a rate of 25 mL/min (as opposed to the usual 40) and flushed through with air. Precipitation was observed immediately on contact between the two solutions. The mixture was permitted to stir for 7 mins at 30 °C, and the backbone was washed with water (4 x 3 mL).

The reaction mixture was then filtered, leaving a white precipitate of calcium carbonate (as vaterite), and washed with water (6 x 6.7 mL), exposed to vacuum to remove lingering solvent for 5 secs, washed with acetone (3 x 4 mL), and exposed to vacuum for a further 5 secs. Heating was ceased, and the filter drained further to ensure no remaining solvent in vessel or tubing.

Vaterite was collected as a white precipitate (1.42 g, 14.2 mmol, 71%) and analysed via IR.

IR $\bar{\nu}$/cm^{-1}: 1490 (sh), 1462 (sh), 1432 (sh), 1399 (s), 1088 (w) 873 (s), 849 (w), 744 (m)

3.3.2 Aragonite

Automated Procedure: Procedure uses vaterite as starting material, and was begun by running the procedure for vaterite above. The filtered and washed vaterite was left in the jacketed filter and this polymorph then converted to aragonite as follows.

Backbone was washed with water (4 x 3 mL) and additional water transferred to the jacketed filter (95.24 mL). Filter was then heated to 100 °C with stirring at 300 rpm for 2 h. Heating and stirring were then ceased and the apparatus paused for 10 mins to permit the white precipitate to sediment.

This was then filtered, and washed with water (6 x 6.7 mL), acetone (3 x 4 mL), and further drained to ensure complete removal of the solvent.

Aragonite was collected as a white precipitate (1.54g, 15.4 mmol, 77%) and analysed via IR.

IR $\bar{\nu}/cm^{-1}$: 1787 (w), 1698 (m), 1649 (w), 1522 (sh), 1462 (sh), 1441 (s), 1365 (sh), 1236 (m), 1083 (m), 851 (s), 843 (sh), 739 (w) 712 (m), 699 (m). Additional broad peak at 3386 (br s) – likely due to small amounts of water.

3.3.3 Comparative IR Data

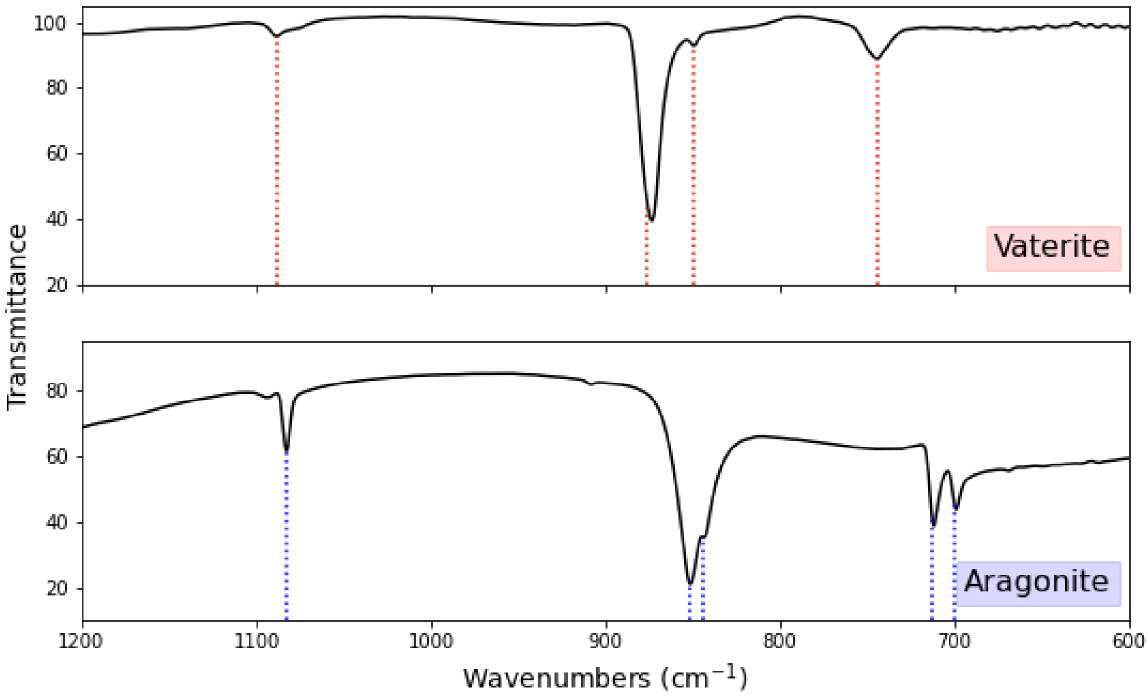

***Figure S28**: Fingerprint region for each polymorph, with the characteristic vibrations for the relevant assignment shown as coloured, dashed lines (as detailed in Sarkar and Mahapatra [8]).*

3.4 Target 1: Tris(2,2'-bipyridine)ruthenium tetrafluoroborate

This target was accessed via a 2-step synthesis, adapted from an in-house procedure.

3.4.1 Bis(2,2'-bipyridine)dichlororuthenium(II), $Ru(bpy)_2Cl_2.xH_2O$

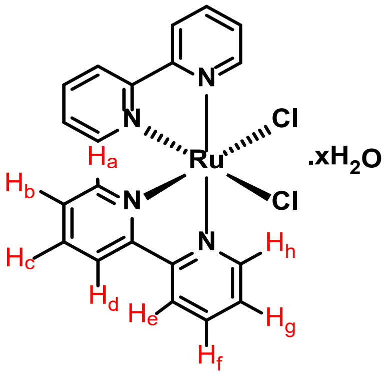

Setup: Jacketed filter was loaded manually with ruthenium trichloride hydrate (0.78 g, 3.0 mmol, Eq: 1.0). Individual 250 mL reagent bottles were charged with (i) reagents: 1.1 M 2,2'-bipyridyl solution in DMF, 2 M LiCl solution in DMF, (ii) solvents: DMF, diethyl ether, deionised water, acetone, and (iii) cleaning solutions: 0.1 M $HCl_{(aq)}$ solution, 0.05 M $Na_4EDTA_{(aq)}$ solution, and acetonitrile. All were stored under ambient conditions in air, and introduced to the platform in large excess.

Automated Procedure: Tubing from each input to the backbone was primed by transferring 3 mL of each input to waste. Backbone was rinsed by transferring DMF (2 x 3 mL) from the relevant input bottle to waste containers at either end of the backbone. Tubing from the backbone to the base of the filter module was then primed with DMF (10 mL).

2,2'-bipyridyl solution in DMF (5.45 mL, 1.1 M, 6.0 mmol, Eq: 2), and LiCl solution in DMF (10.88 mL, 2 M, 21.75 mmol, Eq: 7.25) were added to the jacketed filter containing ruthenium trichloride hydrate, and additions washed through the system with a small amount of additional DMF (0.15 mL). The backbone was then washed again with DMF (2 x 3 mL), and the filter heated to 160 °C with stirring at 150 rpm for 3 h. On completion, the filter was left to stand for 3 h to cool slowly to ambient temperature.

Acetone (49.2 mL) was transferred to the filter and the vessel then further cooled to 4 °C and stirred at 30 rpm for 9 hours to induce crystallisation. The resulting precipitate was filtered (by transferring the solution from the filter to waste), and washed with water (2 x 20 mL) and diethyl ether (20 mL). The product was left to air dry and then manually collected from the filter vessel to afford a blackish powder (1.03 g, 1.98 mmol, 66%).

IR $\bar{\nu}/cm^{-1}$: 3467 (br s), 3099 (w), 3067 (w), 3041 (w), 1612 (w), 1600 (m), 1457 (m), 1442 (s), 1417 (s), 1307 (m), 1260 (m), 1242 (w), 1155 (w), 1122 (m), 1017 (s), 976 (w), 962 (w), 902 (w), 886 (w), 798 (m), 762 (s), 728 (s), 656 (s)

^{1}H NMR (600 MHz, D_2O) δ/ppm: 9.38 (d, *J* = 5.5 Hz, 2H, h), 8.59 (d, *J* = 8.1 Hz, 2H, e), 8.38 (d, *J* = 8.1 Hz, 2H, d), 8.26 (td, *J* = 7.8, 1.5 Hz, 2H, f), 7.90 (ddd, *J* = 7.6, 5.6, 1.3 Hz, 2H, g), 7.79 (td, *J* = 7.9, 1.4 Hz, 2H, c), 7.74 (d, *J* = 6.0 Hz, 2H, a), 7.11 (ddd, *J* = 7.5, 5.9, 1.4 Hz, 2H, b)

3.4.2 Tris-(2,2'-bipyridyl)ruthenium tetrafluoroborate hexahydrate, $[Ru(bpy)_3](BF_4)_2.6H_2O$

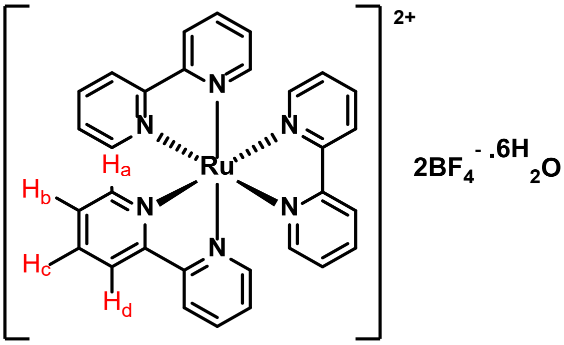

Setup: The jacketed filter was loaded manually with bis(2,2'-bipyridine)dichlororuthenium(II) dehydrate (0.26 g, 0.50 mmol, Eq: 1.0). Individual 250 mL reagent bottles were charged with (i) reagents: 0.2M 2,2'-bipyridyl solution in ethanol, saturated aqueous $NaBF_4$ solution, (ii) solvents: ethanol, diethyl ether, water, and (iii) cleaning solutions: 0.1 M $HCl_{(aq)}$ solution, 0.05 M $Na_4EDTA_{(aq)}$ solution, acetonitrile, and acetone. All were stored under ambient conditions in air, and introduced to the platform in large excess.

Automated Procedure: Tubing from each input to the backbone was primed by transferring 3 mL of each input to waste. Backbone was rinsed by transferring ethanol (2 x 3 mL) from the relevant input bottle to waste containers at either end of the backbone. Tubing from the backbone to the base of the filter module was then primed with ethanol (10 mL).

In the jacketed filter, bis(2,2'-bipyridine)dichlororuthenium(II) was dissolved in ethanol (12 mL) with heating at 85 °C and stirring at 150 rpm for 15 min. A solution of 2,2'-bipyridyl in ethanol (2.50 mL, 0.2 M, 0.50 mmol, Eq: 1.0) was added as one portion to the newly formed solution, and flushed through the backbone to the filter flask with a further aliquot of ethanol (2 mL). The backbone was then washed with further ethanol (2 x 3 mL). Throughout the addition the filter was kept at temperature, and then held at temperature and stirred at 150 rpm for a further 24 h. In order to prevent the reaction running dry, additional ethanol (14 mL) was added every 4.8 h (for a total of 4 additions). Intervals were chosen to maintain a maximum volume approximately equal to the starting volume.

After the reaction was complete, saturated aqueous $NaBF_4$ solution was primed (by transferring 3 mL to waste), and an aliquot added directly to the filter module (1.32 mL). The tubing was flushed with air to ensure a complete transfer, and the backbone then cleaned with ethanol (2 x 3 mL). The reaction mixture was stirred at 30 rpm for 10 min at r.t., during which a fine orange precipitate of the tetrafluoroborate formed. The automated reaction paused at this point, and was manually resumed after an arbitrary time, to permit confirmation that precipitation had occurred.

The jacketed filter was primed with a further aliquot of ethanol (10 mL) to ensure no product in the solution phase had diffused below the filter frit. Additional ethanol (72.2 mL) was then added and the mixture heated to 100 °C with stirring at 150 rpm for 15 min to redissolve the tetrafluoroborate salt. Reaction mixture was permitted to recrystallize overnight in the filter at r.t., forming large, orange-red crystals, better suited for filtration. The mixture was duly filtered, and the precipitate washed with EtOH (2 x 10 mL) and diethyl ether (10 mL). The product was left to air dry and then manually collected from the filter vessel to afford orange-red crystals presumed to be the hexahydrate (0.17 g, 0.20 mmol, 40%).

IR $\bar{\nu}/cm^{-1}$: 3101 (br w), 1603 (m), 1463 (s), 1444 (s), 1424 (s), 1313, 1285, 1243, 1000 (br s), 887 (w), 805 (w), 763 (s), 730 (s), 659 (m), 648 (m)

^{1}**H NMR** (600 MHz, D_2O) δ/ppm: 8.55 (d, *J* = 8.2 Hz, 2H, d), 8.07 (td, *J* = 8.0, 1.5 Hz, 2H, c), 7.85 (ddd, *J* = 5.7, 1.5, 0.6 Hz, 2H, a), 7.39 (ddd, *J* = 7.6, 5.7, 1.2 Hz, 2H, b)

3.5 Target 2: Trinuclear oxo-centred Mn(III) Acetate, {Mn_3O}

This target was accessed via a 1-step synthesis, adapted from a procedure by Vincent *et al.*[9]

3.5.1 Tetra-*n*-butylammonium permanganate, $TBAMnO_4$

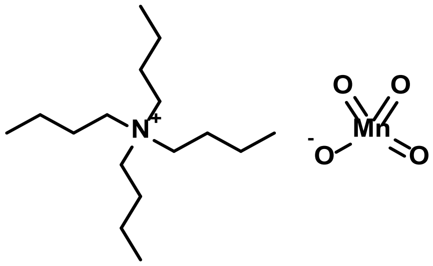

Prepared manually according to the method from Vincent *et al.*[9]

In a 250 mL beaker, a solution of potassium permanganate (2.5 g, 15.8 mmol, Eq: 1.0) in water (35 mL) was prepared and combined with a separately prepared solution of tetra-*n*-butylammonium bromide (6.0 g, 18.6 mmol, Eq: 1.2) in water (65 mL). The mixture was stirred vigorously for 10 min, during which time a purple precipitate of the salt metathesis product was evolved. This suspension was filtered over paper, the residue washed thoroughly with water and diethyl ether, and left overnight to air-dry. The purple precipitate was then collected and dried *in vacuo* for several hours, affording a purple powder of $TBAMnO_4$ (4.77 g, 25.1 mmol, Quant).

As per the literature, the material was stored at 4 °C in a sealed bottle until ready for use, and assumed to be stable under these conditions for 2 weeks.

IR $\bar{\nu}$/cm^{-1}: 2960 (m), 2934 (sh), 2873 (m), 1470 (m), 1381 (w), 1168 (w), 1108 (w), 1065 (w), 1031 (w), 894 (s), 738 (m)

3.5.2 $[Mn_3(\mu_3\text{-}O)(OAc)_6(pyr)_3]ClO_4$

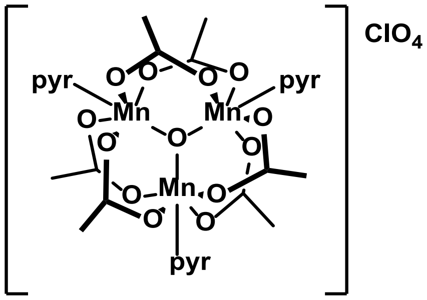

Setup: A reactor module was loaded manually with manganese(II) acetate tetrahydrate (1.00 g, 4.07 mmol, Eq: 1.0), and the jacketed filter with tetra-*n*-butylammonium permanganate (0.60 g, 1.63 mmol, Eq: 0.4). Individual 250 mL reagent bottles were charged with (i) reagents: acetic acid, pyridine, saturated ethanolic sodium perchlorate solution, (ii) solvents: ethanol, diethyl ether, water, acetone, and (iii) cleaning solutions: 0.1 M $HCl_{(aq)}$ solution, 0.05 M $Na_4EDTA_{(aq)}$ solution, and acetonitrile. All were stored under ambient conditions in air, and introduced to the platform in excess.

Automated Procedure: Tubing from a selection of inputs (pyridine, acetic acid, diethyl ether, water, acetone) to the backbone were primed by transferring 3 mL of each input to waste. Backbone was rinsed by transferring ethanol (2 x 3 mL) from the relevant input bottle to waste containers at either end of the backbone.

In the reactor, manganese(II) acetate tetrahydrate was diluted with ethanol (8 mL), pyridine (2 mL), and acetic acid (6 mL) and the tubing flushed through with air to ensure efficient addition. The mixture was then held at r.t. and stirred at 300 rpm for 10 min.

Tubing from the backbone to the base of the filter module was then primed with ethanol (10 mL), and the entirety of the reaction mixture transferred from the reactor to the jacketed filter (via the top port) to combine with solid tetra-*n*-butylammonium permanganate. The permanganate decomposes rapidly one dissolution in ethanol. Additional ethanol (2.0 mL) was used to wash the mixture into the filter. The combined mixture was held at r.t. and stirred at 150 rpm for 15 min.

Saturated ethanolic sodium perchlorate solution was primed as above and then combined (3.0 mL, 0.95 M, 2.85 mmol, Eq: 0.7) with the reaction mixture in the jacketed filter. Air was used to flush through the perchlorate solution to ensure efficient addition, and the backbone washed with ethanol (2 x 3 mL). The mixture was allowed to stir at 150 rpm for 10 min at r.t. to ensure efficient mixing, and then held at r.t. without stirring for 3 h to permit precipitation. The reaction mixture was then filtered, and the brown precipitate washed with ethanol (10 mL), and diethyl ether (2 x 10 mL).

The tubing between backbone and base of the filter was primed with acetone (10 mL), and additional acetone (30 mL) added to the filter via the top port to recrystallize the crude product. The filter was then heated to 60 °C with stirring at 150 rpm for an initial 20 min, and then without stirring for a further 2 h to dissolve the precipitate. The solution was then cooled to 4 °C and held at this temperature without stirring for 1 h to promote recrystallisation. The mixture was then filtered and washed with diethyl ether (10 mL) to afford brown microcrystals (0.58 g, 0.66 mmol, 49%).

IR $\bar{\nu}$/cm^{-1}: 1600 (s), 1489 (w), 1448 (m), 1389 (s), 1341 (s), 1220 (m), 1159 (w), 1094 (s), 1066 (s), 1045 (m) 1018 (m), 946 (w), 766 (m), 694 (s), 662 (s), 650 (s), 616 (s)

3.6 Target 3: Cisplatin

This target was accessed via a 2-step synthesis, adapted from a procedure for the ^{15}N analogue from Boreham *et al.*[10]

3.6.1 Stability of KI(aq) Stock Solution

Initial attempts at the preparation of *cis*-diamminediiodoplatinum(II) failed, and it was observed that the KI(aq) solution had discoloured. Potassium Iodide is unstable in aqueous solution over extended periods, as it decomposes to form a variety of oxidising iodine species, but the speed of decomposition (appearing instantaneous) was concerning in this case.[11]

To ascertain the cause, a selection of samples was prepared to compare the behaviour of pure KI(aq) with those exposed to acetic acid (which had been connected in the same position for the previous run), and/or sonicated for 1 min (probed to determine whether gentle warming and mechanical disruption would trigger decomposition). An additional sample of solid KI in acetic acid was also prepared.

KI(aq)	KI(aq) + AcOH	KI(aq) sonicated	KI(aq) + AcOH sonicated	KI + AcOH

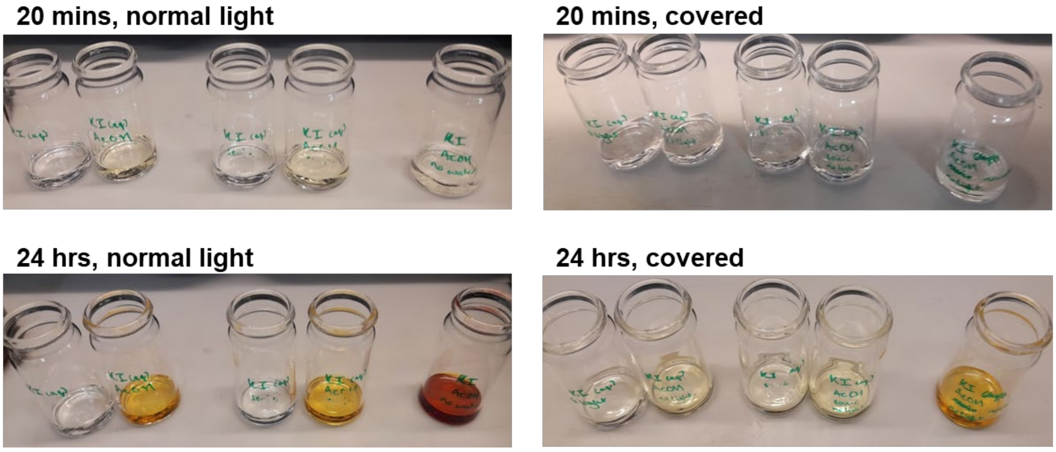

Figure S29: *(Top) Contents of each vial in the order shown below. (Bottom) Images demonstrating the decolourisation of the various samples after 20 min and 24 h. After 20 min, samples of KI(aq) and AcOH exposed to light had faintly discoloured, although the sample of solid KI in AcOH did not appear to have discoloured notably. The latter is likely due to the poor solubility of solid KI in pure AcOH. Sonication made no difference to the rate of decomposition. After 24 h, all solutions containing AcOH had discoloured to various extents, irrespective of exposure to light.*

After 20 min, it became apparent that the acetic acid seemed to catalyse the decomposition of KI(aq) solution when exposed to light. In darkness, however, the decomposition of all samples appeared to occur at a reduced rate. Thus, the tubing between reagent bottle and backbone was flushed copiously with water, to remove any traces of acetic acid, and the bottle covered with foil during the automated experiment to further reduce the likelihood of decomposition.

3.6.2 *cis*-diamminediiodoplatinum(II), [Pt(NH_3)$_2$$I_2$]

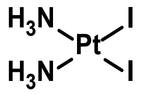

<u>*Setup*</u>: The jacketed filter was loaded manually with potassium tetrachloroplatinate(II) (0.99 g, 2.63 mmol, Eq: 1.0). Individual 250 mL reagent bottles were charged with (i) reagents: saturated aqueous potassium iodide solution, 1.03 M ammonium chloride in 1 M aqueous potassium hydroxide, (ii) solvents: water, diethyl ether, ethanol, and (iii) cleaning solutions: 0.1 M $HCl_{(aq)}$ solution, 0.05 M $Na_4EDTA_{(aq)}$ solution, acetonitrile, and acetone. The potassium iodide solution was refreshed before every run and the bottle wrapped in tin foil to slow decomposition (see above). All other reagents were stored under ambient conditions in air, and introduced to the platform in large excess.

<u>*Automated Procedure*</u>: Tubing from a selection of inputs (potassium iodide solution, ammonium chloride in aqueous potassium hydroxide) to the backbone were primed by transferring 3 mL of each input to waste. Backbone was rinsed by transferring water (2 x 3 mL) from the relevant input bottle to waste containers at either end of the backbone. Tubing from the backbone to the base of the filter module was then primed with water (10 mL).

In the filter module, potassium tetrachloroplatinate(II) was combined with water (5.52 mL) and the resultant mixture stirred at 150 rpm for 5 min to allow dissolution, forming a red solution. To this solution was added saturated aqueous potassium iodide solution (3.06 mL, ~6.0 M, 18.4 mmol, Eq: 7.0) and this washed into the filter with water (2 mL). The filter module was then heated to 40 °C with stirring at 150 rpm for 15 min. Without cooling, a solution of ammonium chloride in 1M aqueous potassium hydroxide (10.20 mL, 1.03 M, 10.5 mmol, Eq: 4.0) was added to the filter module and washed through with water (2 mL). The heating was then stopped and the cooling mixture stirred at 150 rpm for 30 min. Stirring was then paused and the mixture cooled further to 4 °C for 15 min to induce precipitation. The automated reaction paused at this point, and was manually resumed after an arbitrary time, to permit confirmation that precipitation had occurred.

Following this, a further selection of inputs were primed (acetone, diethyl ether, ethanol) in the same manner as above. The reaction mixture was then filtered, and the resulting precipitate washed with water (2 x 10 mL), ethanol (10 mL), and diethyl ether (10 mL) to afford the product as a mustard yellow powder (0.95 g, 1.97 mmol, 75%).

IR $\bar{\nu}$/cm^{-1}: 3273 (s), 3211 (s), 3169 (s), 1597 (br m), 1312 (w), 1289 (s), 1273 (s), 825 (w), 755 (br s)

3.6.3 Cisplatin, $[Pt(NH_3)_2Cl_2]$

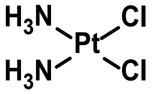

Setup: The reactor module was loaded manually with *cis*-diamminediiodoplatinum(II) (0.42 g, 0.87 mmol, Eq: 1.0) and silver nitrate (0.50 g, 1.74 mmol, Eq: 2.0), and the jacketed filter with potassium chloride (0.19 g, 2.61 mmol, Eq: 3.0). The reactor vessel was then covered in tin foil to prevent exposure to light and subsequent degradation of silver nitrate. Individual 250 mL reagent bottles were charged with (i) solvents: water, diethyl ether, ethanol, and (ii) cleaning solutions: 0.1 M $HCl_{(aq)}$ solution, 0.05 M $Na_4EDTA_{(aq)}$ solution, acetonitrile, and acetone. All other reagents were stored under ambient conditions in air, and introduced to the platform in large excess. The end of the tubing from backbone to reactor module was connected to a Thermo Scientific™ Bottom-of-the-Bottle™ solvent filter, which was present in the reactor from the start of the automated process. This filter is not resilient enough to be used for multiple runs with this chemical system and must be replaced each time this step is attempted.

Automated Procedure: Tubing from a selection of inputs (acetone, diethyl ether, ethanol) to the backbone were primed by transferring 3 mL of each input to waste. Backbone was rinsed by transferring water (2 x 3 mL) from the relevant input bottle to waste containers at either end of the backbone.

In the covered reactor module, the mixture of solid *cis*-diamminediiodoplatinum(II) and silver nitrate was dissolved in water (3.5 mL) and air used to ensure full transfer. The reactor was then held at r.t. and stirred at 150 rpm for 16 h. The automated reaction paused at this point, and was manually resumed after an arbitrary time, to permit observation of the filtration step.

Tubing from the backbone to the base of the filter module was then primed with water (10 mL), and the contents of the reactor diluted with additional water (5 mL) and transferred to the filter module via the Bottom-of-the-Bottle filter, removing the fine, off-white precipitate of silver iodide. The transfer rate was dropped to 10 mL/min (from the more usual 40 mL/min) to overcome the increased resistance from pulling liquid through the filter. The reactor module was then washed with water (3 x 10 mL), with each of these washings transferred to the filter module via the Bottom-of-the-Bottle filter. The backbone was then rinsed with water (2 x 3 mL).

Once the reaction mixture was transferred to the filter module charged with solid potassium chloride, the resultant pale yellow solution was heated to 40 °C and stirred at 150 rpm for 15 min. The stirring was then paused and the module cooled to 4 °C for a further 15 min to induce crystallisation. The automated reaction paused at this point, and was manually resumed after an arbitrary time, to permit additional crystallisation time as required (often 16 h).

The mixture is then filtered and the residue washed with ethanol (10 mL) and diethyl ether (10 mL). This affords the desired product as yellow needles (99.8 mg, 0.33 mmol, 40%).

IR $\bar{\nu}/cm^{-1}$: 3275 (s), 3197 (s), 1619 (m), 1597 (sh), 1577 (sh), 1536 (s), 1314 (m), 1292 (s), 795(sh), 783 (s)

UV (water) λ_{max}/nm: 275, 298, 350

4 Focused Library in Process Space: {Mo_{132}} and {Mo_{154}}

Each of these targets were accessed via a 1-step synthesis, adapted from in-group procedures.[12] The only significant difference between the two procedures was the amount of hydrochloric acid required. Crystallisations were effected *via* evaporation and performed outwith the rig.

4.1 General Procedure

Setup: At least two reactor modules were required for this experiment. The first (r1) was left empty to premix liquid reagents, and the second (r#) loaded manually with sodium dithionite (0.10 g, 0.57 mmol, Eq: 0.6). Up to three experiments could be conducted in parallel by premixing three sets of reagents in r1 and decanting these to the remaining three reactor modules, each of which must be manually loaded with sodium thionite. Individual 250 mL reagent bottles were charged with (i) reagents: 0.32 M aqueous sodium molybdate dihydrate, 0.97 M aqueous sodium acetate, 50 v/v% aqueous acetic acid, 1 M hydrochloric acid, (ii) solvents: water, acetone, and (iii) cleaning solutions: 0.1 M $HCl_{(aq)}$ solution, 0.05 M $Na_4EDTA_{(aq)}$ solution, acetonitrile, and acetone. All other reagents were stored under ambient conditions in air, and introduced to the platform in large excess.

Automated Procedure: Tubing from a selection of inputs (aqueous sodium molydate dihydrate, aqueous sodium acetate, acetic acid, hydrochloric acid) to the backbone were primed by transferring 3 mL of each input to waste. Backbone was rinsed by transferring water (2 x 3 mL) from the relevant input bottle to waste containers at either end of the backbone.

In reactor module r1, aqueous sodium molybdate dihydrate (5.0 mL, 0.32 M, 1.60 mmol, Eq: 1.0), was combined with aqueous sodium acetate (10.0 mL, 0.97 M, 9.70 mmol, Eq: 6.1), acetic acid (5.0 mL, 50 v/v% in water, 8.70 M, 43.71 mmol, Eq: 27.3), hydrochloric acid (varies, 1.0 M, see Table S4), and flushed with air to ensure full transfer. The backbone was washed with water (2 x 3 mL). Reactor r1 was held at r.t. and stirred at 300 rpm for 30 secs to ensure the solution is fully mixed.

The entire volume of reaction mixture was then decanted from reactor r1 to reactor r# to combine with solid sodium dithionite, giving a colour change from colourless to brown (without additional acid), or blue (in the presence of additional acid). Air was used to ensure full transfer and the backbone washed with water (2 x 3 mL), before the reactor r# was held at r.t. stirred at 300 rpm for approx. 30 min to ensure full reaction.

After stirring, the reaction mixture was transferred to the sample wheel. The tubing to the sample wheelwais first flushed into a waste vial with water (3 mL), and advanced to the subsequent position. The contents of the reactor r# were then decanted in full to the second vial and air used to ensure full transfer. The sample wheel was advanced to the subsequent position, the backbone flushed with water (4 x 3 mL), and the tubing to the sample wheel also flushed with water (3 mL). Collected samples can be further analysed offline (see below).

***Table S4**: List of conditions probed in the synthesis of giant reduced molybdates.*

Equivalences of HCl	Volume 1M HCl (mL)	Total Volume (mL)
0	0	20.0
5	8.04	28.0
7	11.25	31.3
9	14.47	34.5
11	17.69	37.7
13	20.9	40.9

4.2 UV-vis Spectroscopy

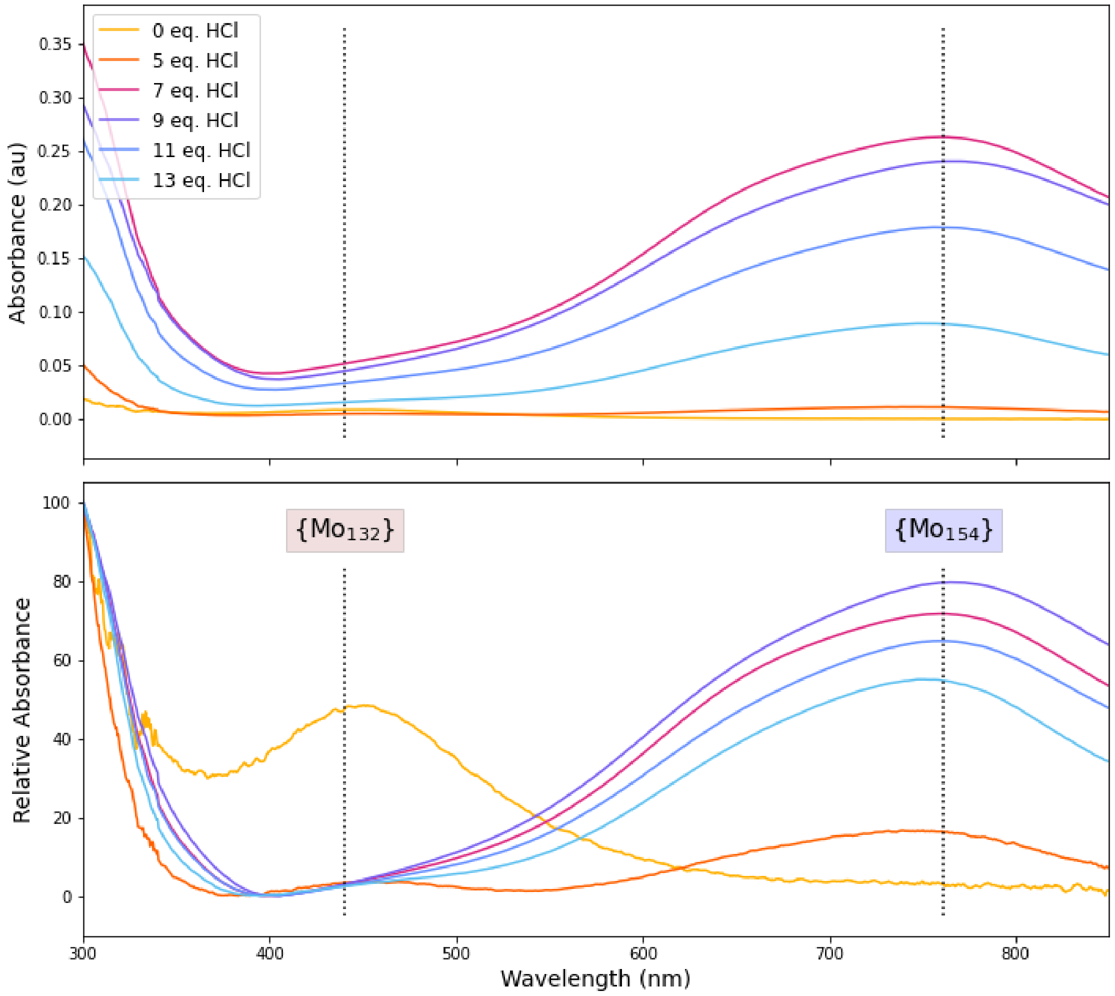

***Figure S30**: UV traces for the various conditions probed in the synthesis of giant reduced molybdates. Expected maximum wavelengths for the {Mo_{132}} Keplerate and {Mo_{154}} wheel are annotated.*

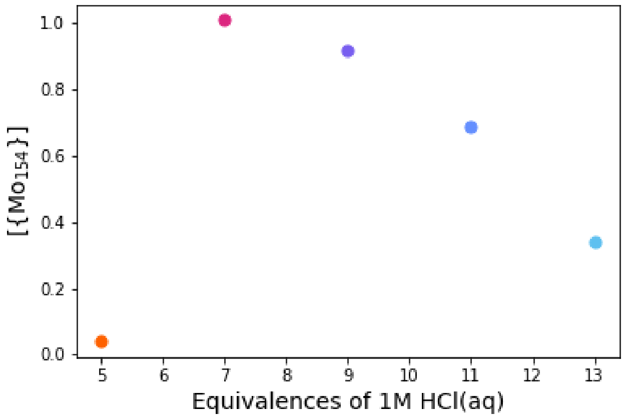

***Figure S31**: Calculated concentration of {Mo_{154}} with equivalences of 1M HCl(aq). Colour as above.*

5 Focused Library in Chemical Space: $Ru(bpy)_2Cl_2$

5.1 General Procedure

Manual Setup: Four reactor modules are required for this experiment, none require charging with solid reagents. The jacketed filter module is loaded manually with bis-(2,2'-bipyridine)dichlororuthenium(II) (1.0 g, 1.56 mmol, Eq: 15.0). Input bottles of differing size were used for this experiment: (i) 100 mL bottles are charged with 0.06 M ethanolic solutions of N-heterocycles: 2,6-lutidine, 2-aminopyridine, 2-picolinic acid, isoquinoline, and piperazine, (ii) a 500 mL bottle charged with ethanol, and (iii) 250 mL bottles charged with cleaning solvents: 0.1 M $HCl_{(aq)}$ solution, 0.05 M $Na_4EDTA_{(aq)}$ solution, acetonitrile, acetone, and water.

Automated Setup: Tubing from a selection of inputs (solutions of 2,6-lutidine, 2-aminopyridine, 2-picolinic acid, isoquinoline, piperazine, 4,4'-bipyridine) to the backbone were primed by transferring 3 mL of each input to waste. Backbone was rinsed by transferring ethanol (2 x 3 mL) from the relevant input bottle to waste containers at either end of the backbone.

The filter was primed with ethanol (10 mL) and an additional aliquot of ethanol (60 mL) added from the top of the filter module to dissolve the bis-(2,2'-bipyridine)dichlororuthenium(II). The suspension was held at 75 °C for 5 min with stirring at 150 rpm to dissolve the ruthenium complex. The tubing from the bottom of the filter to the backbone was then flushed with the ruthenium complex solution by moving 10 mL of the filter volume to waste.

Reaction: Stirring of the ruthenium complex solution was maintained at 150 rpm throughout the preparation of the reactions. Ruthenium complex solution (4 mL, 0.026 M, 0.10 mmol, Eq: 1.0) was transferred to each of the four reactors, and flushed through with air. The backbone was then washed with ethanol (6 x 2 mL), N-heterocyclic components (or ethanol) (4 mL of each, 0.06 M, 0.24 mmol, Eq: 2.4) added to each reactor, and the tubing flushed through with air. Components for each reaction were read in to the executing python script from a .csv file. The backbone was rinsed with further ethanol (4 x 2 mL), and the reactors heated to 85 °C for 16 h with stirring at 300 rpm.

Sampling: After heating had completed, the contents of each reactor were transferred to the sample wheel and flushed through to the collection vial with ethanol (3 mL) and air. The wheel is rotated in preparation to receive the next sample and the backbone washed with ethanol (2 x 3 mL) before the subsequent reactor is sampled. At this point, the reaction mixtures had changed from dark purple-red ruthenium complex solution to orange or orange-red solutions of product.

Cleaning: Following sampling, the reactors are cleaned by moving ethanol (10 mL) to each reactor, heating to 85 °C for 10 min with stirring at 300 rpm, and then emptying the reactors to waste. The reactors are flushed two further times with ethanol (10 mL) without heating.

This 'Reaction-Sample-Clean' process can be run repeatedly until the full library of conditions has been executed.

5.2 Results from Reactions with a Single N-Heterocycle

5.2.1 Overview of Characterisation Data

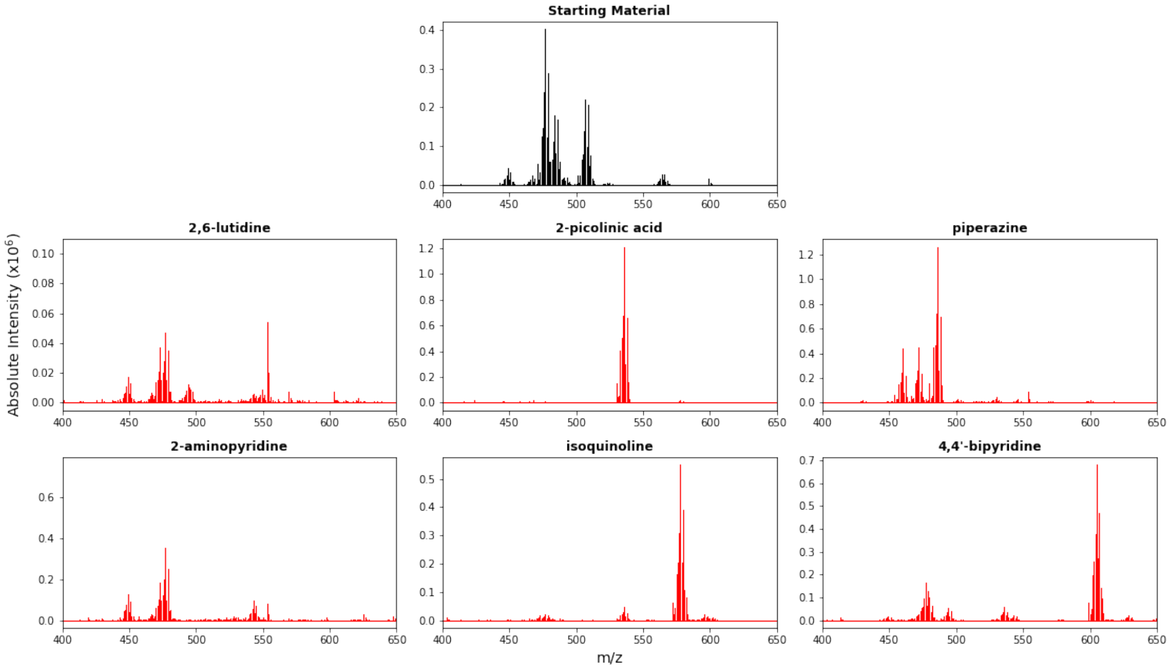

Figure S32: *MS data from samples with a single N-heterocyclic ligand*

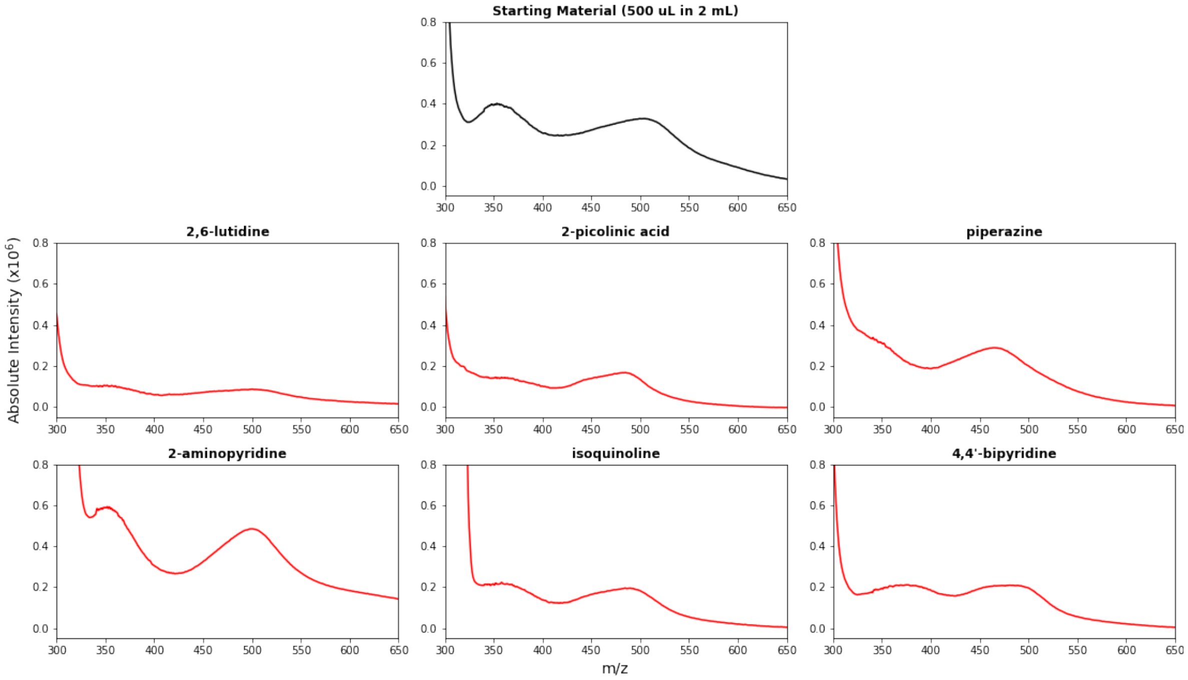

Figure S33: *UV data from samples with a single N-heterocyclic ligand*

5.2.2 Starting Material, $[Ru(bipy)_2Cl_2]$

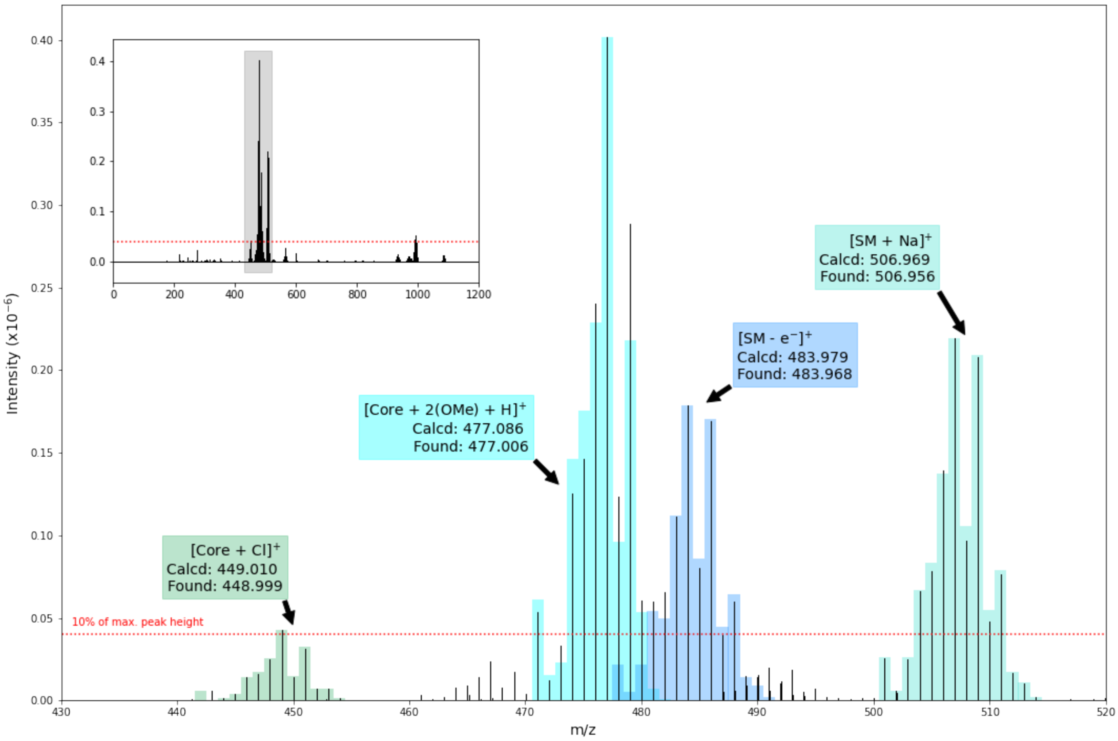

Figure S34: *Assigned MS data. Calculated isotope patterns are show with coloured bars. Assignment values for each identified species are detailed in the annotations on the main plot. The full plot is shown as inset with the area corresponding to the main plot highlighted in grey. 'Core' refers to the fragment* $[Ru(bpy)_2]^{2+}$*, SM to the species* $[Ru(bpy)_2Cl_2]$.

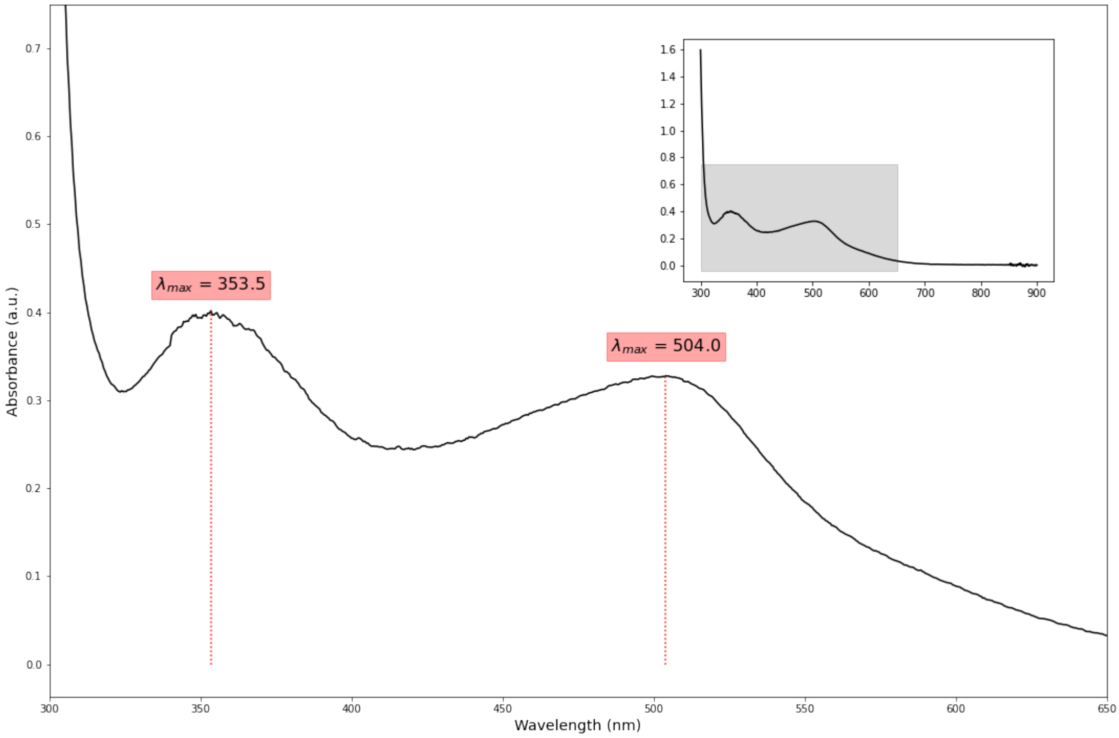

Figure S35: *Assigned UV data.* λ_{max} *values are annotated on the main plot. The full plot is shown as inset, with the area corresponding to the main plot highlighted in grey.*

5.2.3 2,6-Lutidine

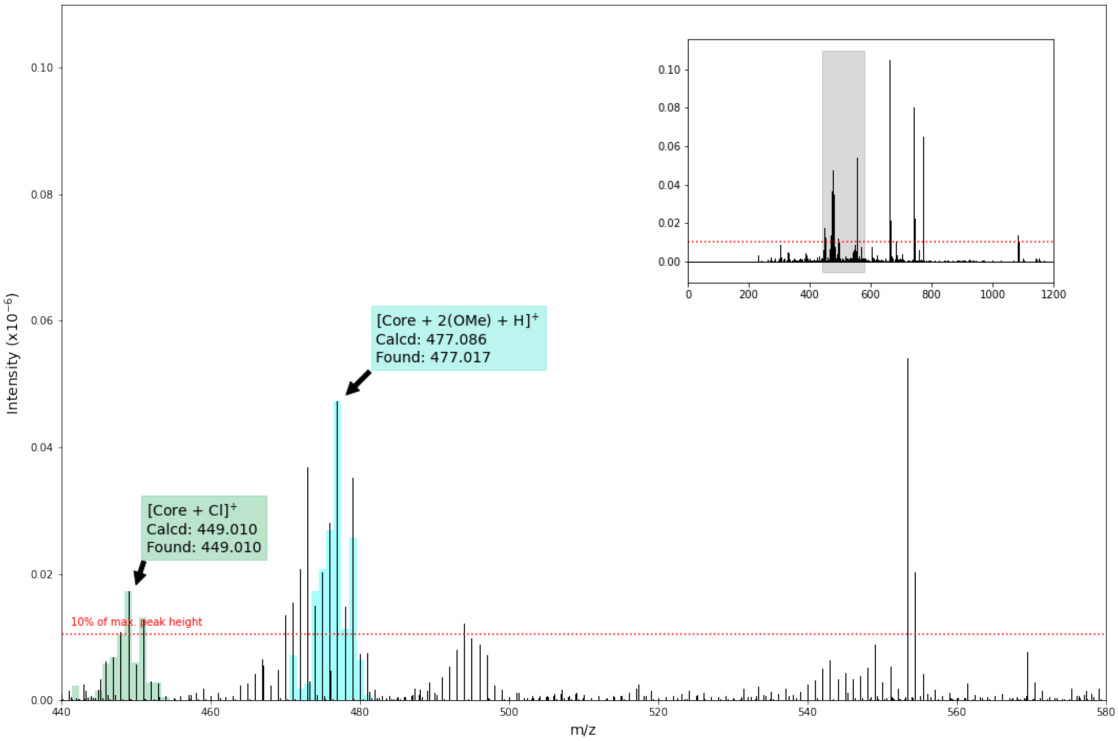

Figure S36: *Assigned MS data. Calculated isotope patterns are show with coloured bars. Assignment values for each identified species are detailed in the annotations on the main plot. The full plot is shown as inset with the area corresponding to the main plot highlighted in grey. 'Core' refers to the fragment $[Ru(bpy)_2]^{2+}$, SM to the species $[Ru(bpy)_2Cl_2]$.*

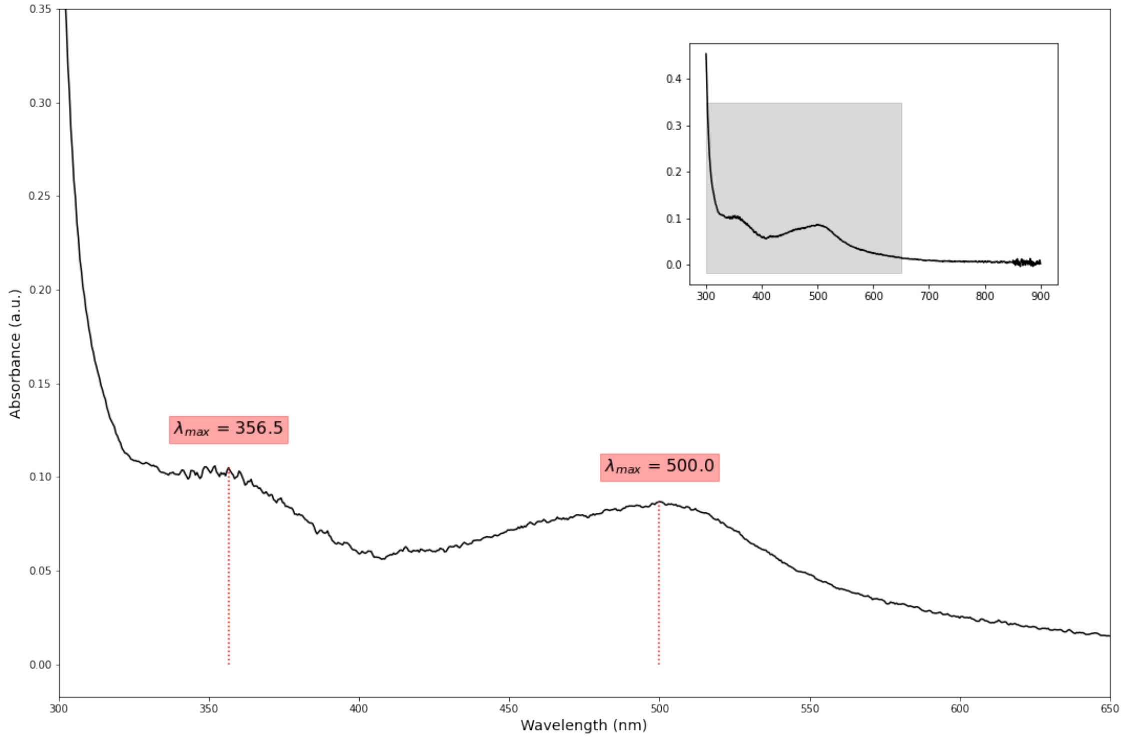

Figure S37: *Assigned UV data. λ_{max} values are annotated on the main plot. The full plot is shown as inset, with the area corresponding to the main plot highlighted in grey.*

5.2.4 2-Picolinic Acid

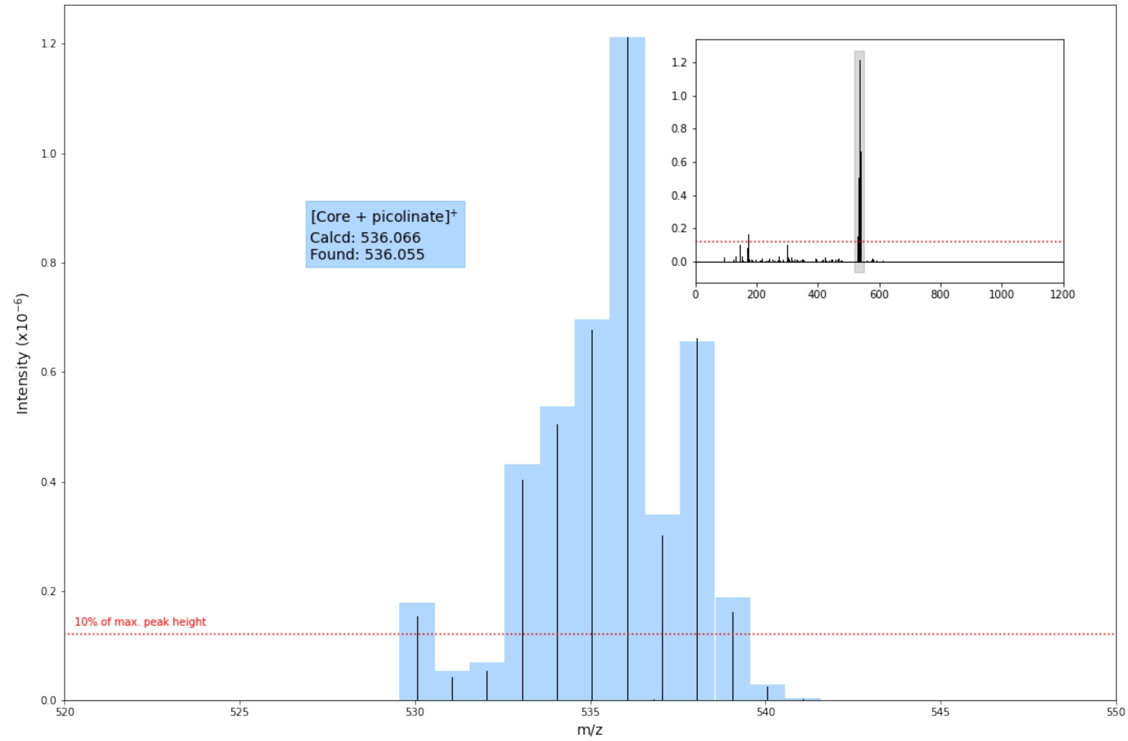

Figure S38: *Assigned MS data. Calculated isotope patterns are show with coloured bars. Assignment values for each identified species are detailed in the annotations on the main plot. The full plot is shown as inset with the area corresponding to the main plot highlighted in grey. 'Core' refers to the fragment* $[Ru(bpy)_2]^{2+}$*, SM to the species* $[Ru(bpy)_2Cl_2]$*.*

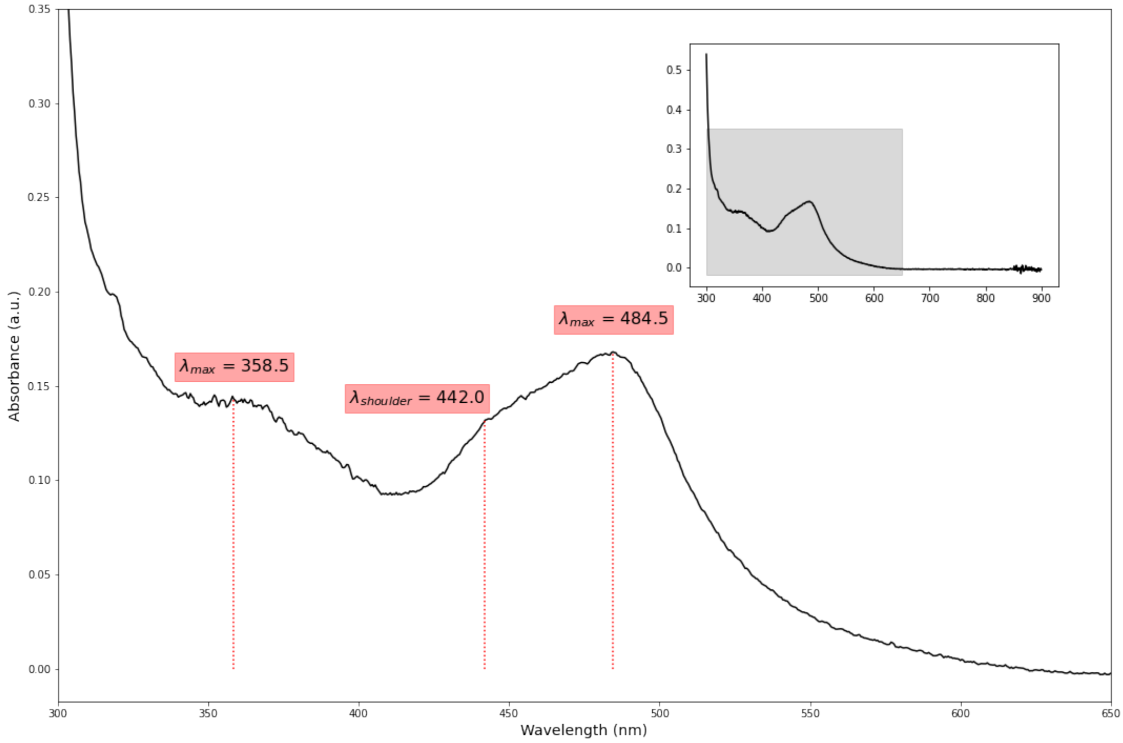

Figure S39: *Assigned UV data.* λ_{max} *values are annotated on the main plot. The full plot is shown as inset, with the area corresponding to the main plot highlighted in grey.*

5.2.5 Piperazine

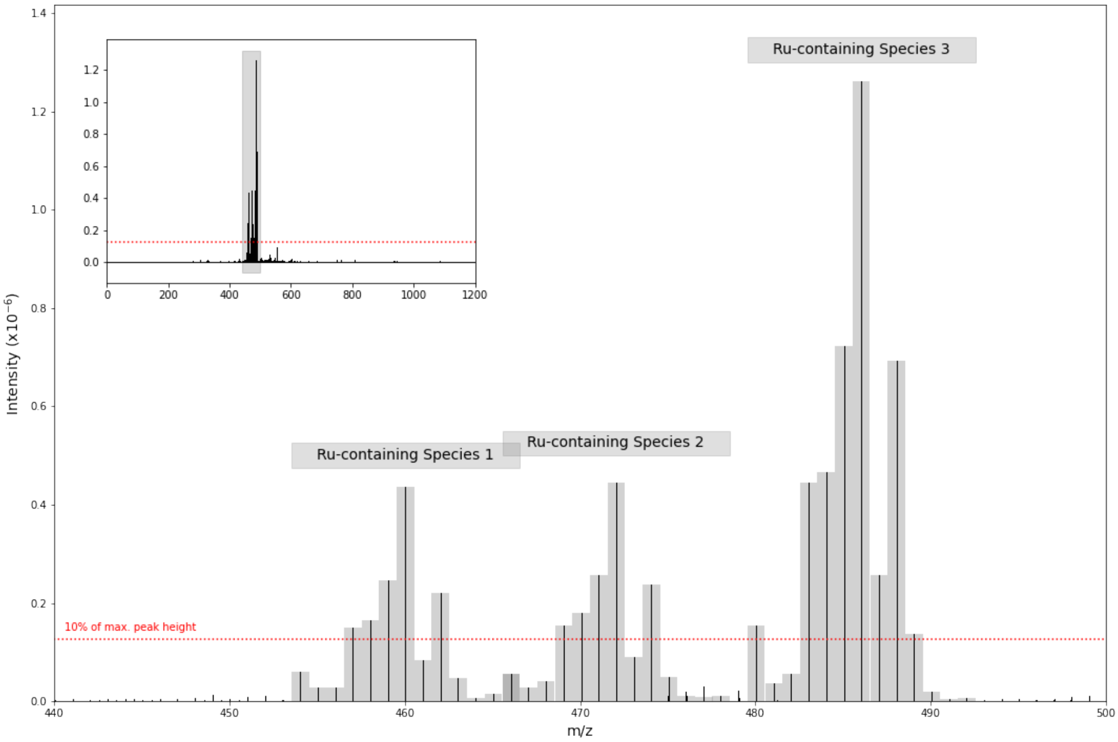

Figure S40: *MS data. Assignment proved problematic, so peaks with the splitting pattern of ruthenium are highlighted, but not formally assigned. The full plot is shown as inset with the area corresponding to the main plot highlighted in grey.*

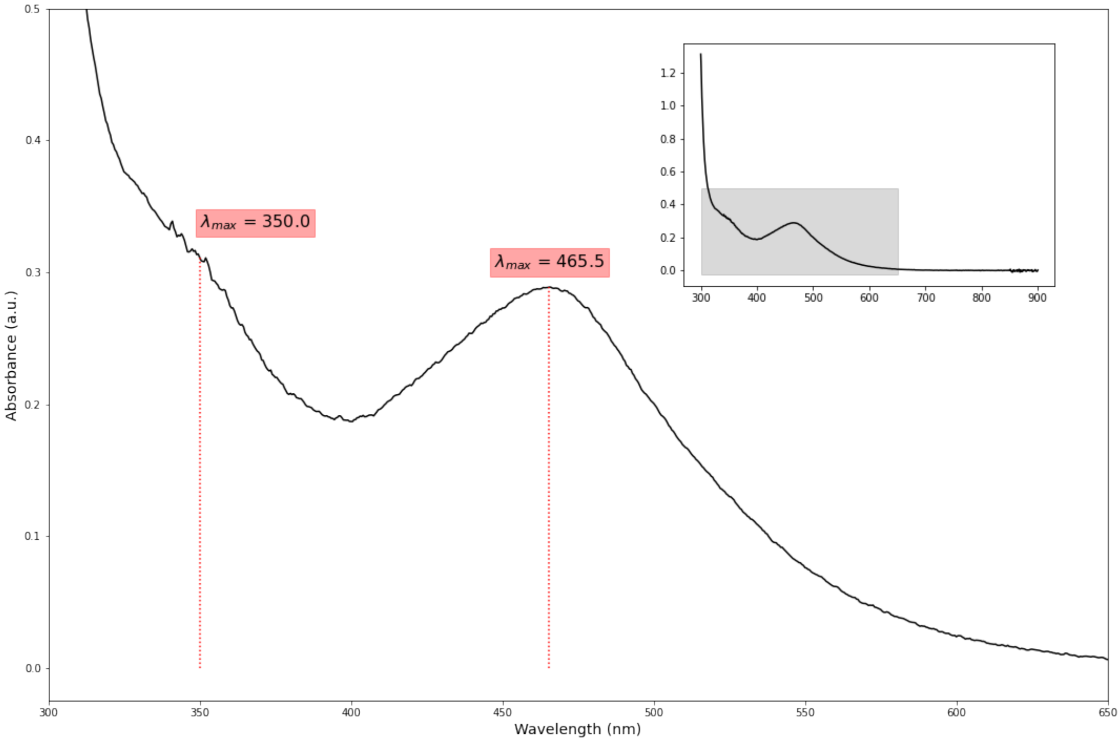

Figure S41: *Assigned UV data.* λ_{max} *values are annotated on the main plot. The full plot is shown as inset, with the area corresponding to the main plot highlighted in grey.*

5.2.6 2-Aminopyridine

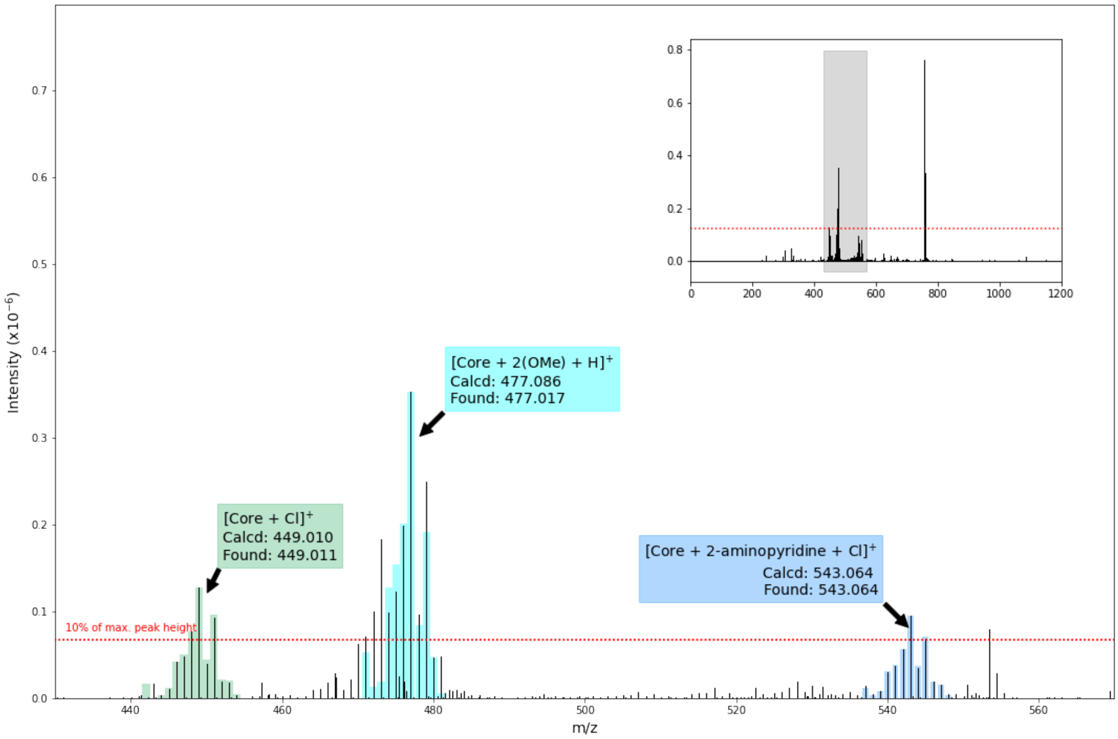

Figure S42: *Assigned MS data. Calculated isotope patterns are show with coloured bars. Assignment values for each identified species are detailed in the annotations on the main plot. The full plot is shown as inset with the area corresponding to the main plot highlighted in grey. 'Core' refers to the fragment [Ru(bpy)$_2$]$^{2+}$, SM to the species [Ru(bpy)$_2$Cl$_2$].*

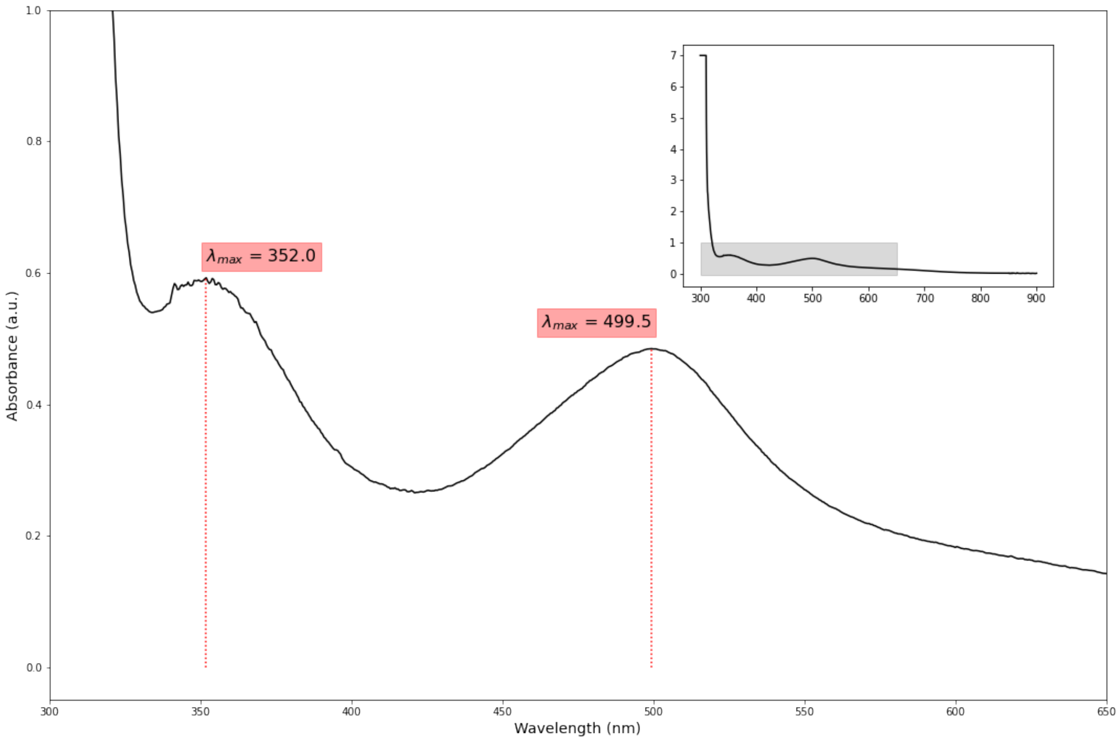

Figure S43: *Assigned UV data. λ_{max} values are annotated on the main plot. The full plot is shown as inset, with the area corresponding to the main plot highlighted in grey.*

5.2.7 Isoquinoline

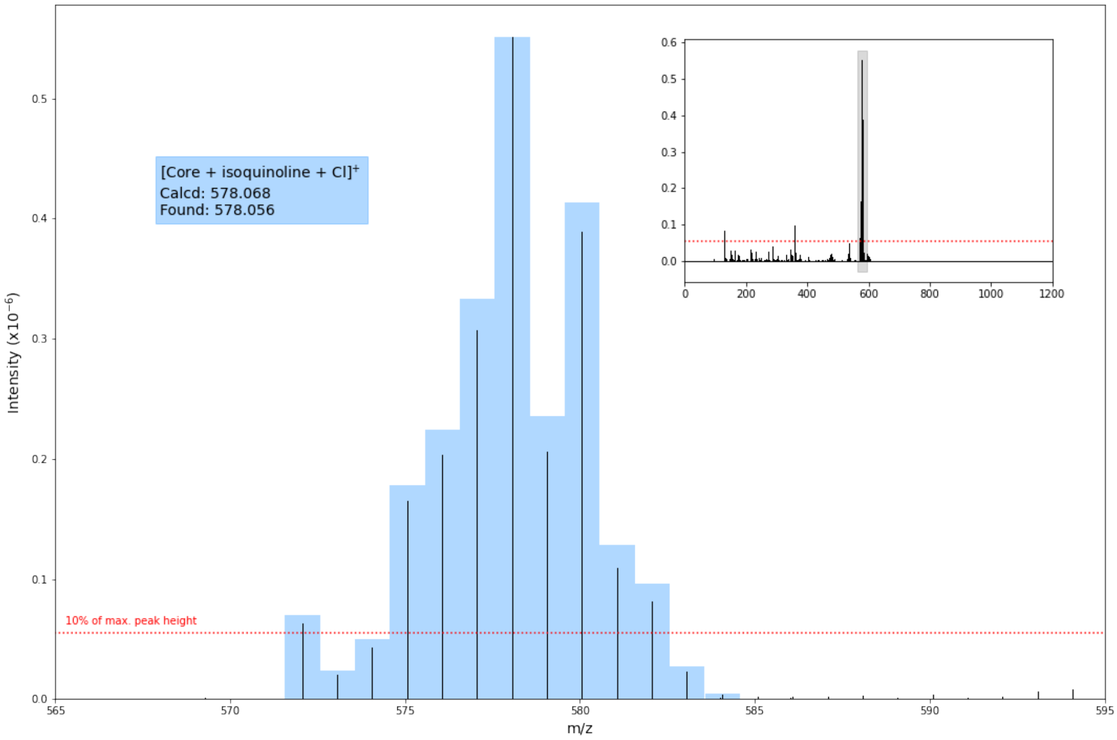

Figure S44: *Assigned MS data. Calculated isotope patterns are show with coloured bars. Assignment values for each identified species are detailed in the annotations on the main plot. The full plot is shown as inset with the area corresponding to the main plot highlighted in grey. 'Core' refers to the fragment* $[Ru(bpy)_2]^{2+}$, *SM to the species* $[Ru(bpy)_2Cl_2]$.

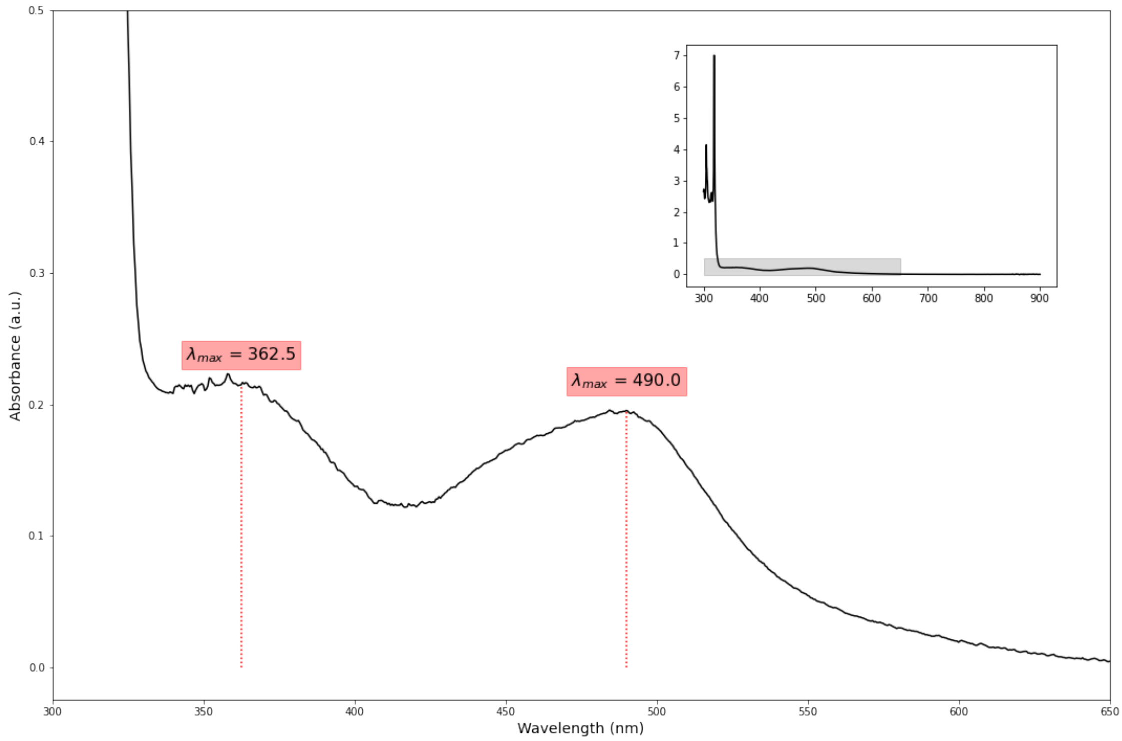

Figure S45: *Assigned UV data.* λ_{max} *values are annotated on the main plot. The full plot is shown as inset, with the area corresponding to the main plot highlighted in grey.*

5.2.8 4,4'-Bipyridine

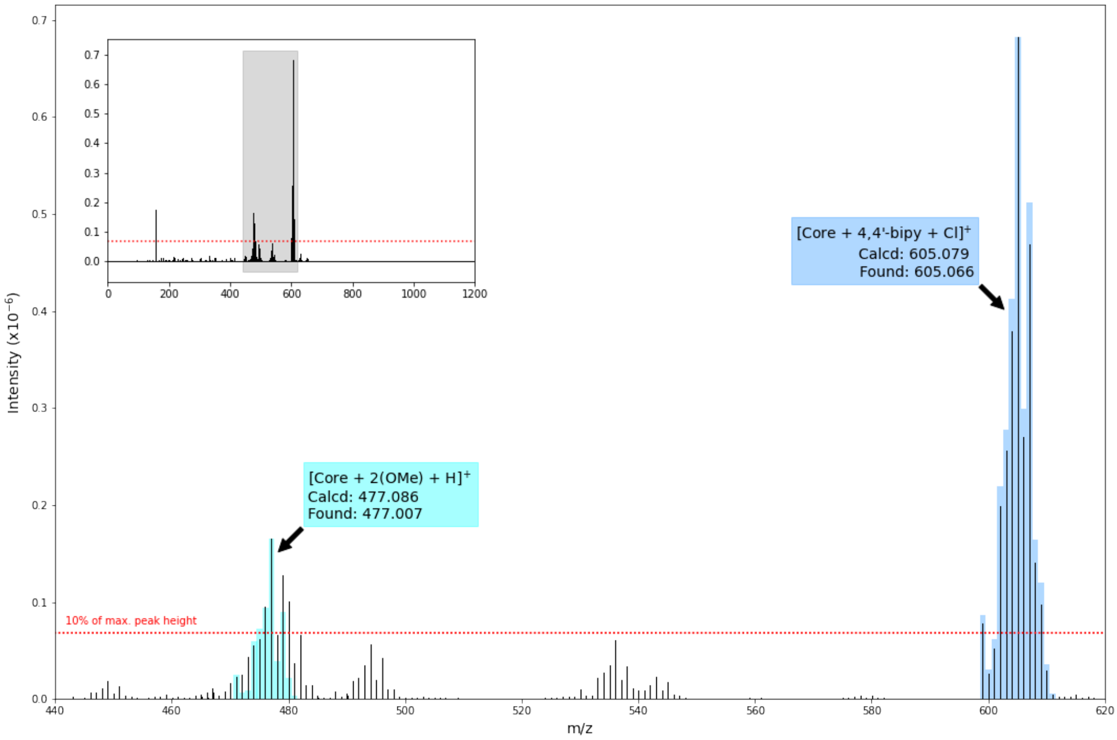

Figure S46: *Assigned MS data. Calculated isotope patterns are show with coloured bars. Assignment values for each identified species are detailed in the annotations on the main plot. The full plot is shown as inset with the area corresponding to the main plot highlighted in grey. 'Core' refers to the fragment [Ru(bpy)$_2$]$^{2+}$, SM to the species [Ru(bpy)$_2$Cl$_2$].*

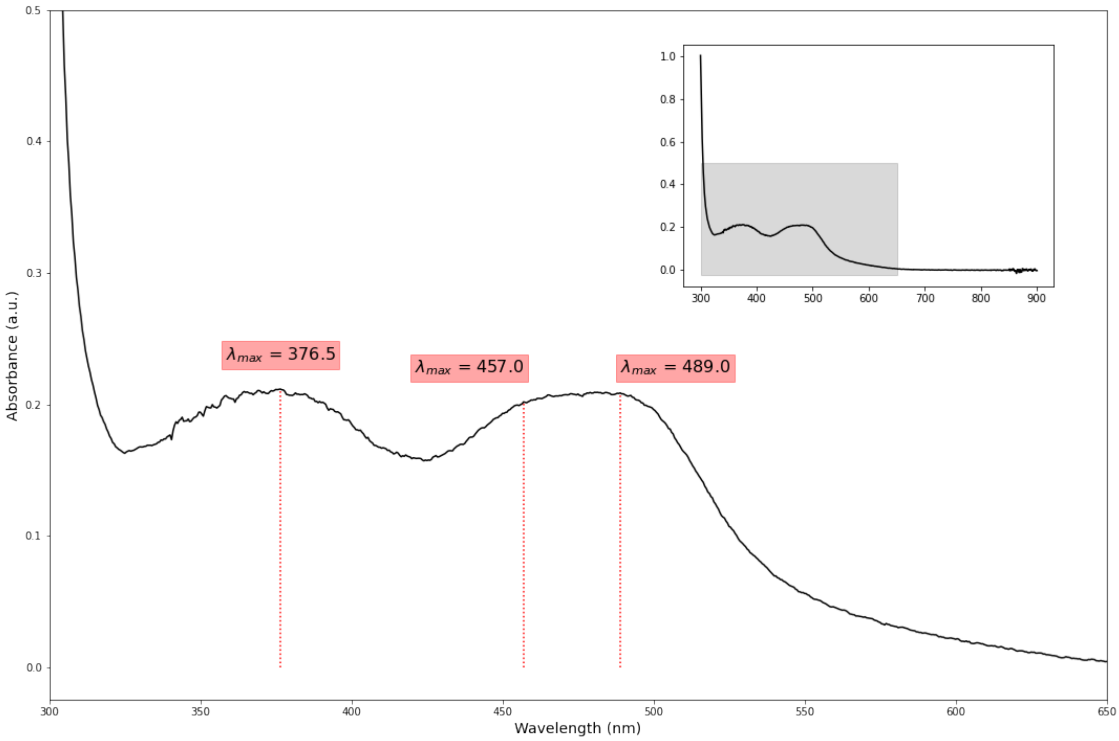

Figure S47: *Assigned UV data. λ_{max} values are annotated on the main plot. The full plot is shown as inset, with the area corresponding to the main plot highlighted in grey.*

5.3 Results from Pairwise Reactions

5.3.1 MS Data

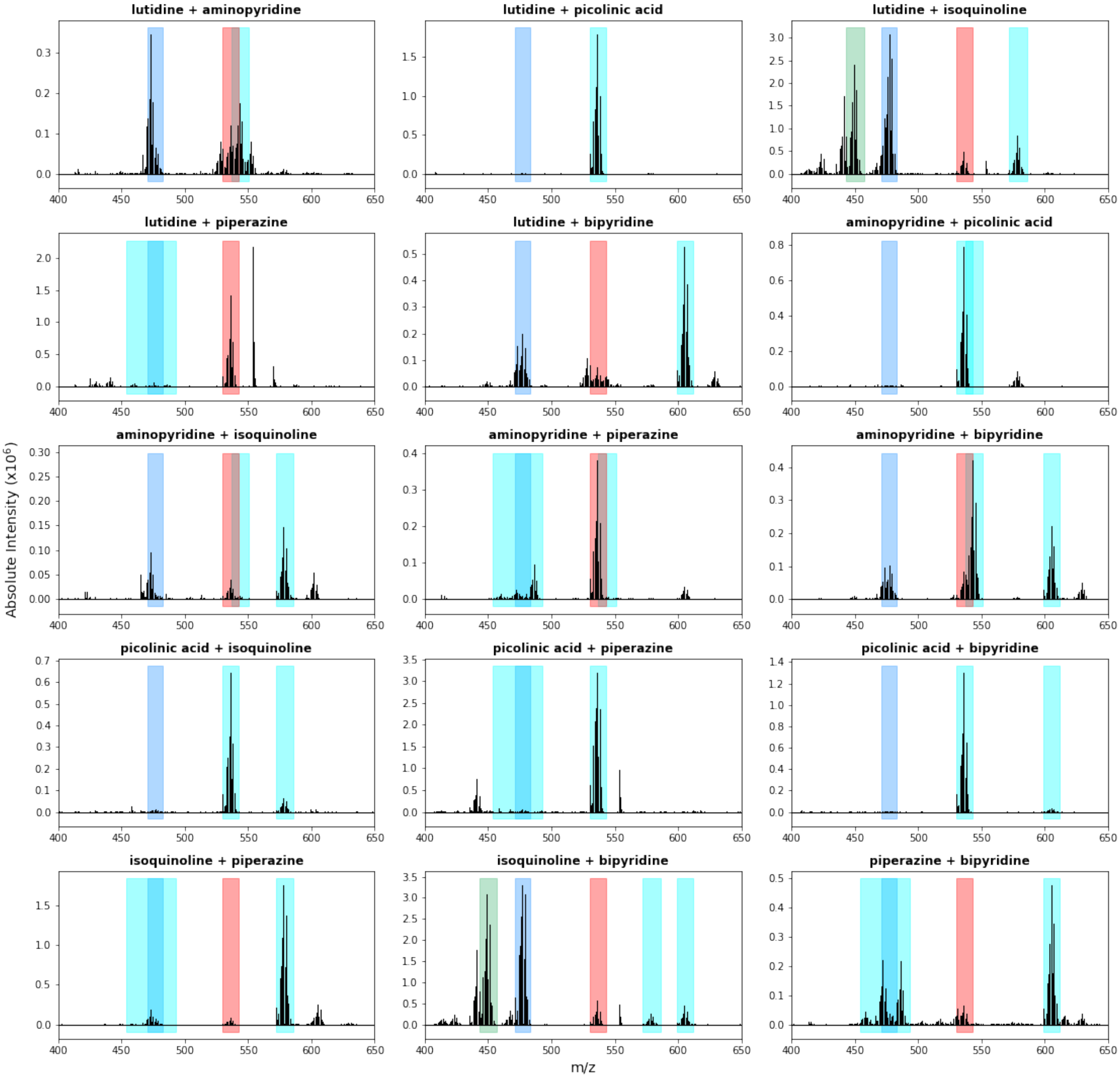

***Figure S48**: MS data from samples with multiple N-heterocyclic ligands. Ligands corresponding to each spectrum are detailed above the spectrum. Highlights as follows: expected peaks for monoadduct species, based on the above data (cyan); $[Ru(bpy)_2(OMe)_2{+}H]^+$ species (darker blue); $[Ru(bpy)_2Cl]^+$ species (green); and undesired presence of 2-picolinic acid impurities (red).*

5.3.2 UV Data

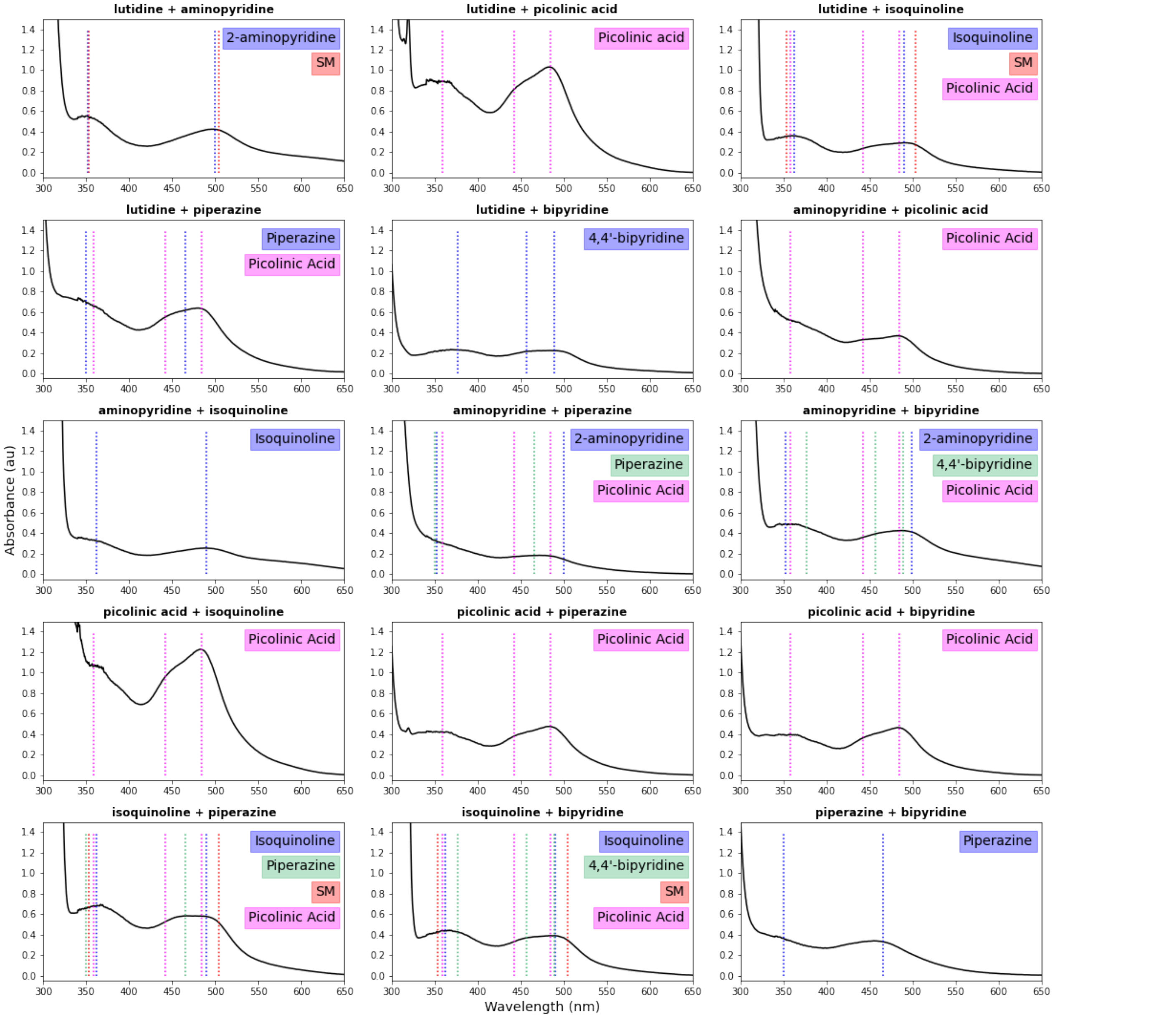

***Figure S49**: UV data from samples with multiple N-heterocyclic ligands. Ligands corresponding to each spectrum are detailed above the spectrum. Comparisons with λ_{max} values from relevant single N-Heterocyclic systems or SM are highlighted for each composition.*

5.3.3 Summary

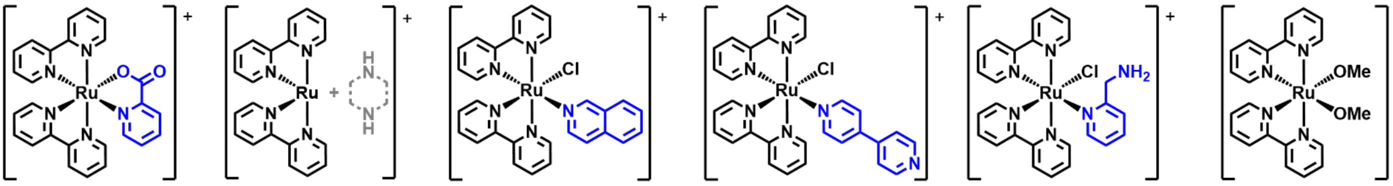

***Figure 50**: Products as assigned based on MS and UV data. Structure of piperidine-derived species is not trivial. Donor atom in 2-aminopyridine complex assigned on literature-observed behaviour Ru(II), Pt(II), and Zn(II) complexes.*[13-15]

***Table S5**: Conclusions from pairwise combination of N-heterocyclic ligands with the $Ru(bpy)_2Cl_2$ precursor. Species which appear to be present in either MS or UV data are indicated by number (see Fig. S50, above). Where the assignment of a given species doesn't appear conclusive, parantheses '()' are used to enclose the assignment.*

#	Components	Observed Species<br>*From MS*	*From UV*	Major Binder
1	Lutidine<br>2-aminopyridine	E, (F)	E, (F)	2-aminopyridine
2	Lutidine<br>Picolinic acid	A	A	Picolinic acid
3	Lutidine<br>Isoquinoline	C, F, A	C, (F), (A)	Isoquinoline
4	Lutidine<br>Piperazine	A	A	*Unclear, only contaminant visible*
5	Lutidine<br>4,4'-bipyridine	D, F, A	D	4,4'-bipyridine
6	2-aminopyridine<br>Picolinic acid	A	A	Picolinic acid
7	2-aminopyridine<br>Isoquinoline	C, F, A	C	Isoquinoline
8	2-aminopyridine<br>Piperazine	A, (B)	(A)	(Piperazine)
9	2-aminopyridine<br>4,4'-bipyridine	D, E, F, A	(A)	*Unclear, contaminant visible*
10	Picolinic acid<br>Isoquinoline	C, A	A	Picolinic acid
11	Picolinic acid<br>Piperazine	A	A	Picolinic acid
12	Picolinic acid<br>4,4'-bipyridine	A	A	Picolinic acid
13	Isoquinoline<br>Piperazine	C, F, A	C, (B)	*Similar strength*
14	Isoqinoline<br>4,4'-bipyridine	C, D, F, A	C, D	*Similar strength*
15	PIperazine<br>4,4'-bipyridine	B, D, A	B	Piperazine

6 Automated Exploration

6.1 Preparation of Starting Materials

6.1.1 $[Cr_3(\mu_3\text{-}O)(OAc)_6(H_2O)_3](OAc).xH_2O$

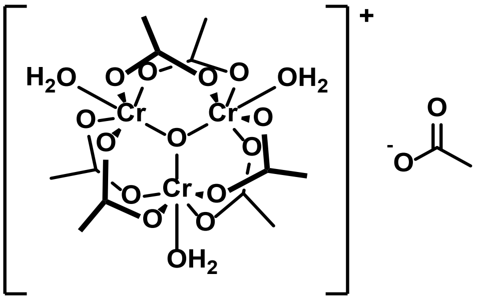

Adapted from literature procedure.[16]

Chromium(III) nitrate nonahydrate (20.0 g, 0.05 mol, Eq: 1.0), was dissolved in a minimum volume of water. Separately, sodium hydroxide (6.0 g, 0.15 mol, Eq: 3.0) was dissolved in water (50 mL), and the resulting solution was added dropwise to the metal solution with continuous stirring. The precipitate of chromium(III) hydroxide was washed with water and dissolved in glacial acetic acid (7.1 mL, 0.125 mol, Eq: 2.5), and the solution refluxed for 2 h. The resulting mixture was cooled in the fridge overnight, and then concentrated to dryness *in vacuo* (requiring 5 h on a rotary evaporator set to 35 mbar, and with water bath at 61.5 °C) forming fibrous green crystals of $[Cr_3O(OAc)_6(H_2O)_3](OAc)\cdot xH_2O$ (9.2 g, 78%). Used directly in the subsequent step.

FT-IR (wavenumber, cm^{-1}): 3348 (w), 2996 (w), 1614 (s), 1531 (s), 1445 (s), 1416 (s), 1348 (m), 1031 (m), 956 (w), 828 (w), 642 (s), 620 (s)

6.1.2 $[Cr_3(\mu_3\text{-}O)(OAc)_6(pyr)_3]ClO_4$, $\{Cr_3O\}$

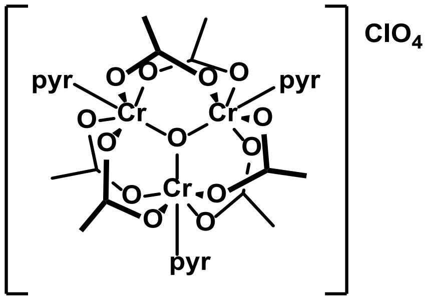

Adapted from literature procedure.[16]

$[Cr_3O(OAc)_6(H_2O)_3](OAc).xH_2O$ (9.0 g, 12.6 mmol, Eq: 1.0) was dissolved in aqueous perchloric acid (90 mL, 0.5 M, 45.0 mmol, Eq: 3.6) and pyridine (9.2 mL, 120.0 mmol, Eq: 9.5) was added. The resulting green solution was heated gently for 20 min, and the greenish-grey precipitate which formed rapidly on cooling was collected by filtration, washed with ethanol and diethyl ether, and dried in vacuo (6.6 g, 60%).

MS (ESI) m/z: $[M]^+$ Calcd: 763.0229, Found: 763.0302; $[M\text{-}pyr+H_2O]^+$ Calcd: 701.9912, Found: 701.9978; $[M\text{-}pyr]^+$ Calcd: 683.9807, Found: 683.9936; $[M\text{-}2(pyr)+2(H_2O)]^+$ Calcd: 640.9596, Found: 640.9676; $[M\text{-}2(pyr)+H_2O]^+$ Calcd: 622.9490, Found: 622.9556; $[M\text{-}2(pyr)]^+$ Calcd: 604.9385, Found: 604.9542; $[M\text{-}3(pyr)+H_2O]^+$ Calcd: 543.9068, Found: 543.9132; $[M\text{-}3(pyr)]^+$ Calcd: 525.8963, Found: 525.9033

FT-IR (wavenumber, cm^{-1}): 3488 (w), 3084 (w), 1607 (vs), 1490 (w), 1448 (vs), 1427 (sh), 1350 (m), 1046 (ob), 1018 (sh), 958 (w), 767 (w), 674 (s), 645 (s), 620 (s)

6.1.3 $[Mn_3(\mu_3\text{-}O)(OAc)_6(pyr)_3]ClO_4$, $\{Mn_3O\}$

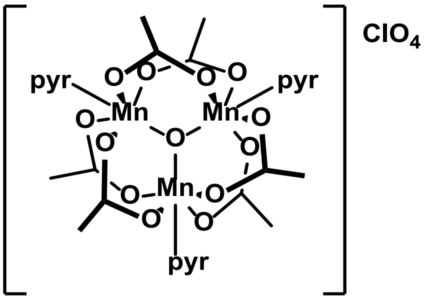

Adapted from literature procedure.[9] See above for automated version.

Manganese(II) acetate tetrahydrate (20.0 g, 81.5 mmol, Eq: 1.0) was dissolved in a solvent mixture comprising absolute ethanol (200 mL), glacial acetic acid (120 mL), and pyridine (30 mL). The resulting solution was stirred while solid tetra-*n*-butylammonium permanganate (11.4 g, 31.5 mmol, Eq: 0.4, synthesised as above) was added in small portions to give a deep brown homogeneous solution. Sodium perchlorate (6.9 g, 56.5 mmol, Eq: 0.7) was then added, and the resultant solution was left to stir at r.t. for a further 3 h. The mixture was transferred to the fridge for 4 h, yielding a brown microcrystalline precipitate which was collected by filtration, washed copiously with ethanol, and dried *in vacuo*. Product was afforded as brown microcrystals (21.4 g, 65%).

MS (MALDI) m/z: $[M\text{-}OAc\text{-}3(pyr)](ClO_4)^+$ Calcd: 574.82, Found: 575.05

FT-IR (wavenumber, cm^{-1}): 3114 (w), 1713 (w), 1599 (vs), 1489 (w), 1340 (s), 1220 (s), 1160 (w), 1086 (vs, ClO4-), 1067 (vs), 1044 (vs), 1017 (s), 943 (w), 768 (s), 694 (s), 664 (s), 647 (s), 610 (s), 585 (s)

6.1.4 [$Fe_3(\mu_3\text{-}O)(OAc)_6(H_2O)_3$]$NO_3.xH_2O$

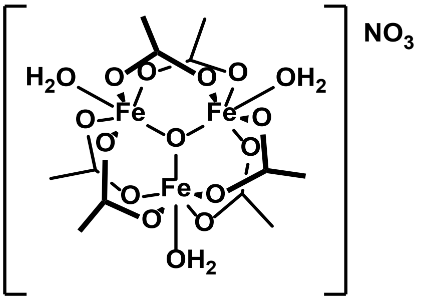

Adapted from literature procedure.[16]

Anhydrous sodium acetate (12.1 g, 147 mmol, Eq: 1.0) was suspended in water (50 mL). The solution was warmed to 50 °C for 105 mins until complete dissolution, and solid iron(III) nitrate nonahydrate (29.7 g, 74 mmol, Eq: 0.5) was added in portions to yield a red solution. The mixture was then filtered and left to crystallise at 4 °C. After one week, the solution yielded desired product as large, red crystals (3.9 g, 24%). Used directly in the subsequent step.

FT-IR (wavenumber, cm^{-1}): 3189 (m), 1686 (w), 1581 (s), 1412(s), 1346 (s), 1289 (s), 1032 (m), 950 (w), 833 (w), 658 (m), 600 (s)

6.1.5 $[Fe_3(\mu_3\text{-}O)(OAc)_6(pyr)_3]NO_3$, $\{Fe_3O\}$

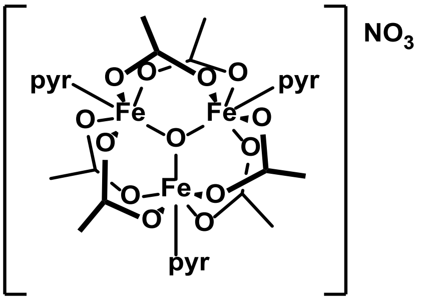

Adapted from literature procedure.[16]

$[Fe_3O(OAc)_6(H_2O)_3]NO_3.xH_2O$ (7.00 g, 9.8 mmol, Eq: 1.0) was dissolved in ethanol (70 mL) and pyridine (3.6 mL). The solution was heated to 50 °C, held at temperature for 2 min, and left to cool to r.t., yielding a yellow-green precipitate. Precipitate was collected by filtration, washed with ethanol and diethyl ether, and dried *in vacuo* to afford desired product (7.7 g, 94%).

FT-IR (wavenumber, cm^{-1}): 3030 (w), 1587 (vs), 1489 (w), 1444 (vs), 1332 (vs), 1041 (vs), 1030 (w), 953 (m), 770 (w), 696 (m), 657 (s), 592 (vs).

6.1.6 $[Co_3(\mu_3\text{-}O)(\mu_2\text{-}OH)(OAc)_5(pyr)_3]PF_6$, $\{Co_3O(OH)\}$

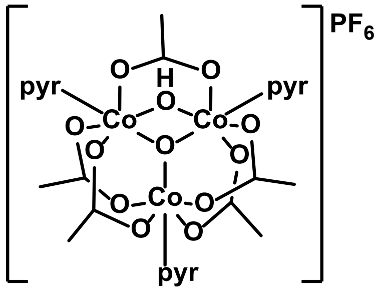

Adapted from literature procedure.[17]

Cobalt(II) acetate (25.0 g, 100 mmol, Eq: 1.0) was dissolved in acetic acid (50 mL) and pyridine (8.9 mL). The resulting mixture was warmed to ~50 °C until all cobalt had dissolved to give a purple solution, and 39% peracetic acid (7.6 g, 100 mol, Eq: 1.0) was added dropwise while the solution was vigorously stirred. The dark brown solution was combined with water (60 mL) and the resultant mixture heated to 82 °C for 1 h, before cooling to 70 °C. A solution of ammonium hexafluorophosphate (11.0 g, 100 mmol, Eq: 1.0) in water (35 mL) was added as one portion, and the resulting mixture was left to cool to r.t. The product was collected by filtration and vacuum dried to yield red-black microcrystals (26.7 g, 90%).

MS (ESI) m/z: $[M]^+$ Calcd: 741.9903, Found: 742.0020; $[M\text{-}pyr+H_2O]^+$ Calcd: 680.9587, Found: 680.9683; $[M\text{-}pyr]^+$ Calcd: 662.9481, Found: 662.9597; $[M\text{-}2(pyr)]^+$ Calcd: 583.9060, Found: 583.9110

FT-IR (wavenumber, cm^{-1}): 3548 (w), 1587 (s), 1572 (m), 1487 (w), 1396 (s), 1354 (w), 1218 (m), 1071 (w), 1047 (w), 833 (s, PF6-), 756 (w), 688 (s), 631 (m), 556 (s)

^{1}H NMR

(600 MHz, CD_2Cl_2, δ/ppm): 9.19 (dt, *J* = 5.1, 1.5 Hz, 2H, pyr-H_{ortho}), 8.74 (dt, *J* = 5.4, 1.5 Hz, 4H, pyr-H_{ortho}), 8.25 (tt, *J* = 7.6, 1.5 Hz, 1H, pyr-H_{para}), 8.25 (tt, *J* = 7.6, 1.5 Hz, 2H, pyr-H_{para}), 7.89 (ddd, *J* = 7.5, 5.4, 1.3 Hz, 2H, pyr-H_{meta}), 7.72 (ddd, *J* = 7.5, 5.4, 1.3 Hz, 2H, pyr-H_{meta}), 2.17 (s, 3H, $-CH_3$), 2.09 (s, 6H, $-CH_3$), 2.01 (s, 6H, $-CH_3$)

(600 MHz, CD_3CN, δ/ppm): 9.26 (d, *J* = 5.1 Hz, 2H, pyr-H_{ortho}), 8.83 (d, *J* = 5.1 Hz, 4H, pyr-H_{ortho}), 8.31 (t, *J* = 7.6 Hz, 1H, pyr-H_{para}), 8.18 (t, *J* = 7.6 Hz, 2H, pyr-H_{para}), 7.92 (t, *J* = 7.0 Hz, 2H, pyr-H_{meta}), 7.76 (t, *J* = 7.0 Hz, 2H, pyr-H_{meta}), 2.08 (s, 3H, $-CH_3$), 2.06 (s, 6H, $-CH_3$), 1.95 (s, 6H, $-CH_3$)

(600 MHz, CD_3OD, δ/ppm): 9.26 (dt, *J* = 5.1, 1.3 Hz, 2H, pyr-H_{ortho}), 9.00 (dt, *J* = 5.1, 1.3 Hz, 4H, pyr-H_{ortho}), 8.30 (tt, *J* = 7.6, 1.4 Hz, 1H, pyr-H_{para}), 8.17 (tt, *J* = 7.6, 1.4 Hz, 2H, pyr-H_{para}), 7.93 (ddd, *J* = 7.4, 5.6, 1.3 Hz, 2H, pyr-H_{meta}), 7.78 (ddd, *J* = 7.5, 5.3, 1.3 Hz, 2H, pyr-H_{meta}), 2.09 (s, 3H, $-CH_3$), 2.08 (s, 6H, $-CH_3$), 2.00 (s, 6H, $-CH_3$) Note that complex begins to degrade in deuterated methanol over a matter of days.

6.1.7 $[Co_3(\mu_3\text{-}O)(OAc)_6(pyr)_3]PF_6$, $\{Co_3O\}$

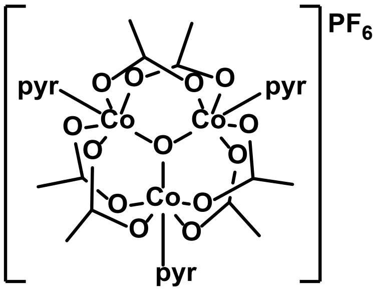

Adapted from literature procedure.[17]

$[Co_3O(OH)(OAc)_5(pyr)_3]PF_6$ (15.0 g, 17 mmol, Eq: 1.0) was suspended in acetic acid (54 mL) and acetic anhydride (6 mL) and heated to reflux for 5 min (from first observation of effervescence). Reaction mixture was left to cool to r.t., then filtered and washed with acetic acid (~30 mL) to afford a red-black microcrystalline powder (11.4 g, 72%).

MS (ESI) m/z: $[M]^+$ Calcd: 784.0009, Found: 784.0102; $[M\text{-}pyr]^+$ Calcd: 704.9587, Found: 704.9744; $[M\text{-}2(pyr)]^+$ Calcd: 625.9464, Found: 625.9165; $[M\text{-}3(pyr)]^+$ Calcd: 546.8743, Found: 546.8825

IR (cm^{-1}): 3010 (w), 1714 (w), 1605 (s), 1490 (w), 1418 (s), 1347 (w), 1218 (s), 1071 (m), 958 (w), 836 (s, PF_6^-), 768 (m), 593 (s), 527 (m), 557 (s)

^{1}H NMR

(600 MHz, CD_2Cl_2) δ/ppm: 8.90 (dt, *J* = 5.1, 1.4 Hz, 6H, pyr-H_{ortho}), 8.10 (tt, *J* = 7.5, 1.5 Hz, 3H, pyr-H_{para}), 7.69 (ddd, J = 7.4, 5.3, 1.2 Hz, 6H, pyr-H_{meta}), 2.10 (s, 18H, $-CH_3$)

(600 MHz, CD_3CN) δ/ppm: 8.88 (dt, *J* = 5.2, 1.3 Hz, 6H, pyr-H_{ortho}), 8.15 (tt, *J* = 7.6, 1.4 Hz, 3H, pyr-H_{para}), 7.74 (ddd, J = 7.4, 5.4, 1.3 Hz, 6H, pyr-H_{meta}), 2.07 (s, 18H, $-CH_3$)

(600 MHz, CD_3OD) δ/ppm: 8.99 (d, *J* = 5.6 Hz, 2H, pyr-H_{ortho}), 8.16 (t, *J* = 7.5 Hz, 3H, pyr-H_{para}), 7.77 (ddd, J = 6.9, Hz, 6H, pyr-H_{meta}), 2.11 (s, 18H, $-CH_3$) Note that complex begins to degrade in deuterated methanol over a matter of hours.

6.1.8 $Co_4(\mu_3\text{-}O)_4(OAc)_4(pyr)_4$, $\{Co_4O_4\}$

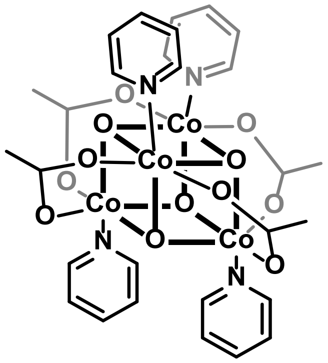

Adapted from literature procedure.[17]

Cobalt(III) nitrate hexahydrate (20.3 g, 70 mmol, Eq: 1.0) in methanol (210 mL) was combined with sodium acetate trihydrate (18.90 g, 140 mmol, Eq: 2.0) and heated to reflux. Pyridine (5.6 mL) was added to the mixture at temperature, followed by 17.5 M aqueous hydrogen peroxide (21.4 mL, 374 mmol, Eq: 5.3), added dropwise over 10 min, to afford a dark purple solution. Reflux was continued for 4 h with stirring, and the solution left to cool to r.t. overnight. The reaction mixture was concentrated *in vacuo*, the concentrate partitioned between dichloromethane and water, and extracted with further aliquots of dichloromethane until the aqueous layer became pink. The pink aqueous phase was discarded, and the dark green combined organic phase dried over sodium sulphate, before evaporation to dryness *in vacuo* to afford desired product as a dark green solid (12.0 g, 81%).

MS (ESI) m/z: $[M+H]^+$ Calcd: 852.9423, Found: 852.9526; $[M+H\text{-}pyr]^+$ Calcd: 773.9001, Found: 773.9094; $[M\text{-}pyr\text{-}AcO]^+$ Calcd: 713.8789, Found: 713.8861; $[M+H\text{-}2(pyr)]^+$ Calcd: 694.8579, Found: 694.8650

IR $\bar{\nu}/cm^{-1}$: 3109 (w), 3076 (w), 3004 (w), 2925 (w), 1719 (w), 1658 (w), 1606 (w), 1573 (w), 1534 (s, br), 1483 (m), 1449 (m), 1405 (s, br), 1337 (m), 1245 (w), 1212 (s), 1152 (m), 1071 (m), 1046 (m), 1019 (w), 878 (w), 760 (s), 690 (s), 647 (sh), 630 (s), 583 (sh), 570 (s)

^{1}H NMR

(600 MHz, CD_3CN) δ/ppm: 8.38 (d, *J* = 5.1 Hz, 8H, pyr-H_{ortho}), 7.58 (t, *J* = 7.5 Hz, 4H, pyr-H_{para}), 7.09 (t, *J* = 6.8 Hz, 8H, pyr-H_{meta}), 1.99 (s, 12H, $-CH_3$)

(600 MHz, CD_3OD) δ/ppm: 8.42 (dt, *J* = 5.0, 1.3 Hz, 8H, pyr-H_{ortho}), 7.68 (tt, *J* = 7.6, 1.4 Hz, 4H, pyr-H_{para}), 7.09 (ddd, *J* = 7.5, 5.0, 1.2 Hz, 8H, pyr-H_{meta}), 2.11 (s, 12H, $-CH_3$) Note that complex begins to degrade in deuterated methanol over a matter of days.

6.2 Autonomous Exploration General Procedure

Individual reaction conditions were generated digitally and imported to the Modular Wheel Platform as a comma-separated values (.csv) file listing addition volumes for each component.

The wheel was first primed twice with each component in turn (1 mL per component), and then reagents dispensed into empty 14 mL glass vials mounted on the wheel. Vials were stirred continuously throughout the addition process using an array of fan-mounted magnets positioned below the rotating wheel. Order of addition was invariant for each vial: (i) cluster solutions, (ii) lanthanide chloride hydrate additive solution, (iii) organic additive solution, and (iv) excess methanol. Cluster stock solutions were prepared as 0.012 M in MeCN, and additives as 0.012 M in MeOH. These and additional MeOH were connected to the platform with one reagent per pump to prevent contamination.

For the Common Components Exploration, clusters were added in the order: $\{Co_3O(OH)\}$, $\{Co_3O\}$, $\{Co_4O_4\}$. Additive order of addition was: $CeCl_3.7H_2O$, $DyCl_3.6H_2O$, $YbCl_3.6H_2O$, Oxalic Acid, Succinic Acid, 1,4,7-trimethyl-1,4,7-triazacyclononane. For the Isostructural Exploration, clusters were added in the order: $\{Cr_3O\}$, $\{Mn_3O\}$, $\{Fe_3O\}$, $\{Co_3O\}$. Additive order of addition was the same.

Once all samples had been combined, the vials were transferred to an aluminium block preheated to 55 °C (to afford an internal temperature of 50 °C) and with bored holes of appropriate diameter for the vials used. Reaction mixtures were held at temperature for 30 min, before being removed from the heat source and cooled to room temperature, sampled for MS analysis, and left to crystallise by slow evaporation.

A second set of samples were then prepared by the same method. Prior to heating, these samples were separated into two aliquots of 3.75 mL each, and subjected to two different heating conditions. One set was immediately transferred to long-term storage to permit the reaction to proceed at room temperature, and the other subjected to solvothermal conditions. 14 mL screw-top vials with septa (for reliable sealing) were charged with the reaction mixtures for solvothermal heating, and these transferred to an oven at 90 °C for four days. Samples of each set were taken for MS analysis, and the vials set aside for crystallisation. In cases where the solvothermal samples produced precipitate or crystalline material, this was separated from the mother liquor via centrifugation and the liquor permitted to crystallise. Both (i) the solid material recovered post-separation, and (ii) any crystals subsequently formed from the mother liquor were probed by SCXRD.

MS samples were prepared from reaction mixture (100 µL) diluted in acetonitrile (1000 µL, analytical grade). Data was exported in the extensible markup language (.xml) format, interpreted digitally, and converted to a single value mapping function indicating likelihood of a given reaction composition affording new species. This mapping function value was then used to inform subsequent data sets chosen by a Bayesian optimiser (using a model of the search space constructed from Gaussian processes as the prior). For more information on the (semi-)random and algorithmic generation of sampling conditions, please see section S6.5.

6.3 Additional Characterisation Data

6.3.1 Heteroleptic Cobalt(III) Anhydride Complex, $[Co(Ac_2O)_2OAc]X_2$

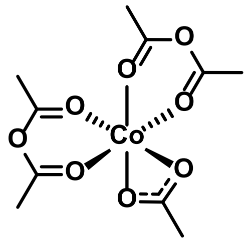

^{1}**H NMR** (600 MHz, MeOD) δ/ppm: 2.48 (s, 6H, anhydride), 2.25 (s, 6H, anhydride), 2.25 (s, 3H, acetate)

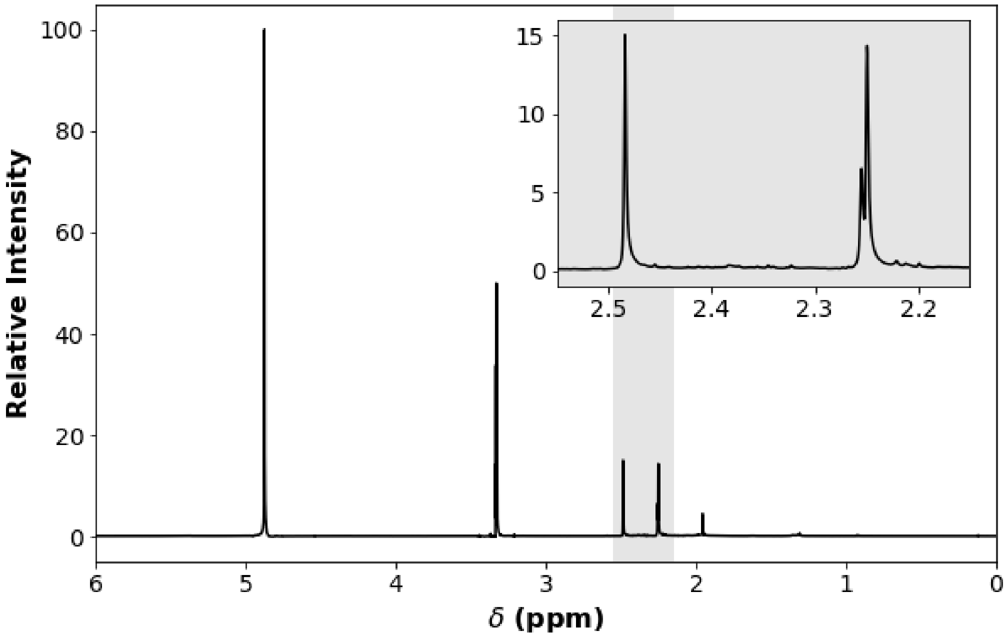

***Figure S51**: NMR spectrum of $[Co(Ac_2O)OAc]X_2$ in CD_3OD. Inset shows an expanded view of the area highlighted in grey containing peaks of interest.*

ESI-MS data deconvoluted from the exploration data via the process, detailed in the manuscript and later in this document, has been assigned further. Note that the resolution of the MS data is reduced during digital interpretation of the data, this reduced data is specified as 'Found' followed by the .

MS (ESI) m/z: $[M+CH_3OH+e^-]^+$ Calcd: 354.035554, Found: 354; $[M+PF_6+H+e^-]^+$ Calcd: 467.981345, Found: 468; $[M+PF_6+H+H_2O+e^-]^+$ Calcd: 485.991910, Found: 486; $[M+PF_6+H+CH_3OH+e^-]^+$ Calcd: 500.007560, Found: 500

6.3.2 $\{Fe_{10}\}$ Ring, $[Fe(OMe)_2OAc]_{10}$

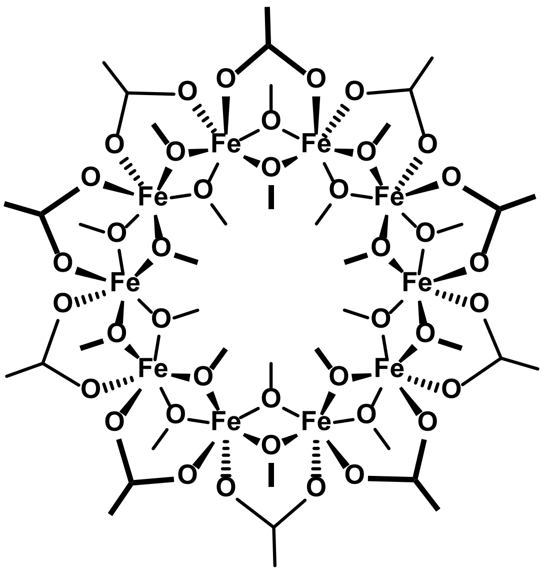

IR $\bar{\nu}$/cm^{-1}: 2923 (m), 2820 (m), 1529 (s), 1433 (s), 1348 (w), 1038 (s), 665 (m), 617 (w), 530 (m)

6.3.3 $\{M_{10}\}$ Ring, $[M(OMe)_2OAc]_{10}$

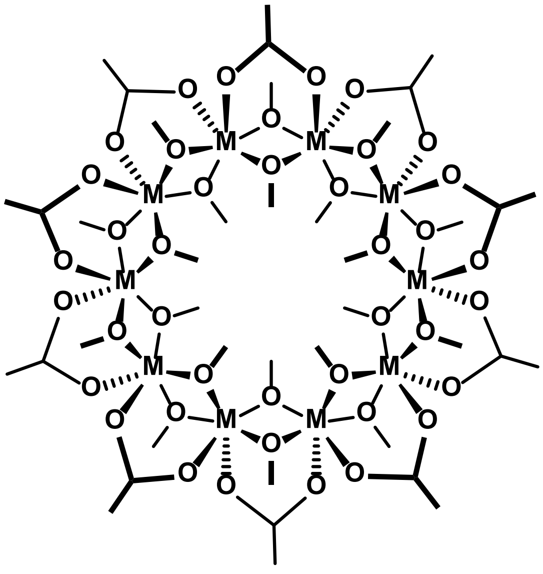

IR $\bar{\nu}$/cm^{-1}: 2926 (m), 2822 (m), 1535 (s), 1412 (s), 1346 (w), 1027 (s), 675 (m), 619 (w), 562 (w), 542 (w), 520 (w), 508 (m)

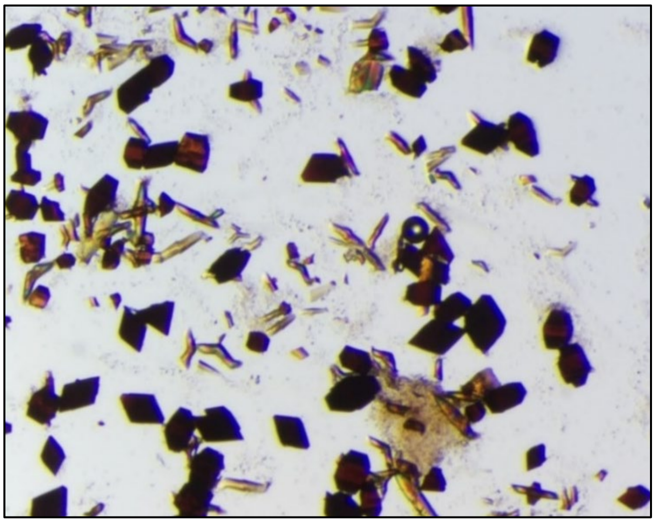

Figure S52: *Mix of crystals present in Solvothermal samples from the Isostructural Exploration. All diffract to give pattern characteristic of {M_{10}}*

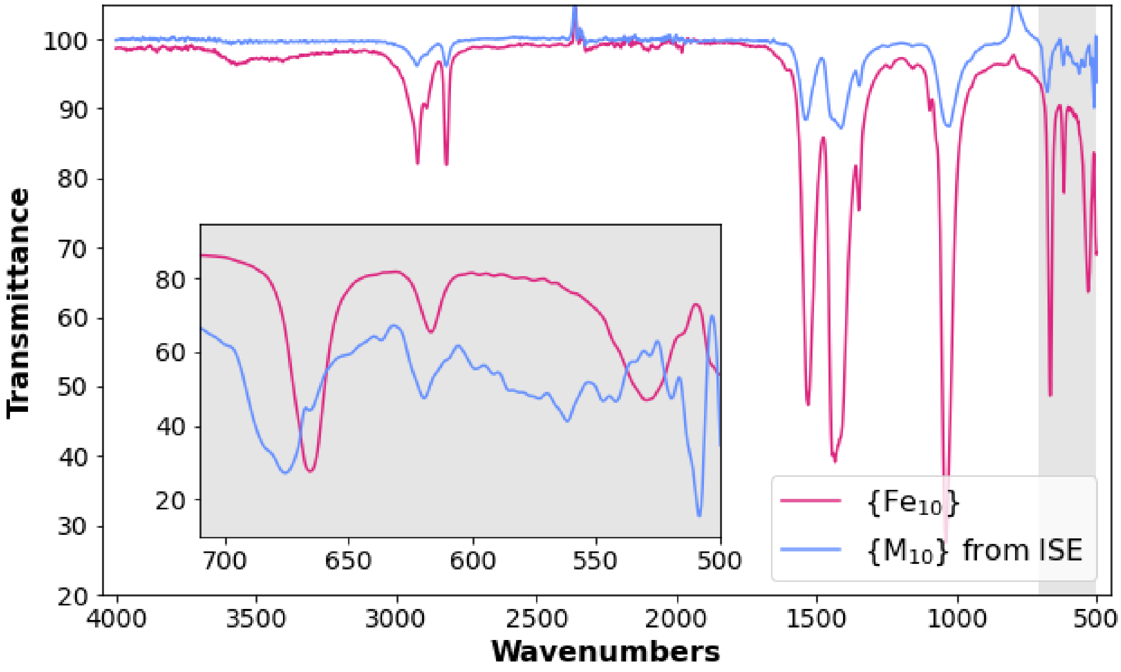

Figure S53: *Comparison of IR spectra for the pure {Fe_{10}} wheel, and the mixed metal {M_{10}} isolated from the Isostructural Exploration. Inset shows the typical M-O stretch region.*

6.4 Additional Experimental Notes

6.4.1 Monitoring Degradation in Starting Material Stock Solutions

The stability of each starting material was tested in those solvents we determined would be of interest for the exploration. These were acetonitrile, a coordinating solvent effective at solubilising metal complexes, and methanol, used to trigger reductive agglomeration of metal carboxylate clusters.[18]

In order to probe the stability of the diamagnetic Co(III) clusters, solutions of each cluster in deuterated solvents - anhydrous MeCN, wet MeCN (i.e. CD_3CN + few drops D_2O), and MeOH – were monitored by NMR after 0 hrs, 1 day and 5/6 days, to determine whether degradation proceeded notably. The spectra for these are shown in Fig. S52-S54. In each case, the most notable growth of degradation product peaks is shown for MeOH, with some evidence in wet MeCN, and little to no change in anhydrous MeCN. This behaviour was presumed to be comparable to the paramagnetic clusters, given their isostructural relationship.

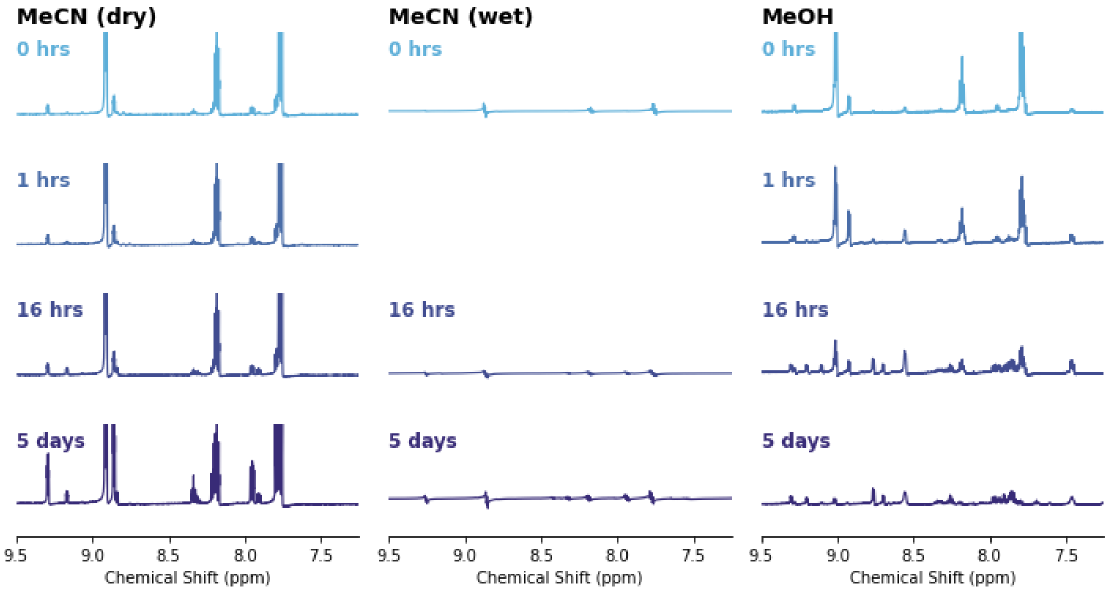

***Figure S54**: Degradation of {Co_3O} in various solvent mixtures. Characteristic peaks in the aromatic region shown only. Data was not recorded for wet MeCN after 1 hour.*

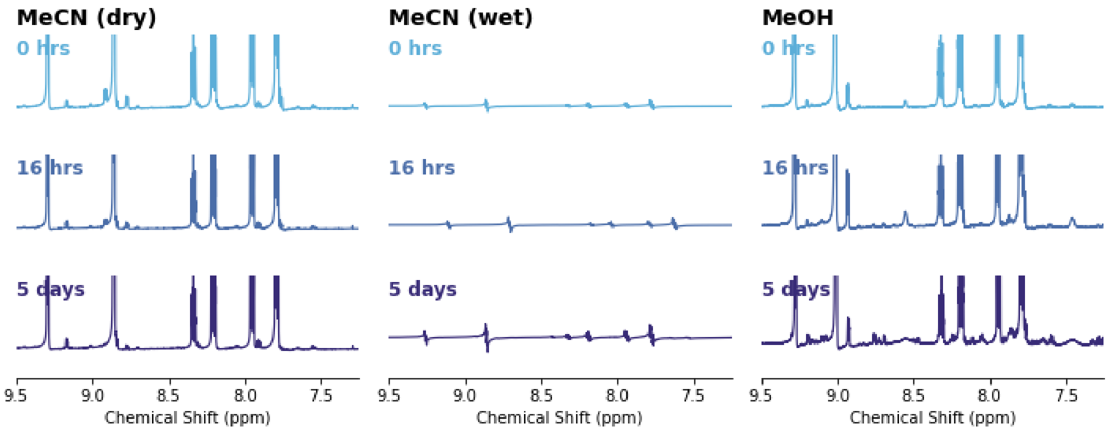

***Figure S55**: Degradation of {$Co_3O(OH)$} in various solvent mixtures. Characteristic peaks in the aromatic region shown only.*

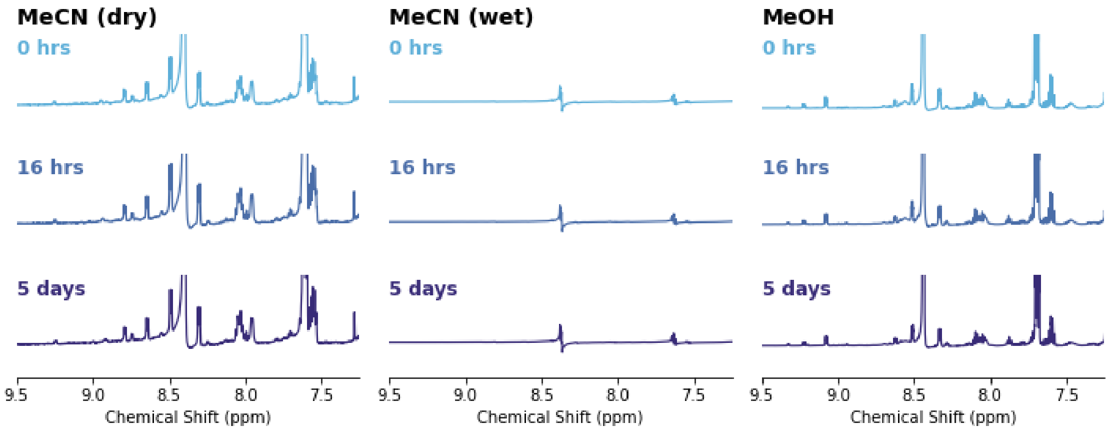

Figure S56: *Degradation of {Co_4O_4} in various solvent mixtures. Characteristic peaks in the aromatic region shown only. Note the growth of degradation product peaks is most noticeable in MeOH, then wet MeCN, and little change occurs for anhydrous MeCN.*

6.4.2 Origins of Acetic Anhydride under Solvothermal Condtions

The origin of acetic anhydride identified under solvothermal conditions in the Common Components exploration was probed via a series of NMR experiments.

Initially, the setup from Experiment 27 was replicated, as this had afforded the heteroleptic $[Co(Ac_2O)_2OAc]X_2$ species without requiring templates, thus simplifying further analysis. All possible combinations of cluster were probed (see Fig. S55) to attempt to isolate any relationship between cluster identity and anhydride generation. A peak with chemical shift, $\delta \cong 2.2$ ppm, assumed to be due to anhydride, was observed for all combinations with the singular exception of the reaction containing solely $\{Co_4O_4\}$.

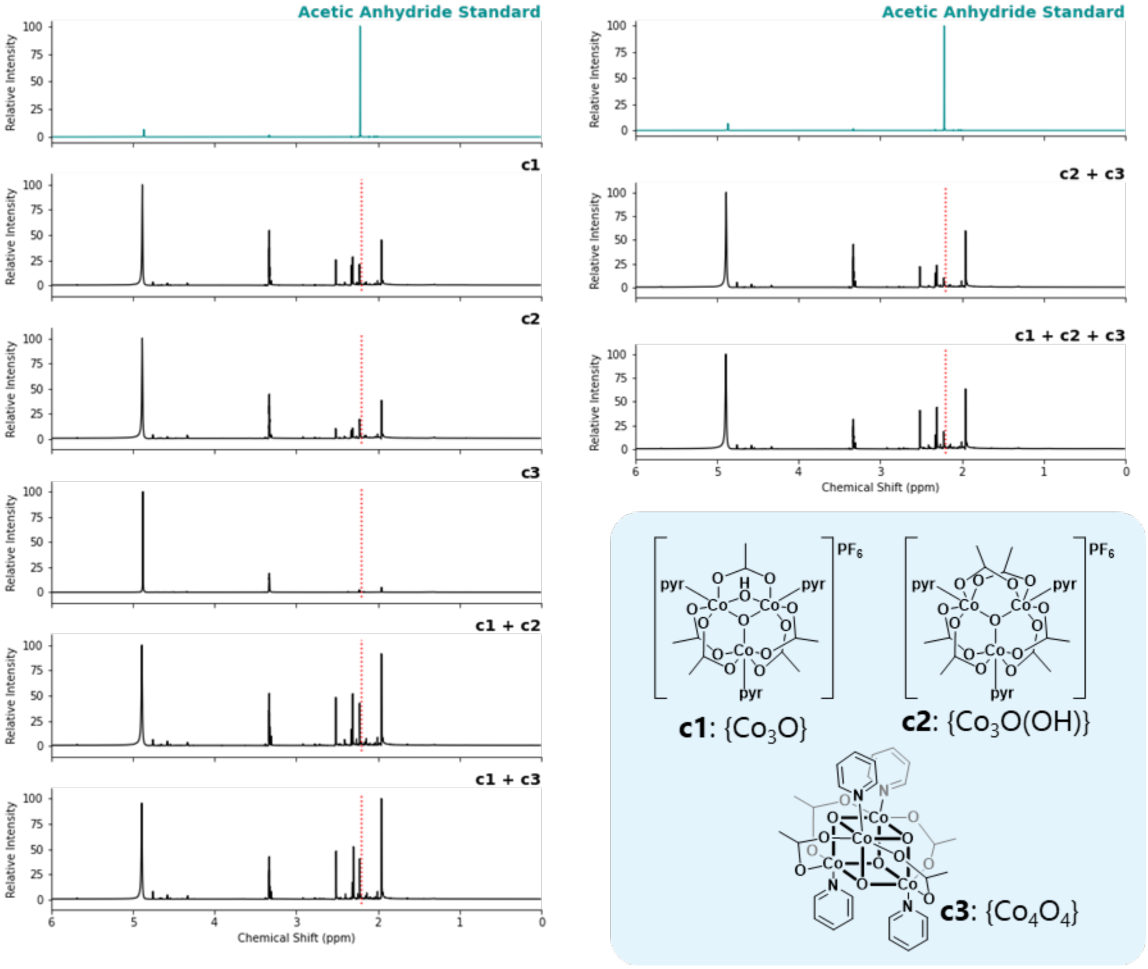

***Figure S57**: NMR spectra for probing reactions based on Common Components Experiment 27 to determine the conditions necessary for acetic anhydride formation. Conditions probed were identical to Experiment 27, but with different clusters omitted (as indicated for each spectrum). Reagent-grade acetic anhydride was used as control (top; spectra in green), red dotted lines indicate position of the single acetic anhydride peak.*

To determine whether the cobalt source or oxidation state was important in the formation of acetic anhydride, the solvothermal process was repeated using alternative cobalt complexes – cobalt(II) acetate tetrahydrate and trisacetoacetonatocobalt(III) – in place of the cluster materials. Additional acetic acid and pyridine were added to give the same concentration of these species in the system as could be expected when all three clusters were present in the amounts used for Common Components Experiment 27. Acetic anhydride peaks were recorded for both of these cobalt sources, indicating the formation does not depend on the oxidiation state or form of cobalt precursor. Crystals isolated from

these systems afforded the extended structure $[Co_5(OH)_2(O_2CMe)_8.2H_2O]_n$ (see Fig. S56). The absence of pyridine appeared to retard formation of anhydride.

Interestingly, without a cobalt source (i.e. acetic acid and pyridine in the 3:1 MeCN/MeOH solvent system), no acetic anhydride formation was observed. Instead, peaks corresponding to methyl acetonate were observed, indicating ester formation (potentially catalysed by the pyridine acting as a base).

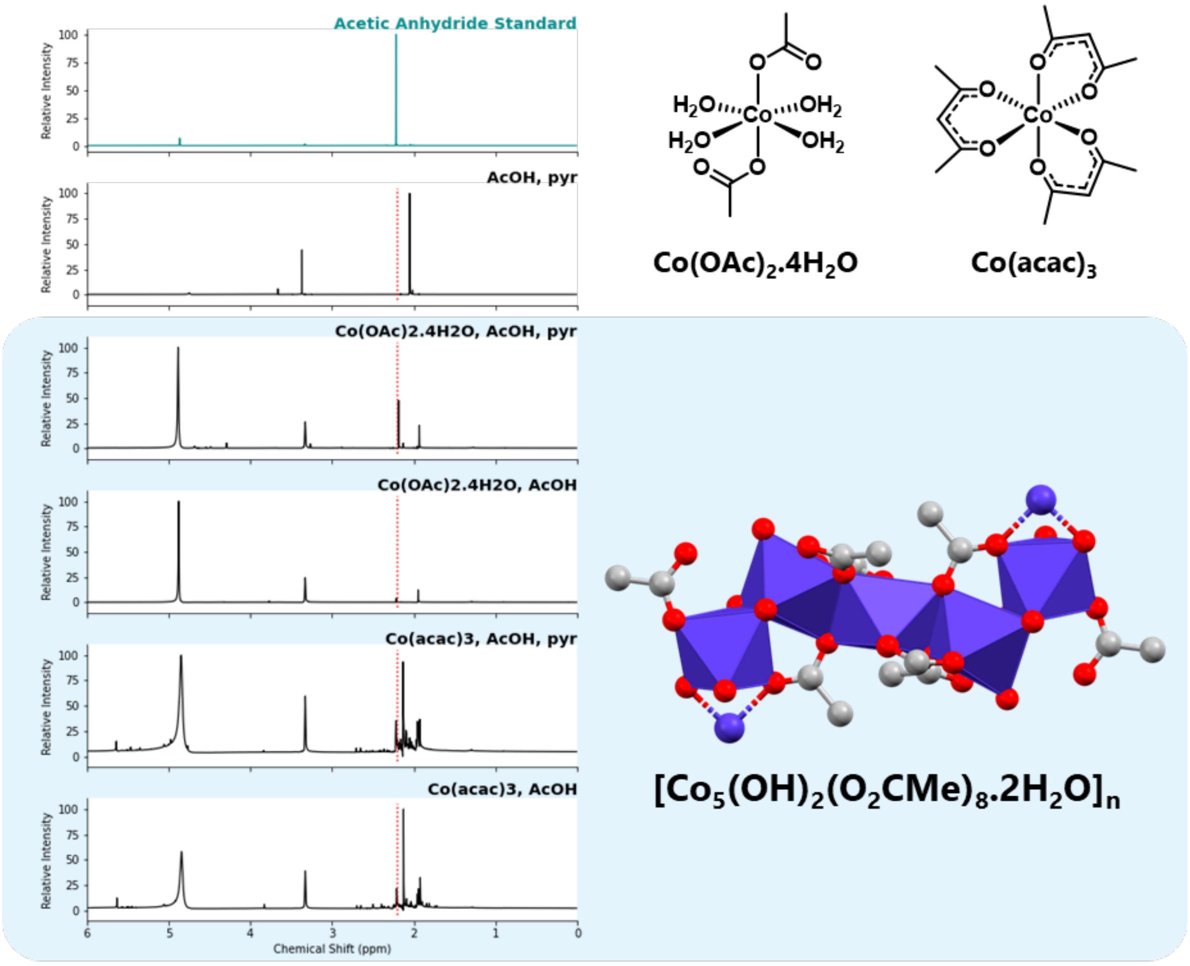

Figure S58: *NMR spectra for probing reactions based on Common Components Experiment 27 to determine the conditions necessary for acetic anhydride formation. Conditions probed used concentrations of Co, acetic acid, and pyridine identical to those present in Experiment 27. Reagent-grade acetic anhydride was used as control (top; spectra in green), red dotted lines indicate position of the single acetic anhydride peak. Extended structure crystallised from reactions with cobalt sources is also shown.*

Finally, samples of AcOH only in the solvent system from Experiment 27 (3:1 MeCN/MeOH), each individual component solvent (MeCN or MeOH) or pure acid were subjected to the same conditions (Fig. S57). No evidence was visible by NMR of anhydride formation under any of these conditions, but this may be considered unreliable, as the large solvent peaks may obscure a relatively small anhydride peak. In future, further quantification of this may be attempted using an adapted Liebermann-Burchard assay.[19]

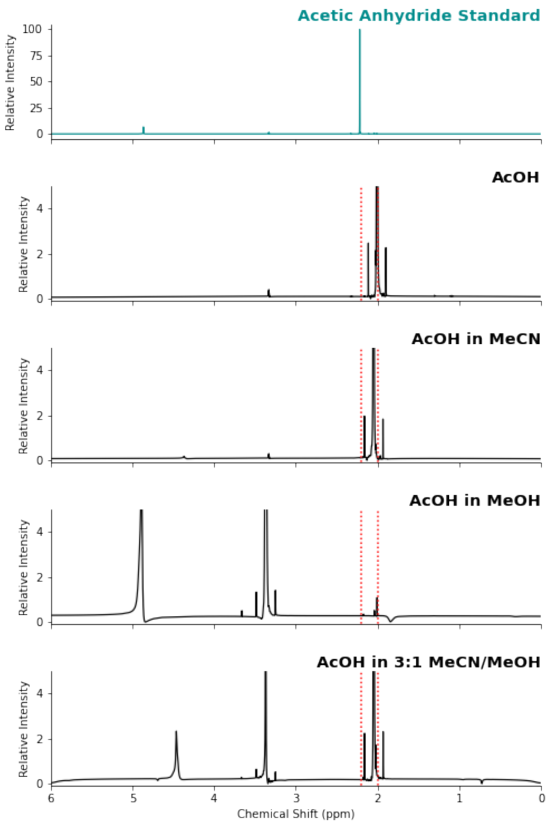

***Figure S59**: NMR spectra for probing reactions to determine the conditions necessary for acetic anhydride formation. Conditions probed used either pure acetic acid, or systems of 10 µL acetic acid in 3 mL solvent. Reagent-grade acetic anhydride was used as control (top; spectra in green), red dotted lines indicate position of the single acetic anhydride and acetic acid peaks.*

The above data implies that cobalt is necessary for the formation of anhydride in sizeable quantities under these solvothermal conditions, with pyridine seeming to act as a promoter, although it is unclear whether pyridine is a required component of this system or not. This may indicate a previously unknown pathway for the condensation of acetic acid under solvothermal conditions. Future work focusing on expanding the scope of the condensation to other carboxylic acids would potentially be of interest.

6.5 (Semi-)Random Sampling Methods

All code for the sampling of the search space, by whichever method used in a given iteration, was written in python3. Purely random sampling was used in Run 3 and quasi-random in Run 1.

Note that there is a strictly enforced limit on the maximum volume that can be dispensed in the system of 7.5 mL. Rather than permit varying volumes up to this limit, and the potential problems with sampling mixtures this may cause, we chose instead to fix the volume of every sample as 7.5 mL and instead permit the concentration to vary. This was achieved through the generation of a relative ratio of each reagent on sampling, that was then scaled according to a dilution factor, and additional methanol used to make the solution up to 7.5 mL.

All reagents can be assigned a value of 0 in the ratio, and the dilution factor may be 0, so it is practically possible for a sample to be any one reagent dispensed as the ‘pure’ solution. It is of note that one algorithm-directed sample was generated that contained solely methanol in Run 4. This is not of concern as the algorithm is primarily tune to explore rather than exploit the space, and the mapping function assigned this system the lowest possible value for novelty. However, this method of enforced makes the dispensing of a pure reagent is unlikely under (semi-)random conditions. Instead, the sampling is largely constrained around lower concentrations of reagents. The sampling distributions thus generated are shown in Figures S60-S63.

6.5.1 Uniform Random Sampling

Run 3 was conducted using uniform random sampling. Equivalent of sampling a probability distribution with the formula:

$$p(x) = \frac{1}{(b - a)}$$

Where x is any given variable defining the search space, a is the lower bound of acceptable values and b is the upper. To achieve this in python, the numpy package’s `numpy.random.uniform()` function was used to draw samples from a uniform distribution between lower bound, a = 0.0 and upper bound, b = 1.0 for clusters and b = 0.5 for templates. The lower value for templates was chosen to bias their concentrations to values to be generally lower than the clusters.

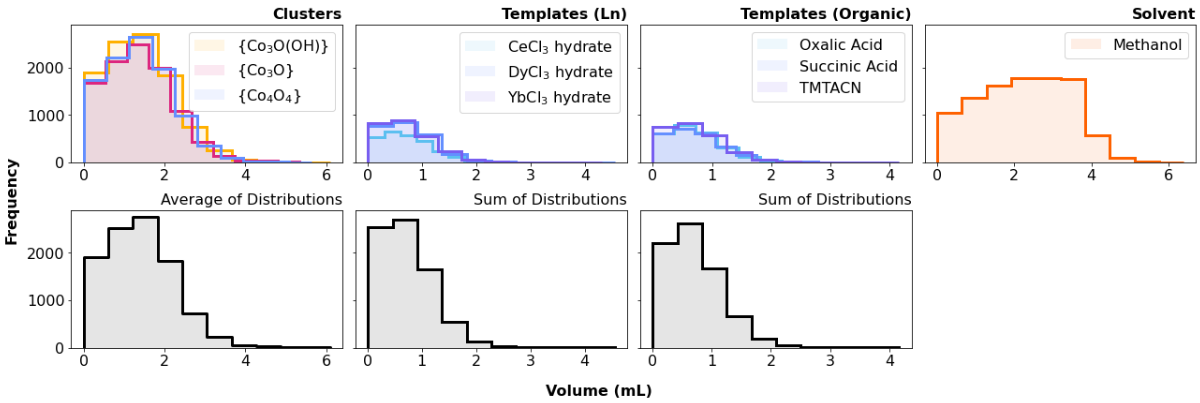

Figure S60*: Distribution of samples for each variable in the Common Component Exploration in the volume domain, on generation of 10032 samples via uniform random sampling in the ratio domain.*

6.5.2 Latin Hypercube Sampling

Run 1 was conducted via Latin Hypercube sampling using the Summit framework (which uses the implementation of LHS from pyDoE2).[20]

LHS provides a quasi-random sampling that ensures the true variability of the dataset is represented, which purely random sampling may or may not achieve. Conceptually, LHS divides the space into a grid with the same number of divisions for each variable/axis and introduces sample points in such a way as to avoid placing any two points in the same row or column. This provides a greater spread of datapoints across each variable than a grid search, which would repeat the same values along each variable multiple times. The LHS method generalises to any arbitrary number of dimensions.[21-22]

Boundaries for the sampling are the same as for the random uniform case.

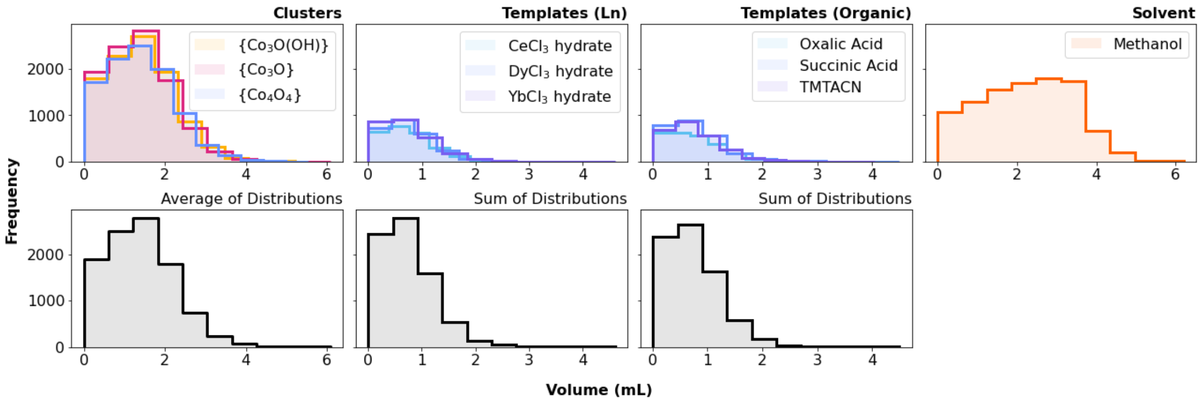

***Figure S61**: Distribution of samples for each variable in the Common Component Exploration in the volume domain, on generation of 10032 samples via Latin Hypercube Sampling in the ratio domain.*

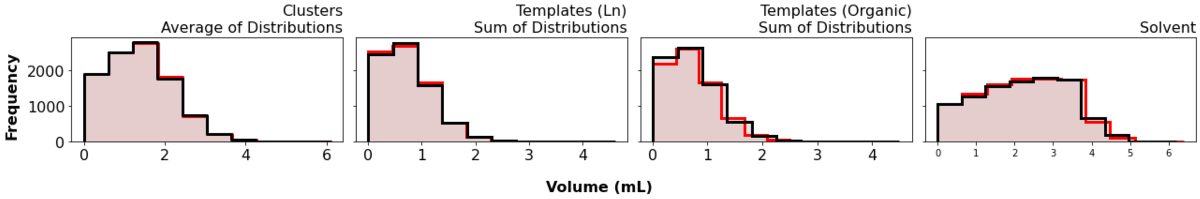

***Figure S62**: Comparison of the Distributions generated by Random Uniform (red) and Latin Hypercube Sampling (black).*

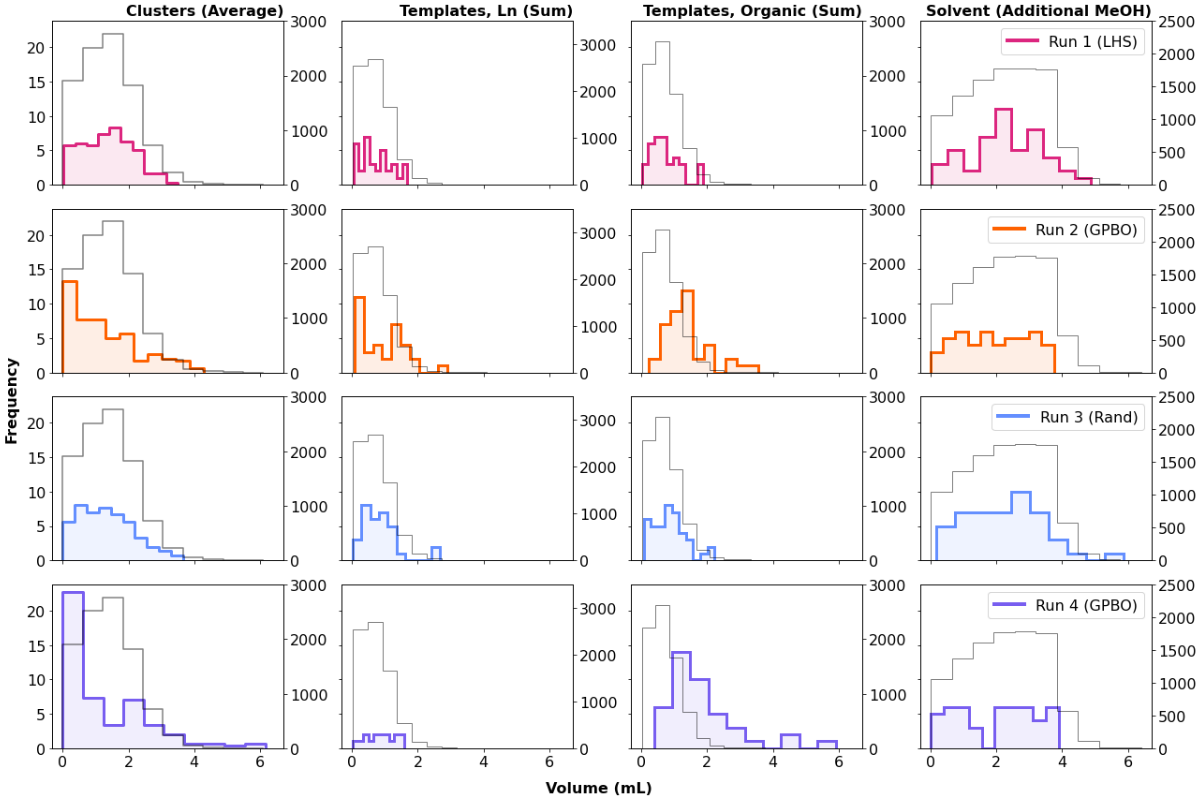

Figure S63: *Distributions of empirical samples from the Common Component Exploration in the volume domain. Light grey line indicates the associated distribution generated by random uniform sampling alone (see Figure S60, for values see right hand axis). Note that the algorithm-directed iterations show a significant deviation from the (semi-)random distributions, indicating the effect of the internal model.*

6.6 Gaussian Process Bayesian Optimisation

Runs 2 and 4 were conducted using a Bayesian Optimiser as the sampling method with a posterior constructed from Gaussian Processes and a Constant Liar strategy used to permit sampling an iteration with size > 1. A conceptual introduction (with minimal equations) is provided below to allow non-specialist chemists to build a better intuition of how the model works.

All code for the sampling of the search space, by whichever method used in a given iteration, was written in python3. GPBO was implemented using scikit-optimize's `skopt.Optimizer()` class and the ask/tell interface. This provides a readymade GP Bayesian optimiser, with the ability to tune key hyperparameters.

6.6.1 Surrogate Model

As a surrogate model, we choose a Gaussian process, which is used to describe a distribution over functions. Each of these functions is a possible description of the observed data.

$$f(\mathbf{x}) \sim \mathcal{GP}\big(m(\mathbf{x}), k(\mathbf{x}, \mathbf{x}')\big)$$

Where f(x) is the distribution or process, m(x) is the mean function and k(x,x') is the covariance function. The mean function is often chosen to be zero for ease. The covariance function specifies covariance between pairs of random variables – implying a particular probability distribution over functions.

To restrict the probability distribution over functions based on observations, we can condition the Gaussian process – effectively 'fixing' the value of the response at the observation point. As chemical observations are inherently noisy, a noise term is introduced to give a predictive distribution for the test points.

The hyperparameters of the covariance function (and potentially the mean function, if not chosen to be zero) are then evaluated internally to maximise the marginal likelihood, and train the Gaussian Process.

$$p(\mathbf{y}|X) = \int p(\mathbf{y}|\mathbf{f}, X)\, p(\mathbf{f}|X) d\mathbf{f}$$

This permits calculation of the probability of a set of noisy function values, $\mathbf{y}$ given the observed data, X based solely on the observed data and covariance matrix, without requiring knowledge of the underlying true function, $\mathbf{f}$. The trained Gaussian Process acts as the Bayesian surrogate model and is used to select the new sampling point(s) by the acquisition function.

This process of generating a GP based on the conditioned data, training the associated hyperparameters, and then using the trained surrogate model to permit the acquisition function to determine where to sample is iterated until the end of the exploration process.

A more detailed explanation can be found in Rasmussen and Williams.[23]

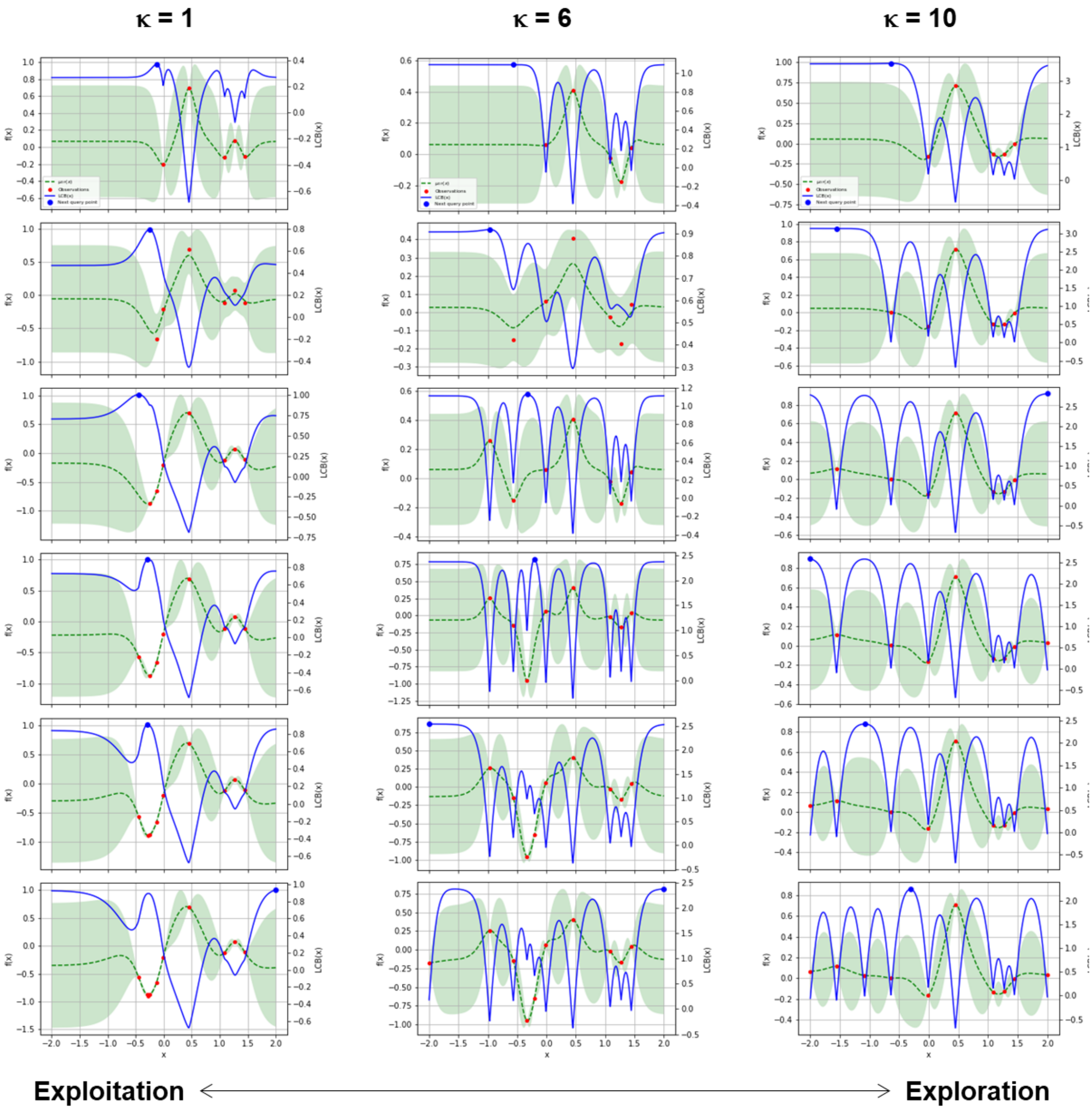

Figure S64: *Exemplar Gaussian process Bayesian optimisations on a simple 1-D function using LCB as acquisition function and with a range of kappa values. Successful minimisation is taken to be an optimised system here (and by default in the scikit-learn package. Red points are observations, green dotted line indiciates the GP model of the function, with green shading showing the confidence.The blue line indicates the acquisition function and blue point the next observation. Note that lower kappa heavily favours the exploitation of the space, whilst higher values increase the tendency toward exploration. Target function used is* $f(x) = sin(5x)\,(1 - tanh(x^2))$ *with the addition of some artificial noise (set to 10% of the function value).*

6.6.1 Acquisition Function

The values of the predicted response and the associated uncertainty can be used to direct exploration. Acquisition functions are used to determine the location of the next sample in the search space.[24-25]

In this work, the acquisition function used was the lower confidence bound (LCB), defined as:

$$LCB(x) = \mu_{GP}(x) + \kappa\sigma_{GP}(x)$$

Where μ is the predicted value of the objective function, σ is the square root of the variance, and κ is a weighting parameter ($\kappa > 0$). Higher values of κ increase the weighting from the variance (i.e. the uncertainty of the prediction) and favour exploration of the search space, whilst lower values favour μ and exploitation. This method is sometimes referred to as the upper confidence bound (UCB) depending on whether the optimisation problem is phrased as a maximisation or minimisation. In keeping with the preset view of the scikit-learn package, we discuss optimisation herein as a minimisation.

LCB was chosen over other methods due to this ability to tune the style of sampling (exploitation vs. exploration) the optimiser employs. Probability of Improvement, PI is geared more toward exploitation and the reward for improvement is constant independent of size of improvement, which can lead to underexploring globally. Expected Improvement, EI accounts for the size of the improvement, and permits larger, less certain improvements to be selected over smaller, very certain ones. As such, EI does capture the exploitation-exploration trade-off innately. EI would be a valid option for our purposes, however, we prefer LCB in this case, as the single parameter to control the trade-off permits tailoring of the activity of the optimisation with minimum additional knowledge. The value of κ may also be altered from interation to interation, depending on need.[26-27]

6.6.2 Constant Liar Strategy

Unaltered Bayesian Optimisation works on a point-by-point basis, as shown in Fig S60, with the prediction updated after each observation. This is prohibitively slow for chemical exploration and so the Constant Liar strategy is employed to permit the optimiser to generate multiple sampling points at once (i.e. iterations of size >1).[28]

The procedure is conducted as follows:

1. An initial Gaussian Process prediction of the response is generated, and the acquisition function used to predict the best point at which to sample.
2. A dummy response (a 'lie') is assumed for this point, which may be set to the minimum, maximum or mean of the current observations. For this exploration, the minimum was chosen.
3. The Gaussian process prediction is updated with this lie and the acquisition function used to predict the next best sampling point.
4. This process is then iterated until the desired sample size is reached.

6.7 Mapping Function

6.7.1 Overview Flowchart

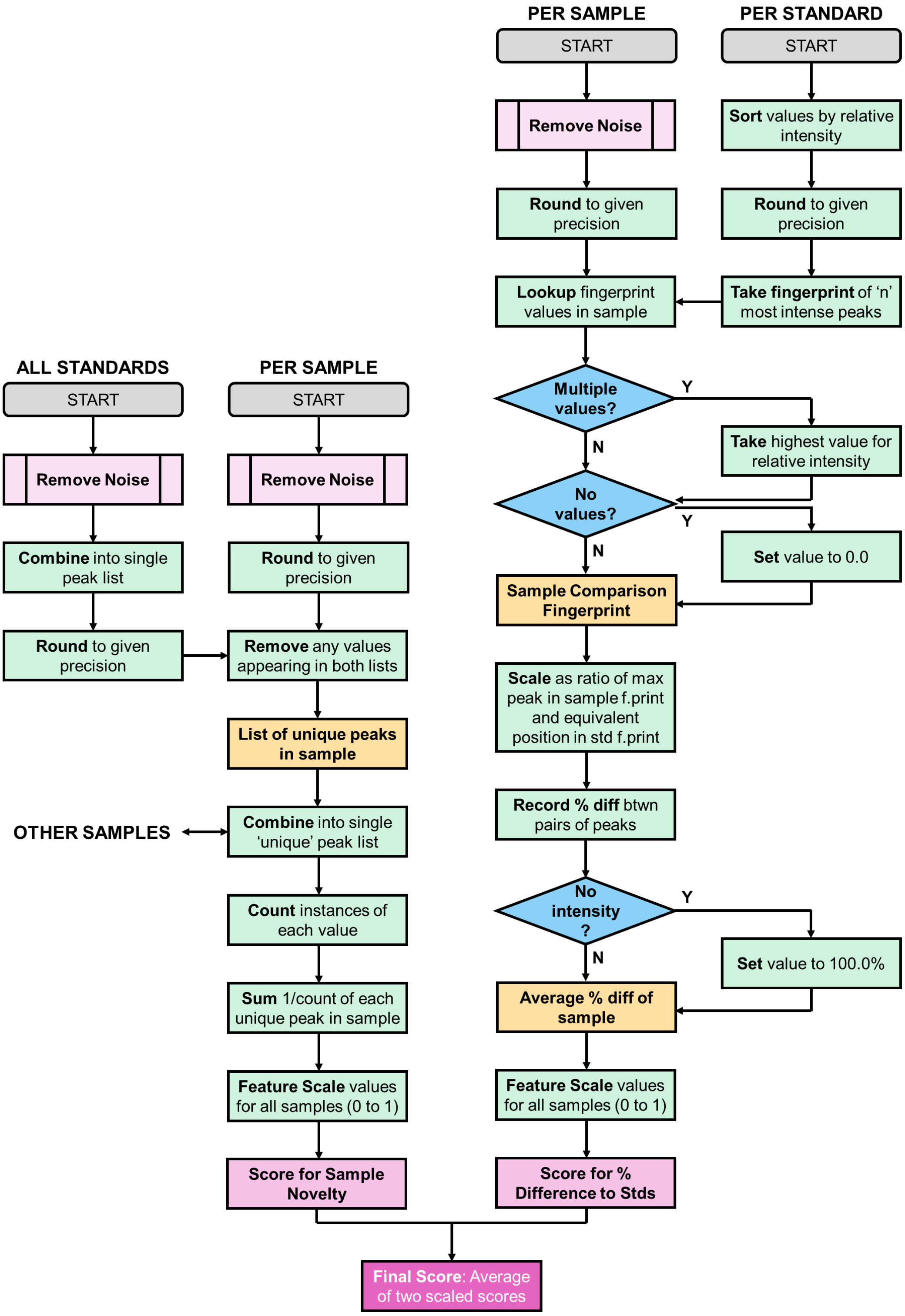

***Figure S65**: Overview of the algorithmic calculation of Analytical Mapping Function score from MS spectral data. Final score is the average of two separate measures: sample novelty (left), and % difference to starting material standards (right).*

6.7.2 Validation of Samples

Prior to calculation of the mapping function, two tests were applied to the dataset to validate that each sample was of sufficient weight to be counted in the calculation. Validation focused on two criteria: validation of reagents and validation of intensity.

Reagent Validation. Simple test to check that the dataset uploaded had the correct number of metal precursors (3 for the Common Component Exploration, and 4 for the Isostructural). Additionally, determined how many of the possible precursors had actually been added in each sample (i.e. had non-zero values).

This data was then used when averaging to ensure the % difference to standards of each sample calculated was not skewed in samples where fewer possible precursors had been used.

Intensity Validation. Consisted of determining the maximum intensity present in any sample across the entire dataset and removal from consideration of any samples which had no peaks with intensity over a certain percentage of that dataset maximum. Aim was the remove samples which completely consisted of noise, which would otherwise give rise to anomalous results.

As intensity was re-validated each iteration to permit for new, more-intense spectra, those samples discounted by this measure varied throughout the experiment. For the final iteration, a threshold of 7.5 % the maximum intensity across the dataset excluded between 5 and 22 % of samples from the calculation of sample novelty, with most exclusions focused on the later samples. This is expected as the final GPBO-led iteration focused on lower reaction concentrations. Were the exploration to run longer, it could be expected that this acts as an effective floor for the acceptable reaction concentration, limiting the exploration to within the range at which our analytical techniques are most effective.

6.7.3 Examples: Sample Novelty

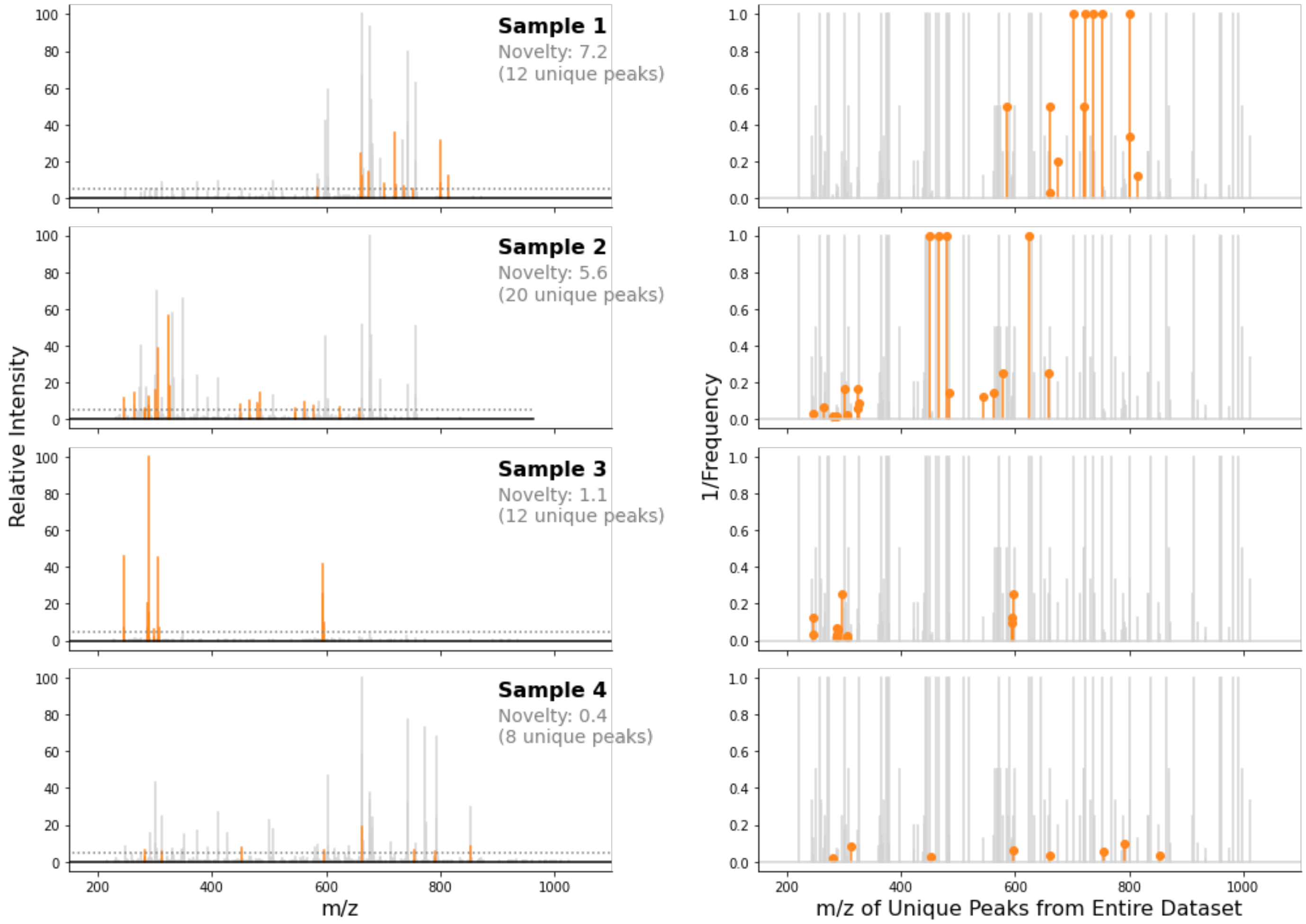

***Figure S66**: Identification of unique peaks in a representative range of sample spectra from the Warm dataset. Sample spectra are displayed with Grey dashed lines indicate the noise threshold (5% of max peak intensity) applied to each sample spectrum.*

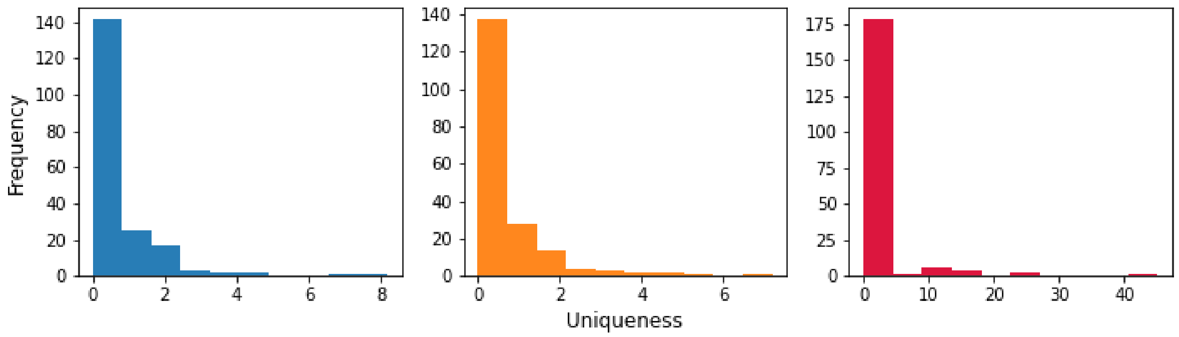

***Figure S67**: Histograms indicating the spread of novelty values across the Common Component datasets. From left to right: Ambient, Warm, Solvothermal.*

6.7.4 Examples: Average % Difference to Standards Component

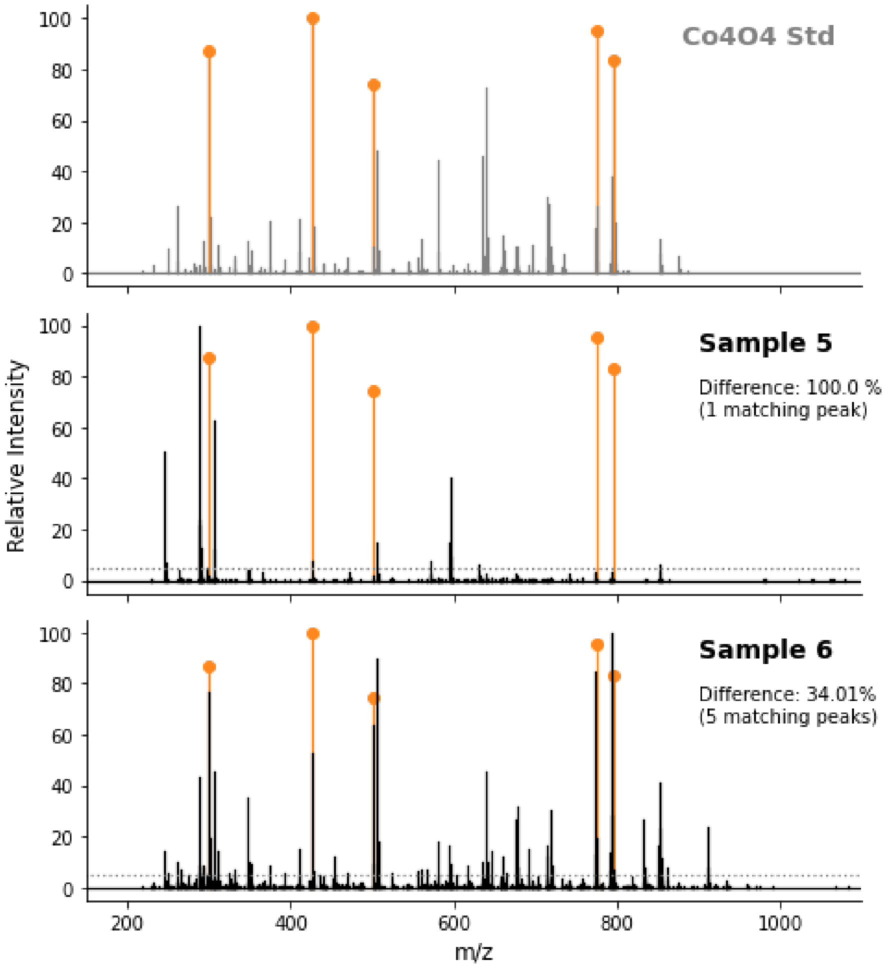

***Figure S68**: Comparison of two sample spectra from the Warm dataset against the {Co_4O_4} standard. A fingerprint of the five most intense peaks is identified for the standard (denoted with red dots), and the relative intensities of this fingerprint compared with the relative intensities of the same peaks in the sample spectra. The standard fingerprint is overlaid with the sample spectra to demonstrate the difference between poor agreement to the standard (Sample 5) and good agreement (Sample 6). Grey dashed lines indicate the noise threshold (5% of max peak intensity) applied to each sample spectrum.*

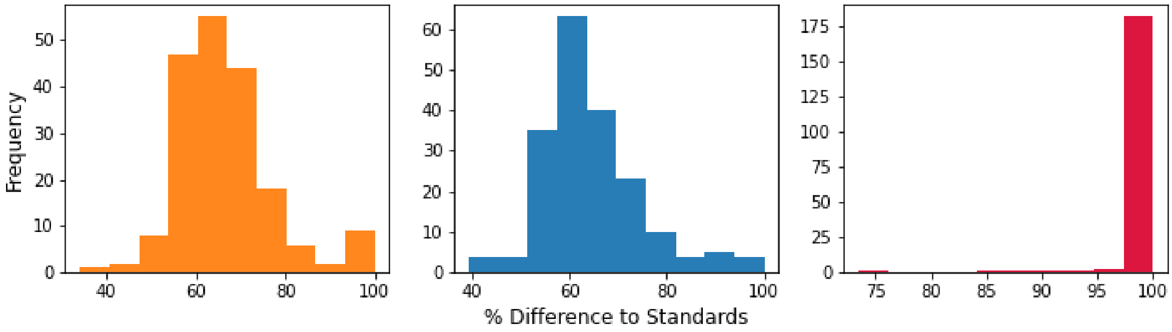

***Figure S69**: Histograms indicating the spread of % difference to all standards values across the Common Component datasets. From left to right: Ambient, Warm, Solvothermal.*

6.7.5 Test Dataset

To test the performance of the metric in a known setting, a test dataset was constructed from real-life MS data.

9 spectra were taken, with 4 assigned as standards and the remaining 5 as samples. Pairwise combinations were then created between each set of standards, between each set of samples, and between each sample and standard pair. These spectra were then subjected to the same mapping function as for the full exploration and the data from these visualised. This workflow is shown in Fig. S64.

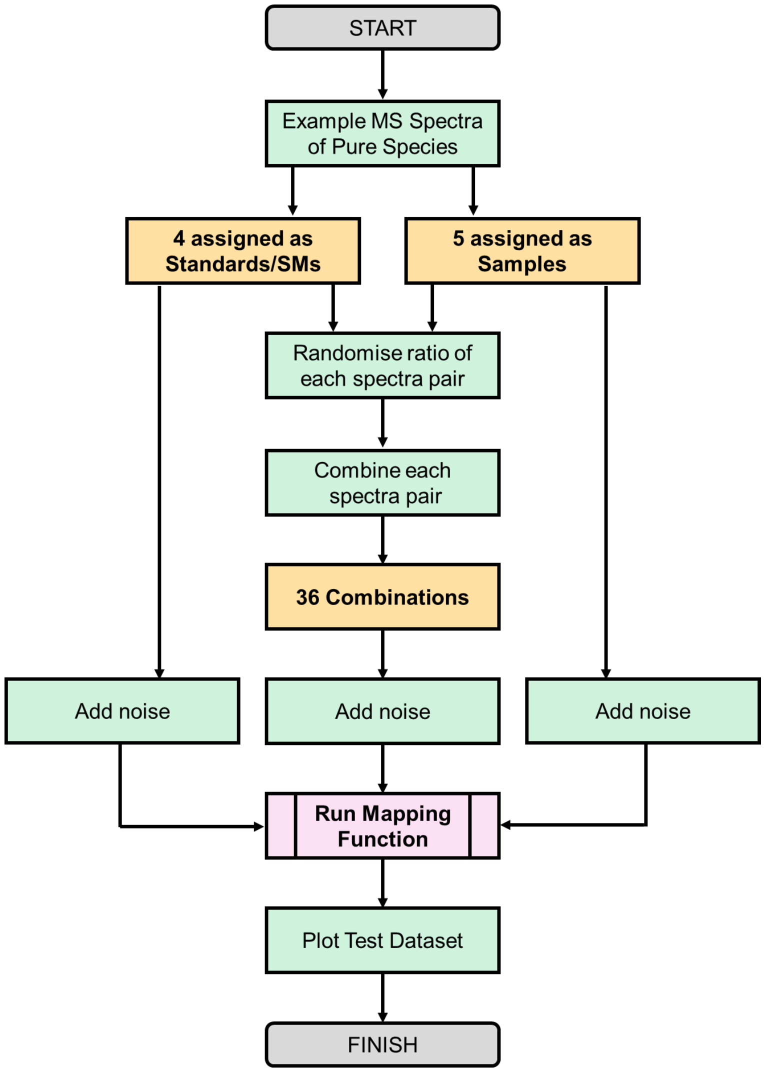

Figure S70*: Workflow for the generation and measurement of the mapping function test dataset.*

A few adjustments were made to help the test dataset best approximate real data. For each spectrum, peak position noise was created by providing each peak a 10% chance of having its m/z value reduced by 1. Peak intensity noise was created by adjusting the height of each peak to within ± 20% of the original value. Additionally, for each combination, the two spectra were scaled by a random factor, up to 100% of their original value, to avoid each combination being in a direct 1:1 ratio.

This process was able to produce datasets with the expected distribution of datapoints based on the method of generating a given datapoint (see Fig. S65).

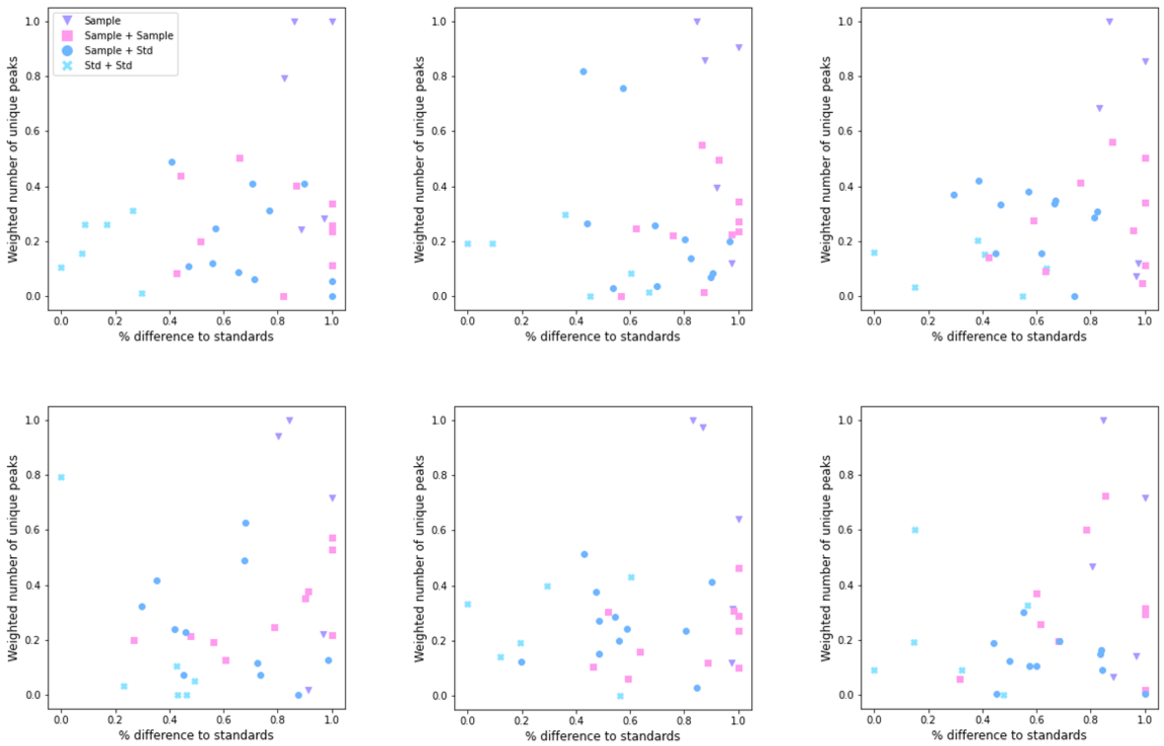

***Figure S71**: Six test datasets plotted to show variation in % difference to starting materials/standards and weighted number of unique peaks – the two halves of the novelty metric. As shown in the legend, noise-adjusted sample spectra are shown as purple triangles, sample+sample combinations as pink squares, sample+standard combinations as blue circles, and standard+standard combinations as cyan crosses.*

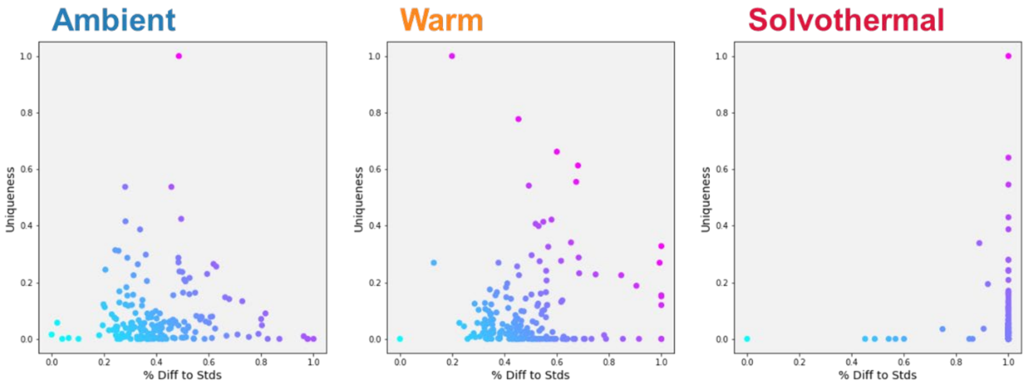

***Figure S72**: Equivalent plots to Fig. S65 using the data measured from the Common Component exploration. Colour of point refers to the combined mapping score (from low: cyan, to high: pink). Note that conversion from starting materials tended to be higher under the solvothermal conditions, resulting in a different spread of data for this plot.*

The spread of data also appeared to be a reasonable approximation of the data collected from explorations. The discrepancy is probably be due to the use of pure standard and sample spectra in the test dataset with only the noise adjustments listed above. These systems are likely to be far purer than the measured data, and the variation between samples is likely much higher in the test dataset (note that the mapping function automatically feature scales the data between 0 and 1 along each component of the metric).

Indeed, if the data are split by their origins, the points originating from the combination of standard and sample spectrum pairs appear to be the closest match to measured data for Ambient and Warm conditions, as would be expected.

6.8 Reproducibility of MS Data from Explorations

Table S6: *Comparison of % of shared peaks between original spectra (O), and two sets of repeated experiments (R1, R2) for high scoring reactions from across the dataset. For a peak with particular m/z to be counted towards or against this % value, it must be above a noise threshold of 5% of max peak height in at least one of the two datasets compared. Repeat 1 for CCE1_19 failed, and so comparisons against this are omitted.*

Rxn ID	% Shared Peaks<br>Ambient Conditions			Warm Conditions		
	R1 vs. R2	*O vs. R1*	*O vs. R2*	*R1 vs. R2*	*O vs. R1*	*O vs. R2*
CCE1_19	---	---	81.25	100.00	79.59	78.00
CCE2_00	100.00	42.42	41.38	100.00	76.92	83.33
CCE2_02	100.00	87.50	88.24	100.00	49.06	47.06
CCE2_04	100.00	54.84	61.29	100.00	47.62	49.21
CCE2_08	100.00	86.21	89.47	100.00	83.08	84.38
CCE2_10	100.00	79.69	80.95	100.00	80.26	81.58
CCE2_12	100.00	78.79	83.78	100.00	90.00	86.44
CCE2_35	100.00	96.15	96.30	100.00	100.00	100.00
CCE2_38	100.00	76.47	80.00	100.00	74.29	78.12
CCE2_47	100.00	92.86	92.65	100.00	88.64	86.05
CCE3_19	100.00	72.97	74.36	100.00	96.55	96.67
CCE4_04	100.00	51.35	45.45	100.00	52.63	57.50
CCE4_07	100.00	75.76	77.14	100.00	80.65	78.12
CCE4_08	100.00	73.53	75.76	100.00	67.86	72.00
CCE4_13	100.00	83.33	80.33	100.00	89.36	93.02
CCE4_31	100.00	80.00	80.43	100.00	97.62	97.62
CCE4_37	100.00	87.88	90.91	100.00	73.33	72.41
CCE4_43	100.00	60.0	60.47	100.00	79.37	81.82

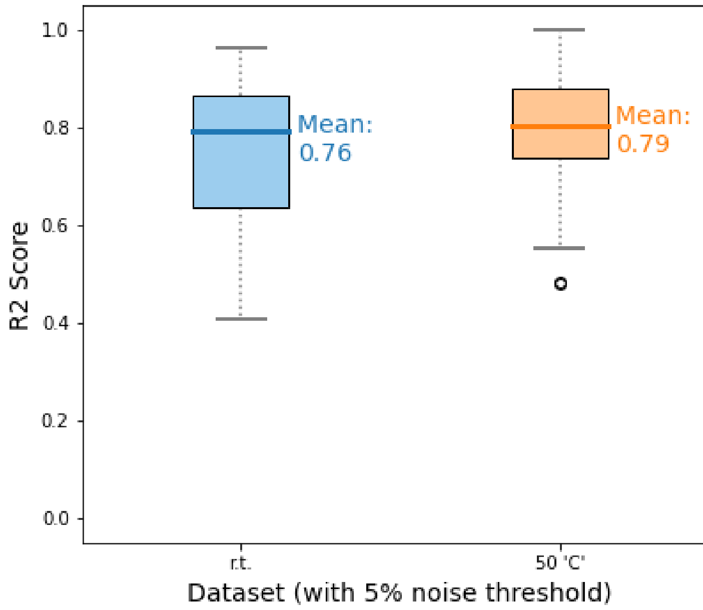

Figure S73: *Boxplots showing the % shared peaks between the originally measured spectra and the average of the two repeated spectra.*

6.9 Reaction Conditions and Results from the Common Component Exploration

6.9.1 Overview

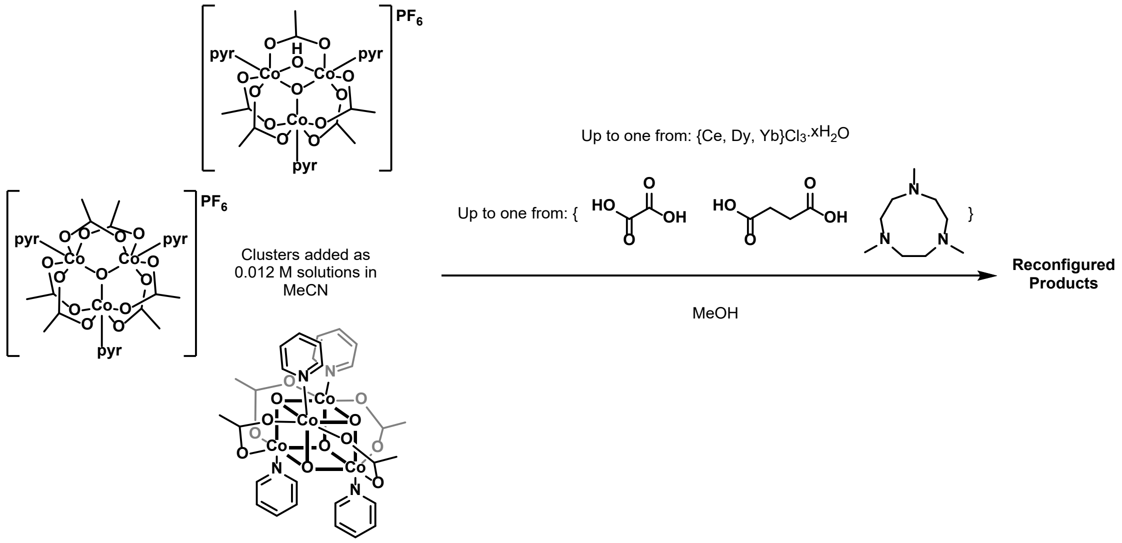

***Scheme S1**: General reaction scheme for the Common Component Exploration. Not all clusters were used in every experiment.*

6.9.2 Graphical Representations of Reaction Conditions Sampled

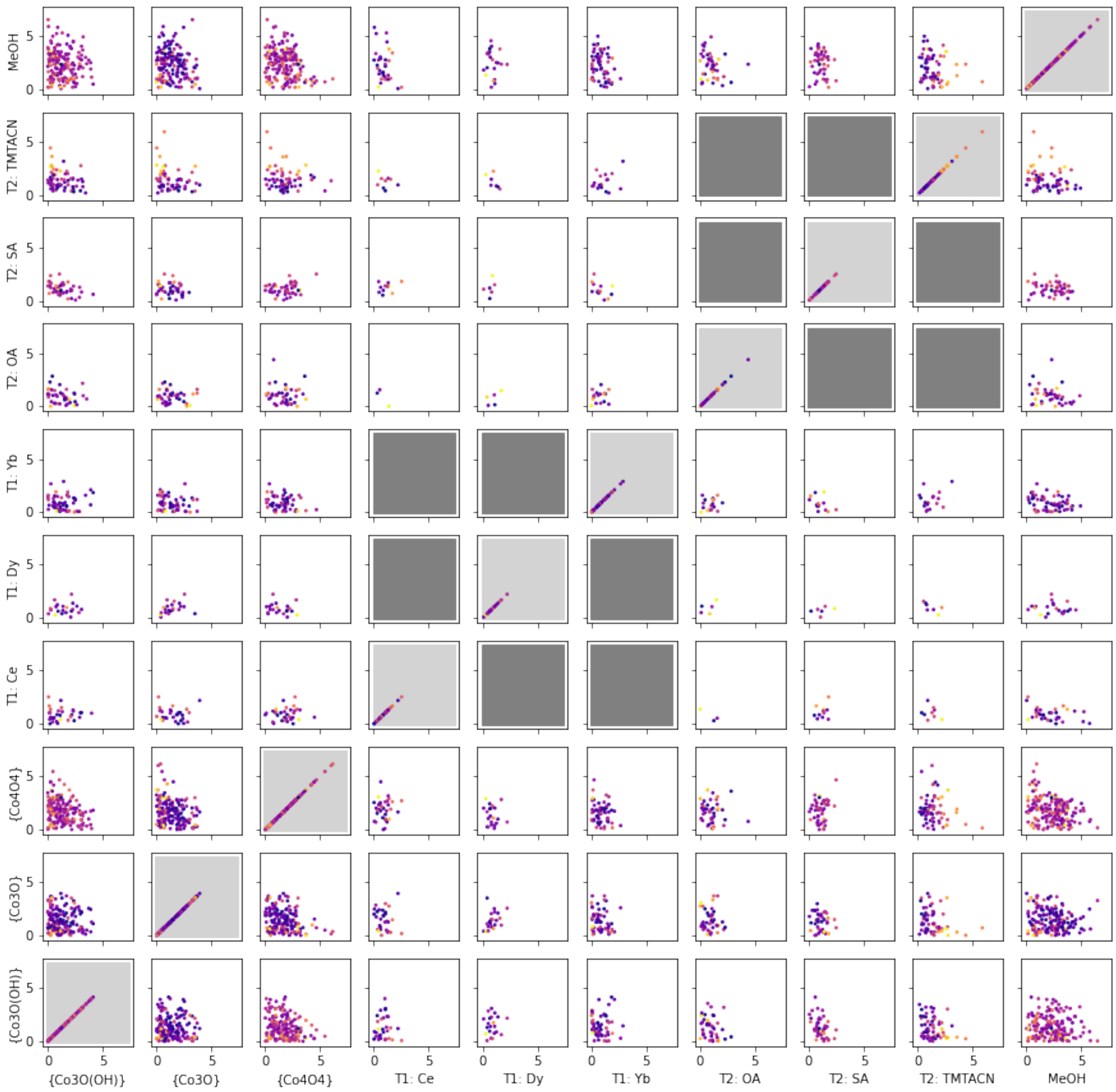

***Figure S74**: Matrix scatter plot of each reagent/solvent for the Common Component Exploration. Points are coloured based on AMF Score, with dark purple indicating the lowest values and yellow indicating the highest. Light grey background indicates the variable plotted against itself – giving an idea of the spread of data points between none of that reagent (0 mL) and only that reagent (7.5 mL). Dark grey background indicates that a potential combination of two variables is not possible as only one template may be chosen from each of the two sets.*

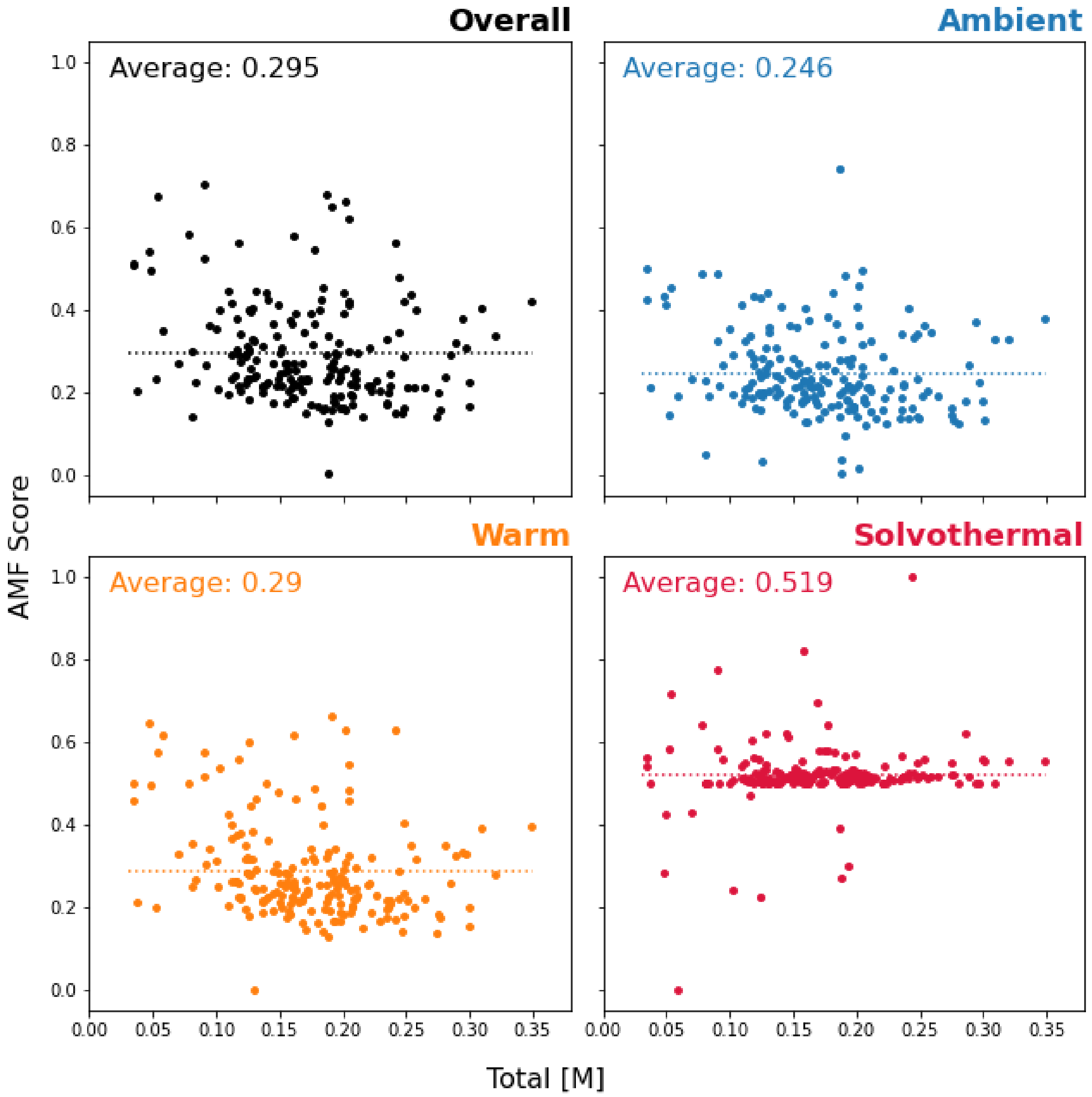

Figure S75: *Plots of Common Component AMF score after Run 4 (overall average across three heating conditions, and score for each condition individually). For each plot, the average AMF value is plotted as a dashed line, and stated in the top left corner.*

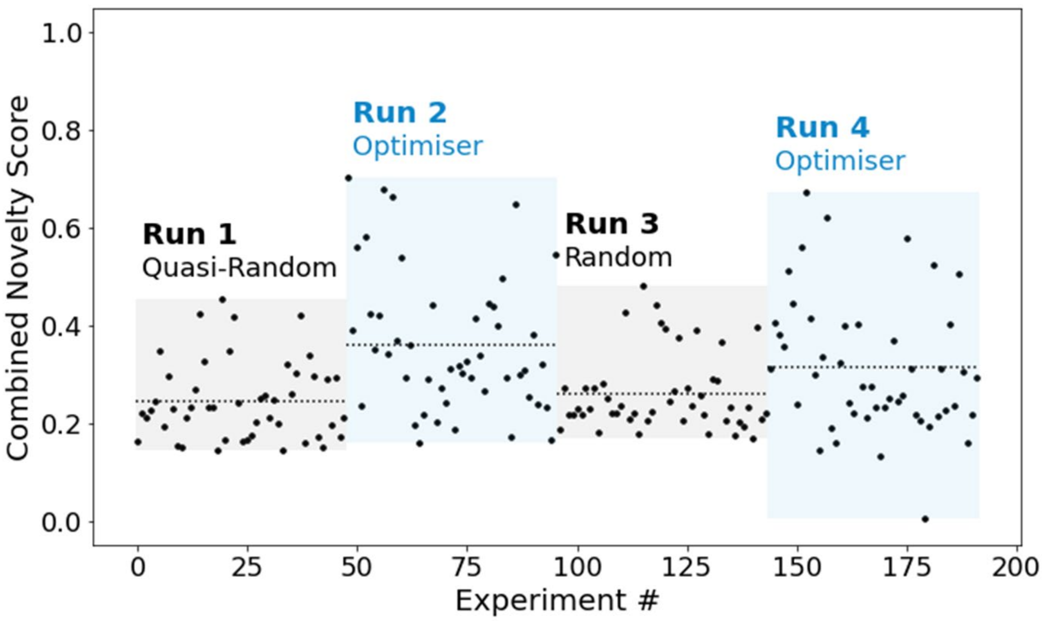

***Figure S76**: Chart indicating the Mapping Function, MF score for each set of reaction conditions probed in the Common Component exploration. Each iteration has 48 data points. Colored bars are used to indicate the range of MF scores obtained, and group the data points by iteration. Text indicates the mode of exploration for that iteration, and grey dashed lines indicate the average MF value for each iteration. MF values are reassessed after each iteration, with the values shown coming from the final assessment, following the fourth iteration. Results are feature scaled within each exploration, so absolute MF values are not comparable with the Isostructural exploration.*

6.9.3 List of Unique Peaks

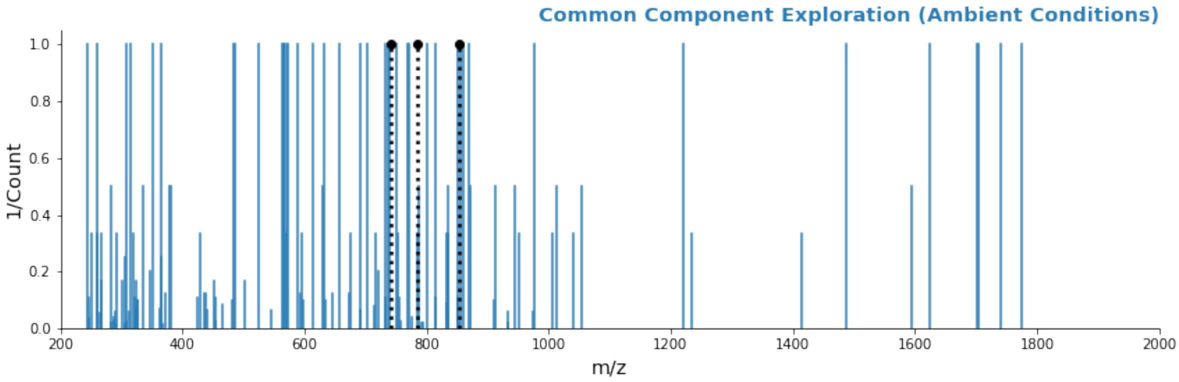

Figure S77: *Chart showing each unique peak identified in the Common Component Exploration at room temperature, with peak height plotted as the inverse of the number of times a given peak appears across the dataset of all 192 experiments ('1/Count'). Black dotted lines indicate the $[M]^+$ or $[M+H]^+$ ions of the cluster starting materials to give context for the observed new peaks.*

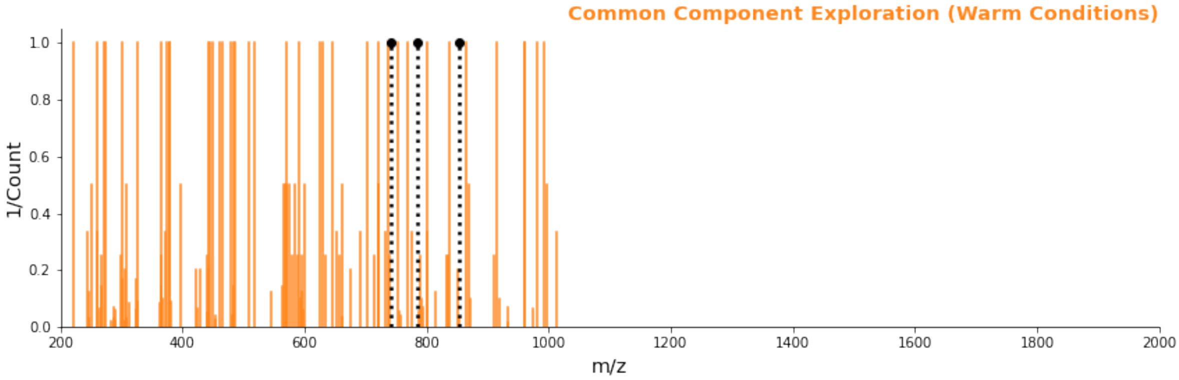

Figure S78: *Chart showing each unique peak identified in the Common Component Exploration under the warm conditions (50 °C, 30 min), with peak height plotted as the inverse of the number of times a given peak appears across the dataset of all 192 experiments ('1/Count'). Black dotted lines indicate the $[M]^+$ or $[M+H]^+$ ions of the cluster starting materials to give context for the observed new peaks.*

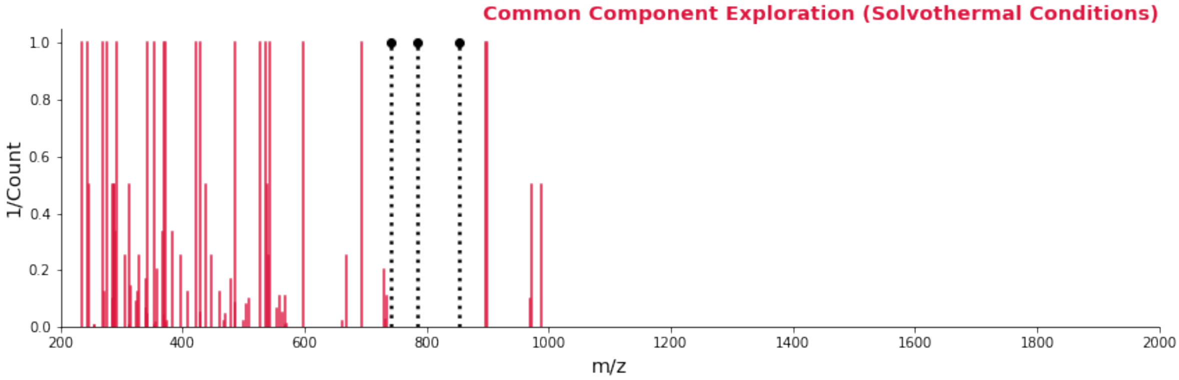

Figure S79: *Spectrum showing each unique peak identified in the Common Component Exploration under the solvothermal conditions (90 °C, 4 days), with peak height plotted as the inverse of the number of times a given peak appears across the dataset of all 192 experiments ('1/Count'). Black dotted lines indicate the $[M]^+$ or $[M+H]^+$ ions of the cluster starting materials to give context for the observed new peaks.*

***Table S7**: Unique Peaks in the Common Component Exploration – m/z values of peaks which do not appear in the corresponding cluster standard MS spectra, as digitally identified from mass spectrometry data during calculation of the mapping function. Note that, despite HRMS being used, the algorithm interprets rounded versions of the peak m/z values, to ensure all common peaks are identified. As such, data is reported to this level of precision.*

**Exp #**	**Room Temperature**	**Warm (50 °C, 30 min)**	**Solvothermal (90 °C, 4 days)**
0	[282.0, 758.0, 814.0]	[282.0, 675.0, 758.0, 814.0]	[255.0, 256.0, 313.0, 354.0, 371.0, 468.0, 500.0, 569.0, 570.0]
1	[282.0, 289.0, 312.0, 453.0]	[282.0, 289.0, 312.0, 453.0]	[255.0, 256.0, 313.0, 324.0, 354.0, 371.0, 569.0, 570.0]
2	[288.0, 306.0, 362.0, 758.0]	[306.0, 307.0, 758.0]	[255.0, 256.0, 313.0, 354.0, 355.0, 371.0, 373.0, 468.0, 500.0, 563.0, 569.0, 570.0, 662.0, 733.0]
3	[675.0, 758.0, 814.0, 933.0]	[675.0, 758.0, 814.0, 933.0]	[255.0, 256.0, 313.0, 354.0, 369.0, 371.0, 373.0, 468.0, 470.0, 486.0, 500.0, 558.0, 569.0, 570.0, 731.0, 733.0, 734.0]
4	[282.0, 289.0, 312.0, 453.0, 597.0, 755.0, 870.0]	[282.0, 289.0, 312.0, 453.0, 597.0, 755.0, 870.0]	[255.0, 256.0, 313.0, 343.0, 354.0, 355.0, 371.0, 373.0, 468.0, 500.0, 569.0, 570.0]
5	[246.0, 288.0, 289.0, 306.0, 307.0, 440.0, 454.0, 482.0, 485.0, 758.0]	[246.0, 288.0, 289.0, 306.0, 307.0, 454.0, 482.0, 758.0, 933.0]	[255.0, 256.0, 313.0, 343.0, 354.0, 355.0, 371.0, 468.0, 500.0, 563.0, 568.0, 569.0, 570.0, 662.0]
6	[282.0, 289.0, 306.0, 453.0, 454.0, 482.0, 933.0]	[282.0, 289.0, 306.0, 454.0, 482.0, 933.0]	[255.0, 256.0, 313.0, 343.0, 354.0, 355.0, 371.0, 468.0, 500.0, 509.0, 563.0, 568.0, 569.0, 570.0, 662.0]
7	[252.0, 261.0, 268.0, 282.0, 312.0, 362.0, 453.0, 933.0]	[252.0, 261.0, 268.0, 282.0, 312.0, 453.0, 854.0, 933.0]	[255.0, 256.0, 313.0, 324.0, 354.0, 355.0, 371.0, 509.0, 541.0, 569.0, 570.0]
8	[814.0, 851.0, 854.0, 933.0]	[814.0, 832.0, 854.0, 911.0, 933.0]	[255.0, 256.0, 313.0, 343.0, 354.0, 371.0, 468.0, 500.0, 569.0, 570.0]
9	[282.0, 453.0, 758.0, 933.0]	[933.0]	[255.0, 256.0, 313.0, 354.0, 371.0, 468.0, 486.0, 500.0, 569.0, 570.0]
10	[282.0, 453.0]	[282.0, 453.0]	[255.0, 256.0, 313.0, 354.0, 371.0, 468.0, 500.0, 509.0, 541.0, 569.0, 570.0]
11	[282.0, 362.0, 453.0, 755.0, 758.0]	[282.0, 453.0, 758.0]	[255.0, 256.0, 313.0, 343.0, 354.0, 371.0, 373.0, 468.0, 486.0, 500.0, 569.0, 570.0, 733.0]
12	[282.0, 453.0, 800.0]	[282.0, 453.0, 800.0]	[255.0, 256.0, 313.0, 354.0, 371.0, 468.0, 500.0, 569.0, 570.0]
13	[246.0, 287.0, 288.0, 289.0, 306.0, 307.0, 424.0, 454.0, 482.0]	[246.0, 288.0, 289.0, 306.0, 307.0, 423.0, 424.0, 454.0, 482.0]	[255.0, 256.0, 313.0, 354.0, 355.0, 371.0, 563.0, 568.0, 569.0, 570.0, 662.0]
14	[289.0, 306.0, 758.0]	[289.0, 306.0, 758.0, 933.0]	[255.0, 256.0, 313.0, 354.0, 369.0, 468.0, 470.0, 486.0, 500.0, 505.0, 569.0, 669.0, 733.0, 734.0]
15	[758.0]	[289.0, 758.0]	[255.0, 256.0, 313.0, 354.0, 371.0, 509.0, 541.0, 569.0, 570.0]
16	[252.0, 261.0, 268.0, 282.0, 362.0, 453.0]	[261.0, 268.0, 282.0, 289.0, 453.0]	[255.0, 256.0, 313.0, 324.0, 354.0, 355.0, 371.0, 509.0, 569.0, 570.0]
17	[246.0, 288.0, 289.0, 306.0, 307.0, 454.0, 482.0]	[246.0, 288.0, 289.0, 306.0, 307.0, 454.0, 482.0]	[255.0, 256.0, 313.0, 354.0, 355.0, 371.0, 509.0, 563.0, 568.0, 569.0, 570.0, 662.0]

18	[282.0, 453.0, 758.0]	[282.0, 758.0]	[255.0, 256.0, 313.0, 343.0, 354.0, 371.0, 373.0, 468.0, 486.0, 500.0, 569.0, 570.0, 733.0]
19	[252.0, 259.0, 261.0, 268.0, 795.0, 854.0, 933.0]	[252.0, 259.0, 261.0, 268.0, 854.0, 933.0]	[255.0, 256.0, 313.0, 354.0, 355.0, 371.0, 509.0, 541.0, 569.0, 570.0]
20	[282.0, 306.0, 758.0]	[306.0, 758.0]	[255.0, 256.0, 313.0, 343.0, 354.0, 355.0, 369.0, 371.0, 373.0, 468.0, 470.0, 486.0, 500.0, 558.0, 569.0, 570.0, 731.0, 733.0]
21	[814.0, 832.0, 851.0, 911.0]	[675.0, 814.0, 832.0, 851.0, 911.0]	[255.0, 256.0, 313.0, 342.0, 354.0, 355.0, 569.0]
22	[246.0, 287.0, 288.0, 289.0, 290.0, 306.0, 307.0, 366.0, 424.0, 440.0, 758.0]	[246.0, 274.0, 287.0, 288.0, 289.0, 290.0, 306.0, 307.0, 366.0, 423.0, 440.0, 454.0, 482.0]	[255.0, 256.0, 313.0, 354.0, 355.0, 569.0, 662.0, 970.0, 971.0]
23	[362.0, 371.0, 378.0, 758.0, 933.0, 934.0, 974.0, 1012.0]	[282.0, 362.0, 545.0, 933.0, 934.0, 974.0]	[255.0, 256.0, 313.0, 343.0, 354.0, 355.0, 371.0, 468.0, 500.0, 509.0, 569.0, 570.0]
24	[282.0, 306.0, 362.0, 371.0, 453.0, 933.0]	[282.0, 306.0, 362.0, 933.0]	[255.0, 256.0, 313.0, 343.0, 354.0, 355.0, 371.0, 468.0, 500.0, 569.0, 570.0, 662.0, 733.0]
25	[453.0, 854.0, 933.0]	[282.0, 453.0, 854.0, 933.0]	[255.0, 256.0, 313.0, 343.0, 354.0, 371.0, 468.0, 500.0, 569.0, 570.0]
26	[282.0, 362.0, 453.0, 545.0, 933.0, 934.0, 974.0, 1007.0]	[282.0, 362.0, 453.0, 545.0, 933.0, 934.0, 974.0]	[255.0, 256.0, 313.0, 343.0, 354.0, 371.0, 468.0, 500.0, 569.0, 570.0]
27	[362.0, 758.0, 933.0, 1007.0]	[362.0, 758.0, 933.0]	[255.0, 256.0, 313.0, 354.0, 369.0, 371.0, 468.0, 486.0, 500.0, 558.0, 569.0, 570.0, 731.0, 733.0, 734.0]
28	[282.0, 453.0, 933.0]	[282.0, 453.0, 933.0]	[255.0, 256.0, 313.0, 354.0, 371.0, 569.0, 570.0]
29	[246.0, 287.0, 288.0, 289.0, 306.0, 307.0, 440.0, 454.0, 482.0]	[246.0, 288.0, 289.0, 306.0, 307.0, 423.0, 440.0, 454.0, 482.0]	[255.0, 256.0, 313.0, 354.0, 355.0, 371.0, 468.0, 500.0, 563.0, 568.0, 569.0, 570.0, 662.0]
30	[362.0, 371.0, 758.0, 933.0]	[268.0, 758.0, 933.0]	[255.0, 256.0, 313.0, 354.0, 371.0, 468.0, 500.0, 509.0, 569.0, 570.0]
31	[282.0, 289.0, 306.0, 312.0, 322.0, 352.0, 453.0, 597.0, 755.0]	[282.0, 289.0, 312.0, 453.0, 597.0]	[255.0, 256.0, 313.0, 324.0, 343.0, 354.0, 355.0, 371.0, 468.0, 500.0, 569.0, 570.0]
32	[362.0, 371.0, 545.0, 758.0, 933.0]	[362.0, 545.0, 933.0, 934.0, 974.0]	[255.0, 256.0, 313.0, 343.0, 354.0, 371.0, 468.0, 500.0, 569.0, 570.0]
33	[282.0, 362.0, 371.0, 453.0, 933.0]	[282.0, 362.0, 453.0, 933.0]	[255.0, 256.0, 313.0, 343.0, 354.0, 371.0, 468.0, 500.0, 569.0, 570.0]
34	[691.0, 713.0, 792.0, 814.0, 832.0, 851.0, 854.0, 911.0]	[453.0, 814.0, 832.0, 851.0, 854.0, 911.0, 933.0]	[255.0, 256.0, 313.0, 354.0, 369.0, 371.0, 468.0, 486.0, 500.0, 569.0, 733.0]
35	[246.0, 282.0, 287.0, 288.0, 289.0, 290.0, 306.0, 307.0, 424.0, 440.0, 454.0, 482.0, 933.0]	[246.0, 288.0, 289.0, 306.0, 307.0, 424.0, 440.0, 454.0, 482.0, 933.0]	[255.0, 256.0, 313.0, 343.0, 354.0, 355.0, 468.0, 500.0, 563.0, 568.0, 569.0, 570.0, 662.0]
36	[282.0, 289.0, 453.0, 597.0, 755.0, 758.0]	[282.0, 289.0, 453.0, 484.0, 563.0, 653.0, 755.0]	[255.0, 256.0, 313.0, 324.0, 343.0, 354.0, 371.0, 468.0, 500.0, 569.0, 570.0]
37	[362.0, 371.0, 378.0, 758.0, 854.0, 933.0, 934.0, 974.0, 1012.0]	[362.0, 371.0, 378.0, 758.0, 854.0, 933.0, 934.0, 974.0, 1012.0]	[255.0, 256.0, 313.0, 343.0, 354.0, 355.0, 371.0, 373.0, 468.0, 500.0, 569.0, 570.0]
38	[282.0, 289.0, 453.0, 597.0, 755.0]	[282.0, 289.0, 453.0, 597.0, 755.0]	[255.0, 256.0, 313.0, 343.0, 354.0, 371.0, 373.0, 468.0, 486.0, 500.0, 569.0, 570.0, 733.0]

39	[289.0, 306.0, 307.0, 454.0, 482.0, 758.0, 933.0]	[289.0, 306.0, 307.0, 454.0, 482.0, 758.0, 933.0]	[255.0, 256.0, 313.0, 354.0, 369.0, 468.0, 470.0, 486.0, 500.0, 558.0, 569.0, 570.0, 662.0, 731.0, 733.0, 734.0]
40	[282.0, 634.0, 713.0, 795.0, 854.0]	[282.0, 634.0, 713.0, 795.0, 837.0, 854.0]	[255.0, 256.0, 313.0, 354.0, 371.0, 569.0, 570.0]
41	[282.0, 362.0, 371.0, 545.0, 933.0, 934.0, 974.0]	[282.0, 362.0, 371.0, 453.0, 545.0, 933.0, 934.0, 974.0]	[255.0, 256.0, 313.0, 343.0, 354.0, 355.0, 371.0, 468.0, 500.0, 569.0, 570.0]
42	[758.0, 933.0, 1007.0]	[545.0, 758.0, 933.0, 934.0, 974.0]	[255.0, 256.0, 313.0, 343.0, 354.0, 371.0, 468.0, 486.0, 500.0, 569.0, 570.0]
43	[246.0, 282.0, 287.0, 288.0, 289.0, 306.0, 307.0, 364.0, 424.0, 440.0, 454.0, 482.0, 933.0]	[246.0, 282.0, 287.0, 288.0, 289.0, 306.0, 307.0, 364.0, 423.0, 424.0, 440.0, 454.0, 482.0, 933.0]	[255.0, 256.0, 313.0, 354.0, 355.0, 398.0, 468.0, 500.0, 509.0, 563.0, 568.0, 569.0, 570.0, 662.0]
44	[282.0, 289.0, 306.0, 324.0, 453.0, 597.0, 755.0]	[282.0, 289.0, 289.0, 306.0, 324.0, 453.0, 755.0]	[255.0, 256.0, 313.0, 343.0, 354.0, 355.0, 371.0, 468.0, 500.0, 563.0, 568.0, 569.0, 570.0, 662.0]
45	[758.0, 933.0]	[443.0, 758.0, 788.0, 933.0]	[255.0, 256.0, 313.0, 354.0, 369.0, 371.0, 468.0, 486.0, 500.0, 544.0, 558.0, 569.0, 570.0, 731.0, 733.0]
46	[282.0, 362.0, 371.0, 758.0, 933.0]	[282.0, 362.0, 371.0, 758.0, 933.0]	[255.0, 256.0, 313.0, 354.0, 355.0, 371.0, 373.0, 468.0, 500.0, 569.0, 570.0]
47	[675.0, 758.0, 814.0, 933.0]	[675.0, 758.0, 814.0, 933.0, 974.0]	[255.0, 256.0, 313.0, 343.0, 354.0, 371.0, 468.0, 500.0, 569.0, 570.0]
48	[]	[265.0, 267.0, 367.0, 571.0, 575.0, 585.0, 589.0, 691.0, 713.0, 732.0, 795.0, 834.0, 872.0]	[245.0, 255.0, 256.0, 286.0, 287.0, 289.0, 306.0, 313.0, 327.0, 328.0, 340.0, 354.0, 355.0, 355.0, 359.0, 539.0, 568.0, 569.0, 662.0]
49	[524.0, 545.0, 566.0, 597.0, 645.0, 658.0, 776.0, 792.0, 854.0]	[282.0, 453.0, 634.0, 662.0, 713.0, 755.0, 792.0, 795.0, 834.0, 854.0, 872.0]	[255.0, 256.0, 306.0, 313.0, 354.0, 355.0, 371.0, 373.0, 569.0, 570.0]
50	[288.0, 306.0, 307.0, 776.0, 792.0, 854.0]	[265.0, 267.0, 302.0, 324.0, 565.0, 569.0, 579.0, 658.0]	[255.0, 256.0, 313.0, 354.0, 355.0, 479.0, 662.0, 970.0]
51	[302.0, 306.0, 324.0, 326.0, 933.0]	[265.0, 267.0, 302.0, 324.0, 565.0, 569.0, 579.0, 658.0]	[255.0, 272.0, 286.0, 313.0, 314.0, 327.0, 328.0, 340.0, 354.0, 355.0, 359.0, 369.0, 373.0]
52	[]	[]	[255.0, 256.0, 272.0, 286.0, 313.0, 314.0, 327.0, 340.0, 341.0, 354.0, 355.0, 369.0, 693.0]
53	[246.0, 265.0, 288.0, 289.0, 306.0, 307.0, 366.0]	[246.0, 247.0, 288.0, 289.0, 290.0, 305.0, 306.0, 307.0, 366.0, 380.0, 424.0, 440.0, 441.0, 454.0, 482.0, 519.0, 594.0, 788.0]	[255.0, 256.0, 313.0, 354.0, 355.0, 367.0, 662.0, 970.0, 971.0]
54	[288.0, 289.0, 306.0, 307.0, 758.0]	[246.0, 265.0, 282.0, 282.0, 289.0, 289.0, 302.0, 306.0, 324.0, 325.0, 326.0, 450.0, 466.0, 480.0, 484.0, 545.0, 563.0, 579.0, 625.0, 658.0]	[]
55	[246.0, 288.0, 289.0, 306.0, 307.0, 366.0, 776.0, 792.0, 854.0, 933.0, 934.0, 974.0]	[245.0, 246.0, 287.0, 288.0, 289.0, 290.0, 305.0, 306.0, 307.0, 366.0, 380.0, 424.0, 440.0, 441.0, 454.0, 482.0, 788.0, 854.0, 919.0, 933.0]	[255.0, 256.0, 313.0, 354.0, 355.0, 569.0, 662.0, 970.0]
56	[306.0, 367.0, 703.0, 713.0, 733.0, 776.0, 792.0, 795.0, 832.0, 833.0, 850.0, 854.0, 855.0, 911.0, 912.0,	[306.0, 634.0, 662.0, 691.0, 713.0, 720.0, 788.0, 792.0, 792.0, 834.0, 854.0, 933.0]	[255.0, 256.0, 313.0, 340.0, 354.0, 355.0, 662.0, 970.0]

	933.0, 934.0, 951.0, 974.0, 1624.0, 1703.0, 1704.0]		
57	[288.0, 289.0, 306.0, 307.0, 324.0, 325.0, 326.0, 367.0, 776.0, 792.0, 795.0, 854.0]	[246.0, 265.0, 282.0, 287.0, 288.0, 289.0, 306.0, 307.0, 324.0, 325.0, 326.0, 366.0, 424.0, 795.0, 872.0]	[255.0, 256.0, 313.0, 354.0, 569.0, 570.0]
58	[306.0, 367.0, 545.0, 758.0, 776.0, 788.0, 792.0, 795.0, 854.0, 933.0, 934.0, 951.0, 974.0, 1041.0, 1235.0, 1415.0, 1595.0, 1775.0]	[282.0, 306.0, 453.0, 545.0, 662.0, 691.0, 776.0, 788.0, 789.0, 792.0, 834.0, 854.0, 872.0, 919.0, 933.0, 934.0, 960.0, 974.0, 982.0, 998.0, 1012.0]	[255.0, 256.0, 313.0, 354.0, 371.0, 555.0, 569.0, 570.0]
59	[367.0, 776.0, 792.0, 795.0, 832.0, 833.0, 854.0, 911.0, 933.0, 934.0, 974.0]	[662.0, 788.0, 832.0, 854.0, 911.0, 933.0]	[255.0, 256.0, 313.0, 354.0, 369.0, 468.0, 470.0, 500.0, 527.0, 539.0, 558.0, 569.0, 570.0, 733.0]
60	[]	[301.0, 312.0, 367.0, 453.0, 645.0, 769.0, 851.0, 865.0]	[]
61	[246.0, 288.0, 289.0, 306.0, 307.0, 366.0, 792.0, 854.0]	[246.0, 287.0, 288.0, 289.0, 306.0, 307.0, 366.0, 380.0, 424.0, 440.0, 441.0, 454.0, 482.0, 662.0]	[255.0, 256.0, 313.0, 354.0, 355.0, 367.0, 408.0, 438.0, 479.0, 563.0, 569.0, 662.0, 970.0]
62	[288.0, 289.0, 306.0, 307.0, 324.0, 326.0, 758.0]	[246.0, 282.0, 288.0, 289.0, 289.0, 306.0, 307.0, 324.0, 326.0, 364.0, 380.0, 440.0, 597.0, 662.0, 755.0]	[255.0, 256.0, 313.0, 340.0, 354.0, 355.0, 367.0, 408.0, 438.0, 479.0, 662.0]
63	[282.0, 367.0, 367.0, 792.0, 854.0]	[282.0, 282.0, 312.0, 367.0, 453.0, 579.0, 597.0, 658.0, 662.0, 755.0]	[255.0, 256.0, 313.0, 324.0, 354.0, 355.0, 371.0, 569.0]
64	[282.0, 289.0, 312.0, 367.0, 453.0, 466.0, 792.0, 795.0, 854.0, 933.0]	[282.0, 312.0, 453.0, 597.0, 662.0, 755.0, 792.0, 854.0]	[255.0, 256.0, 313.0, 354.0, 355.0, 371.0, 373.0, 555.0, 569.0, 570.0]
65	[282.0, 289.0, 312.0, 322.0, 367.0, 453.0, 466.0, 545.0, 792.0, 795.0, 854.0, 872.0, 933.0]	[282.0, 312.0, 453.0, 597.0, 662.0, 755.0, 792.0, 854.0]	[255.0, 256.0, 313.0, 354.0, 371.0, 373.0, 569.0, 570.0]
66	[282.0, 282.0, 283.0, 289.0, 312.0, 320.0, 322.0, 453.0, 466.0, 503.0, 545.0, 597.0, 634.0, 674.0, 713.0, 755.0, 758.0, 792.0]	[453.0, 597.0, 662.0, 755.0]	[255.0, 256.0, 313.0, 354.0, 371.0, 373.0, 569.0, 570.0, 733.0]
67	[282.0, 289.0, 312.0, 322.0, 367.0, 436.0, 437.0, 453.0, 466.0, 503.0, 545.0, 674.0, 758.0, 776.0, 792.0, 854.0, 933.0, 934.0, 974.0, 976.0, 1041.0, 1055.0, 1221.0, 1235.0, 1415.0]	[282.0, 312.0, 442.0, 453.0, 662.0, 788.0, 789.0, 854.0, 919.0, 933.0, 998.0]	[255.0, 256.0, 313.0, 354.0, 371.0, 555.0, 569.0, 570.0]
68	[367.0, 758.0, 776.0, 792.0, 854.0, 933.0]	[282.0, 453.0, 662.0, 788.0]	[255.0, 256.0, 313.0, 354.0, 371.0, 555.0, 569.0, 570.0]
69	[282.0, 367.0, 436.0, 437.0, 451.0, 453.0, 545.0, 776.0, 792.0, 795.0, 854.0, 933.0, 934.0, 974.0]	[282.0, 453.0, 662.0, 788.0, 854.0, 872.0, 933.0]	[255.0, 256.0, 313.0, 354.0, 371.0, 373.0, 555.0, 569.0, 570.0]
70	[282.0, 289.0, 312.0, 322.0, 367.0, 453.0, 466.0, 503.0, 545.0, 674.0, 753.0]	[282.0, 453.0, 597.0, 662.0, 755.0]	[255.0, 256.0, 313.0, 354.0, 355.0, 371.0, 555.0, 569.0, 570.0]
71	[367.0, 367.0, 776.0, 792.0, 795.0, 832.0, 854.0, 911.0, 933.0, 1741.0]	[282.0, 453.0, 662.0, 854.0]	[255.0, 256.0, 313.0, 354.0, 398.0, 555.0, 569.0, 570.0]
72	[282.0, 367.0, 758.0, 792.0]	[282.0, 312.0, 453.0, 597.0, 662.0, 755.0]	[255.0, 256.0, 313.0, 354.0, 355.0, 371.0, 555.0, 569.0, 570.0]
73	[367.0, 776.0, 792.0, 795.0, 832.0, 854.0, 933.0]	[282.0, 453.0, 662.0, 776.0, 792.0, 795.0, 854.0, 872.0]	[255.0, 256.0, 313.0, 354.0, 355.0, 398.0, 555.0, 569.0, 570.0]
74	[367.0, 367.0, 758.0, 792.0, 854.0]	[597.0, 755.0, 854.0]	[255.0, 256.0, 313.0, 354.0, 355.0, 371.0, 555.0, 569.0, 570.0]
75	[367.0, 436.0, 437.0, 451.0, 758.0, 776.0, 792.0, 854.0, 933.0, 934.0, 974.0]	[442.0, 453.0, 758.0, 788.0, 789.0, 854.0, 919.0, 933.0]	[255.0, 256.0, 313.0, 354.0, 429.0, 447.0, 470.0, 505.0, 569.0, 570.0, 669.0, 733.0, 734.0]

76	[282.0, 282.0, 283.0, 288.0, 289.0, 306.0, 307.0, 312.0, 320.0, 322.0, 436.0, 437.0, 451.0, 453.0, 466.0, 503.0, 545.0, 674.0, 776.0, 792.0, 854.0, 933.0, 934.0, 944.0, 974.0]	[288.0, 289.0, 306.0, 307.0, 380.0, 424.0, 440.0, 453.0, 454.0, 482.0, 662.0, 788.0, 919.0, 933.0]	[255.0, 256.0, 313.0, 354.0, 470.0, 569.0, 570.0, 662.0]
77	[282.0, 288.0, 289.0, 306.0, 307.0, 312.0, 322.0, 324.0, 326.0, 367.0, 453.0, 466.0, 482.0, 545.0, 776.0, 792.0, 795.0, 854.0, 933.0]	[221.0, 246.0, 288.0, 289.0, 290.0, 306.0, 307.0, 324.0, 364.0, 365.0, 366.0, 380.0, 424.0, 440.0, 662.0, 872.0]	[255.0, 256.0, 313.0, 354.0, 355.0, 408.0, 662.0]
78	[755.0, 758.0]	[282.0, 597.0, 755.0, 758.0]	[255.0, 256.0, 313.0, 354.0, 355.0, 371.0, 555.0, 569.0, 570.0]
79	[]	[282.0, 453.0, 661.0, 662.0, 721.0, 800.0]	[]
80	[367.0, 367.0, 436.0, 437.0, 451.0, 776.0, 792.0, 795.0, 854.0, 933.0, 934.0, 974.0]	[442.0, 788.0, 854.0, 919.0, 933.0, 934.0, 961.0, 974.0, 992.0, 1012.0]	[255.0, 256.0, 313.0, 354.0, 569.0, 570.0]
81	[282.0, 367.0, 436.0, 437.0, 451.0, 453.0, 758.0, 776.0, 788.0, 792.0, 795.0, 854.0, 872.0, 933.0, 934.0, 951.0, 974.0]	[282.0, 312.0, 442.0, 453.0, 545.0, 662.0, 720.0, 788.0, 789.0, 792.0, 795.0, 854.0, 872.0, 919.0, 933.0, 934.0, 974.0]	[255.0, 256.0, 313.0, 354.0, 429.0, 447.0, 470.0, 505.0, 569.0, 669.0, 733.0]
82	[758.0]	[585.0, 661.0, 662.0, 675.0, 703.0, 721.0, 722.0, 736.0, 753.0, 800.0, 801.0, 814.0]	[255.0, 256.0, 313.0, 354.0, 398.0, 555.0, 569.0, 570.0]
83	[484.0, 563.0]	[271.0, 282.0, 282.0, 289.0, 312.0, 375.0, 453.0, 484.0, 563.0]	[]
84	[367.0, 436.0, 437.0, 758.0, 792.0, 854.0, 933.0, 1041.0]	[662.0, 758.0, 788.0, 854.0, 919.0, 933.0]	[255.0, 256.0, 313.0, 354.0, 429.0, 470.0, 505.0, 569.0, 570.0, 733.0]
85	[282.0, 367.0, 453.0, 758.0, 792.0, 795.0, 854.0, 933.0]	[282.0, 453.0, 597.0, 755.0, 795.0]	[255.0, 256.0, 313.0, 354.0, 355.0, 371.0, 569.0, 570.0]
86	[776.0, 792.0, 854.0]	[246.0, 247.0, 287.0, 288.0, 289.0, 290.0, 306.0, 307.0, 570.0, 594.0, 595.0, 596.0, 630.0]	[255.0, 256.0, 272.0, 286.0, 313.0, 340.0, 354.0, 355.0, 569.0, 570.0, 662.0, 988.0]
87	[265.0, 282.0, 287.0, 288.0, 289.0, 306.0, 312.0, 324.0, 325.0, 326.0, 367.0, 453.0, 466.0, 776.0, 792.0, 795.0, 854.0]	[246.0, 265.0, 282.0, 282.0, 288.0, 289.0, 306.0, 307.0, 324.0, 325.0, 326.0, 380.0, 440.0, 597.0, 662.0, 755.0]	[255.0, 256.0, 313.0, 354.0, 355.0, 662.0]
88	[758.0, 933.0, 934.0, 974.0, 1055.0, 1235.0, 1415.0, 1595.0]	[282.0, 662.0, 758.0, 788.0, 933.0]	[255.0, 256.0, 313.0, 354.0, 355.0, 371.0, 555.0, 569.0, 570.0]
89	[282.0, 289.0, 312.0, 322.0, 453.0, 466.0, 503.0, 545.0, 674.0, 753.0, 933.0]	[442.0, 453.0, 662.0, 933.0]	[255.0, 256.0, 313.0, 354.0, 371.0, 555.0, 569.0, 570.0]
90	[265.0, 282.0, 282.0, 289.0, 289.0, 302.0, 312.0, 320.0, 322.0, 324.0, 325.0, 326.0, 327.0, 367.0, 453.0, 466.0, 503.0, 545.0, 597.0, 674.0, 753.0, 755.0, 792.0, 854.0]	[246.0, 265.0, 282.0, 282.0, 288.0, 289.0, 302.0, 306.0, 307.0, 324.0, 325.0, 326.0, 440.0, 453.0, 597.0, 662.0, 755.0]	[255.0, 256.0, 313.0, 340.0, 354.0, 355.0, 569.0, 662.0, 970.0]
91	[282.0, 288.0, 289.0, 306.0, 307.0, 312.0, 440.0, 453.0, 466.0, 545.0, 674.0, 758.0, 792.0, 854.0, 933.0]	[246.0, 288.0, 289.0, 306.0, 307.0, 380.0, 397.0, 440.0, 454.0, 482.0, 758.0, 788.0, 919.0, 933.0]	[255.0, 256.0, 313.0, 354.0, 470.0, 563.0, 569.0, 570.0, 662.0, 733.0]
92	[282.0, 288.0, 289.0, 306.0, 307.0, 312.0, 436.0, 437.0, 451.0, 453.0, 454.0, 466.0, 482.0, 545.0, 739.0, 758.0, 776.0, 792.0, 854.0, 933.0, 934.0, 944.0, 974.0]	[288.0, 289.0, 306.0, 307.0, 380.0, 440.0, 454.0, 482.0, 662.0, 788.0, 919.0, 933.0]	[255.0, 256.0, 313.0, 354.0, 470.0, 569.0, 570.0, 662.0]
93	[282.0, 453.0, 674.0, 792.0, 795.0, 854.0, 933.0, 934.0, 974.0]	[453.0, 662.0, 788.0, 854.0, 933.0]	[255.0, 256.0, 313.0, 354.0, 555.0, 569.0, 570.0]

94	[282.0, 453.0, 758.0]	[597.0, 662.0, 755.0, 758.0, 788.0]	[255.0, 256.0, 313.0, 354.0, 355.0, 371.0, 569.0, 570.0]
95	[282.0, 288.0, 289.0, 306.0, 307.0, 312.0, 315.0, 453.0, 720.0, 776.0, 792.0, 854.0, 861.0, 933.0]	[245.0, 246.0, 287.0, 288.0, 289.0, 290.0, 305.0, 306.0, 307.0, 364.0, 366.0, 380.0, 424.0, 440.0, 441.0]	[255.0, 256.0, 313.0, 340.0, 354.0, 355.0, 408.0, 479.0, 569.0, 662.0, 970.0, 988.0]
96	[]	[854.0, 933.0, 934.0, 974.0]	[255.0, 256.0, 313.0, 354.0, 371.0, 373.0, 569.0]
97	[]	[854.0, 933.0]	[255.0, 256.0, 312.0, 313.0, 354.0, 370.0, 371.0, 373.0, 569.0]
98	[367.0, 367.0, 795.0]	[870.0]	[255.0, 256.0, 313.0, 354.0, 371.0, 373.0, 429.0, 505.0, 733.0]
99	[367.0, 795.0]	[]	[255.0, 256.0, 313.0, 354.0, 371.0, 373.0, 569.0, 570.0, 733.0]
100	[854.0]	[814.0, 933.0]	[255.0, 256.0, 313.0, 354.0, 371.0, 429.0, 461.0, 505.0, 569.0, 733.0, 734.0]
101	[854.0]	[933.0]	[255.0, 256.0, 313.0, 354.0, 371.0, 373.0, 569.0]
102	[]	[854.0, 933.0, 934.0, 974.0]	[255.0, 256.0, 313.0, 354.0, 371.0, 373.0, 429.0, 461.0, 505.0, 569.0, 570.0, 733.0]
103	[795.0, 854.0]	[795.0, 854.0, 933.0, 934.0, 974.0]	[255.0, 256.0, 313.0, 354.0, 371.0, 373.0, 429.0, 461.0, 569.0, 570.0, 733.0]
104	[854.0]	[362.0, 854.0, 914.0, 933.0]	[]
105	[]	[933.0, 934.0, 974.0]	[255.0, 256.0, 313.0, 354.0, 371.0, 373.0, 429.0, 461.0, 505.0, 569.0, 733.0]
106	[367.0]	[]	[255.0, 256.0, 313.0, 354.0, 371.0, 373.0, 569.0, 570.0]
107	[785.0]	[]	[255.0, 256.0, 313.0, 354.0, 371.0, 429.0, 447.0, 461.0, 505.0, 569.0, 669.0, 733.0, 734.0]
108	[795.0]	[795.0, 854.0, 872.0, 933.0, 934.0, 974.0]	[255.0, 256.0, 272.0, 313.0, 354.0, 355.0, 468.0, 500.0]
109	[]	[429.0]	[255.0, 256.0, 313.0, 324.0, 354.0, 355.0, 369.0, 373.0, 429.0, 468.0, 500.0]
110	[]	[429.0, 484.0, 563.0, 653.0, 851.0]	[255.0, 256.0, 313.0, 354.0, 371.0, 373.0, 429.0, 461.0, 505.0, 569.0, 733.0]
111	[367.0, 367.0, 691.0, 732.0, 750.0, 795.0]	[367.0, 634.0, 732.0, 792.0, 795.0, 851.0, 854.0, 872.0]	[255.0, 256.0, 313.0, 354.0, 359.0, 371.0, 373.0, 569.0]
112	[]	[]	[]
113	[785.0]	[758.0, 933.0]	[255.0, 256.0, 313.0, 354.0, 355.0, 369.0, 468.0, 470.0, 500.0]
114	[367.0, 795.0]	[]	[255.0, 256.0, 313.0, 354.0, 369.0, 371.0, 373.0, 468.0, 470.0, 500.0, 569.0, 570.0, 733.0]
115	[]	[758.0]	[234.0, 255.0, 256.0, 275.0, 288.0, 291.0, 312.0, 313.0, 353.0, 354.0, 373.0, 429.0, 430.0, 447.0, 505.0, 733.0]
116	[367.0, 795.0]	[]	[255.0, 256.0, 313.0, 354.0, 355.0, 429.0, 733.0]
117	[367.0, 795.0]	[]	[255.0, 256.0, 313.0, 354.0, 371.0, 373.0, 569.0, 570.0]

118	[265.0, 291.0]	[246.0, 265.0, 267.0, 287.0, 288.0, 289.0, 290.0, 306.0, 307.0, 324.0, 326.0, 424.0, 570.0, 575.0, 589.0, 591.0, 594.0]	[255.0, 256.0, 313.0, 354.0, 355.0, 408.0, 563.0, 569.0, 662.0, 970.0]
119	[367.0, 367.0, 795.0]	[327.0, 367.0, 367.0, 484.0, 563.0]	[255.0, 256.0, 313.0, 324.0, 354.0, 355.0, 369.0, 373.0, 384.0, 468.0, 500.0, 536.0]
120	[246.0, 265.0, 287.0, 288.0, 289.0, 290.0, 305.0, 306.0, 307.0, 324.0, 424.0]	[245.0, 246.0, 288.0, 289.0, 290.0, 305.0, 306.0, 307.0, 424.0, 454.0, 482.0]	[255.0, 256.0, 285.0, 313.0, 354.0, 373.0, 384.0, 429.0, 563.0, 569.0, 570.0, 662.0, 733.0]
121	[327.0, 367.0, 367.0, 732.0, 795.0]	[367.0, 367.0, 732.0, 795.0]	[255.0, 256.0, 313.0, 354.0, 371.0, 373.0, 569.0, 570.0]
122	[265.0, 267.0, 287.0, 289.0, 302.0, 306.0, 324.0, 325.0, 326.0]	[265.0, 288.0, 289.0, 306.0, 307.0, 324.0, 326.0, 440.0, 454.0, 482.0]	[255.0, 256.0, 313.0, 354.0, 373.0, 429.0, 563.0, 569.0, 662.0, 733.0]
123	[287.0, 289.0, 306.0, 440.0, 854.0]	[288.0, 289.0, 306.0, 454.0, 482.0, 933.0]	[246.0, 255.0, 256.0, 270.0, 289.0, 306.0, 313.0, 354.0, 355.0, 423.0, 485.0, 569.0]
124	[795.0]	[933.0]	[255.0, 256.0, 285.0, 313.0, 354.0, 371.0, 373.0, 429.0, 470.0, 569.0, 570.0, 733.0]
125	[]	[933.0]	[255.0, 256.0, 313.0, 354.0, 373.0, 429.0, 461.0, 470.0, 505.0, 569.0, 570.0, 733.0, 734.0, 897.0, 899.0]
126	[429.0]	[429.0, 600.0]	[255.0, 256.0, 313.0, 324.0, 354.0, 371.0, 373.0, 569.0, 570.0]
127	[265.0, 267.0, 287.0, 289.0, 306.0, 324.0, 325.0, 326.0]	[246.0, 265.0, 288.0, 289.0, 306.0, 307.0, 308.0, 324.0, 326.0, 445.0, 454.0, 461.0, 482.0]	[255.0, 256.0, 313.0, 354.0, 373.0, 563.0, 569.0, 662.0]
128	[265.0, 291.0, 302.0, 795.0]	[265.0, 267.0, 287.0, 289.0, 306.0, 324.0, 325.0, 326.0, 795.0]	[255.0, 256.0, 313.0, 354.0, 355.0, 468.0, 500.0, 563.0, 569.0, 570.0, 662.0]
129	[265.0, 367.0, 795.0]	[265.0, 289.0, 306.0, 324.0, 325.0, 326.0, 795.0]	[255.0, 256.0, 313.0, 354.0, 371.0, 373.0, 468.0, 500.0, 563.0, 569.0, 570.0, 662.0]
130	[]	[933.0]	[255.0, 256.0, 313.0, 354.0, 371.0, 373.0, 429.0, 569.0, 570.0, 733.0]
131	[367.0, 367.0]	[265.0, 302.0, 367.0, 429.0, 484.0, 563.0, 653.0]	[255.0, 256.0, 313.0, 354.0, 371.0, 373.0, 408.0, 569.0]
132	[795.0, 854.0]	[776.0, 792.0, 795.0, 854.0, 933.0]	[255.0, 256.0, 313.0, 354.0, 371.0, 373.0, 569.0, 570.0]
133	[246.0, 287.0, 288.0, 289.0, 290.0, 306.0, 307.0, 424.0, 440.0, 594.0, 854.0]	[246.0, 287.0, 288.0, 289.0, 290.0, 306.0, 307.0, 424.0, 454.0, 482.0]	[246.0, 255.0, 256.0, 289.0, 306.0, 313.0, 354.0, 355.0, 468.0, 500.0, 597.0, 662.0]
134	[854.0]	[933.0]	[255.0, 256.0, 313.0, 354.0, 371.0, 373.0, 429.0, 461.0, 468.0, 470.0, 500.0, 505.0, 569.0, 733.0, 734.0]
135	[289.0, 306.0]	[289.0, 306.0, 454.0, 482.0, 933.0]	[255.0, 256.0, 313.0, 354.0, 369.0, 468.0, 470.0, 500.0, 558.0, 569.0, 662.0, 733.0]
136	[265.0, 289.0, 291.0, 306.0, 324.0, 325.0, 326.0]	[]	[255.0, 256.0, 313.0, 354.0, 355.0, 429.0, 470.0, 500.0, 563.0, 569.0, 662.0, 733.0]
137	[]	[289.0, 306.0, 307.0, 324.0, 326.0, 454.0, 482.0, 933.0]	[255.0, 256.0, 313.0, 354.0, 369.0, 373.0, 468.0, 470.0, 500.0, 558.0, 569.0, 733.0]

138	[246.0, 287.0, 288.0, 289.0, 290.0, 306.0, 307.0, 424.0, 440.0]	[246.0, 287.0, 288.0, 289.0, 290.0, 306.0, 307.0, 424.0, 440.0, 454.0, 482.0, 933.0]	[255.0, 256.0, 313.0, 354.0, 355.0, 569.0, 662.0]
139	[367.0]	[265.0, 267.0, 302.0, 324.0, 367.0, 589.0]	[255.0, 256.0, 313.0, 354.0, 355.0, 371.0, 373.0, 563.0, 569.0, 570.0, 662.0]
140	[367.0]	[933.0]	[255.0, 256.0, 313.0, 354.0, 369.0, 371.0, 373.0, 429.0, 468.0, 470.0, 500.0, 569.0, 570.0, 733.0]
141	[429.0]	[429.0, 484.0, 509.0, 563.0, 600.0]	[255.0, 256.0, 313.0, 324.0, 354.0, 355.0, 369.0, 373.0, 384.0, 468.0, 500.0, 569.0, 733.0]
142	[]	[362.0, 854.0, 933.0]	[255.0, 256.0, 313.0, 354.0, 369.0, 373.0, 468.0, 470.0, 500.0, 558.0, 569.0, 733.0]
143	[]	[362.0, 933.0]	[255.0, 256.0, 288.0, 313.0, 324.0, 354.0, 355.0, 369.0, 468.0, 470.0, 500.0]
144	[327.0, 336.0, 367.0, 367.0, 691.0, 716.0, 732.0, 795.0]	[]	[]
145	[634.0, 645.0, 691.0, 692.0, 713.0, 720.0, 776.0, 792.0, 792.0, 795.0, 834.0, 835.0, 836.0, 854.0]	[]	[]
146	[246.0, 287.0, 288.0, 289.0, 290.0, 306.0, 307.0, 594.0, 634.0, 645.0, 691.0, 713.0, 720.0, 792.0, 792.0, 834.0, 835.0, 836.0, 854.0]	[]	[]
147	[265.0, 306.0, 327.0, 336.0, 367.0, 367.0, 691.0, 713.0, 716.0, 732.0, 795.0, 851.0]	[]	[]
148	[246.0, 287.0, 288.0, 306.0, 347.0, 597.0]	[]	[272.0, 286.0, 313.0, 314.0, 327.0, 340.0, 354.0, 355.0, 359.0, 369.0]
149	[]	[]	[255.0, 256.0, 286.0, 313.0, 327.0, 340.0, 341.0, 354.0, 355.0, 369.0]
150	[265.0, 289.0, 691.0, 792.0, 795.0, 834.0, 854.0]	[]	[]
151	[246.0, 287.0, 288.0, 289.0, 305.0, 306.0, 307.0, 347.0]	[246.0, 247.0, 287.0, 288.0, 289.0, 290.0, 306.0, 307.0, 594.0, 595.0, 596.0]	[255.0, 256.0, 272.0, 286.0, 313.0, 314.0, 327.0, 340.0, 341.0, 354.0, 355.0, 369.0, 662.0]
152	[246.0, 247.0, 287.0, 288.0, 289.0, 290.0, 305.0, 306.0, 307.0, 347.0]	[246.0, 247.0, 287.0, 288.0, 289.0, 290.0, 298.0, 306.0, 307.0, 594.0, 595.0, 596.0]	[255.0, 256.0, 272.0, 286.0, 287.0, 313.0, 314.0, 327.0, 328.0, 340.0, 341.0, 354.0, 355.0, 359.0, 369.0, 372.0, 662.0]
153	[246.0, 247.0, 287.0, 288.0, 289.0, 290.0, 306.0, 307.0, 440.0, 597.0]	[246.0, 247.0, 288.0, 289.0, 290.0, 306.0, 307.0, 379.0, 380.0, 440.0, 454.0, 482.0, 594.0, 595.0, 597.0, 740.0]	[255.0, 256.0, 313.0, 354.0, 355.0, 408.0, 563.0, 662.0]
154	[429.0, 785.0]	[]	[]
155	[]	[]	[]
156	[265.0, 267.0, 302.0, 324.0, 367.0, 795.0]	[]	[]
157	[246.0, 247.0, 265.0, 287.0, 288.0, 289.0, 290.0, 306.0, 307.0, 570.0, 571.0, 572.0, 594.0, 595.0, 630.0, 632.0]	[246.0, 247.0, 288.0, 289.0, 290.0, 298.0, 305.0, 306.0, 307.0, 424.0, 440.0, 594.0, 595.0, 740.0]	[]
158	[814.0]	[]	[]
159	[]	[]	[]

160	[327.0, 367.0, 367.0, 691.0, 716.0, 732.0, 795.0, 851.0, 1487.0]	[]	[]
161	[265.0, 267.0, 302.0, 324.0, 795.0]	[246.0, 247.0, 265.0, 267.0, 288.0, 289.0, 290.0, 298.0, 306.0, 307.0, 324.0, 424.0, 589.0, 594.0, 595.0, 740.0]	[255.0, 256.0, 313.0, 313.0, 340.0, 354.0, 355.0, 662.0]
162	[634.0, 645.0, 691.0, 713.0, 720.0, 776.0, 792.0, 792.0, 795.0, 832.0, 833.0, 834.0, 836.0, 851.0, 854.0, 911.0, 912.0]	[]	[]
163	[634.0, 645.0, 691.0, 713.0, 720.0, 776.0, 792.0, 792.0, 795.0, 832.0, 834.0, 851.0, 854.0, 911.0]	[]	[]
164	[265.0, 267.0, 306.0, 324.0, 565.0, 589.0]	[]	[255.0, 256.0, 313.0, 354.0, 355.0, 408.0, 479.0, 662.0, 970.0]
165	[367.0, 634.0, 691.0, 713.0, 732.0, 772.0, 792.0, 795.0, 832.0, 834.0, 851.0, 911.0]	[]	[]
166	[327.0, 367.0, 367.0, 732.0, 795.0]	[]	[]
167	[691.0, 832.0, 834.0, 854.0, 911.0]	[]	[]
168	[246.0, 265.0, 287.0, 288.0, 289.0, 290.0, 306.0, 307.0, 424.0, 440.0]	[]	[]
169	[634.0, 645.0, 691.0, 713.0, 792.0, 795.0, 834.0, 854.0]	[]	[]
170	[834.0]	[]	[]
171	[]	[]	[255.0, 256.0, 313.0, 354.0, 355.0, 563.0, 569.0, 662.0]
172	[289.0, 327.0, 367.0, 367.0, 732.0, 769.0, 795.0]	[]	[]
173	[832.0, 854.0, 911.0]	[]	[]
174	[327.0, 367.0, 367.0, 732.0, 795.0, 851.0]	[]	[]
175	[246.0, 247.0, 287.0, 288.0, 289.0, 290.0, 306.0, 307.0, 308.0, 380.0, 440.0, 570.0, 594.0]	[246.0, 288.0, 289.0, 290.0, 306.0, 307.0, 308.0, 397.0, 423.0, 440.0, 454.0, 482.0, 483.0, 485.0, 594.0, 595.0, 740.0]	[]
176	[289.0, 306.0, 675.0, 736.0, 814.0, 815.0, 854.0]	[]	[]
177	[246.0, 247.0, 287.0, 288.0, 289.0, 290.0, 306.0, 307.0, 347.0, 594.0, 595.0, 834.0]	[]	[]
178	[]	[]	[]
179	[691.0, 834.0]	[]	[]
180	[634.0, 645.0, 691.0, 713.0, 792.0, 795.0, 834.0, 854.0]	[]	[]
181	[246.0, 247.0, 287.0, 288.0, 289.0, 290.0, 306.0, 307.0, 347.0]	[246.0, 247.0, 287.0, 288.0, 289.0, 290.0, 298.0, 306.0, 594.0, 595.0, 596.0]	[255.0, 256.0, 272.0, 286.0, 313.0, 314.0, 327.0, 328.0, 340.0, 341.0, 354.0, 355.0, 369.0, 662.0]
182	[246.0, 247.0, 287.0, 288.0, 289.0, 290.0, 306.0, 307.0, 440.0, 594.0]	[]	[]
183	[246.0, 265.0, 267.0, 287.0, 288.0, 289.0, 290.0, 306.0, 307.0, 324.0, 326.0, 440.0, 613.0, 634.0, 645.0, 691.0, 713.0, 792.0, 834.0]	[]	[]
184	[289.0, 327.0, 367.0, 367.0, 795.0]	[]	[]
185	[245.0, 246.0, 247.0, 287.0, 288.0, 289.0, 290.0, 305.0, 306.0, 307.0,	[]	[255.0, 256.0, 313.0, 354.0, 355.0, 479.0, 662.0]

	380.0, 424.0, 440.0, 570.0, 594.0, 595.0, 630.0, 834.0]		
186	[327.0, 367.0, 367.0, 795.0]	[]	[]
187	[]	[]	[255.0, 286.0, 313.0, 314.0, 340.0, 341.0, 354.0, 355.0, 369.0]
188	[246.0, 247.0, 287.0, 288.0, 289.0, 290.0, 306.0, 307.0, 440.0, 594.0]	[]	[]
189	[814.0]	[]	[]
190	[]	[]	[]
191	[]	[]	[255.0, 256.0, 313.0, 313.0, 340.0, 354.0, 355.0, 662.0]

6.10 Reaction Conditions and Results from the Isostructural Exploration

6.10.1 Overview

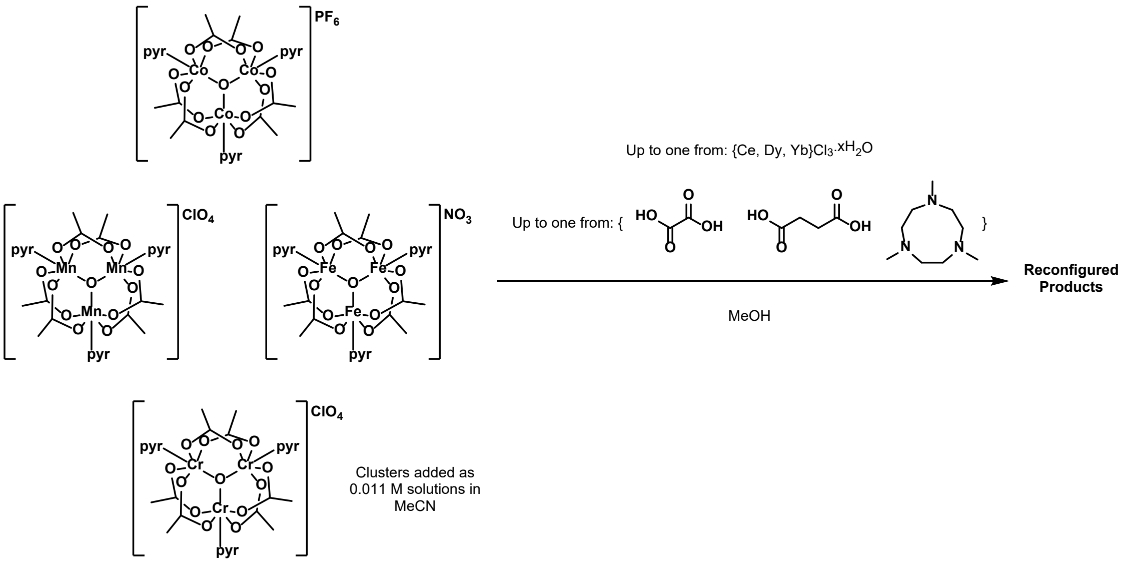

***Scheme S2**: General reaction scheme for the Isostructural Exploration. Not all clusters were used in every experiment.*

6.10.2 Graphical Representation of Reaction Conditions Sampled

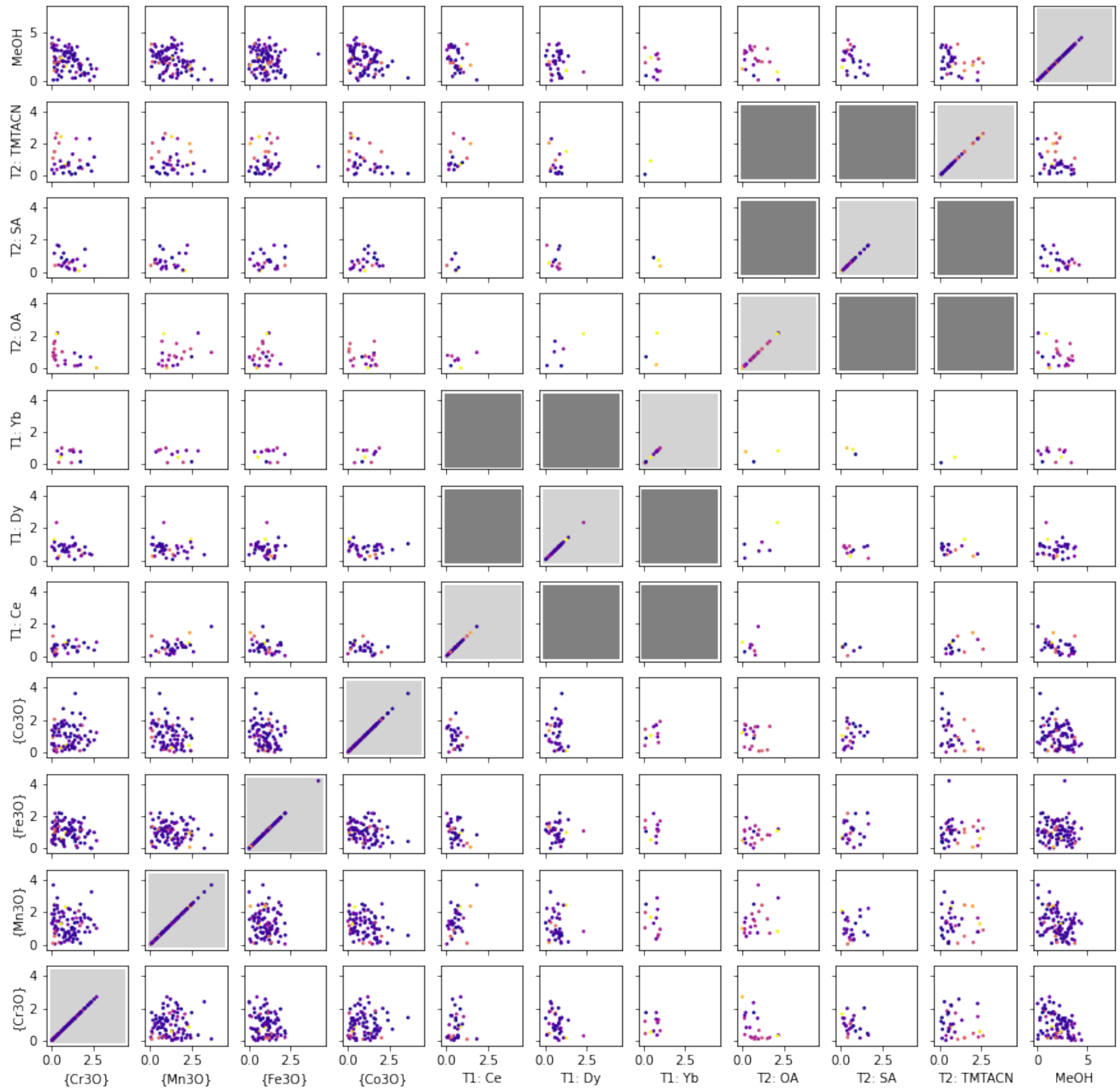

Figure S80: *Matrix scatter plot of each reagent/solvent for the Isothermal Exploration. Points are coloured based on AMF Score, with dark purple indicating the lowest values and yellow indicating the highest. Light grey background indicates the variable plotted against itself – giving an idea of the spread of data points between none of that reagent (0 mL) and only that reagent (7.5 mL). Dark grey background indicates that a potential combination of two variables is not possible as only one template may be chosen from each of the two sets.*

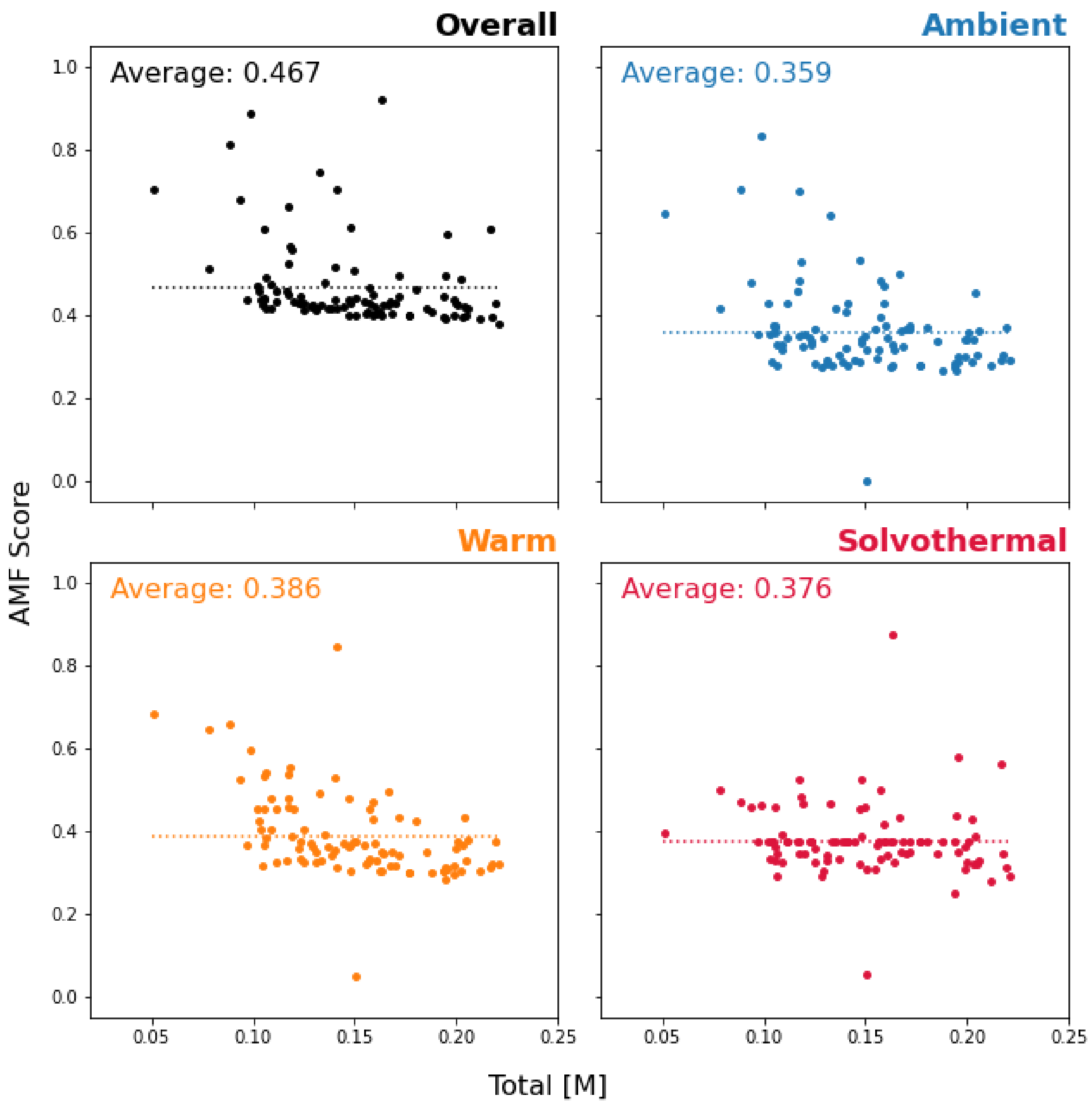

Figure S81*: Plots of Isostructural AMF score after Run 2 (overall average across three heating conditions, and score for each condition individually). For each plot, the average AMF value is plotted as a dashed line, and stated in the top left corner.*

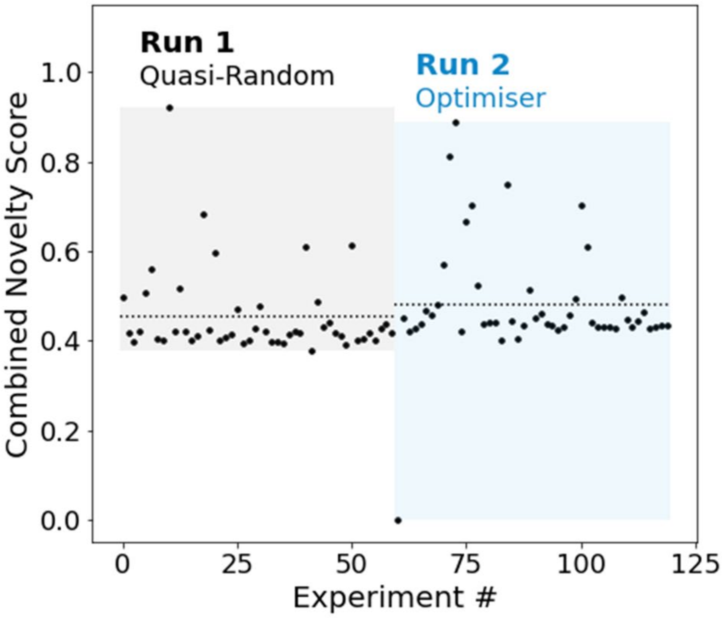

***Figure S82**: Chart indicating the Mapping Function, MF score for each set of reaction conditions probed in the Isostructural exploration. Each itera-tion has 48 data points. Colored bars are used to indicate the range of MF scores obtained, and group the data points by iteration. Text indicates the mode of exploration for that iteration, and grey dashed lines indicate the average MF value for each iteration. MF values are reassessed after each iteration, with the values shown coming from the final assessment, following the fourth iteration. Results are feature scaled within each exploration, so absolute MF values are not comparable with the Common Component exploration.*

6.10.3 List of Unique Peaks

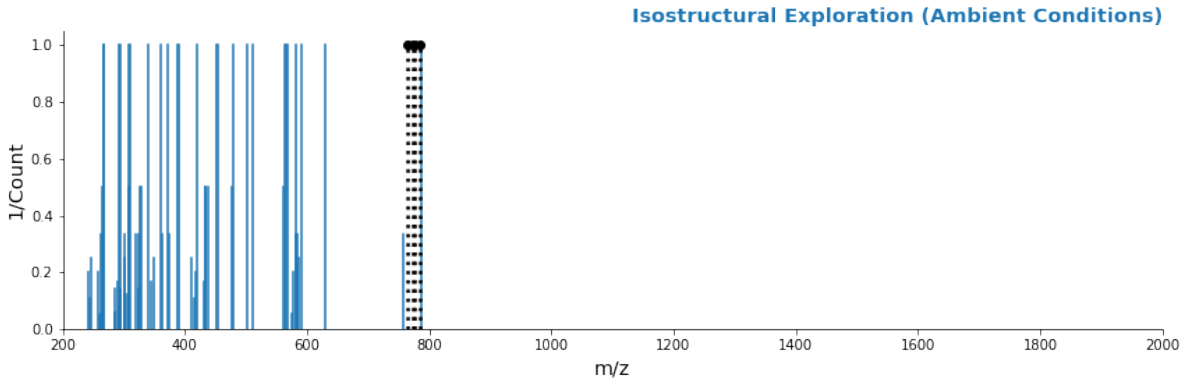

Figure S83: *Chart showing each unique peak identified in the Solvothermal Exploration at room temperature, with peak height plotted as the inverse of the number of times a given peak appears across the dataset of all 96 experiments ('1/Count'). Black dotted lines indicate the $[M]^+$ ions of the cluster starting materials to give context for the observed new peaks.*

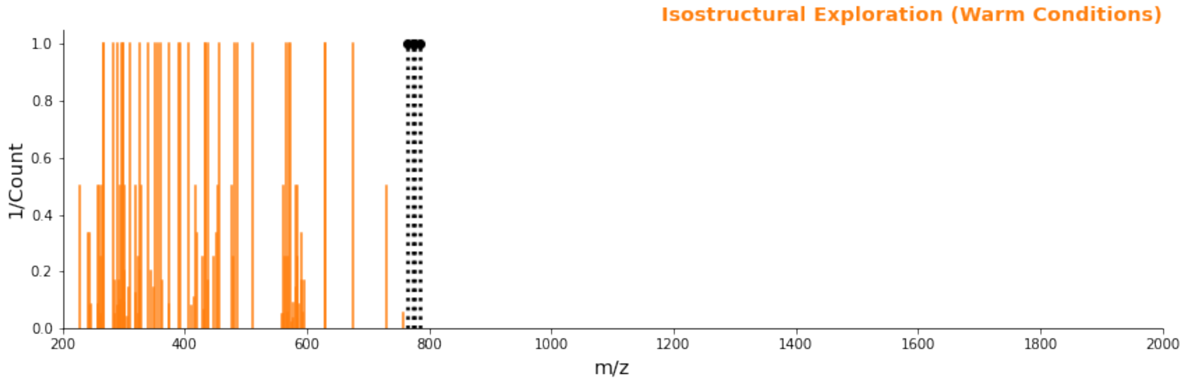

Figure S84: *Chart showing each unique peak identified in the Isostructural Exploration under the warm conditions (50 °C, 30 min), with peak height plotted as the inverse of the number of times a given peak appears across the dataset of all 96 experiments ('1/Count'). Black dotted lines indicate the $[M]^+$ ions of the cluster starting materials to give context for the observed new peaks.*

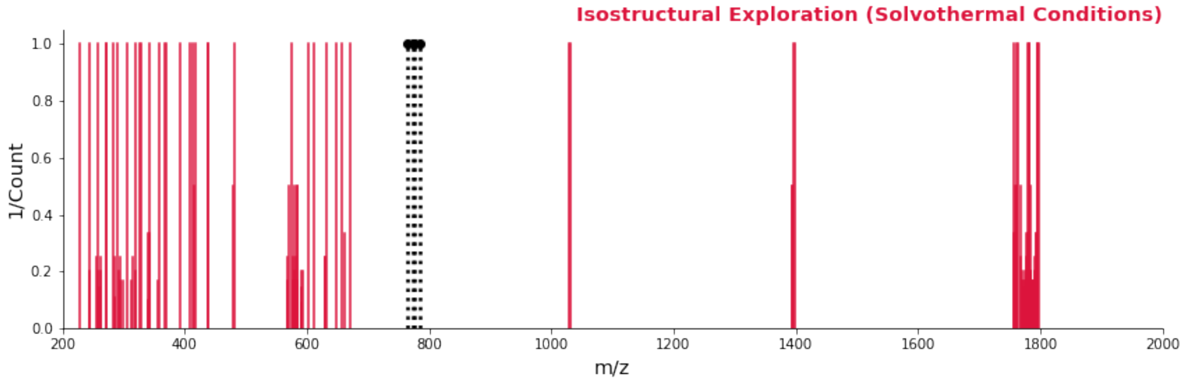

Figure S85: *Spectrum showing each unique peak identified in the Isostructural Exploration under the solvothermal conditions (90 °C, 4 days), with peak height plotted as the inverse of the number of times a given peak appears across the dataset of all 96 experiments ('1/Count'). Black dotted lines indicate the $[M]^+$ ions of the cluster starting materials to give context for the observed new peaks.*

***Table S8**: Unique Peaks in the Isostructural Exploration – m/z values of peaks which do not appear in the corresponding cluster standard MS spectra, as digitally identified from mass spectrometry data during calculation of the mapping function. Note that, despite HRMS being used, the algorithm interprets rounded versions of the peak m/z values, to ensure all common peaks are identified. As such, data is reported to this level of precision.*

Exp #	Room Temperature	Warm (50 °C, 30 min)	Solvothermal (90 °C, 4 days)
0	[576.0]	[576.0, 577.0, 585.0]	[1394.0, 1395.0, 1397.0, 1398.0, 1768.0, 1769.0, 1770.0, 1771.0, 1772.0, 1773.0, 1774.0, 1775.0, 1784.0, 1785.0, 1786.0, 1787.0, 1788.0, 1789.0, 1790.0]
1	[410.0]	[374.0, 410.0, 758.0]	[]
2	[]	[410.0, 758.0]	[]
3	[576.0, 577.0]	[560.0, 562.0, 568.0, 576.0, 577.0, 578.0, 585.0, 586.0, 594.0]	[]
4	[243.0, 261.0, 285.0, 306.0]	[246.0, 289.0, 306.0, 307.0, 324.0, 431.0, 758.0]	[256.0, 286.0, 313.0, 327.0, 328.0, 355.0, 359.0, 662.0]
5	[243.0, 261.0, 285.0, 576.0]	[243.0, 257.0, 260.0, 261.0, 263.0, 285.0, 293.0, 301.0, 306.0, 319.0, 416.0, 560.0, 562.0, 576.0, 577.0, 585.0, 594.0]	[227.0, 243.0, 244.0, 257.0, 258.0, 319.0, 320.0, 591.0]
6	[758.0]	[576.0, 758.0]	[]
7	[]	[576.0, 585.0]	[]
8	[243.0, 257.0, 261.0, 263.0, 285.0, 345.0, 431.0]	[243.0, 246.0, 257.0, 261.0, 285.0, 289.0, 293.0, 306.0, 319.0, 324.0, 326.0, 431.0, 576.0]	[282.0, 299.0, 313.0, 315.0, 340.0, 341.0, 342.0, 343.0, 355.0, 367.0, 370.0, 393.0, 408.0, 413.0, 437.0, 438.0, 479.0, 481.0, 590.0, 591.0, 648.0, 1759.0, 1760.0, 1761.0, 1762.0, 1763.0, 1771.0, 1772.0, 1773.0, 1774.0, 1775.0, 1776.0, 1777.0, 1778.0, 1779.0, 1780.0, 1781.0, 1786.0, 1787.0, 1788.0, 1789.0, 1790.0, 1791.0, 1792.0, 1793.0, 1794.0, 1795.0, 1796.0, 1797.0]
9	[576.0]	[560.0, 562.0, 563.0, 568.0, 576.0, 577.0, 578.0, 585.0, 586.0, 594.0, 595.0]	[]
10	[311.0, 374.0, 410.0, 758.0]	[292.0, 311.0, 351.0, 374.0, 392.0, 410.0, 758.0]	[]
11	[576.0]	[306.0, 560.0, 562.0, 568.0, 576.0, 577.0, 578.0, 585.0, 594.0]	[]
12	[576.0]	[576.0, 577.0, 585.0]	[]
13	[576.0, 577.0]	[560.0, 562.0, 568.0, 576.0, 577.0, 585.0, 594.0]	[]
14	[241.0, 243.0, 261.0, 263.0, 263.0, 285.0, 286.0, 289.0, 293.0, 294.0, 301.0, 302.0, 306.0, 348.0, 416.0, 417.0, 477.0, 581.0, 583.0, 586.0]	[241.0, 243.0, 257.0, 261.0, 263.0, 263.0, 285.0, 286.0, 289.0, 293.0, 294.0, 294.0, 301.0, 302.0, 306.0, 307.0, 324.0, 348.0, 363.0, 416.0, 417.0, 437.0, 477.0, 569.0, 576.0, 581.0, 583.0, 586.0]	[285.0, 293.0, 294.0, 295.0, 340.0, 569.0, 577.0, 578.0, 579.0, 580.0, 581.0, 583.0, 590.0, 591.0, 592.0, 594.0, 629.0, 630.0, 657.0, 671.0]
15	[576.0, 577.0]	[306.0, 560.0, 562.0, 568.0, 576.0, 577.0, 578.0, 585.0, 586.0, 594.0, 595.0]	[]

16	[]	[]	[315.0, 340.0, 343.0, 1756.0, 1757.0, 1758.0, 1759.0, 1760.0, 1761.0, 1768.0, 1769.0, 1770.0, 1771.0, 1772.0, 1773.0, 1774.0, 1775.0, 1776.0, 1777.0, 1778.0, 1779.0, 1782.0, 1783.0, 1784.0, 1785.0, 1786.0, 1787.0, 1788.0, 1789.0, 1790.0, 1791.0, 1792.0, 1793.0, 1794.0]
17	[]	[]	[]
18	[576.0]	[576.0]	[]
19	[]	[]	[]
20	[]	[]	[]
21	[374.0]	[374.0, 410.0, 758.0]	[]
22	[]	[576.0]	[]
23	[410.0]	[374.0, 410.0, 758.0]	[]
24	[300.0, 576.0]	[306.0, 324.0, 326.0, 374.0, 410.0, 560.0, 562.0, 568.0, 576.0, 577.0, 585.0, 594.0, 676.0, 758.0]	[256.0, 313.0, 355.0, 662.0]
25	[261.0, 324.0, 576.0]	[306.0, 324.0, 374.0, 410.0, 560.0, 562.0, 576.0, 577.0, 585.0, 758.0]	[]
26	[]	[576.0]	[]
27	[374.0, 410.0]	[292.0, 374.0, 410.0, 560.0, 576.0, 577.0, 758.0]	[]
28	[]	[]	[]
29	[576.0]	[560.0, 562.0, 568.0, 576.0, 577.0, 578.0, 585.0, 594.0]	[]
30	[576.0, 577.0]	[560.0, 562.0, 563.0, 568.0, 576.0, 577.0, 578.0, 585.0, 586.0, 594.0, 595.0]	[]
31	[758.0]	[576.0, 758.0]	[]
32	[431.0]	[306.0, 324.0]	[272.0, 289.0, 306.0, 315.0, 340.0, 341.0, 343.0, 590.0, 1030.0, 1031.0, 1757.0, 1758.0, 1759.0, 1769.0, 1770.0, 1771.0, 1772.0, 1773.0, 1774.0, 1775.0, 1776.0, 1777.0, 1778.0, 1779.0, 1784.0, 1785.0, 1786.0, 1787.0, 1788.0, 1789.0, 1790.0, 1791.0, 1792.0, 1793.0, 1794.0]
33	[]	[]	[]
34	[]	[]	[1394.0, 1395.0, 1757.0, 1758.0, 1759.0, 1769.0, 1770.0, 1771.0, 1772.0, 1773.0, 1774.0, 1775.0, 1776.0, 1777.0, 1784.0, 1785.0, 1786.0, 1787.0, 1788.0, 1789.0, 1790.0, 1791.0, 1792.0]
35	[]	[576.0, 758.0]	[256.0, 313.0, 355.0]
36	[576.0, 577.0]	[560.0, 562.0, 563.0, 568.0, 576.0, 577.0, 578.0, 585.0, 586.0, 590.0, 594.0, 595.0]	[]
37	[576.0]	[374.0, 410.0, 560.0, 562.0, 576.0, 577.0, 585.0, 758.0]	[]
38	[]	[576.0, 758.0]	[]
39	[]	[758.0]	[]
40	[243.0, 261.0, 263.0, 285.0, 292.0, 293.0, 325.0, 576.0]	[243.0, 261.0, 285.0, 345.0, 431.0, 576.0, 577.0, 585.0]	[243.0, 257.0, 261.0, 262.0, 263.0, 271.0, 285.0, 299.0, 319.0, 340.0,

			341.0, 355.0, 568.0, 569.0, 577.0, 578.0, 579.0, 580.0, 581.0, 583.0, 591.0, 592.0, 594.0, 1770.0, 1771.0, 1772.0, 1773.0, 1774.0, 1783.0, 1784.0, 1785.0, 1786.0, 1787.0, 1788.0, 1789.0, 1790.0, 1791.0]
41	[289.0, 348.0]	[306.0, 374.0, 410.0, 758.0]	[]
42	[]	[374.0, 410.0, 576.0, 577.0, 758.0]	[]
43	[576.0]	[560.0, 562.0, 576.0, 577.0, 585.0, 594.0]	[]
44	[576.0]	[374.0, 410.0, 560.0, 562.0, 568.0, 576.0, 577.0, 585.0, 594.0, 758.0]	[]
45	[576.0]	[560.0, 562.0, 568.0, 576.0, 577.0, 578.0, 585.0, 586.0, 594.0, 595.0]	[]
46	[246.0, 261.0, 263.0, 285.0, 286.0, 293.0, 294.0, 301.0, 306.0, 324.0, 416.0, 431.0]	[]	[315.0, 340.0, 590.0, 591.0, 1773.0, 1774.0, 1775.0, 1776.0]
47	[]	[]	[]
48	[]	[]	[]
49	[]	[]	[]
50	[]	[]	[]
51	[]	[]	[]
52	[]	[576.0, 577.0, 585.0, 594.0]	[]
53	[261.0, 263.0, 285.0, 293.0, 325.0, 345.0, 416.0]	[243.0, 257.0, 261.0, 263.0, 285.0, 319.0, 325.0, 345.0, 431.0, 576.0, 585.0]	[]
54	[]	[]	[]
55	[246.0, 261.0, 263.0, 285.0, 289.0, 293.0, 306.0, 324.0, 326.0, 345.0, 431.0]	[243.0, 246.0, 257.0, 261.0, 289.0, 306.0, 324.0, 389.0, 431.0, 576.0]	[]
56	[241.0, 243.0, 257.0, 261.0, 262.0, 263.0, 263.0, 285.0, 286.0, 293.0, 294.0, 301.0, 416.0, 417.0, 581.0, 583.0, 586.0]	[241.0, 243.0, 257.0, 261.0, 262.0, 263.0, 263.0, 285.0, 286.0, 293.0, 294.0, 301.0, 319.0, 416.0, 560.0, 562.0, 568.0, 576.0, 577.0, 581.0, 582.0, 583.0, 584.0, 585.0, 591.0, 594.0]	[261.0, 262.0, 263.0, 285.0, 299.0, 569.0, 577.0, 579.0, 581.0, 582.0, 583.0, 591.0, 592.0, 594.0]
57	[241.0, 243.0, 257.0, 261.0, 262.0, 263.0, 263.0, 285.0, 286.0, 293.0, 294.0, 301.0, 302.0, 319.0, 345.0, 416.0, 417.0, 434.0, 561.0, 569.0, 581.0, 582.0, 583.0, 586.0]	[227.0, 243.0, 244.0, 257.0, 258.0, 261.0, 262.0, 263.0, 263.0, 285.0, 286.0, 293.0, 294.0, 301.0, 319.0, 320.0, 416.0, 561.0, 569.0, 581.0, 582.0, 583.0, 584.0, 585.0, 586.0, 591.0]	[243.0, 257.0, 261.0, 262.0, 263.0, 285.0, 293.0, 319.0, 416.0, 568.0, 569.0, 570.0, 577.0, 581.0, 582.0, 583.0, 584.0, 585.0, 591.0, 592.0]
58	[241.0, 243.0, 246.0, 257.0, 261.0, 263.0, 263.0, 285.0, 286.0, 289.0, 293.0, 294.0, 295.0, 301.0, 302.0, 306.0, 307.0, 309.0, 319.0, 339.0, 348.0, 388.0, 389.0, 416.0, 417.0, 431.0, 433.0, 477.0, 586.0, 591.0, 629.0]	[227.0, 243.0, 244.0, 246.0, 257.0, 258.0, 261.0, 263.0, 263.0, 285.0, 286.0, 289.0, 293.0, 294.0, 295.0, 301.0, 302.0, 306.0, 307.0, 319.0, 320.0, 348.0, 416.0, 431.0, 576.0, 581.0, 590.0, 591.0, 629.0, 630.0]	[243.0, 257.0, 261.0, 263.0, 285.0, 299.0, 313.0, 319.0, 340.0, 568.0, 569.0, 575.0, 577.0, 578.0, 579.0, 581.0, 582.0, 583.0, 590.0, 591.0, 592.0, 594.0, 629.0, 630.0]
59	[261.0, 285.0, 325.0]	[261.0, 325.0]	[261.0, 285.0]
60	[246.0, 261.0, 285.0, 289.0, 293.0, 306.0, 307.0, 324.0, 348.0, 361.0, 363.0, 363.0, 419.0, 433.0, 437.0, 451.0, 455.0, 479.0]	[246.0, 261.0, 263.0, 285.0, 289.0, 306.0, 307.0, 324.0, 348.0, 363.0, 363.0, 419.0, 437.0, 438.0, 451.0, 455.0, 457.0, 479.0]	[]
61	[261.0, 263.0, 265.0, 266.0, 267.0, 289.0, 300.0, 302.0, 306.0, 324.0, 363.0, 437.0, 561.0, 563.0, 565.0]	[261.0, 265.0, 266.0, 267.0, 282.0, 289.0, 300.0, 302.0, 306.0, 307.0,	[256.0, 313.0, 340.0, 355.0, 662.0]

		324.0, 325.0, 326.0, 327.0, 348.0, 437.0]	
62	[261.0, 263.0, 263.0, 265.0, 285.0, 286.0, 293.0, 294.0, 301.0, 329.0, 416.0]	[243.0, 257.0, 261.0, 263.0, 285.0, 293.0, 301.0, 329.0, 431.0, 560.0, 562.0, 568.0, 576.0, 577.0, 578.0, 585.0, 586.0, 594.0]	[261.0, 285.0, 286.0, 293.0, 294.0, 294.0, 299.0, 416.0, 591.0, 592.0, 629.0, 630.0]
63	[261.0, 329.0, 510.0]	[261.0, 329.0, 576.0, 585.0]	[]
64	[]	[]	[]
65	[]	[261.0, 292.0, 510.0, 581.0, 585.0]	[286.0, 293.0, 294.0, 294.0, 295.0, 295.0, 417.0, 479.0]
66	[]	[]	[]
67	[241.0, 243.0, 257.0, 261.0, 262.0, 263.0, 263.0, 285.0, 286.0, 293.0, 294.0, 300.0, 301.0, 319.0, 345.0, 345.0, 372.0, 416.0, 417.0, 431.0, 434.0, 503.0, 788.0]	[243.0, 244.0, 246.0, 257.0, 260.0, 261.0, 262.0, 263.0, 285.0, 292.0, 293.0, 294.0, 294.0, 301.0, 319.0, 324.0, 325.0, 345.0, 407.0, 416.0, 431.0, 434.0, 581.0]	[243.0, 257.0, 261.0, 262.0, 263.0, 285.0, 299.0, 319.0, 340.0, 568.0, 569.0, 570.0, 577.0, 578.0, 579.0, 581.0, 582.0, 583.0, 584.0, 585.0, 591.0, 592.0, 594.0]
68	[]	[]	[]
69	[]	[]	[]
70	[]	[]	[]
71	[]	[297.0, 298.0, 299.0, 301.0, 324.0, 560.0, 562.0, 563.0, 568.0, 576.0, 577.0, 578.0, 585.0, 586.0, 594.0, 595.0]	[]
72	[]	[306.0, 324.0]	[]
73	[306.0, 324.0, 326.0]	[576.0, 577.0, 585.0]	[]
74	[]	[]	[]
75	[]	[246.0, 261.0, 285.0, 289.0, 306.0, 431.0, 568.0, 576.0, 577.0, 585.0]	[]
76	[261.0, 285.0, 293.0, 301.0, 416.0]	[246.0, 261.0, 285.0, 289.0, 306.0, 324.0, 431.0]	[]
77	[261.0, 263.0, 285.0, 293.0, 324.0]	[576.0, 577.0, 585.0]	[]
78	[]	[429.0, 447.0, 562.0, 565.0, 568.0, 569.0, 576.0, 577.0, 585.0, 586.0]	[]
79	[]	[243.0, 246.0, 285.0, 289.0, 292.0, 306.0, 307.0, 324.0, 348.0, 363.0, 431.0, 437.0, 451.0, 479.0, 481.0, 486.0]	[]
80	[]	[241.0, 243.0, 246.0, 257.0, 261.0, 263.0, 263.0, 265.0, 285.0, 286.0, 289.0, 290.0, 292.0, 293.0, 294.0, 294.0, 295.0, 301.0, 302.0, 306.0, 307.0, 319.0, 348.0, 361.0, 363.0, 373.0, 416.0, 417.0, 419.0, 431.0, 433.0, 437.0, 477.0, 479.0, 561.0, 569.0, 571.0, 573.0, 581.0, 583.0, 585.0, 586.0, 590.0, 591.0, 591.0, 731.0]	[]
81	[]	[261.0, 285.0, 289.0, 306.0, 324.0, 340.0, 355.0, 429.0, 447.0, 591.0, 731.0]	[285.0, 286.0, 293.0, 294.0, 295.0, 340.0, 591.0, 602.0, 612.0, 629.0, 630.0, 631.0]
82	[]	[429.0, 447.0, 568.0, 585.0]	[]
83	[]	[429.0, 447.0]	[]
84	[]	[576.0]	[]
85	[]	[560.0, 562.0, 568.0, 576.0, 577.0, 578.0, 585.0, 594.0]	[]
86	[]	[261.0, 263.0, 285.0, 293.0, 306.0, 324.0, 431.0]	[]

87	[]	[246.0, 261.0, 263.0, 263.0, 285.0, 286.0, 289.0, 293.0, 294.0, 301.0, 306.0, 324.0, 348.0, 363.0, 416.0, 419.0, 431.0, 437.0, 451.0, 455.0, 479.0]	[]
88	[]	[261.0, 262.0, 263.0, 285.0, 293.0, 306.0, 324.0, 326.0, 576.0, 577.0, 585.0]	[]
89	[]	[261.0, 576.0]	[]
90	[]	[246.0, 257.0, 261.0, 263.0, 285.0, 293.0, 301.0, 306.0, 324.0, 345.0, 416.0, 431.0]	[]
91	[]	[300.0, 302.0, 324.0, 326.0, 374.0, 410.0, 576.0]	[]
92	[]	[576.0]	[]
93	[]	[261.0, 263.0, 306.0, 324.0, 345.0, 431.0]	[]
94	[]	[261.0, 576.0]	[]
95	[]	[]	[]

6.11 Dataset Analysis of the Unique Peak Data for the Common Component Exploration

This section deals with the deconvolution of MS data to gain insights into the chemistry via data science techniques. The analysis pipeline is approached chronologically and uses the unique peak data presented in Tables S7 or S8.

6.11.1 Dimensionality Reduction by Iteration

The lists of unique peaks for each experiment were converted into a binary-style notation consisting of a string of equal length to the number of unique peaks across the entire dataset, with a '1' used to indicate presence of that peak and a '0' for absence. These binary-style 'barcodes' were combined to give an $m \times n$ array where m is experiment number and n a list of all unique peaks across the dataset. Arrays were formulated for each temperature, and sliced to give subarrays for each iterations.

The arrays were then subjected to matrix decomposition via Non-negative Matrix Factorisation (NMF) to give a low-rank approximations NMF was chosen for its ablity to learn the 'parts of objects' as shown in the seminal paper by Lee and Seung.[29] Here, the parts should correspond to clusters of m/z peaks which commonly occur together.

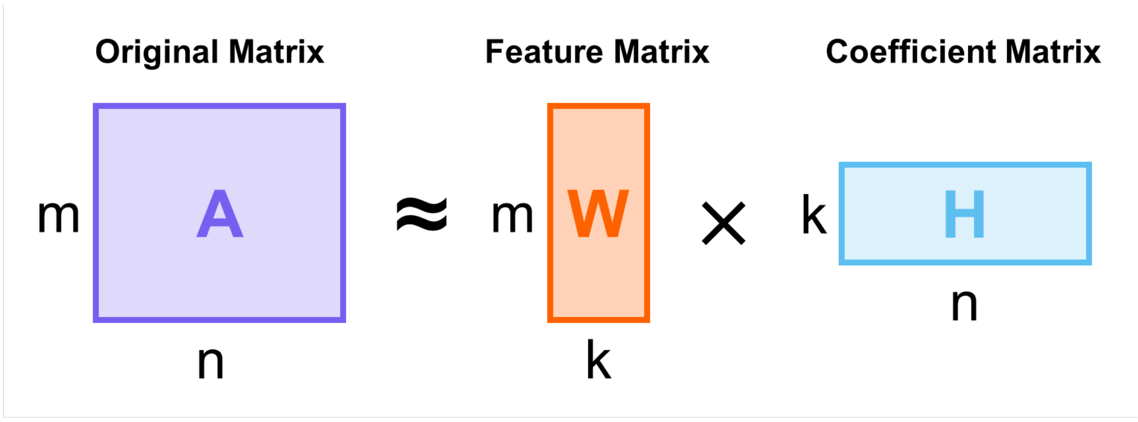

***Figure S86**: Diagrammatic representation of the decomposition of an original matrix, A to a feature matrix, W and coefficient matrix, H by non-negative matrix factorisaiton, NMF. The lower rank approximation, k corresponds to our MS Archetypes. Dimensions of the original matrix, m and n correspond to sample number and a list of the m/z values of all unique peaks across the dataset respectively.*

Low-rank approximations with sequentially increasing numbers of components from 2 to 48 (equivalent rank to the original dataset) were generated. Approximations of each rank are generated multiple times and an average taken as equivalent NMF approximations may exist. However, in this instance, we see very no variation between different approximations of the same rank until the reconstruction error is minimised. This is beyond the region of our interest, and the results are treated as deterministic.

Datasets with less variance in the co-occurrence of their unique peaks would require less components to accurately reproduce the data, and would therefore provide a metric to indicate the success of an exploration.

For the Common Component Exploration, Runs 2 and 4 with GPBO sampling generally indicated a greater diversity of product distributions than Runs 1 or 3. The only exception was for the

Solvothermal conditions, where Run 2 required fewer components to minimise the error in the lower rank approximation than Run 3.

Prinicipal Component Analysis (PCA), another matrix decomposition technique, is more common in chemistry. Fig. S79 shows the use of both NMF and PCA for decomposition to demonstrate that both techniques give near identical results.

However, as NMF is constrained to give only positive values as component scores, the data is more generally interpretable, and this technique was used for proceeding steps in the data analysis pipeline.

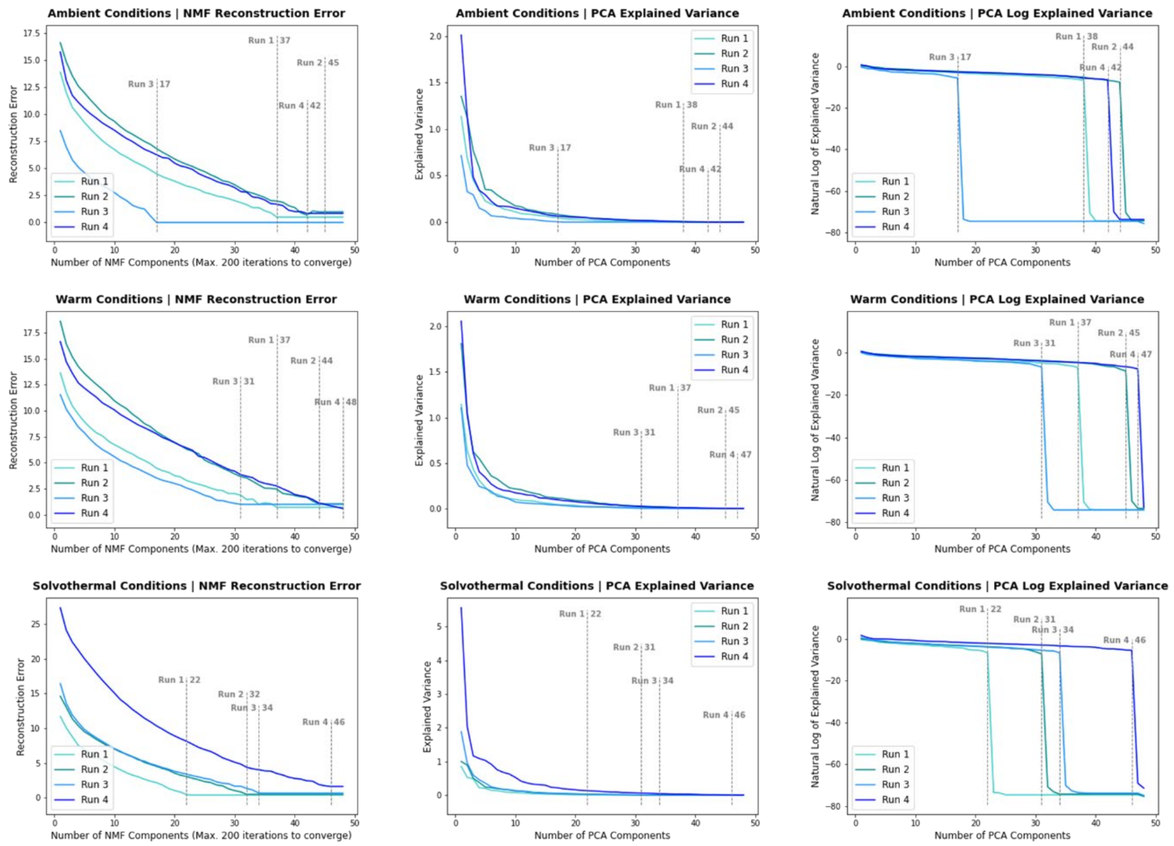

Figure S87: *Graphs indicating how the error in reconstruction of the dataset is minimised by increasing the rank of the low-rank approximation: (left) NMF, (center and right) PCA. Note that the error measure used for each technique is different: reconstruction error for NMF and explained variance for PCA. As this work is only interested in these values relative to the minimum value observed for each individual trendline, the difference does not affect the comparison. PCA is plotted with both absolute explained variance (center) and the log value (right). This is to make the point of transition from a exponential decay to a constant value clear. Grey dotted lines indicate the number of components at which the minimal value is first reached for each trendline, and are labelled with the iteration and number of components.*

6.11.2 Dimensionality Reduction by Temperature

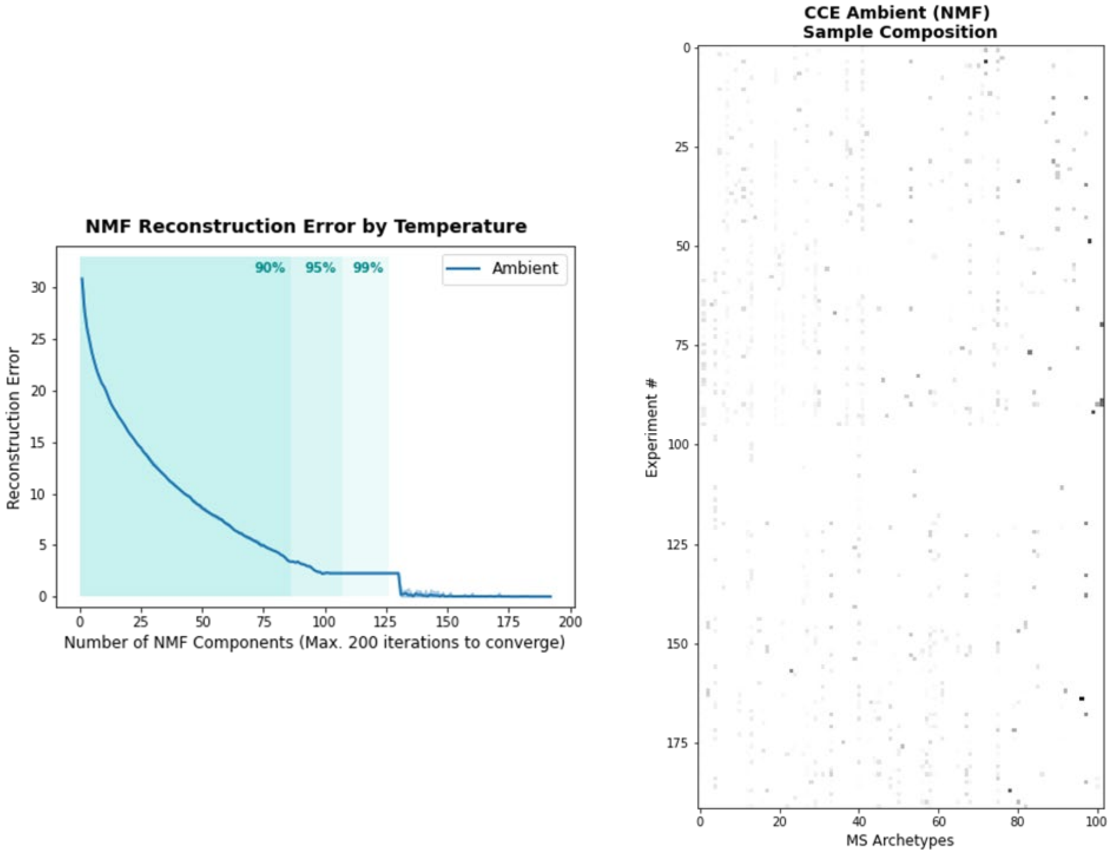

***Figure S88**: Minimisation of reconstruction error with increasing component number of NMF components for the Common Component Exploration Ambient conditions (left). Resulting NMF W matrix with 102 components (right).*

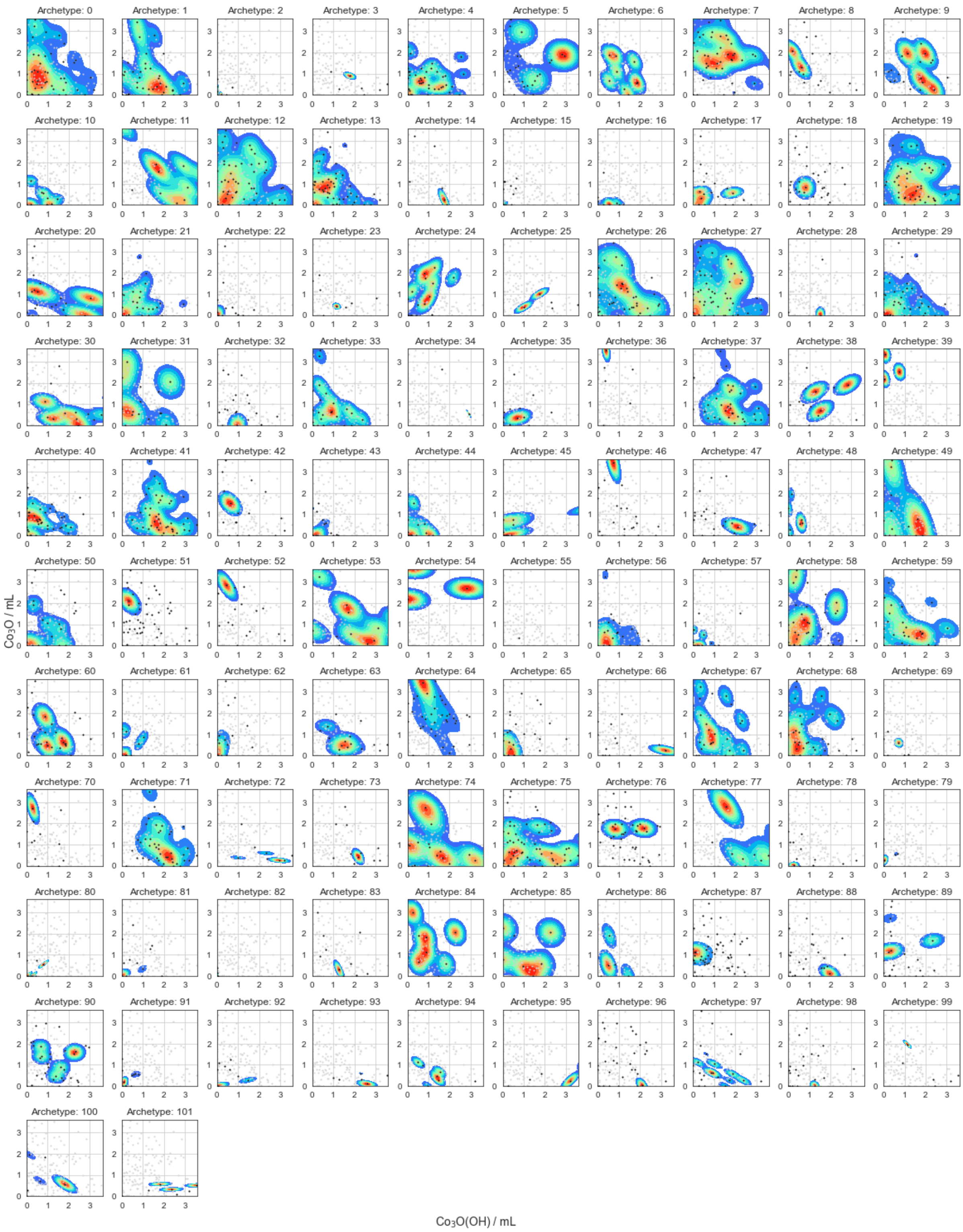

Figure S89: *Kernel density estimation weighted by component score for each Common Component Ambient Conditions archetype. Correlations shown between the volumes of clusters: {$Co_3O(OH)$} and {Co_3O}. Points with a non-negligible component score for the archetype are coloured black, and the remainder of the dataset shown in light grey. Unweighted kernel density estimates are shown for each variable on the diagonal.*

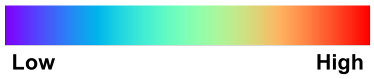

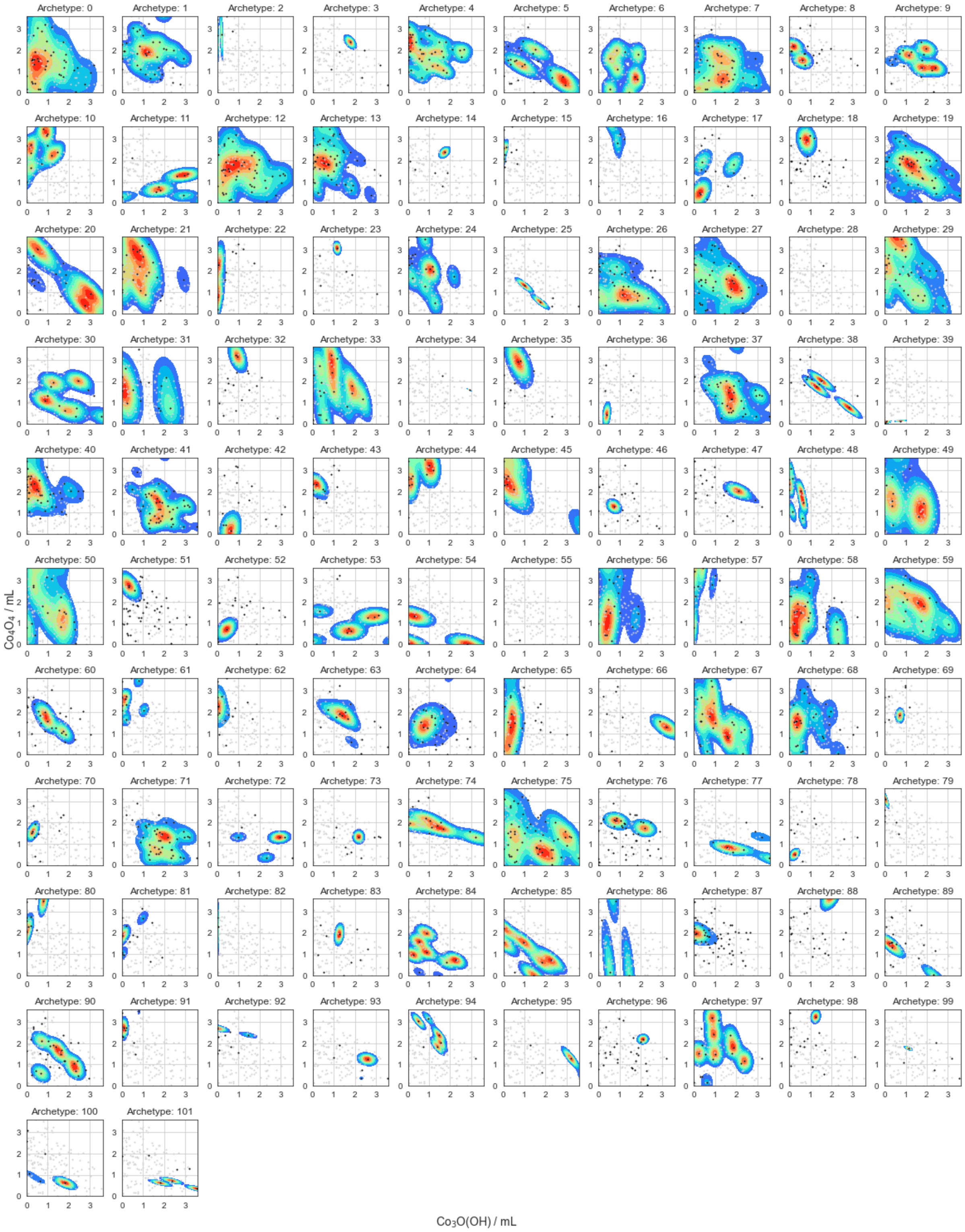

Figure S90: *Kernel density estimation weighted by component score for each Common Component Ambient Conditions archetype. Correlations shown between the volumes of clusters: {$Co_3O(OH)$} and {Co_4O_4}. Points with a non-negligible component score for the archetype are coloured black, and the remainder of the dataset shown in light grey. Unweighted kernel density estimates are shown for each variable on the diagonal.*

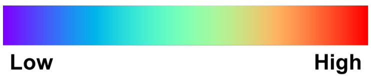

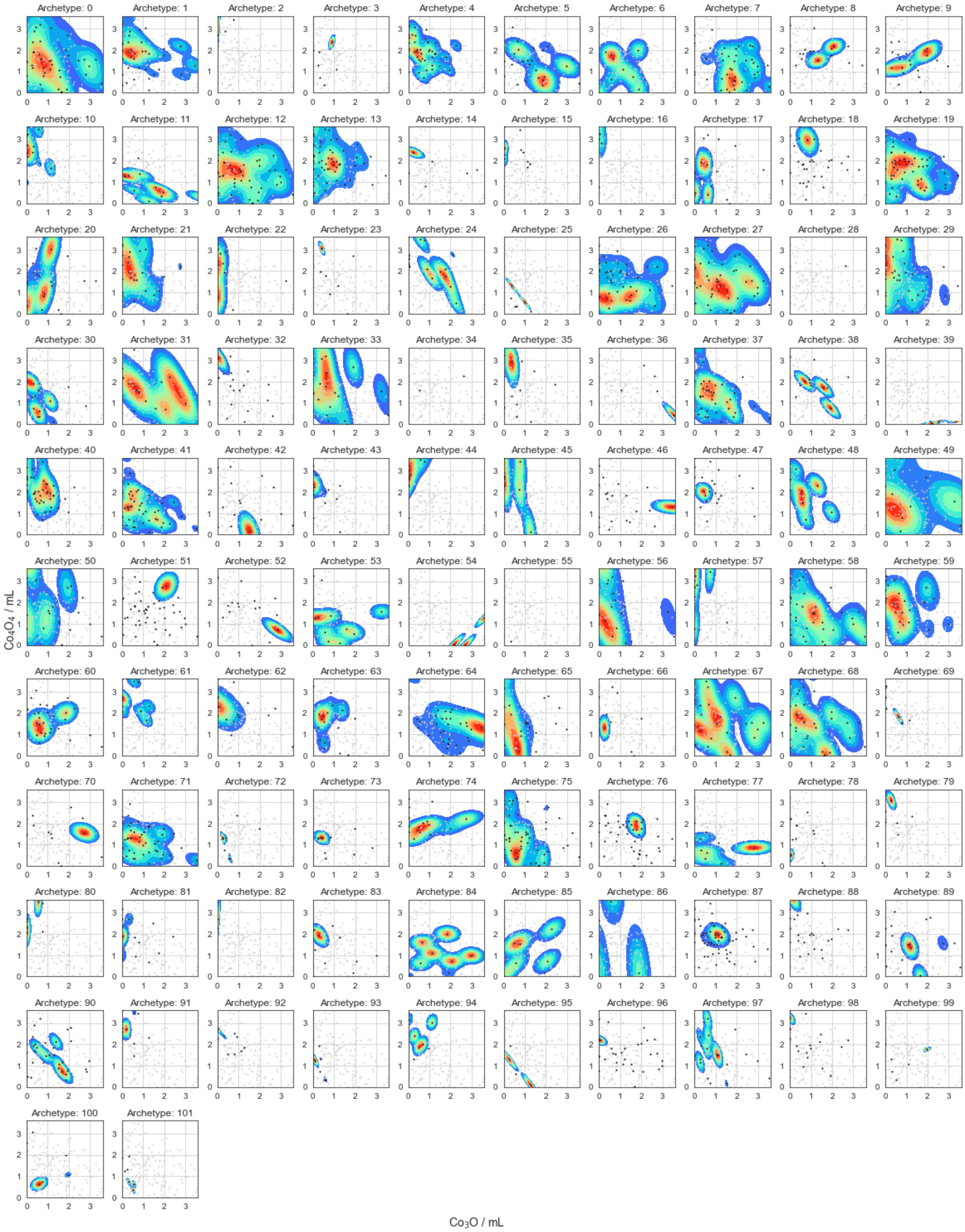

***Figure S91**: Kernel density estimation weighted by component score for each Common Component Ambient Conditions archetype. Correlations shown between the volumes of clusters: {Co_3O} and {Co_4O_4}. Points with a non-negligible component score for the archetype are coloured black, and the remainder of the dataset shown in light grey. Unweighted kernel density estimates are shown for each variable on the diagonal.*

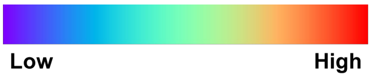

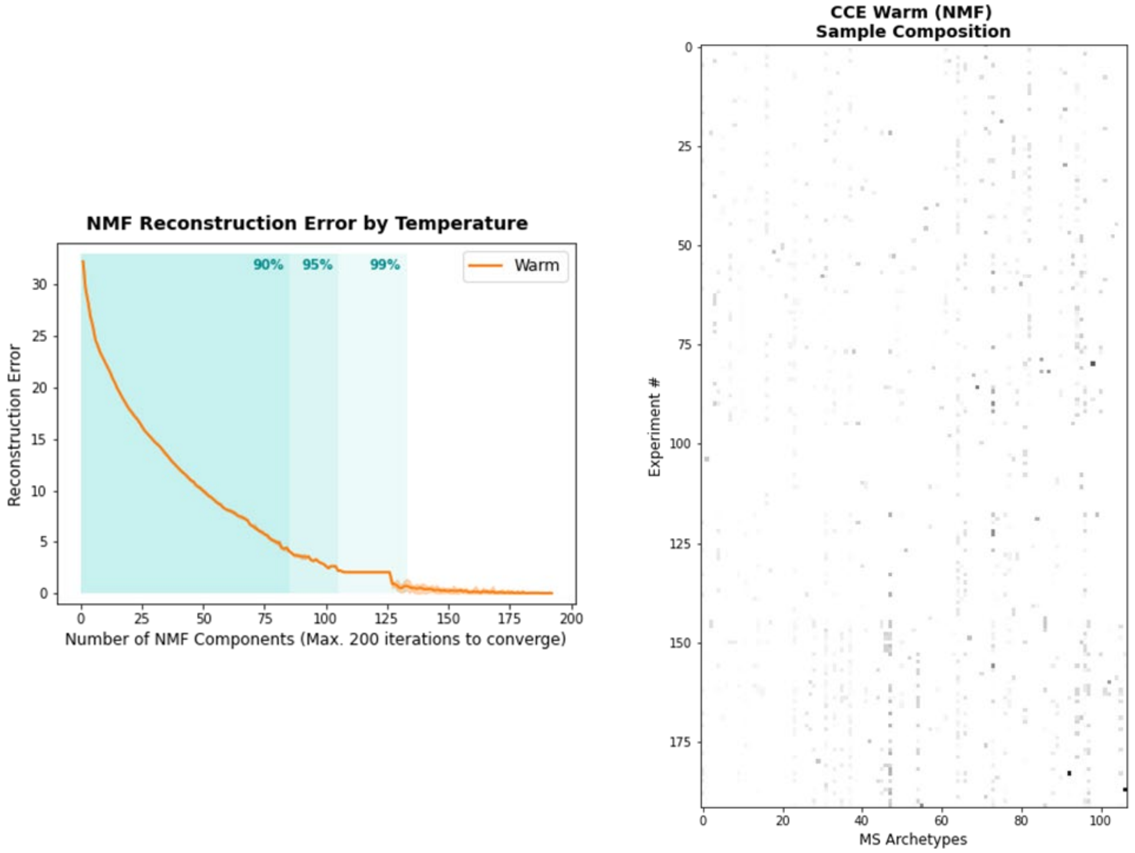

Figure S92*: Minimisation of reconstruction error with increasing component number of NMF components for the Common Component Exploration Warm conditions (left). Resulting NMF W matrix with 107 components (right).*

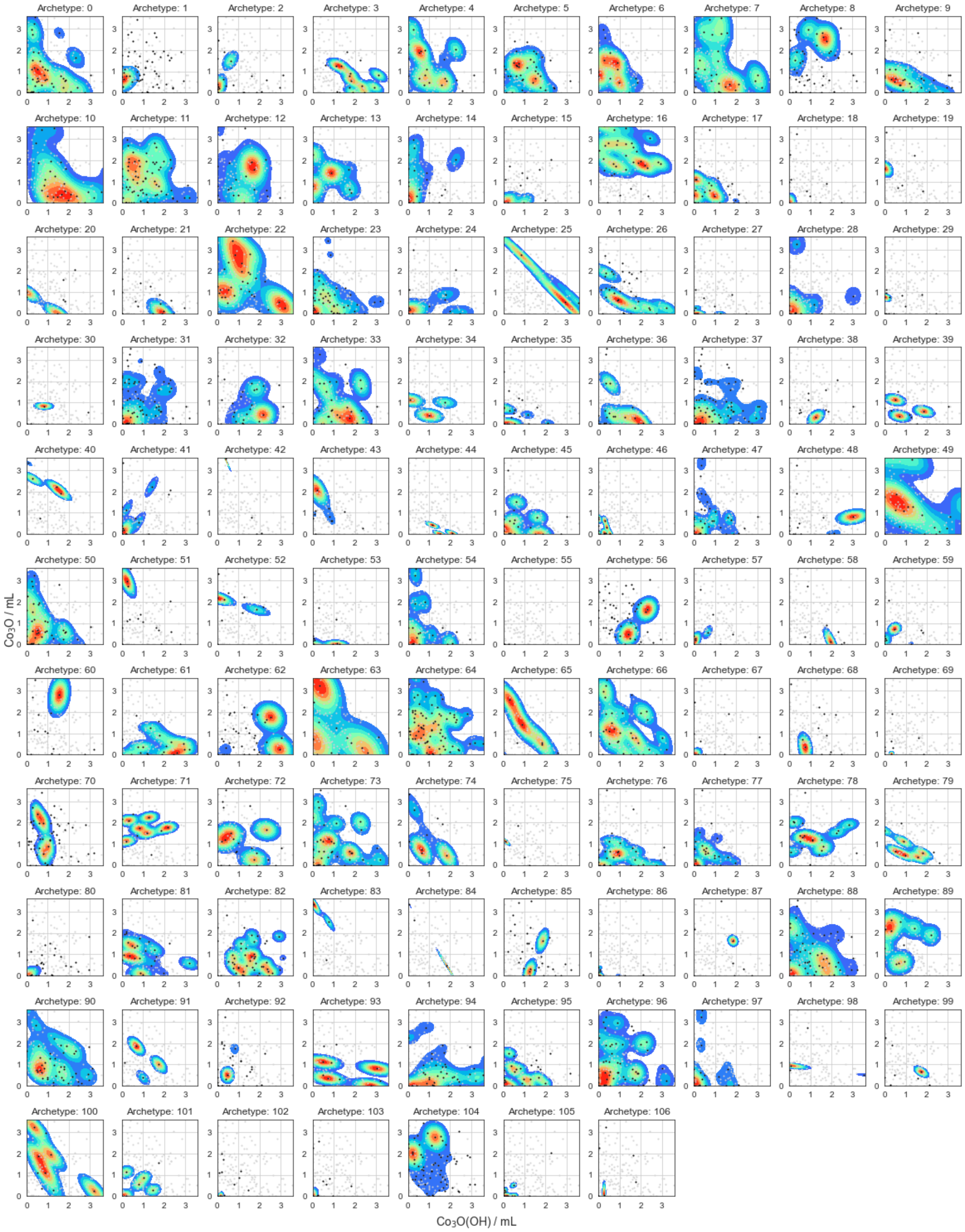

Figure S93: *Kernel density estimation weighted by component score for each Common Component Warm Conditions archetype. Correlations shown between the volumes of clusters: {$Co_3O(OH)$} and {Co_3O}. Points with a non-negligible component score for the archetype are coloured black, and the remainder of the dataset shown in light grey. Unweighted kernel density estimates are shown for each variable on the diagonal.*

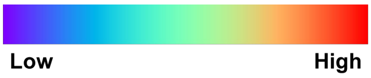

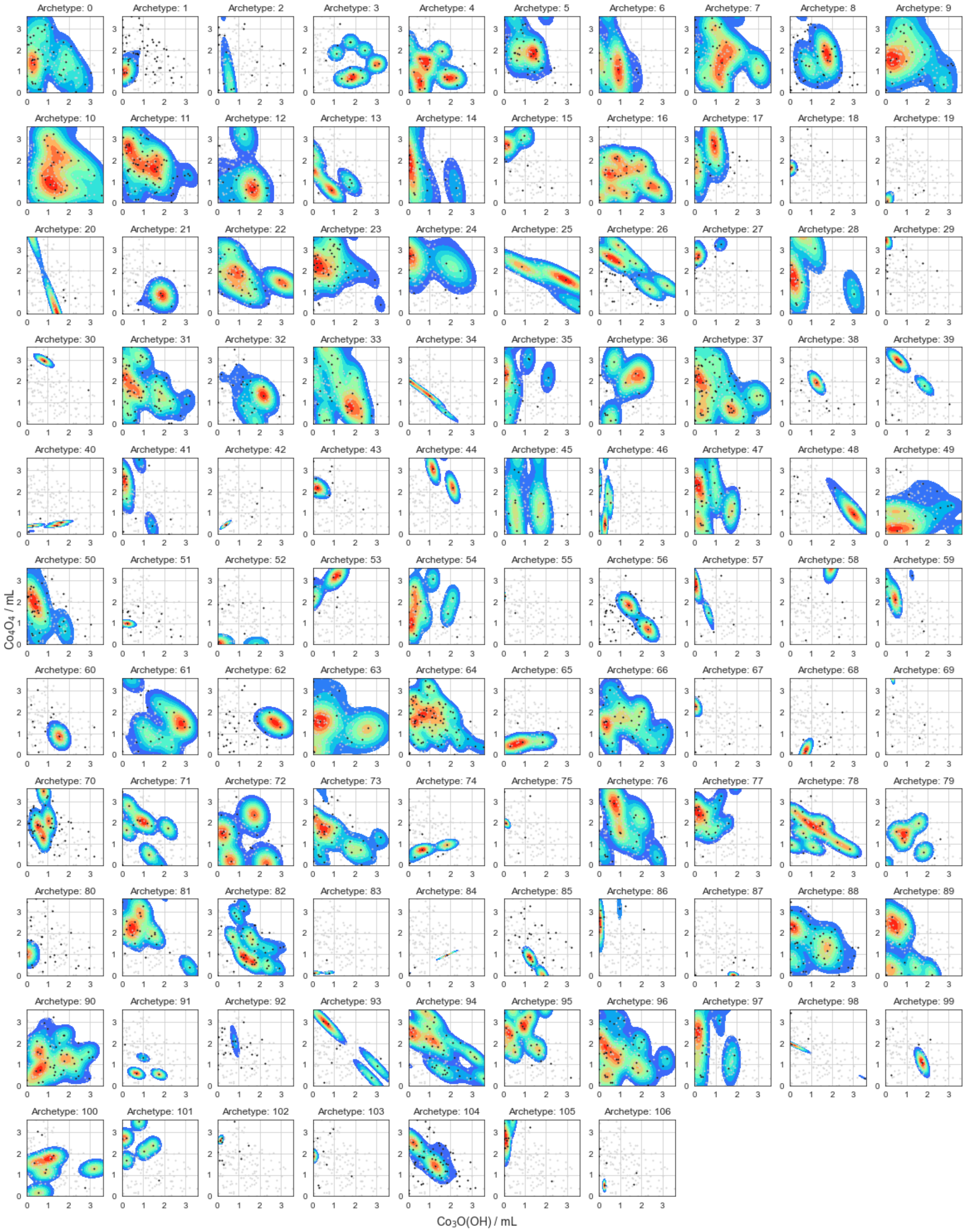

Figure S94*: Kernel density estimation weighted by component score for each Common Component Warm Conditions archetype. Correlations shown between the volumes of clusters: {$Co_3O(OH)$} and {Co_4O_4}. Points with a non-negligible component score for the archetype are coloured black, and the remainder of the dataset shown in light grey. Unweighted kernel density estimates are shown for each variable on the diagonal.*

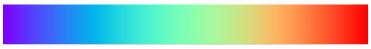

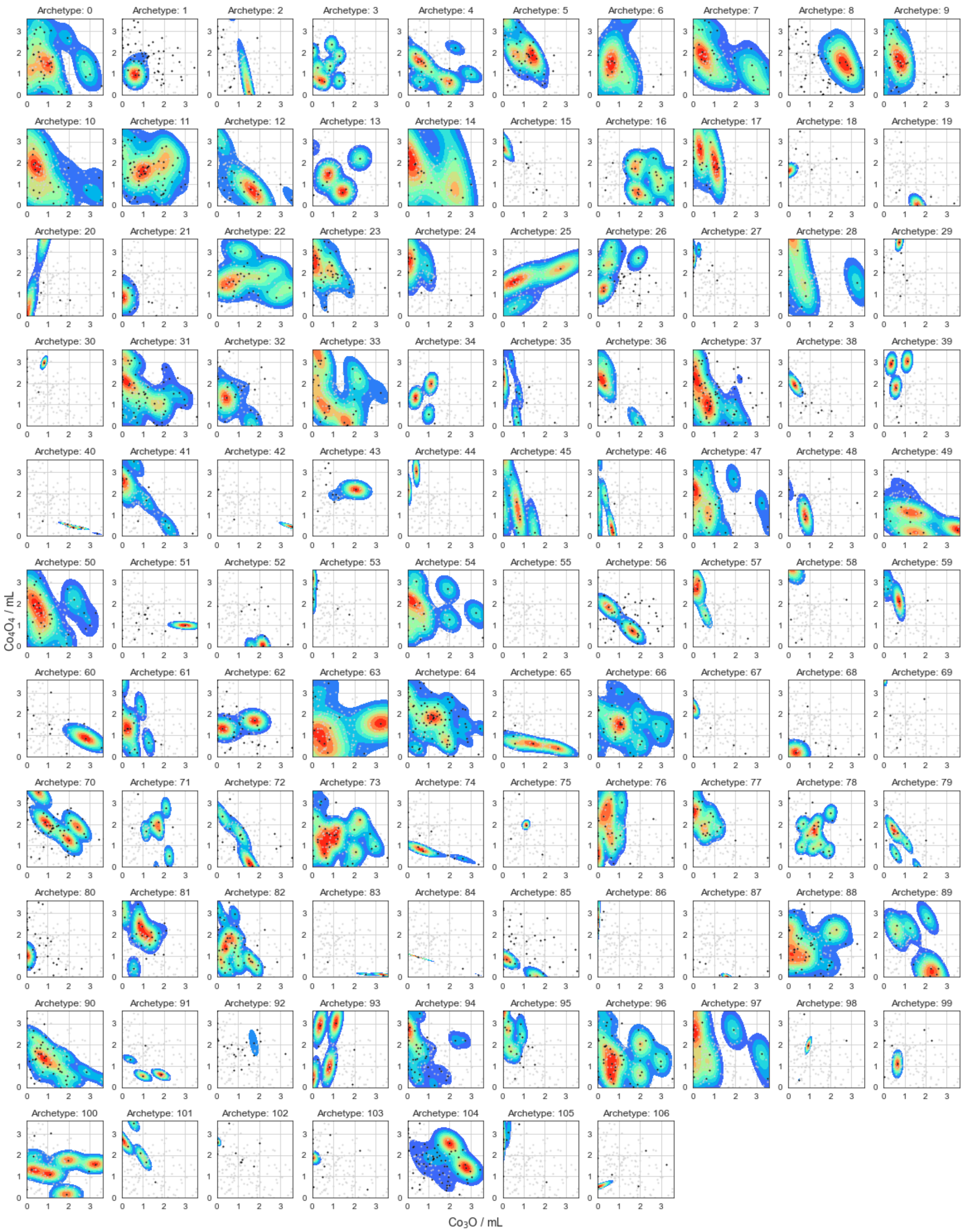

***Figure S95**: Kernel density estimation weighted by component score for each Common Component Warm Conditions archetype. Correlations shown between the volumes of clusters: {Co_3O} and {Co_4O_4}. Points with a non-negligible component score for the archetype are coloured black, and the remainder of the dataset shown in light grey. Unweighted kernel density estimates are shown for each variable on the diagonal.*

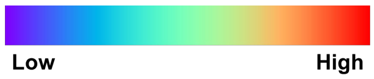

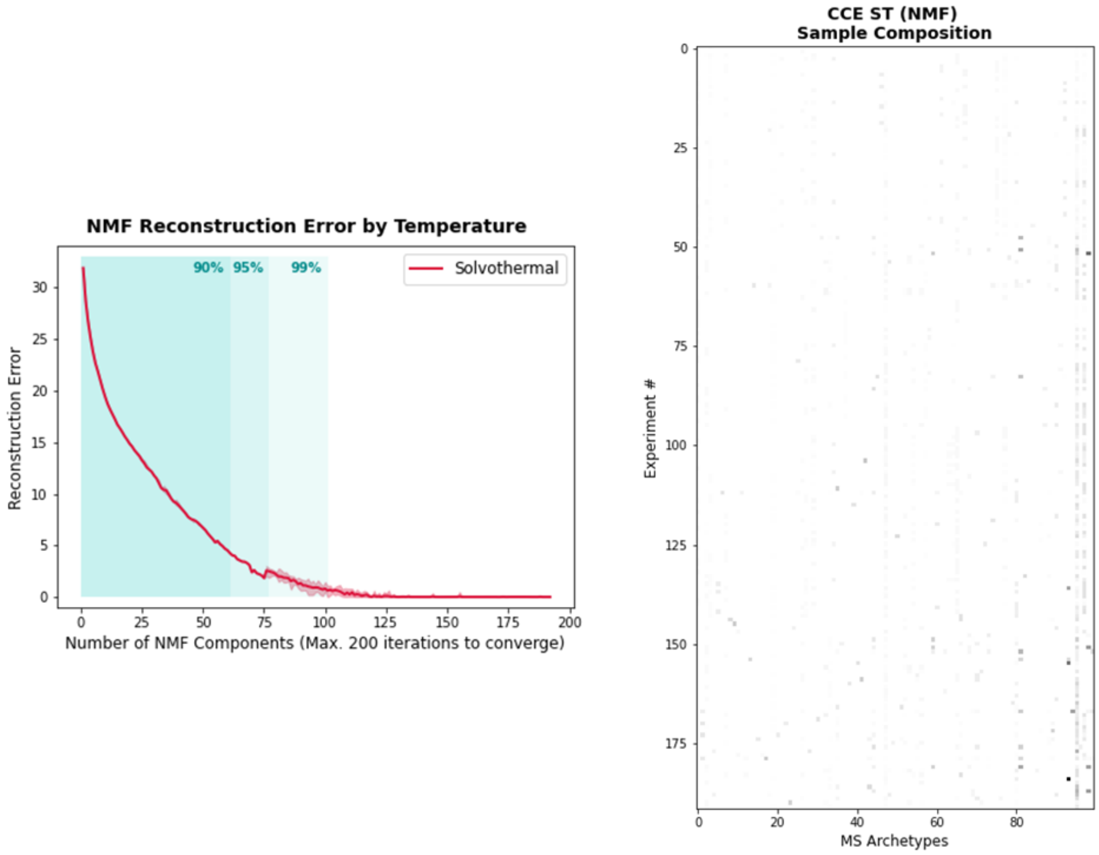

Figure S96: *Minimisation of reconstruction error with increasing component number of NMF components for the Common Component Exploration Solvothermal conditions (left). Resulting NMF W matrix with 100 components (right).*

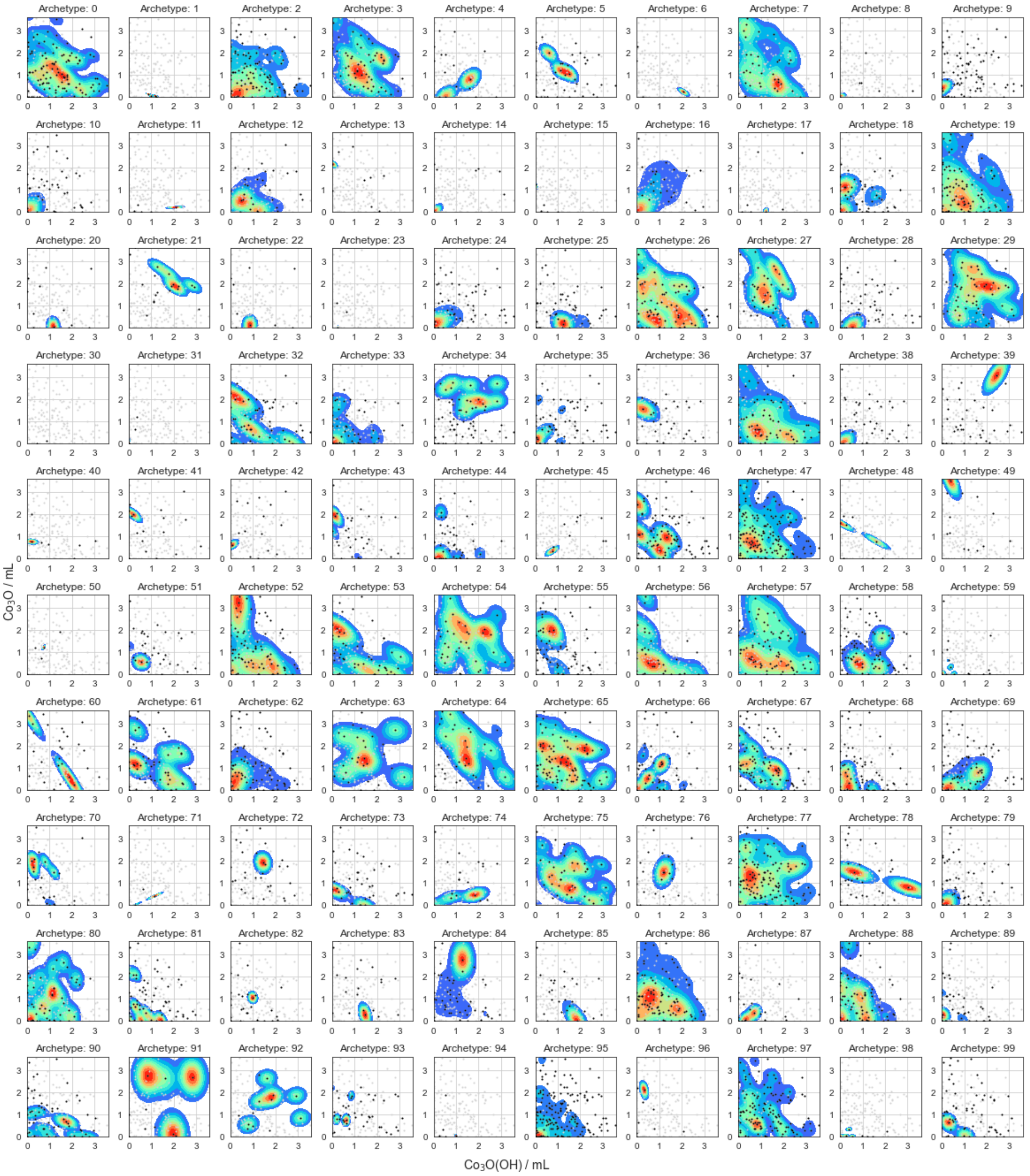

***Figure S97**: Kernel density estimation weighted by component score for each Common Component Solvothermal Conditions archetype. Correlations shown between the volumes of clusters: {$Co_3O(OH)$} and {Co_3O}. Points with a non-negligible component score for the archetype are coloured black, and the remainder of the dataset shown in light grey. Unweighted kernel density estimates are shown for each variable on the diagonal.*

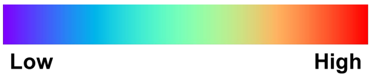

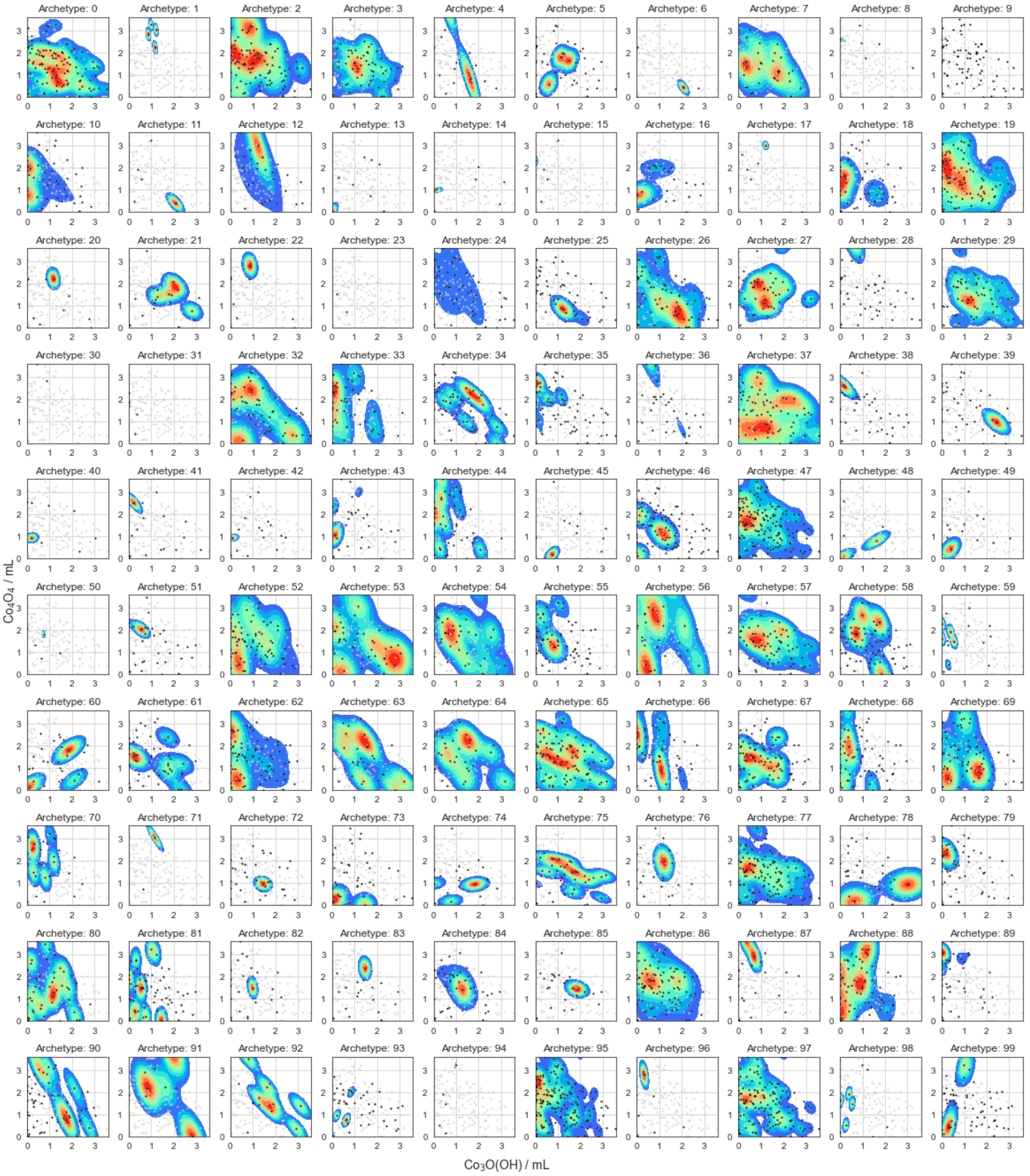

***Figure S98**: Kernel density estimation weighted by component score for each Common Component Solvothermal Conditions archetype. Correlations shown between the volumes of clusters: {$Co_3O(OH)$} and {Co_4O_4}. Points with a non-negligible component score for the archetype are coloured black, and the remainder of the dataset shown in light grey. Unweighted kernel density estimates are shown for each variable on the diagonal.*

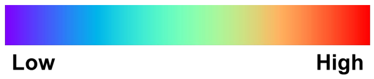

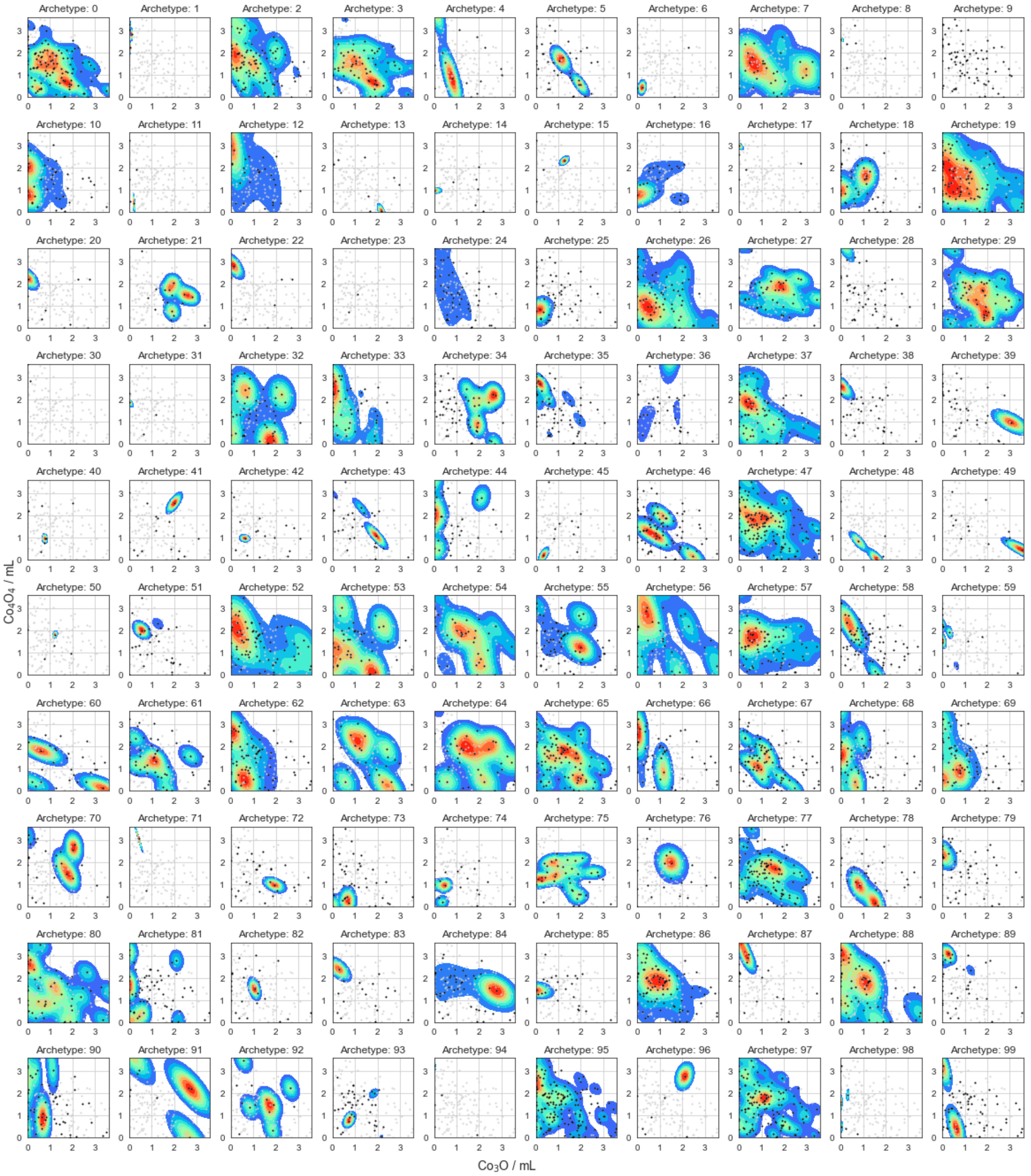

Figure S99: *Kernel density estimation weighted by component score for each Common Component Sovlothermal Conditions archetype. Correlations shown between the volumes of clusters: {Co_3O} and {Co_4O_4}. Points with a non-negligible component score for the archetype are coloured black, and the remainder of the dataset shown in light grey. Unweighted kernel density estimates are shown for each variable on the diagonal.*

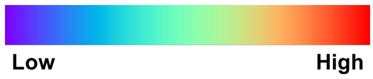

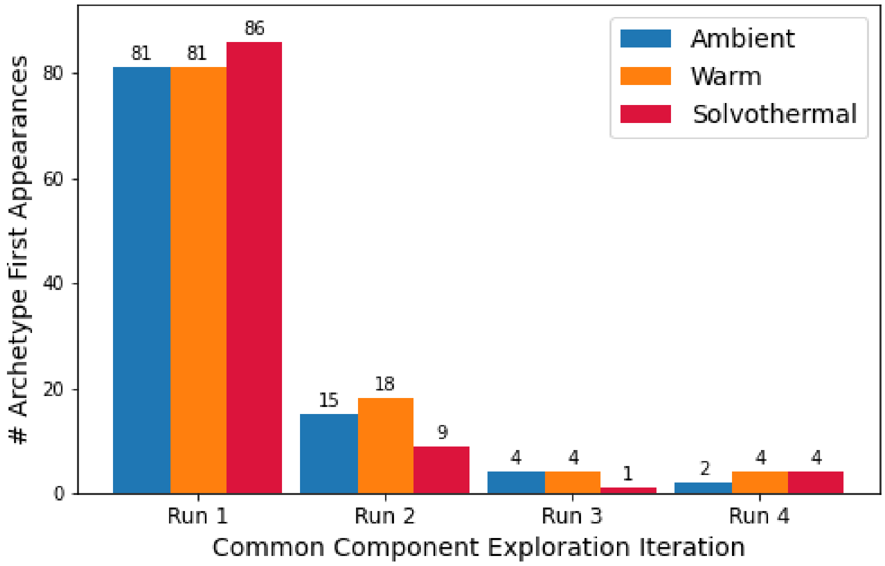

***Figure S100**: Number of archetypes originating in each Run in the Common Component Exploration.*

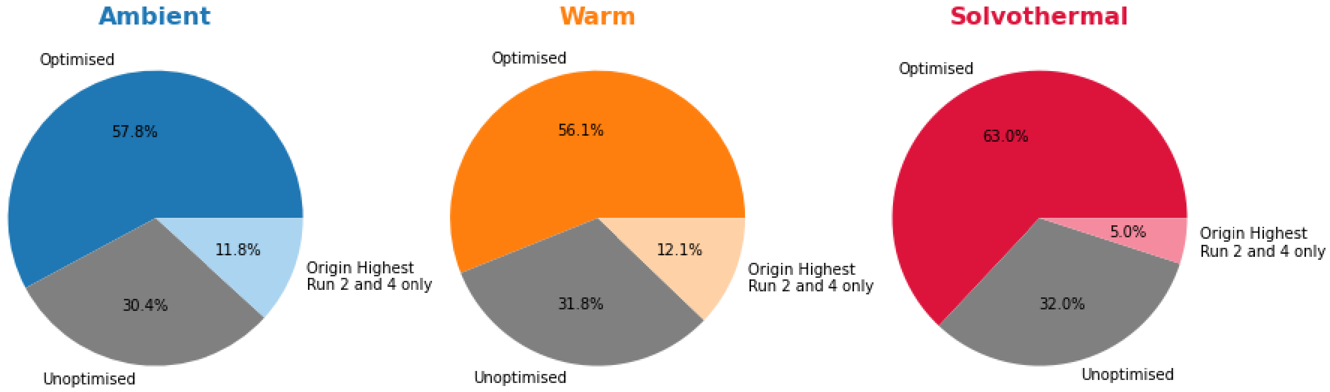

***Figure 101**: Pi charts indicating the outcomes for archetypes. Optimised archetypes have their highest value in the optimiser-led Runs 2 or 4, but originate in an earlier run.*

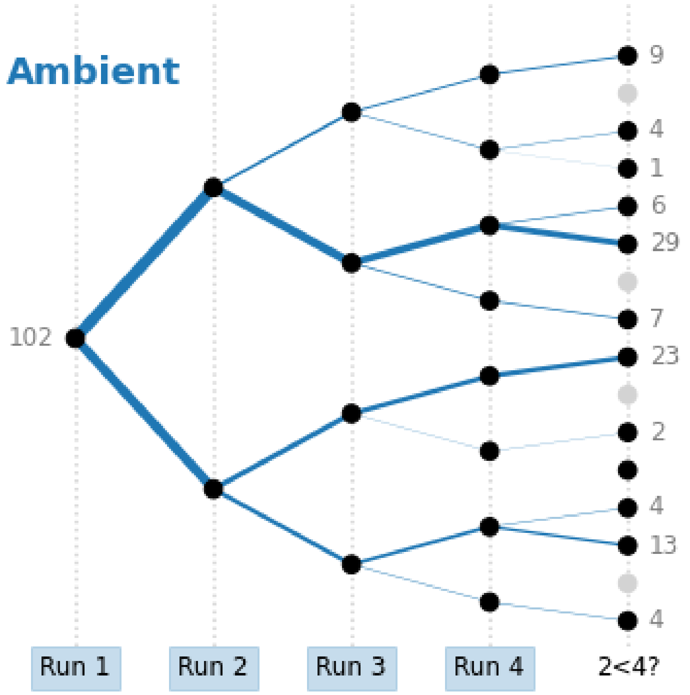

Figure S102*: Graph indicating the patterns in relative prevalence of archetypes throughout the Common Component Exploration under Ambient conditions. Thickness of edges corresponds to number of archetypes. Upward sloping edges correspond to increasing prevalence between Runs 1 to 4, and to the prevalence in Run 2 being less than Run 4 in the rightmost column.*

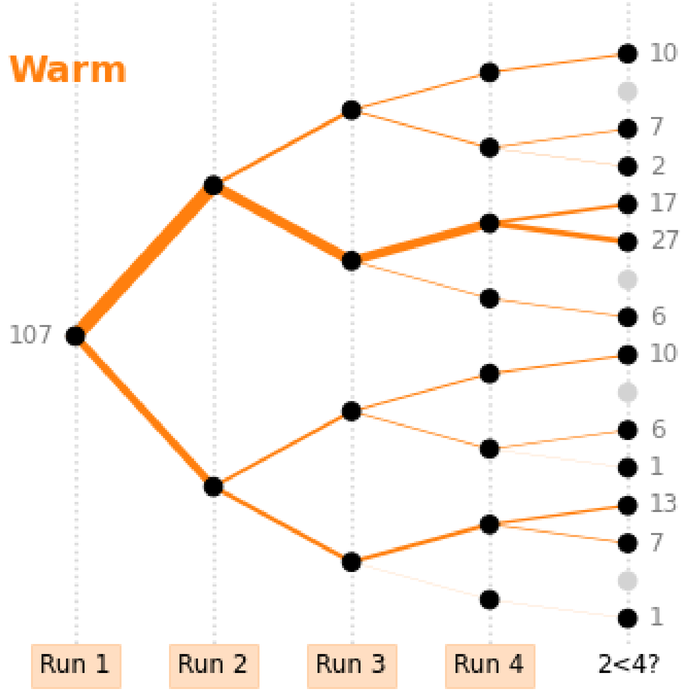

Figure S103*: Graph indicating the patterns in relative prevalence of archetypes throughout the Common Component Exploration under Warm conditions. Thickness of edges corresponds to number of archetypes. Upward sloping edges correspond to increasing prevalence between Runs 1 to 4, and to the prevalence in Run 2 being less than Run 4 in the rightmost column.*

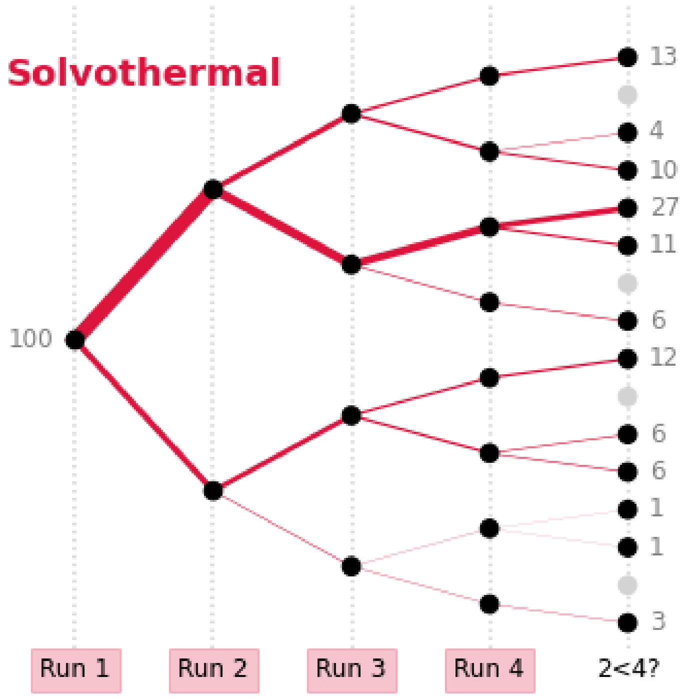

Figure S104: *Graph indicating the patterns in relative prevalence of archetypes throughout the Common Component Exploration under Solvothermal conditions. Thickness of edges corresponds to number of archetypes. Upward sloping edges correspond to increasing prevalence between Runs 1 to 4, and to the prevalence in Run 2 being less than Run 4 in the rightmost column.*

6.12 Dataset Analysis of the Unique Peak Data for the Isostructural Exploration

6.12.1 Dimensionality Reduction by Iteration

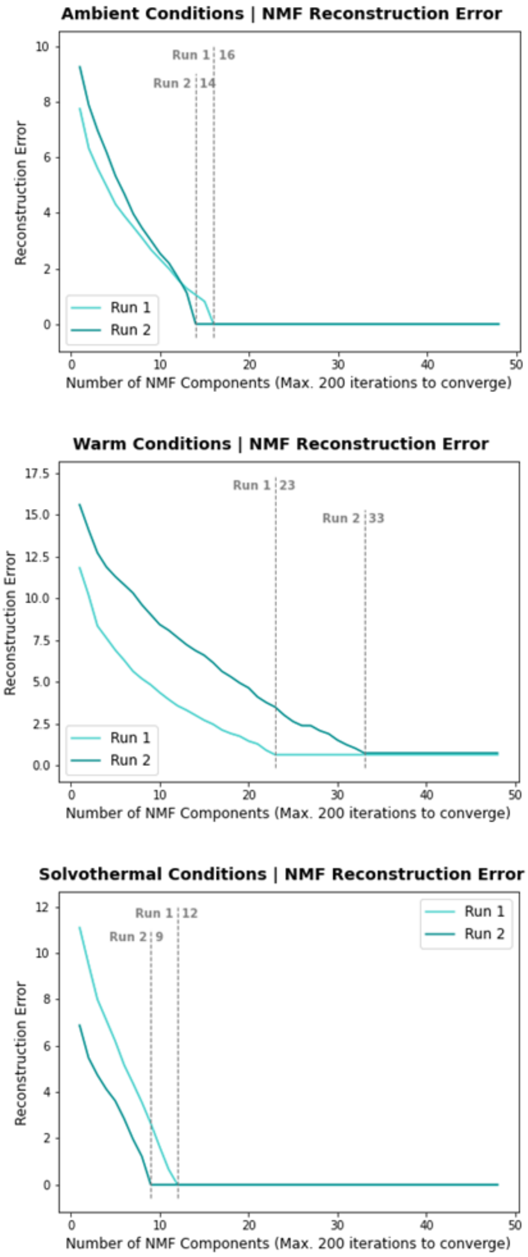

***Figure S105**: Graphs indicating how the error in reconstruction of the dataset is minimised by increasing the rank of the low-rank NMF approximation. Grey dotted lines indicate the number of components at which the minimal value is first reached for each trendline, and are labelled with the Run number and the number of components to make this explicit.*

6.12.2 Dimensionality Reduction by Temperature

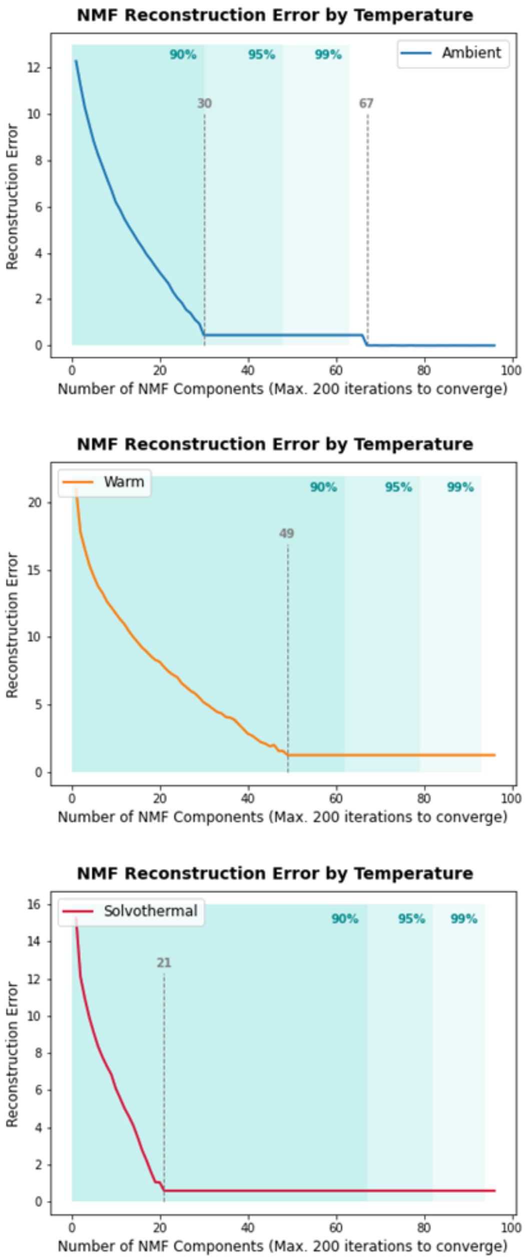

***Figure S106**: Minimisation of reconstruction errors with increasing component number of NMF components for the Isostructural Exploration, split by temperature. Note the unusually long plateau above minimum reoncstruction error for the Ambient condition.*

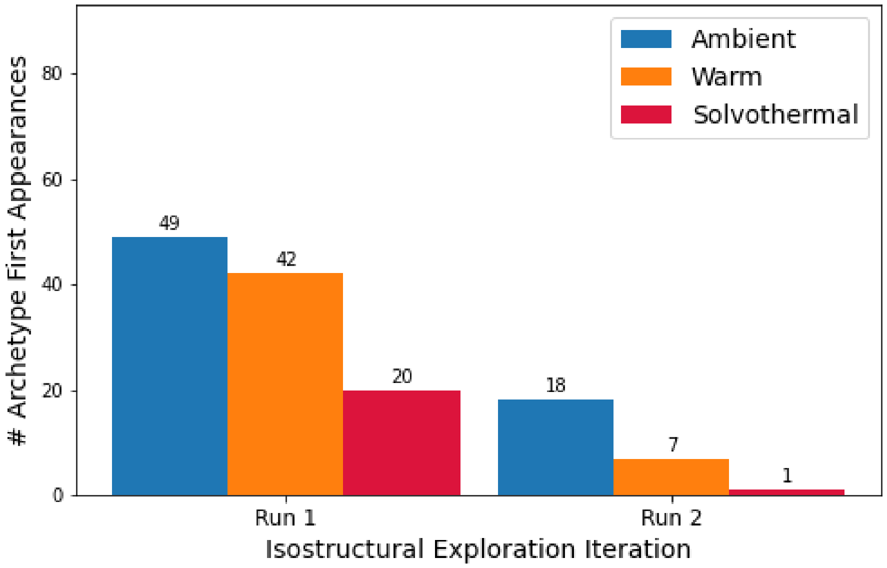

Figure S107: *Number of archetypes originating in each Run in the Isostructural Exploration.*

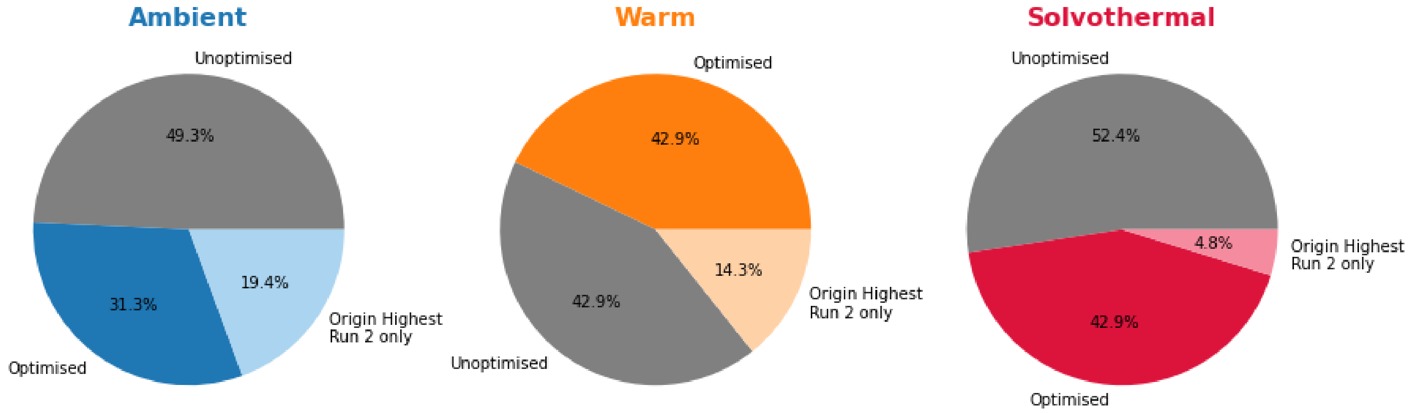

Figure 108: *Pi charts indicating the outcomes for archetypes. Optimised archetypes have their highest value in the optimiser-led Runs 2 or 4, but originate in an earlier run.*

6.13 Chemical Validation of NMF Archetypes

6.13.1 Correlation with Archetypes by Temperature for entire Solvothermal Dataset

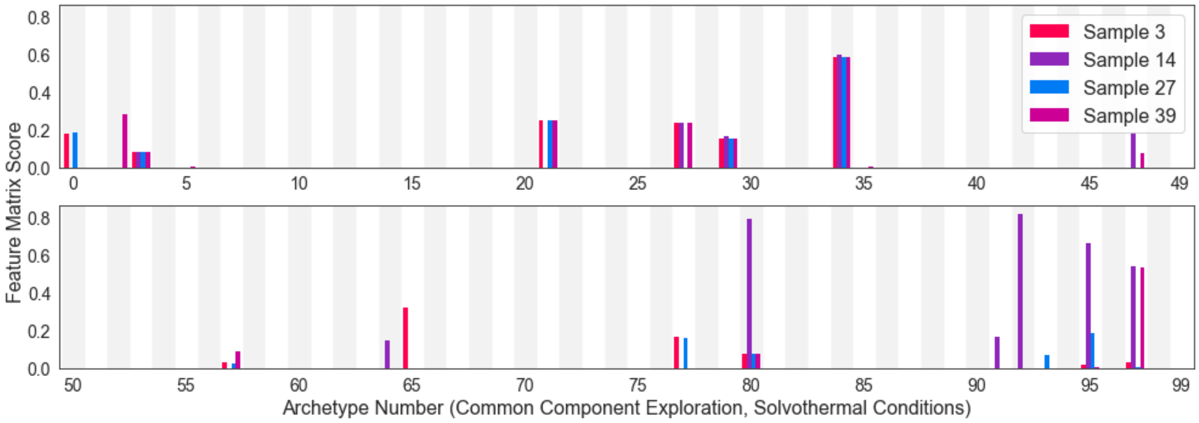

***Figure S109**: Component score for the four samples known to contain [Co(Ac$_2$O)$_2$(OAc)]$^{2+}$ (sample numbers 3, 14, 27, and 39) for each component in the expanded archetype set for all Solvothermal reactions (as opposed to just the first iteration).*

As can be seen from the bar chart above, the four instances of crystallised [Co(Ac$_2$O)$_2$(OAc)]$^{2+}$ all show high and comparable component scores for Archetype 34 based on NMF decomposition of the entire Solvothermal dataset.

Interrogation of this archetype reveals a high selectivity for known samples of the desired species over the first iteration, and highlights several samples in later iterations which likely also contain the species of interest.

The area of the process space highlighted by the weighted kernel density estimations for Archetype 34 shows good agreement with those indicated by the archetypes shown to correspond well when only the first iteration of the solvothermal dataset is decomposed (above).

Comparison of the actual MS spectra for these species show a very similar result for each sample with a high component score for Archetype 34, and the unique peaks highlighted may be assigned to likely products of electrospray ionisation. Notably, peaks with m/z ≈ 354 likely correspond to the reduced [Co^{II}(Ac$_2$O)$_2$(OAc)]$^+$ ion flying with a molecule of methanol (i.e. [M+CH$_3$OH+e$^-$]$^+$). Despite the low voltage (50 V) used during ionisation, the reduction of Co(III) to Co(II) is highly favourable (for Co^{3+} + e^- → Co^{2+}, E^O = +1.82 V), and likely still proceeds.[30]

Comparable scores across the four known samples are also seen for Archetype 29, although the lower component score appears to show less of a selectivity for this species than Archetype 34. Individual component scores for each sample and weighted kernel density estimations across the search space are also included below for Archetype 29 to demonstrate the implications of this.

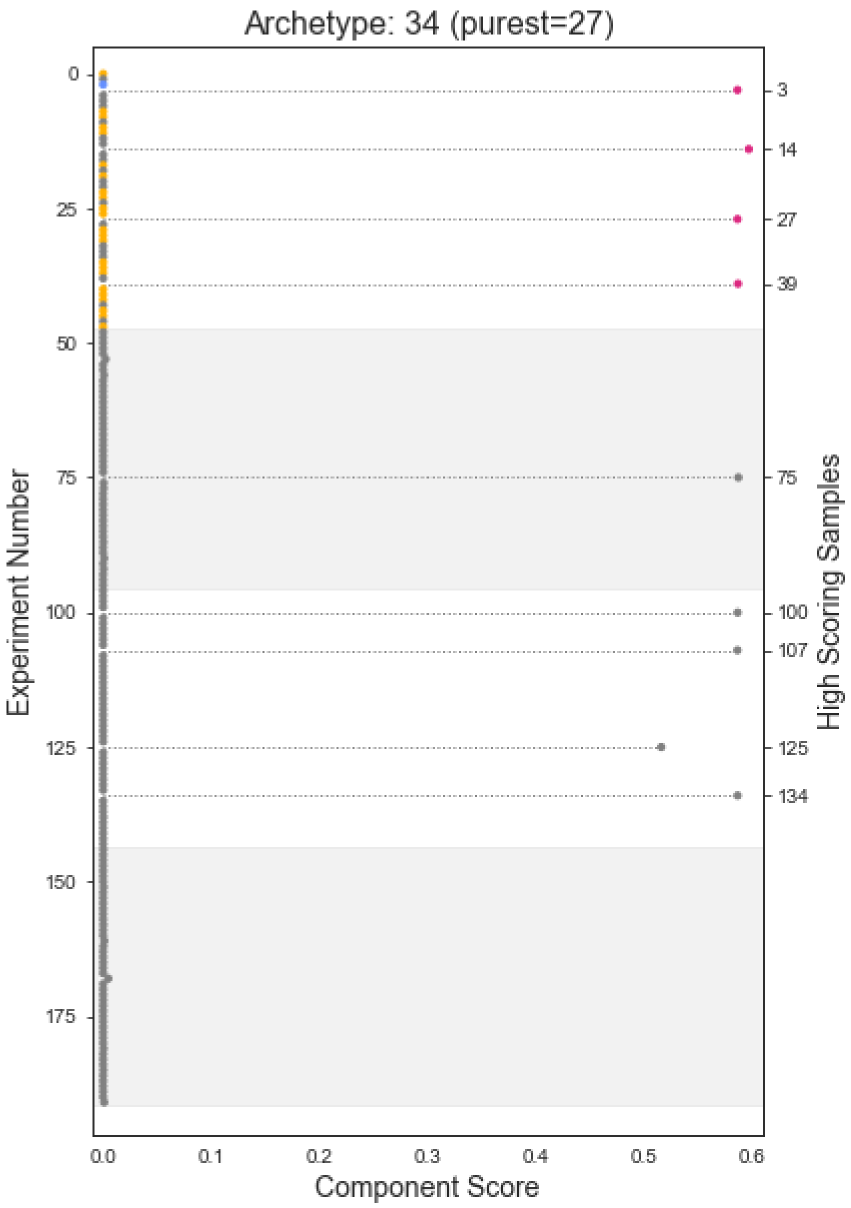

***Figure S110**: Component score vs. sample number for Solvothermal Archetype 34. Points in the first iteration are coloured according to the crystals that were isolated from them: yellow for [Co(Ac_2O)$_3$][Co(CN)$_6$], pink for [Co(Ac_2O)$_2$OAc]X_2, blue for* trans-*[Co(Ac_2O)$_2$(OH)$_2$]PF_6, and grey for no isolable species.*

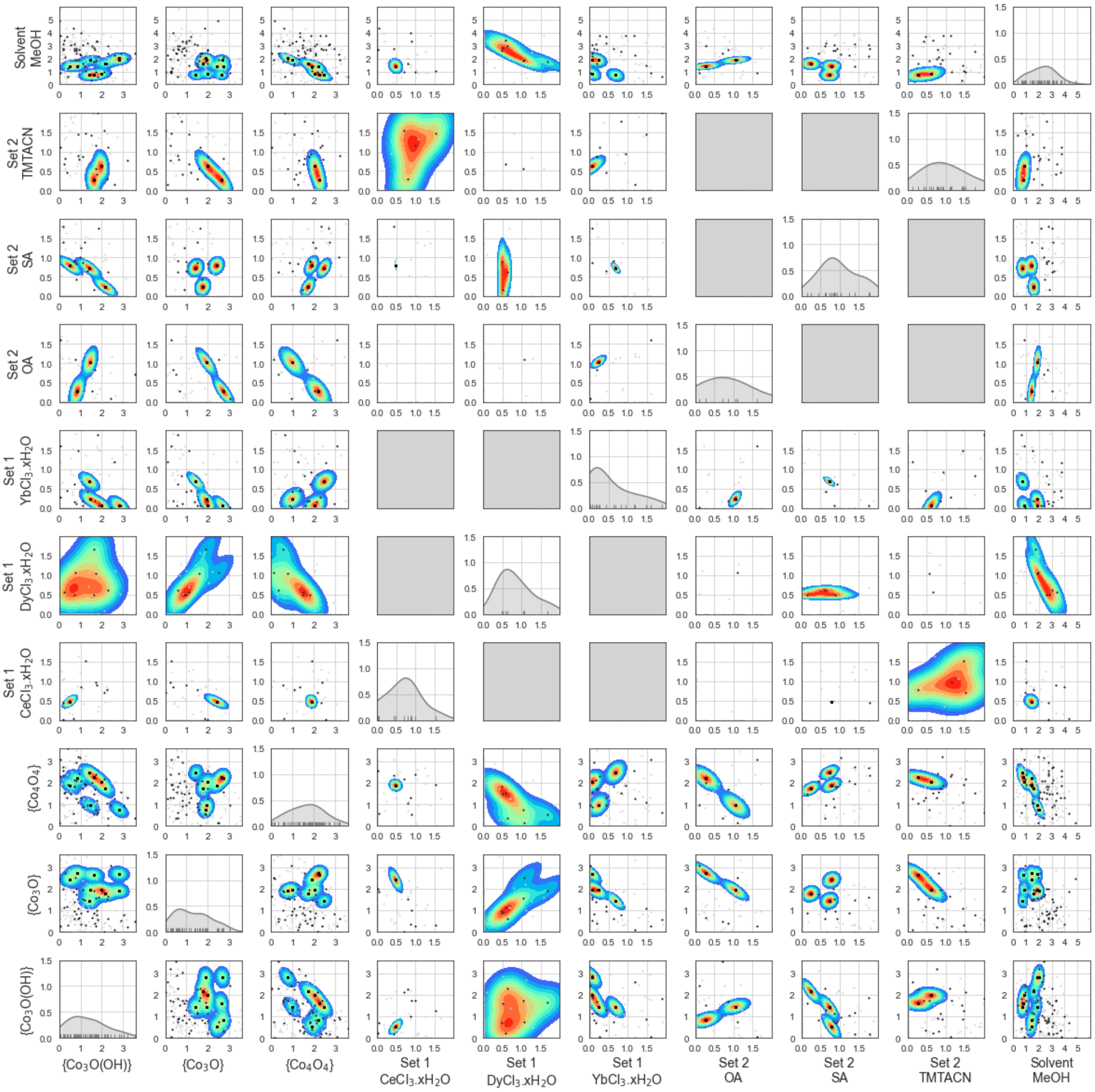

Figure S111: *Kernel density estimation weighted by component score to indicate the prevalence of Solvothermal Archetype 34 across the search space. Axes plotted as volumes of reagent stock solution. Points with a non-negligible component score for the archetype are coloured black, and the remainder of the dataset shown in light grey. Unweighted kernel density estimates are shown for each variable on the diagonal.*

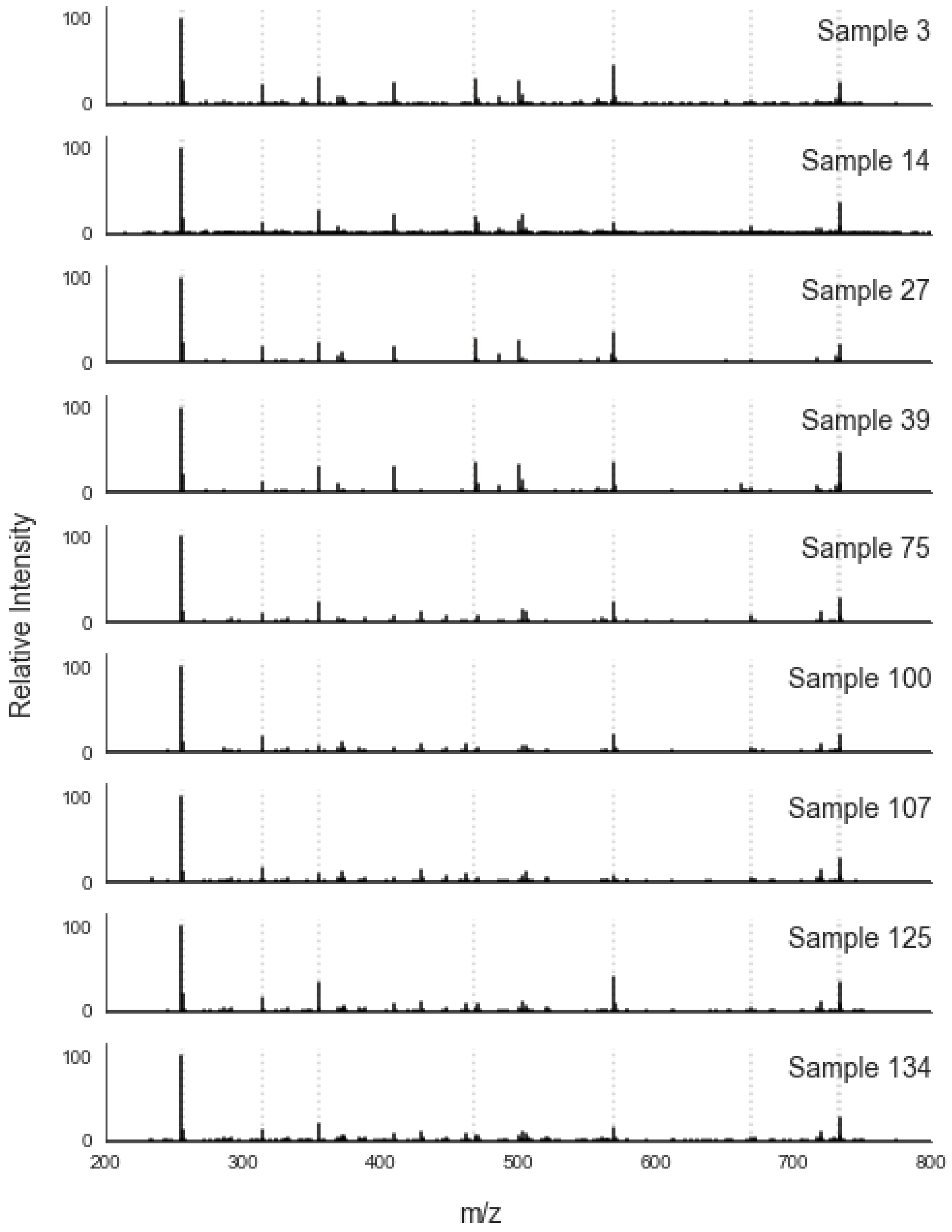

***Figure S112**: MS spectra from all samples with a high component score in Archetype 34. Light grey dotted lines correspond to unique peaks characteristic of Solvothermal Archetype 34.*

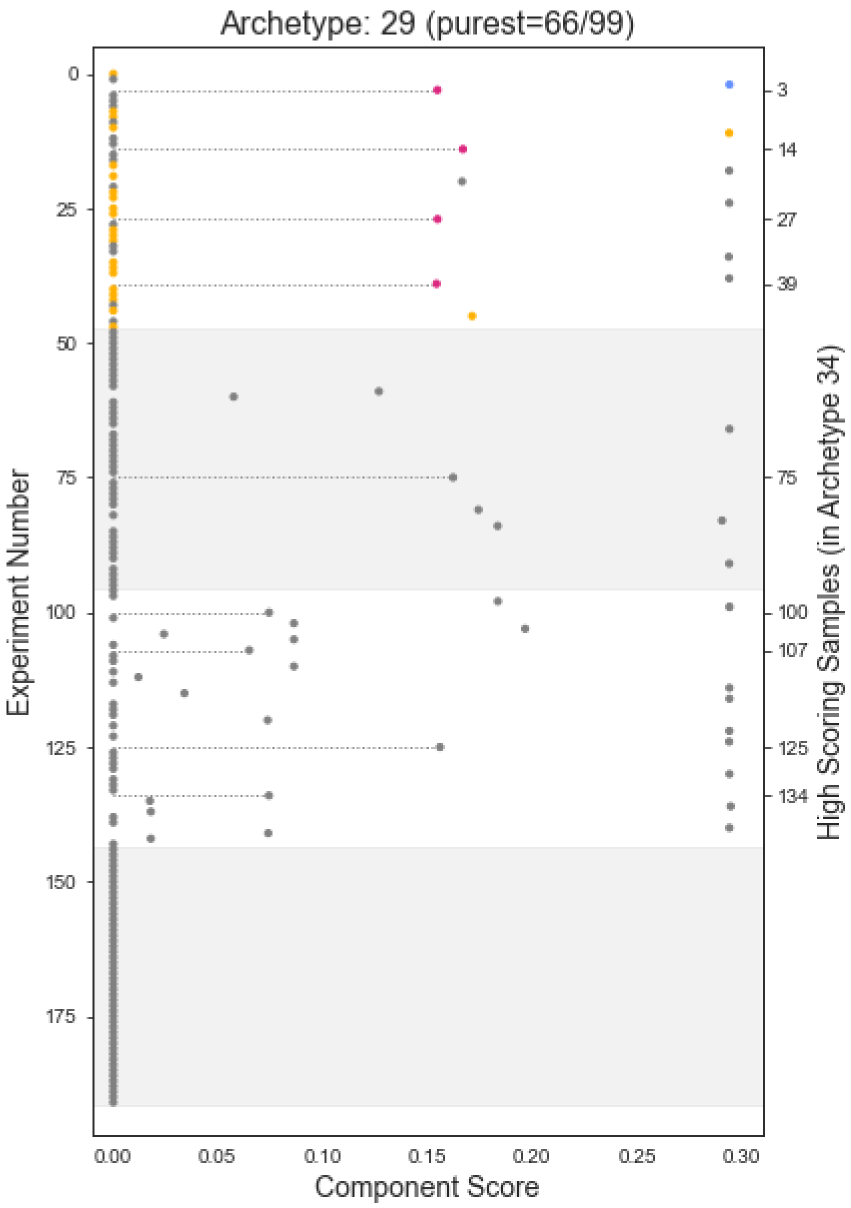

***Figure S113**: Component score vs. sample number for Solvothermal Archetype 29. Points in the first iteration are coloured according to the crystals that were isolated from them: yellow for [Co(Ac_2O)$_3$][Co(CN)$_6$], pink for [Co(Ac_2O)$_2$OAc]X_2, blue for* trans-*[Co(Ac_2O)$_2$(OH)$_2$]PF_6, and grey for no isolable species.*

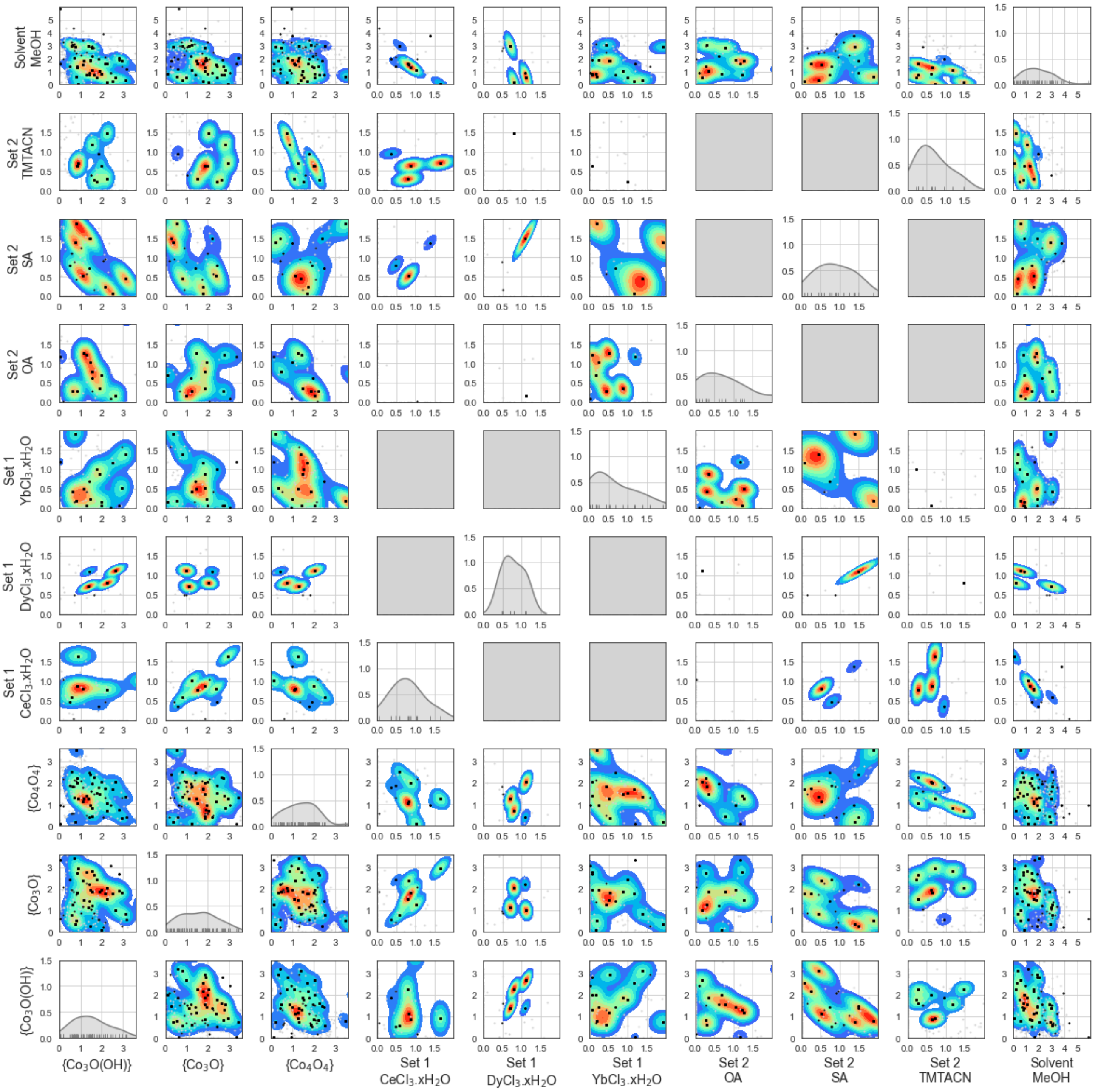

***Figure S114**: Kernel density estimation weighted by component score to indicate the prevalence of Solvothermal Archetype 29 across the search space. Axes plotted as volumes of reagent stock solution. Points with a non-negligible component score for the archetype are coloured black, and the remainder of the dataset shown in light grey. Unweighted kernel density estimates are shown for each variable on the diagonal.*

6.14 Crystallographic Data

6.14.1 Data for novel species

Table S9: *Crystal data and structure refinement details for Co(III) anhydride complexes from the Common Component Exploration.*

**Compound code**	**$[Co(Ac_2O)_2OAc](OAc)_2$**	**$[Co(Ac_2O)_2(OH)_2]PF_6.CH_3OH$**	**$[Co(Ac_2O)_3][Co(CN)_6]$**
**Empirical formula**	C14 H21 Co O12	C9 H20 Co F6 O9 P	C18 H18 Co2 N6 O9
**Formula weight**	440.24	476.15	580.24
**Temperature (K)**	150(2)	150(2)	150(2)
**Crystal system**	Monoclinic	Tetragonal	Trigonal
**Space group**	I 2/a	I 4/m	R 3 :H
***a* (Å)**	8.2147(4)	9.7691(3)	14.3994(13)
***b* (Å)**	22.2483(9)	9.7691(3)	14.3994(13)
***c* (Å)**	18.7369(12)	19.0174(7)	10.8295(11)
**α (°)**	90	90	90
**β (°)**	100.601(5)	90	90
**γ (°)**	90	90	120
**Volume ($Å^3$)**	3366.0(3)	1814.93(13)	1944.6(4)
**Z**	8	4	3
**Density calculated (Mg/m^3)**	1.737	1.743	1.486
**Absorption coefficient (mm^{-1})**	1.087	1.132	1.334
***F*(000)**	1824	968	882
**θ range for data collection (°)**	2.684 to 25.993	2.344 to 25.984	2.491 to 25.988
**Index ranges**	-10 <= *h* <= 10	-12 <= *h* <= 12	-17 <= *h* <= 15
	-27 <= *k* <= 27	-12 <= *k* <= 11	-17 <= *k* <= 16
	-23 <= *l* <= 22	-23 <= *l* <= 22	-13 <= *l* <= 12
**Reflections collected**	14178	6341	3420
**Independent reflections**	3300	923	1576
	[R(int) = 0.0351]	[R(int) = 0.0209]	[R(int) = 0.0416]
**Completeness (θ = 25.242)**	99.9 %	99.9 %	100.0 %
**Data/restraints/parameters**	3300 / 1 / 212	923 / 18 / 68	1576 / 102 / 153
**Goodness-of-fit on F^2**	1.043	1.162	1.045
**Final *R* indices [I>2σ(I)]**	R1 = 0.0765	R1 = 0.0710	R1 = 0.0592
	wR2 = 0.2114	wR2 = 0.2225	wR2 = 0.1576
***R* indices (all data)**	R1 = 0.0804	R1 = 0.0766	R1 = 0.0946
	wR2 = 0.2140	wR2 = 0.2355	wR2 = 0.1788
**Max/min Δρ (e $Å^{-3}$)**	1.87 and -0.80	1.782 and -0.842	0.721 and -0.379

Table S10: *Unit cell parameters for the {M_{10}} wheel from the Isostructural Exploration. Multimetallic nature has been determined by IR, but exact composition in each case is unknown – solved assuming Fe, but is some combination of Cr, Fe, Mn, and Co.*

**Compound code**	**[M(OMe)$_2$OAc]$_{10}$**
**Empirical formula**	C40 H90 M10 O40
**Formula weight**	1769.61
a **(Å)**	17.4348(10)
b **(Å)**	15.8754(11)
c **(Å)**	26.1523(13)
**α (°)**	90
**β (°)**	98.907(6)
**γ (°)**	90
**Volume (Å^3)**	7151.3(7)
**Z**	4

6.14.2 Bond Valence Calculations

Bond valence calculations were performed for each bond using the bond distance (R) measured and empirical parameters R0 and B: bond valence = EXP((R0-R)/B). BVS for each metal centre was then summed from all bond valences of the bonds listed. The parameters R0 and B were taken from Gagne & Hawthorne.[31]

Parameter list:

Bond	R0	B
Co(3+)-O	1.655	0.364
Co(3+)-C	1.634	0.376

$[Co(Ac_2O)_2OAc](OAc)_2$

			Co3+
Co	O8	1.881	0.537
Co	O6	1.887	0.529
Co	O5	1.899	0.512
Co	O3	1.925	0.476
Co	O2	1.946	0.450
Co	O1	1.953	0.441
			2.945

$[Co(Ac_2O)_2(OH)_2]PF_6.CH_3OH$

			Co3+
Co1	O2	1.901	0.509
Co1	O2	1.901	0.509
Co1	O2	1.901	0.509
Co1	O2	1.901	0.509
Co1	O1	1.979	0.411
Co1	O1	1.979	0.411
			2.856

$[Co(Ac_2O)_3][Co(CN)_6]$

			Co3+
Co1	O2	1.906	0.502
Co1	O2	1.906	0.502
Co1	O2	1.906	0.502
Co1	O1	1.926	0.475
Co1	O1	1.926	0.475
Co1	O1	1.926	0.475
			2.930
Co2	C6	1.860	0.543
Co2	C6	1.860	0.543
Co2	C6	1.860	0.543
Co2	C7	1.886	0.506
Co2	C7	1.886	0.506
Co2	C7	1.886	0.506
			3.147

6.14.3 Data for previously reported species

***Table S11**: List of compounds crystallised where the unit cell check indicated the species present was a known compound. Literature references containing the crystallographic data of these species are provided.*

Molecular Formula	Abbreviation (if applicable)	Reference for Crystallographic Parameters
**$[Co_3O(OMe)(OAc)_5(pyr)_3]PF_6$**	$\{Co_3O(OMe)\}$	(Sumner; 1988) [17]
**$[Co_3O(OH)_2(OAc)_3(pyr)_5](PF_6)_2$**	$\{Co_3O(OH)_2\}$	(Sumner; 1988) [17]
**$[Co_2(OH)_2(OAc)_3(pyr)_4]PF_6$**	$\{Co_2(OH)_2\}$	(Sumner; 1988) [17]
**$[Co_5(OH)_2(O_2CMe)_8.2H_2O]_n$**	N/A	(Kuhlman *et al.*; 1999) [32]
**$[Fe(OMe)_2OAc]_{10}$**	$\{Fe_{10}\}$	(Parsons *et al.*; 1996) [33]<br>(Stamatatos *et al.*; 2008) [34]

7 References

1. Angelone, D.; Hammer, A. J. S.; Rohrbach, S.; Krambeck, S.; Granda, J. M.; Wolf, J.; Zalesskiy, S.; Chisholm, G.; Cronin, L., Convergence of multiple synthetic paradigms in a universally programmable chemical synthesis machine. *Nature Chemistry* **2021,** *13* (1), 63-69.
2. Steiner, S.; Wolf, J.; Glatzel, S.; Andreou, A.; Granda, J. M.; Keenan, G.; Hinkley, T.; Aragon-Camarasa, G.; Kitson, P. J.; Angelone, D.; Cronin, L., Organic synthesis in a modular robotic system driven by a chemical programming language. *Science* **2019,** *363* (6423), eaav2211.
3. UofGlasgow Glassblowing (School of Chemistry). (accessed May).
4. Asche, S.; Cooper, G. J. T.; Keenan, G.; Mathis, C.; Cronin, L., A robotic prebiotic chemist probes long term reactions of complexifying mixtures. *Nature Communications* **2021,** *12* (1), 3547.
5. Mehr, S. H. M.; Craven, M.; Leonov, A. I.; Keenan, G.; Cronin, L., A universal system for digitization and automatic execution of the chemical synthesis literature. *Science* **2020,** *370* (6512), 101-108.
6. CroninGroup ChemIDE. https://croningroup.gitlab.io/chemputer/xdlapp/ (accessed 26th April 2022).
7. Salley, D. S.; Keenan, G. A.; Long, D.-L.; Bell, N. L.; Cronin, L., A Modular Programmable Inorganic Cluster Discovery Robot for the Discovery and Synthesis of Polyoxometalates. *ACS Central Science* **2020,** *6* (9), 1587-1593.
8. Sarkar, A.; Mahapatra, S., Synthesis of All Crystalline Phases of Anhydrous Calcium Carbonate. *Crystal Growth & Design* **2010,** *10* (5), 2129-2135.
9. Vincent, J. B.; Chang, H. R.; Folting, K.; Huffman, J. C.; Christou, G.; Hendrickson, D. N., Preparation and physical properties of trinuclear oxo-centered manganese complexes of general formulation [Mn3O(O2CR)6L3]0,+ (R = methyl or phenyl; L = a neutral donor group) and the crystal structures of [Mn3O(O2CMe)6(pyr)3](pyr) and [Mn3O(O2CPh)6(pyr)2(H2O)].cntdot.0.5MeCN. *Journal of the American Chemical Society* **1987,** *109* (19), 5703-5711.
10. Boreham, C. J.; Broomhead, J. A.; Fairlie, D. P., A ^{195}Pt and ^{15}N N.M.R. study of the anticancer drug, *cis*-diammine-dichloroplatinum(II), and its hydrolysis and oligomerization products. *Australian Journal of Chemistry* **1981,** *34* (3), 659-664.
11. Shirgaonkar, I. Z.; Pandit, A. B., Degradation of aqueous solution of potassium iodide and sodium cyanide in the presence of carbon tetrachloride. *Ultrasonics Sonochemistry* **1997,** *4* (3), 245-253.
12. Miras, H. N.; Mathis, C.; Xuan, W.; Long, D.-L.; Pow, R.; Cronin, L., Spontaneous formation of autocatalytic sets with self-replicating inorganic metal oxide clusters. *Proceedings of the National Academy of Sciences* **2020,** *117* (20), 10699.
13. Standfest-Hauser, C. M.; Mereiter, K.; Schmid, R.; Kirchner, K., Some binding modes of 2-aminopyridine to ruthenium(ii) fragments. *Dalton Transactions* **2003,** (11), 2329-2334.
14. Erdemir, F.; Celepci, D. B.; Aktaş, A.; Gök, Y.; Kaya, R.; Taslimi, P.; Demir, Y.; Gulçin, İ., Novel 2-aminopyridine liganded Pd(II) N-heterocyclic carbene complexes: Synthesis, characterization, crystal structure and bioactivity properties. *Bioorganic Chemistry* **2019,** *91*, 103134.
15. Konakanchi, R.; Mallela, R.; Guda, R.; Kotha, L. R., Synthesis, characterization, biological screening and molecular docking studies of 2-aminonicotinaldehyde (ANA) and its metal complexes. *Research on Chemical Intermediates* **2018,** *44* (1), 27-53.
16. Johnson, M. K.; Powell, D. B.; Cannon, R. D., Vibrational spectra of carboxylato complexes—III. Trinuclear 'basic' acetates and formates of chromium(III), iron(III) and other transition metals. *Spectrochimica Acta Part A: Molecular Spectroscopy* **1981,** *37* (11), 995-1006.
17. Sumner, C. E., Interconversion of dinuclear and oxo-centered trinuclear cobaltic acetates. *Inorganic Chemistry* **1988,** *27* (8), 1320-1327.
18. Aromí, G.; Brechin, E. K., Synthesis of 3d Metallic Single-MoleculeMagnets. In *Single-Molecule Magnets and Related Phenomena*, Winpenny, R., Ed. Springer Berlin Heidelberg: Berlin, Heidelberg, 2006; pp 1-67.

19. Burke, R. W.; Diamondstone, B. I.; Velapoldi, R. A.; Menis, O., Mechanisms of the Liebermann-Burchard and Zak Color Reactions for Cholesterol. *Clinical Chemistry* **1974,** *20* (7), 794-801.
20. Felton, K. C.; Rittig, J. G.; Lapkin, A. A., Summit: Benchmarking Machine Learning Methods for Reaction Optimisation. *Chemistry–Methods* **2021,** *1* (2), 116-122.
21. Bergstra, J.; Bengio, Y., Random search for hyper-parameter optimization. *J. Mach. Learn. Res.* **2012,** *13* (null), 281–305.
22. Lee, A. pyDOE: Randomized Designs (Latin-Hypercube). https://pythonhosted.org/pyDOE/randomized.html#latin-hypercube (accessed 29th April 2022).
23. Rasmussen, C. E.; Williams, C. K. I., Regression. In *Gaussian Processes for Machine Learning*, MIT Press: Massachusetts Institute of Technology, 2006; pp 7-31.
24. Louppe, G.; Kumar, M.; Nahrstaedt, H. Bayesian optimization with skopt. https://scikit-optimize.github.io/stable/auto_examples/bayesian-optimization.html (accessed 28th June 2022).
25. Carlen, S.; Nahrstaedt, H. Exploration vs exploitation. (accessed 28th June 2022).
26. Garnett, R. In *Bayesian Optimisation*, CSE 515T: Bayesian Methods in Machine Learning, Washington University, St Louis, Washington University, St Louis, 2015.
27. Ahmed, M. O.; Prince, S. Bayesian optimization. https://www.borealisai.com/en/blog/tutorial-8-bayesian-optimization/ (accessed 28th June 2022).
28. Chevalier, C.; Ginsbourger, D. In *Fast Computation of the Multi-Points Expected Improvement with Applications in Batch Selection*, Learning and Intelligent Optimization, Berlin, Heidelberg, 2013//; Nicosia, G.; Pardalos, P., Eds. Springer Berlin Heidelberg: Berlin, Heidelberg, 2013; pp 59-69.
29. Lee, D. D.; Seung, H. S., Learning the parts of objects by non-negative matrix factorization. *Nature* **1999,** *401* (6755), 788-791.
30. McIndoe, J. S.; Vikse, K. L., Assigning the ESI mass spectra of organometallic and coordination compounds. *Journal of Mass Spectrometry* **2019,** *54* (5), 466-479.
31. Gagné, O. C.; Hawthorne, F. C., Comprehensive derivation of bond-valence parameters for ion pairs involving oxygen. *Acta Crystallographica Section B* **2015,** *B71*, 562-578.
32. Kuhlman, R.; Schimek, G. L.; Kolis, J. W., An Extended Solid from the Solvothermal Decomposition of Co(Acac)3: Structure and Characterization of Co5(OH)2(O2CCH3)8·2H2O. *Inorganic Chemistry* **1999,** *38* (1), 194-196.
33. Parsons, S.; Solan, G. A.; Winpenny, R. E. P.; Benelli, C., Ferric Wheels and Cages: Decanuclear Iron Complexes with Carboxylato and Pyridonato Ligands. *Angewandte Chemie International Edition in English* **1996,** *35* (16), 1825-1828.
34. Stamatatos, T. C.; Christou, A. G.; Mukherjee, S.; Poole, K. M.; Lampropoulos, C.; Abboud, K. A.; O'Brien, T. A.; Christou, G., High-Yield Syntheses and Reactivity Studies of Fe10 "Ferric Wheels": Structural, Magnetic, and Computational Characterization of a Star-Shaped Fe8 Complex. *Inorganic Chemistry* **2008,** *47* (19), 9021-9034.
